# Stereoselective Synthesis and Antimicrobial Studies of *Allo*-Gibberic Acid-Based 2,4-Diaminopyrimidine Chimeras

**DOI:** 10.3390/ph18020168

**Published:** 2025-01-26

**Authors:** Dima Depp, Noémi Regina Sebők, András Szekeres, Zsolt Szakonyi

**Affiliations:** 1 Institute of Pharmaceutical Chemistry, University of Szeged, Eötvös Utca 6, H-6720 Szeged, Hungary; dima.depp@hotmail.com; 2 Department of Microbiology, University of Szeged, Közép Fasor 52, H-6726 Szeged, Hungary; sebok.noemi024@gmail.com (N.R.S.); andras.j.szekeres@gmail.com (A.S.)

**Keywords:** GA3, diterpene, *allo*-gibberic acid, 2,4-diaminopyrimidine, stereoselective, antibacterial, antifungal, *P. aeruginosa*

## Abstract

**Background**: Gibberellins (GAs) are a family of tetracyclic *ent*-kaurenoid diterpenes found widely in several commonly used plants. Besides agricultural applications, gibberellins play an important role in the synthesis of bioactive compounds, especially those with antiproliferative and antibacterial activity. **Methods**: A series of gibberellic acid-based 2,4-diaminopyrimidines was designed and synthesized from commercially available gibberellic acid. The antimicrobial activity of the prepared compounds was also explored in *B. subtilis*, *S. aureus*, *E. coli,* and *P. aeruginosa* bacteria, as well as in *C. krusei* and *C. albicans* fungi. **Results**: The treatment of gibberellic acid with hydrochloric acid under reflux conditions resulted in aromatization followed by rearrangement to form *allo*-gibberic acid. The key intermediate azido alcohol was prepared according to the literature methods. The second key intermediate azidotriol was synthesized by the stereoselective dihydroxylation of the allylic function by the osmium (VIII)-tetroxide/NMO system. Starting from azide intermediates, click reactions were also carried out with 4-monoamino- and 2,4-diaminopyrimidines functionalized with the *N*-propargyl group. The new chimeric compounds, coupled with gibberellins thus obtained, were characterized by 1D- and 2D-NMR techniques and HRMS measurements. While the 4-monoamino-substituted derivatives exhibited only weak antibacterial activity, they demonstrated significant antifungal effectiveness against *C. krusei*. In general, 5-chloro-substituted pyrimidine derivatives displayed more consistent biological activities compared to their 5-fluoro counterparts, with the exception of one derivative, which showed acceptable activity against both *C. krusei* and *C. albicans*. The two derivatives featuring 5-chloro and 2-((4-(trifluoromethyl)phenyl)amino substituents proved to be highly effective against *P. aeruginosa*, making them promising candidates for further research. Aiming to elucidate the molecular interactions between the active compounds and their potential targets, molecular docking studies were conducted using AutoDock Vina 1.1.2. involving the most active compounds against *P. aeruginosa.* **Conclusions:** The biological effects of 2-monoamino or 2,4-diamino substitution as well as the effect of chloro or fluoro substitution at position 5 of the pyrimidine ring combined with the *allo*-gibberic acid moiety were determined. Compounds with selective antibacterial activity against *P. aeruginosa* as well as selective antifungal activity against *C. krusei* and *C. albicans* fungi were identified.

## 1. Introduction

**Scheme 1:**
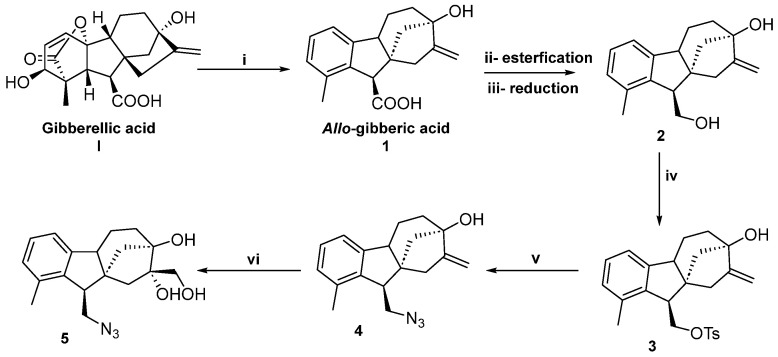
Synthesis of *allo*-gibberic acid azides. (i) HCl, 1.2 M, 65 °C, 3 h, 70%. (ii) CH_3_I, Cs_2_CO_3_, MeCN, reflux, 1 h, 92%. (iii) LiAlH_4_, dry THF, 40 °C, 3 h, 81%. (iv) TsCl, DIPEA, DMAP (cat.), DCM, 40 °C, 24 h, 75%. (v) NaN_3_, DMF, 80 °C, 5 h, 78%. (vi) OsO_4_, NMO, acetone, RT, 24 h, 98%.

Resistant microorganisms represent a significant global health challenge. According to a study published by the Global Research on Antimicrobial Resistance (GRAM) Project, more than 1.2 million people lost their lives in 2019 due to antibiotic-resistant bacterial infections [1]. Additionally, the Annual Epidemiological Report 2023 from the European Centre for Disease Prevention and Control highlights that, based on EARS-Net data from 2020, over 35,000 deaths occur annually in the EU/EEA as a direct result of antimicrobial-resistant infections. In 2023, the highest estimated incidence of invasive bacterial isolates reported by laboratories in the EU/EEA was attributed to *E. coli* (71.4 per 100,000 population), followed by *S. aureus* (36.9 per 100,000), *K. pneumoniae* (24.2 per 100,000), *E. faecalis* (14.1 per 100,000), *E. faecium* (10.7 per 100,000), *P. aeruginosa* (10.5 per 100,000), *S. pneumoniae* (7.2 per 100,000), and *Acinetobacter* spp. (4.6 per 100,000) [2]. Consequently, the search for new antimicrobials has become essential, as the number of non-susceptible organisms is gradually increasing, while the introduction of new antimicrobials to the market remains limited. In several cases, these were isolated from exotic marine organisms with complex structures [3,4]. Biologically validated products with a natural origin serve as an important resource for the development of new pharmaceuticals [5,6,7]. Their commercially available natural sources have historically provided a significant inspiration for the design and synthesis of bioactive compounds in medicinal chemistry. Gibberellins (GAs), a family of tetracyclic *ent*-kaurenoid diterpenes, are present widely in several commonly used plants, and some of them are used in agriculture as regulators to promote plant germination and growth. Gibberellic acid (GA3, **I**, Scheme 1) is nowadays produced commercially in large quantities by the fermentation of the fungus *Gibberella fujikuroi* and used in agriculture worldwide [8,9,10,11]. It reduces the time to flowering, and it also affects seed germination, leaf area expansion, stem elongation, and the maturation of plant sexual organs.

Besides agricultural applications, gibberellins play an important role in the synthesis of bioactive compounds, especially with antiproliferative activity [12,13,14]. In 2009, Koehler discovered that gibberellic acid and 9α-H *allo*-gibberic acid could modulate the NF-κB signaling pathway [15], a transcription pathway associated with various cancer diseases [16,17]. In recent years, we have reported the synthesis of *allo*-gibberic acid-based aminoalcohols, aminodiols, and aminotriols and studied their antiproliferative activity [18,19,20]. Similarly, the synthesis of 4-aryl-substituted triazole-coupled *allo*-gibberic acid derivatives with remarkable antiproliferative activities was reported by Wu et al. [21]. Since there are only a few articles addressing the antibacterial activity of *allo*-gibberic acid [22,23,24], this encouraged us to undertake investigation in this area.

2,4-Diaminopyrimidine derivatives possess biological and pharmaceutical importance as well as antimicrobial [25], antiprotozoal [26], and anticancer therapy uses [27,28,29]. Therefore, it seemed interesting to combine the *allo*-gibberic acid-based moiety with a mono- or diaminopyrimidine unit to obtain a promising chimeric structure with antimicrobial activity.

## 2. Results and Discussion

### 2.1. Synthesis of Allo-Gibberic Acid-Based Azides

*allo*-Gibberic acid **1** was obtained starting from commercially available gibberellic acid (**I**) through HCl-mediated hydrolysis, which involves lactone opening, decarboxylation, and the aromatization of ring A [14,19]. Afterwards, key intermediate azide **4** was prepared according to the literature, as shown in Scheme 1, in a four-step synthesis via esterification, followed by LiAlH_4_-mediated reduction, tosylation of the primary alcohol and, finally, tosyl–azide exchange [22]. Moreover, by the oxidation of the double bond using catalytic amounts of OsO_4_ and NMO, we were able to synthesize another key intermediate, triol-azide **5**, under mild conditions [30].

### 2.2. Synthesis of 2,4-Diaminopyrimidine-Derived Alkynes

**Scheme 2:**
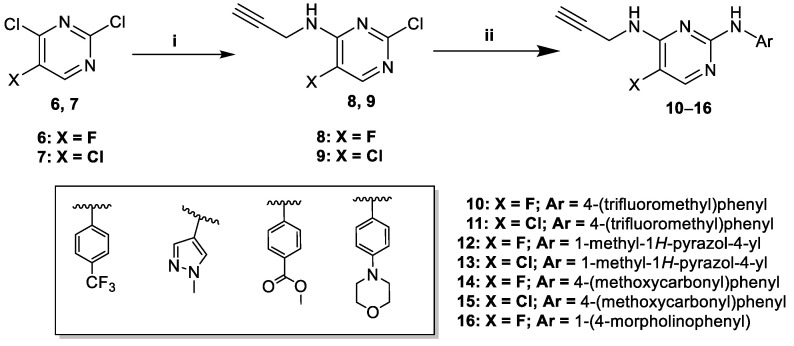
Synthesis of 2,4-diaminopyrimidine-derived alkynes. (i) Propargylamine (2 eq.), TEA, MeCN, 25 °C, 24 h, **8** (65%), **9** (88%). (ii) (a) Trifluoromethylaniline (1.1 eq.), EtOH, MW, 150 °C, 200 W, 19 bar, 2 h, **10** (58%), **11** (52%); (b) 1-methyl-1*H*-pyrazol-4-amine (1.1 eq.), EtOH, MW, 150 °C, 200 W, 19 bar, 2 h, **12** (91%), **13** (50%); (c) methyl 4-aminobenzoate (1 eq.), EtOH, MW, 100 °C, 200 W, 17 bar, 2 h, **14** (28%), **15** (81%); (d) 4-morpholinoaniline (1 eq.), EtOH, MW, 100 °C, 200 W, 17 bar, 2 h, **16** (52%).

Nitrogen-containing heterocycles hold a unique and significant position as a valuable source of therapeutic agents in medicinal chemistry [25,28,31,32]. Among them, 2,4-diaminopyrimidines are particularly important in cancer chemotherapy and the development of new antitrypanosomal, antimalarial, and antibacterial drugs [26,33,34,35,36]. Compounds **8**–**13** were synthesized according to our recently published article starting from halogen-substituted pyrimidines and propargylamine in two steps [20]. Furthermore, we were able to synthesize new 5-fluoro- and 5-chloro-substituted 2,4-diaminopyrimidines with 4-(methoxycarbonyl)phenyl and 1-(4-morpholinophenyl) moieties, as shown in Scheme 2. The aim of these reactions was to couple the products with the diterpene skeleton. Compounds **14** and **15** have low solubility, and they precipitated in ethanol as the reaction mixture started to cool down.

### 2.3. Coupling the Diterpene Skeleton with Monoamino- and Diaminopyrimidines via Click Reaction

**Scheme 3:**
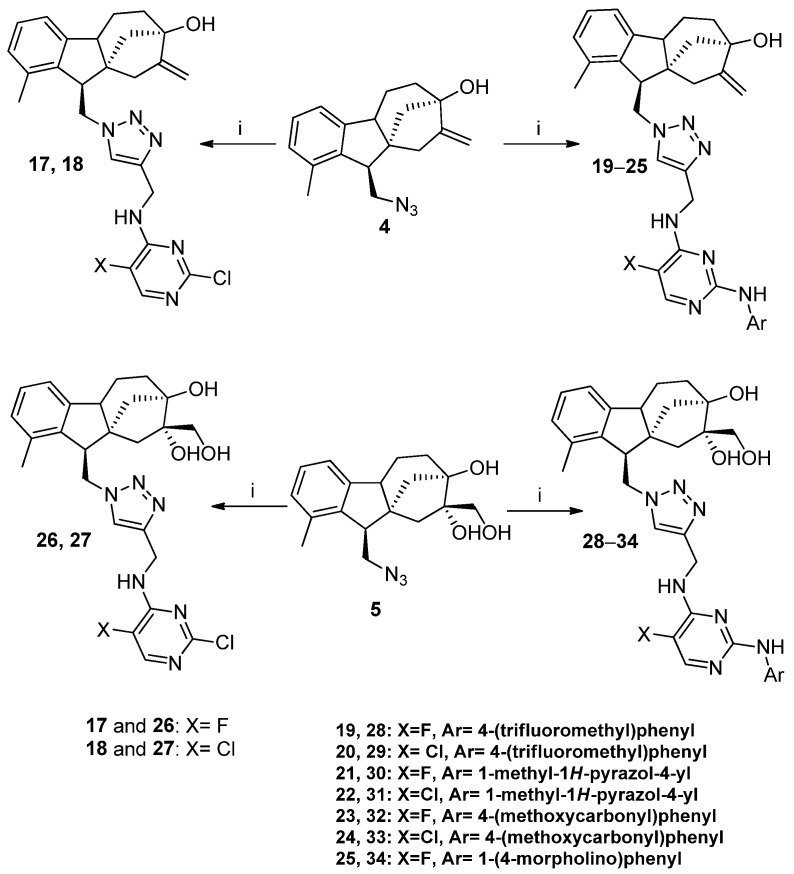
Synthesis of *allo*-gibberic acid-based monoamino- and diaminopyrimidines. (i) Cu(OAc)_2_.H_2_O (5 mol%), Na ascorbate (10 mol%), alkyne (1.1 eq.), 45 °C, 48 h; solvent mixture: *tert*-BuOH/H_2_O (2:1) for **17** (42%), **18** (33%), **19** (30%), **20** (59%), **21** (45%), **22** (47%), **24** (36%), **25** (91%), **26** (51%), **27** (34%), **28** (52%), **29** (36%), **30** (69%), **31** (95%), and **34** (92%), THF: H_2_O (2:1) for **23** (79%), **32** (44%), and **33** (43%), and *i*-PrOH:H_2_O (2:1) for **24** (36%).

The CuAAC reaction (copper-catalyzed azide–alkyne cycloaddition) is a widely used click chemistry approach for the synthesis of 1,2,3-triazoles with high efficiency and regioselectivity. Its simplicity, versatility, and compatibility with various functional groups make it invaluable in medicinal chemistry [37,38]. In order to prepare monoamino- and diaminopyrimidines derived from *allo*-gibberic acid, both azides **4** and **5** were subjected to a CuAAC reaction with the previously prepared alkynes, as shown in Scheme 3. Reactions were carried out under mild conditions at 45 °C with catalytic amounts of Cu(OAc)_2_.H_2_O and sodium ascorbate. The solubility of the corresponding alkyne played a crucial role in determining the type and amount of solvent required for the reaction. As for monoaminopyrimidines and 2,4-diaminopyrimidines with 4-(trifluoromethyl)phenyl, 1-methyl-1*H*-pyrazol-4-yl, and 1-(4-morpholino)phenyl substituents, a mixture of *tert*-BuOH and water (2:1) was used. In contrast, for compounds **23**, **24**, **32**, and **33**, involving the use of 4-(methoxycarbonyl)phenyl alkynes, a mixture of *i*-PrOH and water (2:1) or THF and water (2:1) was employed due to the insolubility of these alkynes in the *tert*-BuOH/water mixture. Moreover, the choice of solvent mixture influenced the solubility of the products during the reactions. In fact, compound **32** precipitated in the reaction mixture, whereas the alkyne precipitated when preparing compound **23**. Compounds **30** and **31** exhibited higher polarity than initially indicated by TLC analysis, necessitating the use of a more polar solvent system during column chromatography for effective separation.

### 2.4. Determination of the Relative Configuration of Azide **5**

**Figure 1:**
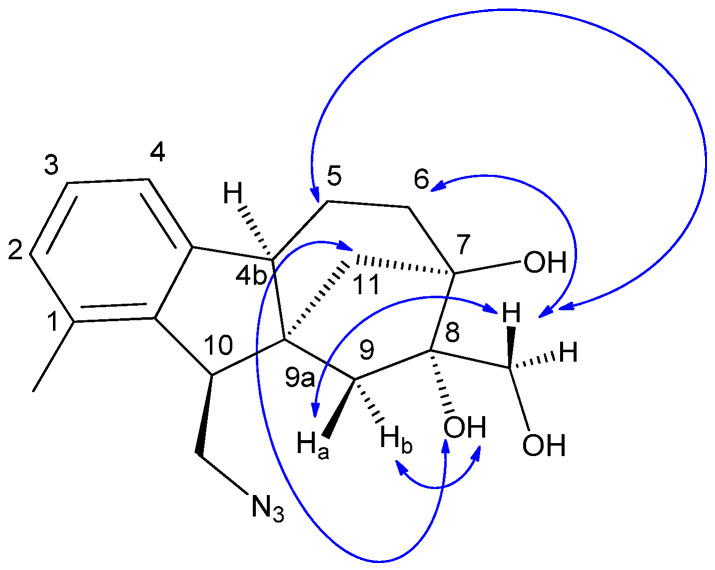
Relative configuration of azide **5** determined by NOESY experiment.

A new chiral center was generated by the dihydroxylation of the double bond, yielding azide **5**. The relative configuration of the obtained new stereocenter at position 8 was established using 2D-NMR experiments (Figure 1).

Despite the complexity caused by overlapping peaks in the aliphatic range in ^1^H NMR, clear NOE signals were observed between the CH_2_ proton of the primary alcoholic function [3.83 ppm (dd)] and the H_a_ proton at position 9 and similarly with CH_2_ at positions 5 and 6. Moreover, clear NOE signals were observed between OH at position 8 and H_b_ proton at position 9 as well as with CH_2_ at position 11. The HO group at position 8 was determined by HMBC measurements.

### 2.5. In Vitro Studies of Antimicrobial Effects of 4-Amino- and 2,4-Diaminopyrimidine Derivatives and Their Structure–Activity Relationship (SAR)

Since several diterpenes with an allylic alcohol function as well as several 2,4-diaminopyrimidines exerted antimicrobial activities against various bacterial and fungal strains, the antimicrobial activities of the prepared mono- and trihydroxy-2,4-diaminopyrimidine analogues were tested against two yeasts as well as two Gram-positive and two Gram-negative bacteria (Table 1) [39]. Interestingly, key intermediate azide **4** with an allylic alcohol function showed more potential antimicrobial activity against both Gram-positive and Gram-negative bacteria and similarly against yeasts. It is also evident from the results presented in Table 1 that 4-monoamino-substituted derivatives (**17**, **18**, **26**, and **27**) have only weak antibacterial activity. In contrast, these compounds possess remarkable antifungal activity against *Candida krusei*, especially compound **27**, which exhibits better activity compared to the reference compound *nystatine* at a concentration of 5 μg/mL. Generally, 5-chloro-substituted pyrimidine derivatives have more reliable biological activities compared with 5-fluoro derivatives, with the only exception of 2-(pyrazol-4-yl)amino derivative **21**, which possesses acceptable activity against both *C. krusei* and *C. albicans*. Derivative **29**, the best diaminopyrimidine with 5-chloro and 2-((4-(trifluoromethyl)phenyl)amino substituents, proved to be excellent against both *P. aeruginosa* and *C. krusei*, and it would be an excellent lead molecule for further investigation. Similarly, compound **20** [monohydroxy, with 5-chloro and 2-((4-(trifluoromethyl)phenyl substitution] shows a selective antibacterial effect against *P. aeruginosa*, while compound **28** was effective and selective against fungus.

### 2.6. Molecular Docking

Aiming to elucidate the molecular interactions between the active compounds and their potential targets, molecular docking studies were conducted using AutoDock Vina [40]. Two compounds, **20** and **29**, which demonstrated the highest activity against *P. aeruginosa* were selected for docking.

Compounds that strongly inhibit bacterial growth are likely to disrupt fundamental processes such as DNA replication. DNA gyrase is a well-recognized target for several antibiotics, including fluoroquinolones. While fluoroquinolones act by inhibiting the GyrA subunit of the enzyme, novobiocin uniquely targets the GyrB subunit. Targeting the GyrB subunit is particularly significant because it contains the ATP-binding site, which is essential for enzymatic activity. Additionally, the conserved nature of the ATP-binding site minimizes the likelihood of spontaneous resistance to ATP-competitive inhibitors [41]. Similarly, nitrogen-containing heterocycles have the ability to mimic the structure of ATP, enabling them to effectively compete for the ATP-binding site [42,43]. Furthermore, our previous research suggested that allo-gibberic acid derivatives containing nitrogen heterocycles exhibit affinity for the ATP-binding sites of certain kinase enzymes [20]. As such, selecting DNA gyrase B as a docking target aligns with the compound’s observed ability to completely inhibit *P. aeruginosa* growth. Moreover, the essential role of DNA gyrase B, its validated druggability, and the availability of high-resolution crystal structures make it an ideal target. These factors, combined with the compound’s potential ATP-binding site affinity, enable precise docking studies to be conducted.

**Figure 2:**
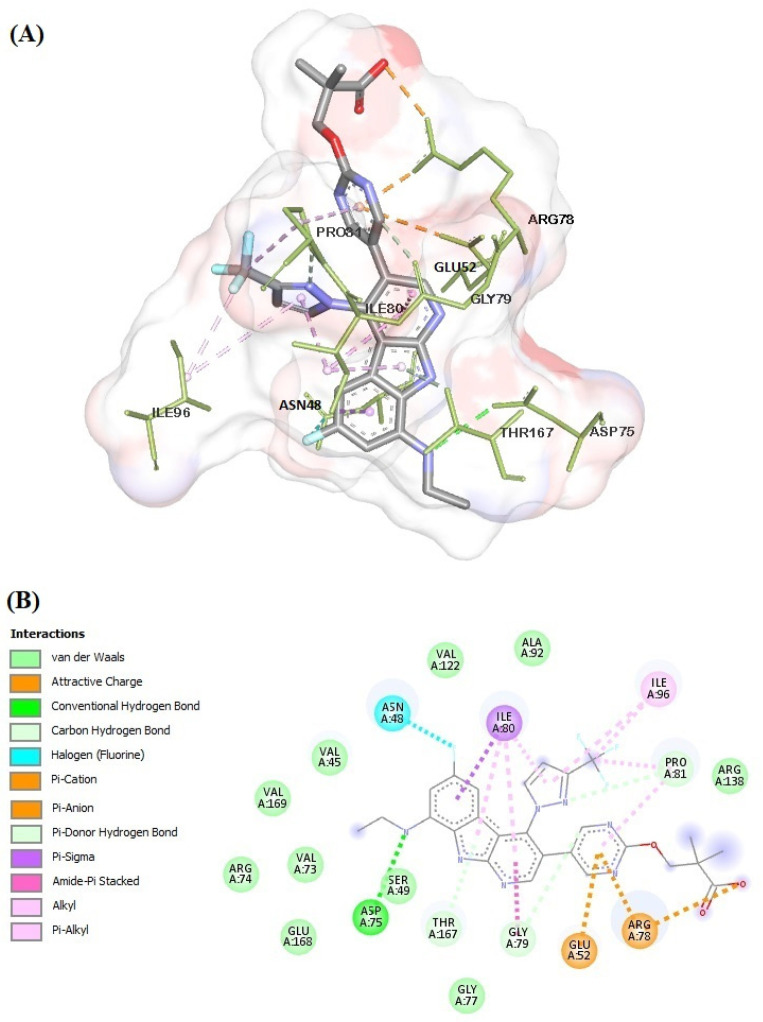
(**A**) Three-dimensional presentation of the complex between a known inhibitor and *P. aeruginosa* DNA gyrase (PDB code: 6M1S). (**B**) Two-dimensional presentation of the interactions between a known inhibitor and *P. aeruginosa* DNA gyrase (PDB code: 6M1S).

The DNA gyrase B structure of *P. aeruginosa* involves two amino acids, Arg78 and Arg138, which are crucial for its enzymatic activity. Additionally, it contains a hydrophobic groove formed by Ile80, Pro81, and Ile96. Moreover, a solvent-exposed area opposite two conserved amino acids, Asn48 and Asp51, may further contribute to improving the binding affinity [44]. In Figure 2A, a 3D illustration shows the interactions between a known co-crystalized pyrido [2,3-*b*]indole-type inhibitor and *P. aeruginosa* DNA gyrase B. The co-crystalized ligand has reliable interactions with key residues, as shown in Figure 2B, such as Arg78, Ile80, Pro81, and Ile96, as well as Asn48. The redocking of the co-crystalized ligand with the protein for the validation of the docking protocol yielded similar poses and interactions with binding energies below –7.0 kcal/mol and RMSD below 3 Å (detailed information can be found in the Supplementary Materials).

**Figure 3:**
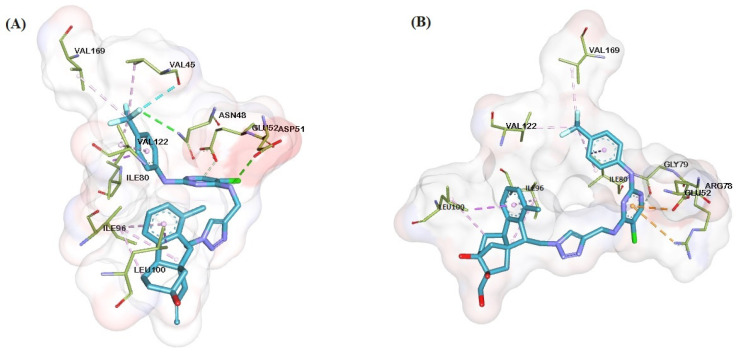
Three-dimensional presentation of (**A**) interactions between compound **20** and DNA gyrase of *P. aeruginosa* (PDB code: 6M1S) and (**B**) interactions between compound **29** and DNA gyrase of *P. aeruginosa* (PDB code: 6M1S).

The docking results indicate that compound **20** exhibits strong affinity with binding energies ranging from –8.9 to –8.2 kcal/mol. As suggested by the software and shown in Figure 3A, compound **20** may form two conventional hydrogen bonds: one between a trifluoromethyl fluorine atom and Asn48 and another between the amino group and Asp51. Moreover, van der Waals interactions with Arg78 can be observed. The diterpene portion of compound **20** can also interact with Ile96 within the hydrophobic pocket. Similarly, compound **29** demonstrated high affinity, with binding energies ranging from –9.0 to –8.1 kcal/mol. In addition to the interactions mentioned previously, compound **29** may be able to form cation–π and anion–π stacking interactions between the pyrimidine ring and Arg78 and Glu52, as visualized in Figure 3B, which might increase the affinity. All of these interactions may contribute to the binding affinity of compounds **20** and **29** with the protein, stabilizing the complex by enhancing the associations between the ligand and the receptor. On the other hand, it is worth mentioning that despite sharing the same structure with compounds **20** and **29**, with the exception of a fluorine atom replacing chlorine, compounds **19** and **28** exhibited almost no antibacterial activity against *P. aeruginosa* compared to **20** and **29**. This finding is consistent with previous results, which suggest that chlorine atom substitutions play a key role in modulating the antibacterial profile [45]. For better illustration, all 2D diagrams of the interactions of compounds with the proteins can be found in the Supplementary Materials.

**Figure 4:**
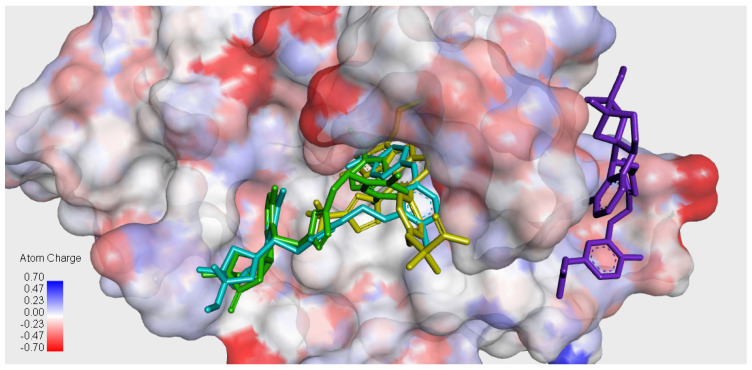
Three-dimensional presentation of the binding locations of the original ligand (yellow), compound **20** (green), compound **29** (cyan), and compound **30** (purple).

To gain deeper insights into the structure–activity relationship, the docking of a non-active compound was performed to compare its binding interactions with those of active compounds. Compound **30** was selected for this analysis. The results revealed binding affinities ranging from –7.4 to –6.8 kcal/mol, which are noticeably lower than the scores observed for active compounds. Although the compound was able to form four hydrogen bonds with Tyr57, Ser59, Asn76, and His139, an unfavorable interaction occurred between the 1-methyl-1H-pyrazol-4-yl group and Arg138. Moreover, the results indicated that all poses with low binding energies (below –7.0 kcal/mol) were located outside the original ligand’s binding site. In contrast, all poses of the active compounds were confined to the same binding site. Figure 4 illustrates a comparison of the binding locations of the original ligand, compound **20**, compound **29**, and compound **30**. Detailed 3D representations of all poses suggested by the program are provided in the Supplementary Materials.

## 3. Materials and Methods

### 3.1. General Methods

Commercially available reagents were used as obtained from suppliers (Novochem Co., Ltd., 1089 Budapest, Hungary, Orczy út 6.; Merck Ltd., Budapest, Hungary; and VWR International Ltd., Debrecen, Hungary), while solvents were dried according to standard procedures. Chromatographic separations and monitoring of reactions were carried out on Merck Kieselgel 60 (Merck Ltd., Budapest, Hungary). Optical rotations were measured in MeOH at 20 °C with a PerkinElmer 341 polarimeter (PerkinElmer Inc., Shelton, CT, USA). Melting points were determined with a Kofler apparatus (Nagema, Dresden, Germany). HRMS flow injection analysis was performed with a Thermo Scientific Q Exactive Plus hybrid quadrupole-Orbitrap (Thermo Fisher Scientific, Waltham, MA, USA) mass spectrometer coupled to a Waters Acquity I-Class UPLC™ (Waters, Manchester, UK). ^1^H-, ^13^C *J*-MOD-, and ^19^F-NMR spectra were recorded on a Bruker Avance DRX 500 spectrometer (Bruker Biospin, Karlsruhe, Baden-Württemberg, Germany) [500 MHz (^1^H), 125 MHz (^13^C *J*-MOD), and 470 MHz (^19^F) δ = 0 (TMS)]. Chemical shifts are expressed in ppm (δ) relative to TMS as an internal reference. *J* values are given in Hz. All ^1^H-, ^13^C, *J*-MOD-, ^19^F-NMR, COSY, NOESY, 2D-HMBC, and 2D-HMQC spectra are available in the Supporting Information file.

### 3.2. Starting Materials

Natural gibberellin (GA3, **I**) was obtained from AK Scientific Inc., Union City, CA, USA. The preparation of *allo*-gibberic acid **1**, intermediates **2**–**4**, and azide **5** was accomplished according to the literature methods starting from GA3 with spectroscopic data identical to those reported therein. Compounds **8** and **9** were prepared according to procedures reported in the literature [20,46].

### 3.3. Synthesis of New Compounds

#### 3.3.1. (7S,8S,9aR,10R)-10-(Azidomethyl)-8-(hydroxymethyl)-1-methyl-4b,5,6,8,9,10-hexahydro-7H-7,9a-methanobenzo[a]azulene-7,8-diol (**5**)

To a solution of **4** (0.91 g, 3.08 mmol) in acetone (15 mL), an aqueous solution of NMO (1.8 mL, 50% aqueous solution) and a solution of OsO_4_ in *tert*-BuOH (1.08 mL, 2% solution) were added in one portion. The reaction mixture was stirred at room temperature for 24 h and then quenched by the addition of a saturated aqueous solution of Na_2_SO_3_ (25 mL) and extracted with EtOAc (3 × 50 mL). The organic layer was dried over Na_2_SO_4_ and evaporated. The crude product was purified by chromatography on silica gel by using EtOAc. Yield: 0.99 g, 98%; white crystals; m.p.: 161–163 °C; [α]_D_^20^ = –7.933 (*c* 0.3 MeOH); ^1^H NMR (500 MHz, CDCl_3_) δ (ppm): 1.23 (d, *J* = 14.8 Hz, 1H), 1.59–1.50 (m, 3H), 1.91 (dd, *J* = 6.2, 13.5 Hz, 1H), 2.01 (dt, *J* = 6.0, 13.1 Hz, 1H), 2.07 (t, *J* = 5.8 Hz, 1H), 2.32 (ddd, *J* = 6.3, 6.3, 13.1 Hz, 1H), 2.37 (s, 3H), 2.57 (s, 1H), 2.76–2.69 (m, 2H), 3.25 (t, *J* = 6.6 Hz, 1H), 3.28 (s, 1H), 3.59 (dd, *J* = 6.3, 11.3 Hz, 1H), 3.72 (dd, *J* = 7.3, 12.8 Hz, 1H), 3.83 (dd, *J* = 5.0, 11.4 Hz, 1H), 4.10 (dd, *J* = 6.0, 12.8 Hz, 1H), 6.89 (d, *J* = 7.3 Hz, 1H), 6.95 (d, *J* = 7.6 Hz, 1H), 7.08 (t, *J* = 7.5 Hz, 1H). ^13^C-NMR (125 MHz, CDCl3) δ (ppm): 20.6, 22.2, 33.5, 35.3, 45.8, 48.2, 50.9, 51.4, 53.7, 64.8, 78.0, 80.6, 120.1, 127.0, 129.73, 134.0, 140.6, 144.7. HRMS (ESI): *m*/*z* calcd. for C_36_H_47_N_6_O_6_ [2M + H]^+^: 659.3557. Found: 659.3552.

#### 3.3.2. General Procedure for Preparing the *N*-Propargyl-Substituted Diaminopyrimidine Derivatives

In total, 1.0 mmol of **8** or **9** (obtained according to the literature method [20]) was dissolved in dry EtOH (8 mL), and the appropriate amine (1 eq.) was added to the solution. The reaction mixture was irradiated in a microwave reactor (200 W, 100 °C, 17 bar) for 2 h. After the completion of the reaction and cooling, the crude product was filtered off (**14** and **15**) or the mixture was evaporated, and the crude product was purified by column chromatography on silica gel (**16**).

##### Methyl 4-((5-Fluoro-4-(prop-2-yn-1-ylamino)pyrimidin-2-yl)amino)benzoate (**14**)

The reaction was accomplished starting from **8** (0.4 g, 1.0 mmol) and methyl 4-aminobenzoate (0.165 g, 1 mmol). The product precipitating in EtOH was obtained by filtration. Yield: 0.18 g, 28%; white crystals; m.p.: 212–214 °C; ^1^H NMR (500 MHz, DMSO-d_6_) δ (ppm): 3.14 (s, 1H), 3.80 (s, 3H), 4.17 (dd, *J* = 1.9, 5.6 Hz, 2H), 7.83 (d, *J* = 8.8 Hz, 2H), 7.92 (d, *J* = 8.8 Hz, 2H), 8.01–7.95 (m, 2H), 9.62 (s, 1H). ^13^C-NMR (125 MHz, DMSO-d_6_) δ (ppm): 29.8, 52.1, 73.2, 81.8, 117.6 (2C), 121.5, 130.5 (2C), 139.7 (d, *J* = 18.7 Hz), 141.8 (d, *J* = 246.5 Hz), 146.2, 152.4 (d, *J* = 12.5 Hz), 155.6 (d, *J* = 2.9 Hz), 166.6; ^19^F-NMR (470 MHz, DMSO-d_6_) δ (ppm): −167.8; HRMS (ESI): *m*/*z* calcd. for C_15_H_14_FN_4_O_2_ [M + H]^+^: 301.1101. Found: 301.1091.

##### Methyl 4-((5-Chloro-4-(prop-2-yn-1-ylamino)pyrimidin-2-yl)amino)benzoate (**15**)

The reaction was accomplished starting from **9** (0.202 g, 1.0 mmol) and methyl 4-aminobenzoate (0.165 g, 1 mmol). The product precipitating in EtOH was filtrated off as pure product. Yield: 0.257 g, 81%; white crystals; m.p.: 228–231 °C; ^1^H NMR (500 MHz, DMSO) δ (ppm): 3.12 (s, 1H), 3.81 (s, 3H), 4.17 (dd, *J* = 1.7, 5.6 Hz, 2H), 7.72 (t, *J* = 5.6 Hz, 1H), 7.85 (d, *J* = 8.5 Hz, 2H), 7.94 (d, *J* = 8.5 Hz, 2H), 8.07 (s, 1H), 9.75 (s, 1H). ^13^C-NMR (125 MHz, DMSO-d_6_) δ (ppm): 30.4, 52.1, 72.9, 81.9, 105.2, 118.1 (2C), 121.9, 130.4 (2C), 145.8, 153.9, 157.6, 157.9, 166.5; HRMS (ESI): *m*/*z* calcd. for C_15_H_14_ClN_4_O_2_ [M + H]^+^: 317.0805. Found: 317.0794.

##### 5-Fluoro-N^2^-(4-Morpholinophenyl)-N^4^-(prop-2-yn-1-yl)pyrimidine-2,4-diamine (**16**)

The reaction was accomplished starting from **8** (0.40 g, 1.0 mmol) and 4-morpholino aniline (0.178 g, 1.0 mmol). The product was purified by column chromatography on silica gel with DCM/MeOH (39:1). Yield: 0.37 g, 52%; white crystals; m.p.: 161–164 °C; ^1^H NMR (500 MHz, CDCl_3_) δ (ppm): 2.28 (s, 1H), 3.11 (dd, *J* = 4.6, 4.6 Hz, 4H), 3.86 (dd, *J* = 4.6, 4.6 Hz, 4H), 4.26 (dd, *J* = 2.2, 5.4 Hz, 2H), 5.09 (s, 1H), 6.80 (s, 1H), 6.89 (d, *J* = 8.7 Hz, 2H), 7.46 (d, *J* = 8.7 Hz, 2H), 7.81 (d, *J* = 2.7 Hz, 1H). ^13^C-NMR (125 MHz, CDCl_3_) δ (ppm): 30.2, 50.2 (2C), 67.0 (2C), 71.6, 79.8, 116.6 (2C), 120.7 (2C), 133.0, 139.6 (d, *J* = 19.4 Hz), 141,3 (d, *J* = 244.5 Hz), 146.9, 151.7 (d, *J* = 12.0 Hz), 156.1 (d, *J* = 2.8 Hz); ^19^F-NMR (470 MHz, DMSO-d_6_) δ (ppm): −169.9; HRMS (ESI): *m*/*z* calcd. for C_17_H_19_FN_5_O [M + H]^+^: 328.1574. Found: 328.1563.

#### 3.3.3. General Procedure for Preparation of 1,2,3-Triazols by Click Reaction

To a solution of azide **4** or **5** (0.304 mmol) in a mixture of *tert*-BuOH/H_2_O (2:1, 12 mL), THF/H_2_O (2:1, 12 mL), or *i*-PrOH/H_2_O (2:1, 12 mL), Cu(OAc)_2_·H_2_O (0.1 eq.), sodium ascorbate (0.1 eq.), and the appropriate acetylene derivative **17**–**34** (0.334 mmol, 1.1 eq.) were added. The mixture was stirred for 48 h at 45 °C. In the case of crystalline product separation, the crude product was obtained by filtration. In other cases, the organic solvent was evaporated, and the residue was dissolved in water (10 mL) and then extracted with DCM or CHCl_3_ (3 × 50 mL). The organic phase was dried over Na_2_SO_4_ and evaporated at low pressure, and the crude product was purified by column chromatography on silica gel.

##### (7*S*,9a*S*,10*R*)-10-((4-(((2-Chloro-5-fluoropyrimidin-4-yl)amino)methyl)-1*H*-1,2,3-triazol-1-yl)methyl)-1-methyl-8-methylene-4b,5,6,8,9,10-hexahydro-7*H*-7,9a-methanobenzo[a]azulen-7-ol (**17**)

The reaction was accomplished starting from **4** and alkyne **8** in *tert*-BuOH/H_2_O (2:1), and the product was purified by column chromatography on silica gel with DCM/MeOH (19:1). Yield: 0.061 g, 42%; white crystals; m.p.: 104–107 °C; [α]_D_^20^ = –87.1 (*c* 0.28 MeOH); ^1^H NMR (500 MHz, CDCl_3_) δ (ppm): 0.39 (dd, *J* = 1.9, 10.2 Hz, 1H), 1.40 (dd, *J* = 1.9, 10.2 Hz, 1H), 1.51 (dq, *J* = 5.4, 12.8 Hz, 1H), 1.65 (s, 1H), 1.82 (dt, *J* = 5.2, 12.2 Hz, 1H), 1.93 (dd, *J* = 1.6, 17.0 Hz, 1H), 2.19 (ddd, *J* = 5.9, 5.9, 12.5 Hz, 1H), 2.38 (s, 3H), 2.61 (d, *J* = 17.1 Hz, 1H), 2.68 (dd, *J* = 5.0, 12.4 Hz, 1H), 3.84 (dd, *J* = 4.9, 11.5 Hz, 1H), 4.82–4.70 (4H, m), 4.93 (s, 1H), 5.32 (dd, *J* = 5.1, 14.0 Hz, 1H), 6.49 (s, 1H), 6.95 (d, *J* = 7.4 Hz, 1H), 6.97 (d, *J* = 7.5 Hz, 1H), 7.12 (t, *J* = 7.5 Hz, 1H), 7.77 (s, 1H), 7.89 (d, *J* = 2.6 Hz, 1H); ^13^C-NMR (125 MHz, CDCl_3_) δ (ppm): 21.4, 21.9, 30.7, 35.9, 38.8, 47.8, 49.3, 49.6, 51.6, 53.2, 79.6, 104.1, 120.5, 123.1, 127.4, 129.9, 133.3, 139.0, 140.0 (d, *J* = 19.6 Hz), 144.2, 144.4, 145.1, 146.5, 153.3 (d, *J* = 12.7 Hz), 153.6; ^19^F-NMR (470 MHz, CDCl_3_) δ (ppm): −158.8; HRMS (ESI): *m*/*z* calcd. for C_25_H_27_ClFN_6_O [M + H]^+^: 481.1919. Found: 481.1905.

##### (7*S*,9a*S*,10*R*)-10-((4-(((2,5-Dichloropyrimidin-4-yl)amino)methyl)-1*H*-1,2,3-triazol-1-yl)methyl)-1-methyl-8-methylene-4b,5,6,8,9,10-hexahydro-7*H*-7,9a-methanobenzo[a]azulen-7-ol (**18**)

The reaction was accomplished starting from **4** and alkyne **9** in *tert*-BuOH/H_2_O (2:1). Purification with column chromatography on silica gel with DCM/MeOH gave compound **18**. Yield: 0.050 g, 33%; white crystals; m.p.: 111–114 °C; [α]_D_^20^ = –72.87 (*c* 0.26 MeOH); ^1^H NMR (500 MHz, CDCl_3_) δ (ppm): 0.42 (dd, *J* = 2.0, 10.1 Hz, 1H), 1.43 (dd, *J* = 1.9, 10.3 Hz, 1H), 1.51 (dq, *J* = 5.3, 12.8 Hz, 1H), 1.64 (d, *J* = 12.2 Hz, 1H), 1.83 (dt, *J* = 5.2, 12.3 Hz, 1H), 1.93 (d, *J* = 17.1 Hz, 1H), 2.19 (ddd, *J* = 5.8, 5.8, 12.5 Hz, 1H), 2.38 (s, 3H), 2.58 (d, *J* = 17.2 Hz, 1H), 2.68 (dd, *J* = 4.9, 12.5 Hz, 1H), 3.86 (dd, *J* = 5.0, 11.3 Hz, 1H), 4.84–4.69 (4H, m), 4.93 (s, 1H), 5.31 (dd, *J* = 5.1, 14.1 Hz, 1H), 6.49 (t, *J* = 5.7 Hz, 1H), 6.95 (d, *J* = 7.5 Hz, 1H), 6.97 (d, *J* = 7.4 Hz, 1H), 7.12 (t, *J* = 7.5 Hz, 1H), 7.76 (s, 1H), 8.05 (s, 1H). ^13^C-NMR (125 MHz, CDCl_3_) δ (ppm): 21.3, 21.9, 30.8, 36.4, 38.8, 47.9, 49.3, 49.5, 51.5, 53.3, 79.6, 104.1, 113.7, 120.5, 123.1, 127.3, 129.9, 133.3, 139.1, 144.1, 145.1, 153.7, 153.9, 158.1, 158.5; HRMS (ESI): *m*/*z* calcd. for C_25_H_27_C_l2_N_6_O [M + H]^+^: 497.1623. Found: 497.1616.

##### (7*S*,9a*S*,10*R*)-10-((4-(((5-Fluoro-2-((4-(trifluoromethyl)phenyl)amino)pyrimidin-4-yl)amino)methyl)-1*H*-1,2,3-triazol-1-yl)methyl)-1-methyl-8-methylene-4b,5,6,8,9,10-hexahydro-7*H*-7,9a-methanobenzo[a]azulen-7-ol (**19**)

The reaction was accomplished starting from **4** and alkyne **10** in *tert*-BuOH/H_2_O (2:1). Purification was performed by column chromatography on silica gel with DCM/MeOH (39:1). Yield: 0.055 g, 30%; pink crystals; m.p.: 123–125 °C; [α]_D_^20^ = –74.4 (*c* 0.33 MeOH); ^1^H NMR (500 MHz, CDCl_3_) δ (ppm): 0.53 (dd, *J* = 2.0, 10.3 Hz, 1H), 1.43 (dd, *J* = 1.9, 10.2 Hz, 1H), 1.55–1.46 (1H, m), 1.73–1.59 (1H, m), 1.86–1.78 (1H, m), 1.91 (d, *J* = 17.3 Hz, 1H), 2.22–2.14 (1H, m), 2.32 (s, 3H), 2.56 (d, *J* = 17.1 Hz, 1H), 2.64 (dd, *J* = 4.9, 12.3 Hz, 1H), 3.77 (dd, *J* = 5.1, 11.0 Hz, 1H), 4.67 (dd, *J* = 11.2, 14.0 Hz, 1H), 4.87–4.75 (3H, m), 4.94 (s, 1H), 5.25 (dd, *J* = 5.1, 14.1 Hz, 1H), 5.84 (s, 1H), 6.93 (d, *J* = 7.3 Hz, 1H), 6.95 (d, *J* = 7.4 Hz, 1H), 7.11 (t, *J* = 7.5 Hz, 1H), 7.21 (s, 1H), 7.54 (d, *J* = 8.6 Hz, 2H), 7.55 (s, 2H), 7.72 (d, *J* = 8.5 Hz, 2H), 7.84 (s, 1H). ^13^C-NMR (125 MHz, CDCl_3_) δ (ppm): 21.2, 21.9, 30.8, 36.2, 38.9, 47.9, 49.2, 49.4, 51.5, 53.3, 79.7, 104.0, 117.9 (2C), 120.4, 122.0, 123.5, 126.1 (q, *J* = 3.7 Hz, 2C), 127.3, 129.9, 133.2, 139.0, 139.1 (d, *J* = 20.3 Hz), 141.8 (d, *J* = 246.9 Hz), 143.2, 145.0, 145.1 (2C), 152.3 (d, *J* = 12 Hz), 153.7, 155.1 (d, *J* = 3.6 Hz); ^19^F-NMR (470 MHz, CDCl_3_) δ (ppm): −166.8, −61.6; HRMS (ESI): *m*/*z* calcd. for C_32_H_32_F_4_N_7_O [M + H]^+^: 606.2604. Found: 606.2589.

##### (7*S*,9a*S*,10*R*)-10-((4-(((5-Chloro-2-((4-(trifluoromethyl)phenyl)amino)pyrimidin-4-yl)amino)methyl)-1*H*-1,2,3-triazol-1-yl)methyl)-1-methyl-8-methylene-4b,5,6,8,9,10-hexahydro-7*H*-7,9a-methanobenzo[a]azulen-7-ol (**20**)

The reaction was accomplished starting from **4** and alkyne **11** in *tert*-BuOH/H_2_O (2:1). Purification was carried out with column chromatography on silica gel with DCM/MeOH (39:1). Yield: 0.112 g, 59%; white crystals; m.p.: 128–130 °C; [α]_D_^20^ = –110.67 (*c* 0.26 MeOH); ^1^H NMR (500 MHz, CDCl_3_) δ (ppm): 0.53 (dd, *J* = 2.1, 10.2 Hz, 1H), 1.44 (dd, *J* = 1.9, 10.1 Hz, 1H), 1.49 (dq, *J* = 5.4, 12.7 Hz, 1H), 1.69–1.65 (1H, m), 1.82 (dt, *J* = 5.2, 12.3 Hz, 1H), 1.91 (dd, *J* = 1.8, 17.3 Hz, 1H), 2.18 (ddd, *J* = 5.8, 5.8, 12.6 Hz, 1H), 2.32 (s, 3H), 2.54 (d, *J* = 17.2 Hz, 1H), 2.64 (dd, *J* = 4.9, 12.3 Hz, 1H), 3.77 (dd, *J* = 5.0, 11.1 Hz, 1H), 4.65 (dd, *J* = 11.0, 14.1 Hz, 1H), 4.76 (s, 1H), 4.82 (dq, *J* = 5.9, 18.6 Hz, 2H), 4.94 (s, 1H), 5.24 (dd, *J* = 5.2, 14.1 Hz, 1H), 6.01 (t, *J* = 5.7 Hz, 1H), 6.93 (d, *J* = 7.4 Hz, 1H), 6.94 (d, *J* = 7.3 Hz, 1H), 7.11 (t, *J* = 7.4 Hz, 1H), 7.22 (s, 1H), 7.53 (s, 1H), 7.56 (d, *J* = 8.5 Hz, 2H), 7.72 (d, *J* = 8.5 Hz, 2H), 7.98 (s, 1H). ^13^C-NMR (125 MHz, CDCl_3_) δ (ppm): 21.2, 21.9, 30.8, 36.7, 38.8, 48.0, 49.2, 49.4, 51.5, 53.3, 79.7, 104.0, 106.2, 118.4 (2C), 120.4, 122.1 (2C), 123.9, 126.1 (q, *J* = 3.8 Hz, 2C), 127.3, 129.9, 133.3, 139.1, 142.8, 145.0, 145.2, 153.3, 153.8, 157.5, 157.7; ^19^F-NMR (470 MHz, CDCl_3_) δ (ppm): −61.7; HRMS (ESI): *m*/*z* calcd. for C_32_H_32_ClF_3_N_7_O [M + H]^+^: 622.2309. Found: 622.2293.

##### (7*S*,9a*S*,10*R*)-10-((4-(((5-Fluoro-2-((1-methyl-1*H*-pyrazol-4-yl)amino)pyrimidin-4-yl)amino)methyl)-1*H*-1,2,3-triazol-1-yl)methyl)-1-methyl-8-methylene-4b,5,6,8,9,10-hexahydro-7*H*-7,9a-methanobenzo[a]azulen-7-ol (**21**)

The reaction was accomplished starting from **4** (0.12 g, 0.406 mmol), alkyne **12**, and *tert*-BuOH and water (2:1, 18 mL). Purification with silica gel with DCM/MeOH (19:1) gave compound **21**. Yield: 0.099 g, 45%; white crystals; m.p.: 138–141 °C; [α]_D_^20^ = –78 (*c* 0.28 MeOH); ^1^H NMR (500 MHz, CDCl_3_) δ (ppm): 0.53 (dd, *J* = 1.6, 10.3 Hz, 1H), 1.44 (dd, *J* = 2.0, 10.3 Hz, 1H), 1.51 (dq, *J* = 5.3, 12.7 Hz, 1H), 1.65 (dd, *J* = 5.1, 11.7 Hz, 1H), 1.82 (dt, *J* = 5.2, 12.4 Hz, 1H), 1.91 (d, *J* = 17.2 Hz, 1H), 2.18 (ddd, *J* = 5.7, 5.7, 12.4 Hz, 1H), 2.36 (s, 3H), 2.58 (d, *J* = 17.2 Hz, 1H), 2.65 (dd, *J* = 4.9, 12.3 Hz, 1H), 3.81 (dd, *J* = 4.8, 11.4 Hz, 1H), 3.86 (s, 3H), 4.67 (dd, *J* = 11.6, 13.9 Hz, 1H), 4.84–4.71 (3H, m), 4.94 (s, 1H), 5.28 (dd, *J* = 5.0, 14.1 Hz, 1H), 5.73 (t, *J* = 5.8 Hz, 1H), 6.83 (s, 1H), 6.93 (d, *J* = 7.4 Hz, 2H), 6.96 (d, *J* = 7.4 Hz, 2H), 7.11 (t, *J* = 7.5 Hz, 1H), 7.50 (s, 1H), 7.55 (s, 1H), 7.70 (s, 1H), 7.79 (d, *J* = 3.1 Hz, 1H). ^13^C-NMR (125 MHz, CDCl_3_) δ (ppm): 21.3, 21.9, 30.8, 36.3, 38.9, 39.2, 48.1, 49.2, 49.4, 51.5, 53.3, 79.6, 104.0, 120.4, 121.1, 122.0, 123.4, 127.3, 129.9, 130.9, 133.3, 139.0, 139.1 (d, *J* = 19.5 Hz), 141.2 (d, *J* = 243 Hz), 145.1, 145.7, 152.5 (d, *J* = 12.7 Hz), 153.8, 155.7 (d, *J* = 2.5 Hz); ^19^F-NMR (470 MHz, CDCl_3_) δ (ppm): −169.8; HRMS (ESI): *m*/*z* calcd. for C_29_H_33_FN_9_O [M + H]^+^: 542.2792. Found: 542.2776.

##### (7*S*,9a*S*,10*R*)-10-((4-(((5-Chloro-2-((1-methyl-1*H*-pyrazol-4-yl)amino)pyrimidin-4-yl)amino)methyl)-1*H*-1,2,3-triazol-1-yl)methyl)-1-methyl-8-methylene-4b,5,6,8,9,10-hexahydro-7*H*-7,9a-methanobenzo[a]azulen-7-ol (**22**)

The reaction was accomplished starting from **4** and alkyne **13** in *tert*-BuOH/H_2_O (2:1). Column chromatography on silica gel with DCM/MeOH (19:1) was used for purification. Yield: 0.080 g, 47%; white crystals; m.p.: 165–170 °C; [α]_D_^20^ = –71.6 (*c* 0.28 MeOH); ^1^H NMR (500 MHz, CDCl_3_) δ (ppm): 0.53 (d, *J* = 10.3 Hz, 1H), 1.45 (dd, *J* = 1.9, 10.2 Hz, 1H), 1.55–1.47 (1H, m), 1.65 (dd, *J* = 5.0, 12.0 Hz, 1H), 1.82 (dt, *J* = 5.3, 12.2 Hz, 1H), 1.91 (d, *J* = 17.2 Hz, 1H), 2.21–2.14 (1H, m), 2.36 (s, 3H), 2.57 (d, *J* = 17.2 Hz, 1H), 2.65 (dd, *J* = 4.9, 12.4 Hz, 1H), 3.81 (dd, *J* = 4.9, 11.3 Hz, 1H), 3.86 (s, 3H), 4.66 (dd, *J* = 11.7, 13.9 Hz, 1H), 4.85–4.72 (3H, m), 4.94 (s, 1H), 5.27 (dd, *J* = 5.0, 14.1 Hz, 1H), 5.92 (t, *J* = 5.8 Hz, 1H), 6.89 (s, 1H), 6.93 (d, *J* = 7.4 Hz, 1H), 6.96 (d, *J* = 7.3 Hz, 1H), 7.11 (t, *J* = 7.5 Hz, 1H), 7.51 (s, 1H), 7.52 (s, 1H), 7.68 (s, 1H), 7.91 (s, 1H); ^13^C-NMR (125 MHz, CDCl_3_) δ (ppm): 21.3, 21.9, 29.7, 30.8, 36.8, 39.2, 48.1, 49.2, 49.4, 51.5, 53.3, 79.6, 103.9, 120.4, 121.4, 122.0, 122.9, 127.3, 129.9, 131.1, 133.3, 139.1, 145.1 (2C), 145.8, 153.4, 153.8, 157.8, 157.9; HRMS (ESI): *m*/*z* calcd. for C_29_H_33_ClN_9_O [M + H]^+^: 558.2497. Found: 558.2486.

##### Methyl 4-((5-Fluoro-4-(((1-(((7*S*,9a*S*,10*R*)-7-hydroxy-1-methyl-8-methylene-5,6,7,8,9,10-hexahydro-4b*H*-7,9a-methanobenzo[a]azulen-10-yl)methyl)-1*H*-1,2,3-triazol-4-yl)methyl)amino)pyrimidin-2-yl)amino)benzoate (**23**)

The reaction was accomplished starting from **4** and alkyne **14** in *tert*-BuOH/H_2_O (2:1). Compound **28** was purified by column chromatography on silica gel with DCM/MeOH (19:1). Yield: 0.143 g, 79%; pale pink crystals; m.p.: 140–144 °C; [α]_D_^20^ = –89.3 (c 0.15 MeOH); ^1^H NMR (500 MHz, CDCl_3_) δ (ppm): 0.54 (d, *J* = 10.2 Hz, 1H), 1.43 (d, *J* = 10.2 Hz, 1H), 1.50 (dq, *J* = 5.3, 12.8 Hz, 1H), 1.65 (dd, *J* = 4.8, 11.9 Hz, 1H), 1.82 (dt, *J* = 5.2, 12.3 Hz, 1H), 1.90 (d, *J* = 17.0 Hz, 1H), 2.21–2.13 (m, 1H), 2.31 (s, 3H), 2.54 (d, *J* = 17.1 Hz, 1H), 2.63 (dd, *J* = 4.8, 12.4 Hz, 1H), 3.75–3.69 (m, 1H), 3.85 (s, 3H), 4.65 (dd, *J* = 11.2, 14.0 Hz, 1H), 4.87–4.73 (m, 3H), 4.95 (s, 1H), 5.22 (dd, *J* = 5.1, 14.1 Hz, 1H), 5.92 (t, *J* = 6.1 Hz, 1H), 6.92 (d, *J* = 7.5 Hz, 2H), 6.94 (d, *J* = 7.5 Hz, 2H), 7.10 (t, *J* = 7.5 Hz, 1H), 7.34 (s, 1H), 7.57 (s, 1H), 7.68 (d, *J* = 8.4 Hz, 2H), 7.84 (s, 1H), 7.98 (d, *J* = 8.4 Hz, 2H). ^13^C-NMR (125 MHz, CDCl_3_) δ (ppm): 21.2, 21.9, 30.8, 36.2, 38.9, 48.0, 49.2, 49.4, 51.5, 51.8, 53.3, 79.6, 104.0, 117.5 (2C), 120.4, 122.3, 123.1, 127.3, 129.9, 130.8 (2C), 133.3, 139.1, 139.2 (d, *J* = 19.5 Hz), 141.8 (d, *J* = 247.5 Hz), 144.5, 145.1 (2C), 152.4 (d, *J* = 12.0 Hz), 153.7, 155.0 (d, *J* = 2.8 Hz), 166.8; ^19^F-NMR (470 MHz, CDCl_3_) δ (ppm): −166.7; HRMS (ESI): *m*/*z* calcd. for C_33_H_35_FN_7_O_3_ [M + H]^+^: 596.2785. Found: 596.2770.

##### Methyl 4-((5-Chloro-4-(((1-(((7*S*,9a*S*,10*R*)-7-hydroxy-1-methyl-8-methylene-4b,6,7,8,9,10-hexahydro-5*H*-7,9a-methanobenzo[a]azulen-10-yl)methyl)-1*H*-1,2,3-triazol-4-yl)methyl)amino)pyrimidin-2-yl)amino)benzoate (**24**)

The reaction was accomplished starting from **4** and alkyne **15** in *i*-PrOH/H_2_O (2:1). Compound **24** was purified by column chromatography on silica gel with DCM/MeOH (19:1). Yield: 0.066 g, 36%; pale yellow crystals; m.p.: 135–138 °C; [α]_D_^20^ = –81.7 (*c* 0.17 MeOH); ^1^H NMR (500 MHz, CDCl3) δ (ppm): 0.54 (d, *J* = 10.3 Hz, 1H), 1.43 (d, *J* = 10.4 Hz, 1H), 1.50 (dq, *J* = 5.3, 12.8 Hz, 1H), 1.64 (dd, *J* = 5.2, 12.2 Hz, 1H), 1.82 (dt, *J* = 5.3, 12.3 Hz, 1H), 1.90 (d, *J* = 17.1 Hz, 1H), 2.21–2.14 (m, 1H), 2.31 (s, 3H), 2.52 (d, *J* = 17.2 Hz, 1H), 2.62 (dd, *J* = 5.0, 12.4 Hz, 1H), 3.72 (dd, *J* = 5.1, 10.9 Hz, 1H), 3.86 (s, 3H), 4.63 (dd, *J* = 11.1, 14.0 Hz, 1H), 4.90–4.74 (m, 3H), 4.94 (s, 1H), 5.21 (dd, *J* = 5.1, 14.1 Hz, 1H), 6.08 (t, *J* = 5.8 Hz, 1H), 6.92 (d, *J* = 7.4 Hz, 2H), 6.94 (d, *J* = 7.4 Hz, 2H), 7.10 (t, *J* = 7.5 Hz, 1H), 7.38 (s, 1H), 7.54 (s, 1H), 7.68 (d, *J* = 8.5 Hz, 2H), 7.96 (s, 1H), 7.99 (d, *J* = 8.5 Hz, 2H); ^13^C-NMR (125 MHz, CDCl_3_) δ (ppm): 21.2, 21.9, 30.8, 36.7, 38.9, 48.0, 49.2, 49.4, 51.5, 51.8, 53.3, 79.6, 104.0, 117.9 (2C), 120.4, 122.2, 123.4, 127.3, 129.9, 130.8 (2C), 133.3, 139.1 (2C), 144.0, 145.0 (2C), 153.1, 153.7, 157.3, 157.7, 166.7; HRMS (ESI): *m*/*z* calcd. for C_33_H_35_ClN_7_O_3_ [M + H]^+^: 612.2490. Found: 612.2478.

##### (7*S*,9a*S*,10*R*)-10-((4-(((5-Fluoro-2-((4-morpholinophenyl)amino)pyrimidin-4-yl)amino)methyl)-1*H*-1,2,3-triazol-1-yl)methyl)-1-methyl-8-methylene-4b,5,6,8,9,10-hexahydro-7*H*-7,9a-methanobenzo[a]azulen-7-ol (**25**)

The reaction was accomplished starting from **4** and alkyne **16** in *tert*-BuOH/H_2_O (2:1). Purification by column chromatography on silica gel with DCM/MeOH (19:1) delivered compound **25**. Yield: 0.172 g, 91%; pale yellow crystals; m.p.: 139–141 °C; [α]_D_^20^ = –87.41 (*c* 0.17 MeOH); ^1^H NMR (500 MHz, CDCl_3_) δ (ppm): 0.44 (d, *J* = 10.2 Hz, 1H), 1.36 (d, *J* = 10.2 Hz, 1H), 1.50 (dq, *J* = 5.3, 12.7 Hz, 1H), 1.68–1.59 (m, 1H), 1.81 (dt, *J* = 5.2, 12.2 Hz, 1H), 1.89 (d, *J* = 17.2 Hz, 1H), 2.21–2.14 (m, 1H), 2.33 (s, 3H), 2.57 (d, *J* = 17.2 Hz, 1H), 2.66 (dd, *J* = 4.7, 12.4 Hz, 1H), 3.08–2.97 (m, 4H), 3.64 (dd, *J* = 4.9, 11.1 Hz, 1H), 3.82–3.73 (m, 4H), 4.68–4.58 (m, 2H), 4.80–4.74 (m, 2H), 4.94 (s, 1H), 5.15 (dd, *J* = 4.9, 14.0 Hz, 1H), 5.79 (t, *J* = 6.0 Hz, 1H), 6.78 (s, 1H), 6.88 (d, *J* = 8.6 Hz, 2H), 6.94 (d, *J* = 7.5 Hz, 2H), 6.95 (d, *J* = 7.4 Hz, 2H), 7.11 (t, *J* = 7.4 Hz, 1H), 7.40 (s, 1H), 7.45 (d, *J* = 8.6 Hz, 2H), 7.77 (s, 1H); ^13^C-NMR (125 MHz, CDCl_3_) δ (ppm): 21.3, 21.9, 30.8, 36.0, 38.9, 47.9, 49.3 (2C), 50.0 (2C), 51.6, 53.2, 66.8 (2C), 79.6, 104.0, 116.4 (2C), 120.4, 121.8 (2C), 122.9, 127.3, 129.9, 133.0, 133.3, 139.2, 139.5 (d, *J* = 19.3 Hz), 141.4 (d, *J* = 244.5 Hz), 145.1, 145.3, 147.2, 152.2 (d, *J* = 12.3 Hz), 153.8, 156.3 (d, *J* = 2.7 Hz); ^19^F-NMR (470 MHz, CDCl_3_) δ (ppm): −169.3; HRMS (ESI): *m*/*z* calcd. for C_35_H_40_FN_8_O_2_ [M + H]^+^: 623.3258. Found: 623.3244.

##### (7*S*,8*S*,9a*R*,10*R*)-10-((4-(((2-Chloro-5-fluoropyrimidin-4-yl)amino)methyl)-1*H*-1,2,3-triazol-1-yl)methyl)-8-(hydroxymethyl)-1-methyl-4b,5,6,8,9,10-hexahydro-7*H*-7,9a-methanobenzo[a]azulene-7,8-diol (**26**)

The reaction was accomplished starting from **5** and alkyne **8** in *tert*-BuOH/H_2_O (2:1). Compound **26** was purified by column chromatography on silica gel with DCM/MeOH (9:1). Yield: 0.040 g, 51%; white crystals; m.p.: 137–140 °C; [α]_D_^20^ = –6.28 (*c* 0.29 MeOH); ^1^H NMR (500 MHz, DMSO-d_6_) δ (ppm): 1.20–1.12 (m, 2H), 1.53–1.42 (m, 3H), 1.70–1.62 (m, 2H), 2.15 (s, 3H), 2.20–2.17 (m, 1H), 2.63 (dd, *J* = 5.1, 11.8 Hz, 1H), 3.35 (d, *J* = 5.8 Hz, 2H), 3.69 (s, 1H), 3.86 (t, *J* = 6.9 Hz, 1H), 4.08 (s, 1H), 4.26 (t, *J* = 5.7 Hz, 1H), 4.69–4.54 (m, 3H), 5.16 (dd, *J* = 6.3, 14.3 Hz, 1H), 6.89 (d, *J* = 7.6 Hz, 2H), 6.91 (d, *J* = 7.5 Hz, 2H), 7.05 (t, *J* = 7.5 Hz, 1H), 8.12–8.10 (m, 2H), 8.65 (t, *J* = 5.7 Hz, 1H). ^13^C-NMR (125 MHz, DMSO-d_6_) δ (ppm): 20.6, 22.3, 33.4, 35.3, 36.1, 45.5, 48.9 (2C), 51.4, 53.6, 63.4, 78.4, 79.2, 120.6, 124.8, 127.1, 129.8, 133.6, 140.4, 140.9, 143.9, 144.7, 145.7, 153.7 (d, *J* = 13.00 Hz), 153.9 (d, *J* = 2.5 Hz). ^19^F-NMR (470 MHz, DMSO-d_6_): δ (ppm): −157.1; HRMS (ESI): *m*/*z* calcd. for C_25_H_29_ClFN_6_O_3_ [M + H]^+^: 515.1974. Found: 515.1960.

##### (7*S*,8*S*,9a*R*,10*R*)-10-((4-(((2,5-Dichloropyrimidin-4-yl)amino)methyl)-1*H*-1,2,3-triazol-1-yl)methyl)-8-(hydroxymethyl)-1-methyl-4b,5,6,8,9,10-hexahydro-7*H*-7,9a-methanobenzo[a]azulene-7,8-diol (**27**)

The reaction was accomplished starting from **5** and alkyne **9** in *tert*-BuOH/H_2_O (2:1). The obtained crude product was purified by column chromatography on silica gel with toluene/EtOH (9:1). Yield: 0.055 g, 34%; white crystals; m.p.: 240–243 °C; [α]_D_^20^ = –3.4 (*c* 0.26 MeOH); ^1^H NMR (500 MHz, DMSO-d_6_) δ (ppm): 1.20–1.13 (m, 2H), 1.55–1.45 (m, 3H), 1.71–1.63 (m, 2H), 2.14 (s, 3H), 2.20–2.17 (m, 1H), 2.63 (dd, *J* = 5.7, 11.7 Hz, 1H), 3.36 (d, *J* = 5.7 Hz, 2H), 3.66 (s, 1H), 3.86 (t, *J* = 6.7 Hz, 1H), 4.05 (s, 1H), 4.22 (t, *J* = 5.6 Hz, 1H), 4.57 (dd, *J* = 7.8, 14.4 Hz, 1H), 4.66 (ddd, *J* = 6.1, 15.5, 21.9 Hz, 2H), 5.15 (dd, *J* = 6.3, 14.3 Hz, 1H), 6.88 (d, *J* = 7.6 Hz, 1H), 6.90 (d, *J* = 7.6 Hz, 1H), 7.05 (t, *J* = 7.4 Hz, 1H), 8.08 (s, 1H), 8.19 (s, 1H), 8.38 (t, *J* = 5.9 Hz, 1H). ^13^C-NMR (125 MHz, DMSO-d_6_) δ (ppm): 20.6, 22.3, 33.4, 35.3, 36.7, 45.5, 48.9 (2C), 51.4, 53.6, 63.4, 78.4, 79.2, 113.5, 120.5, 124.7, 127.1, 129.8, 133.6, 140.9, 144.0, 145.7, 154.4, 157.8, 159.0. HRMS (ESI): *m*/*z* calcd. for C_25_H_29_Cl_2_N_6_O_3_ [M + H]^+^: 531.1678. Found: 531.1669.

##### (7*S*,8*S*,9a*R*,10*R*)-10-((4-(((5-Fluoro-2-((4-(trifluoromethyl)phenyl)amino)pyrimidin-4-yl)amino)methyl)-1*H*-1,2,3-triazol-1-yl)methyl)-8-(hydroxymethyl)-1-methyl-4b,5,6,8,9,10-hexahydro-7*H*-7,9a-methanobenzo[a]azulene-7,8-diol (**28**)

The reaction was accomplished starting from **5** and alkyne **10** in *tert*-BuOH/H_2_O (2:1) mixture. The obtained crude product was purified by column chromatography on silica gel with toluene/EtOH (9:1). Yield: 0.10 g, 52%; white crystals; m.p.: 146–148 °C; [α]_D_^20^ = +3.2 (*c* 0.27 MeOH); ^1^H NMR (500 MHz, DMSO-d_6_) δ (ppm): 1.19–1.12 (m, 2H), 1.53–1.43 (m, 2H), 1.55 (d, *J* = 10.2 Hz, 1H), 1.71–1.61 (m, 2H), 2.08 (s, 3H), 2.15 (q, *J* = 5.6 Hz, 1H), 2.60 (dd, *J* = 5.5, 11.9 Hz, 1H), 3.36 (d, *J* = 5.7 Hz, 2H), 3.72 (s, 1H), 3.84 (t, *J* = 6.8 Hz, 1H), 4.09 (s, 1H), 4.25 (t, *J* = 5.7 Hz, 1H), 4.55 (dd, *J* = 7.6, 14.3 Hz, 1H), 4.70 (ddt, *J* = 5.9, 14.2, 13.6 Hz, 2H), 5.11 (dd, *J* = 6.4, 14.4 Hz, 1H), 6.85 (d, *J* = 7.6 Hz, 1H), 6.89 (d, *J* = 7.3 Hz, 1H), 7.03 (t, *J* = 7.4 Hz, 1H), 7.52 (d, *J* = 8.7 Hz, 2H), 7.88 (d, *J* = 8.6 Hz, 2H), 7.97 (d, *J* = 3.5 Hz, 1H), 8.04 (t, *J* = 5.7 Hz, 1H), 8.15 (s, 1H), 9.50 (s, 1H). ^13^C-NMR (125 MHz, DMSO-d_6_) δ (ppm): 20.5, 22.3, 33.5, 35.3, 36.2, 45.4, 48.8, 48.9, 51.4, 53.6, 63.4, 78.4, 79.2, 117.9 (2C), 120.5, 120.5, 120.7, 124.5, 126.0 (d, *J* = 3.6 Hz, 2C), 127.0, 129.7, 133.5, 139.2 (d, *J* = 18.8 Hz), 141.0, 141.8 (d, *J* = 246.1 Hz), 145.1, 145.3, 145.6, 152.4 (d, *J* = 12.5 Hz), 155.7 (d, 2.8 Hz). ^19^F-NMR (470 MHz, DMSO-d_6_) δ (ppm): −165.9, −59.7 HRMS (ESI): *m*/*z* calcd. for C_32_H_34_F_4_N_7_O_3_ [M + H]^+^: 640.2659. Found: 640.2642.

##### (7*S*,8*S*,9a*R*,10*R*)-10-((4-(((5-Chloro-2-((4-(trifluoromethyl)phenyl)amino)pyrimidin-4-yl)amino)methyl)-1*H*-1,2,3-triazol-1-yl)methyl)-8-(hydroxymethyl)-1-methyl-4b,5,6,8,9,10-hexahydro-7*H*-7,9a-methanobenzo[a]azulene-7,8-diol (**29**)

The reaction was accomplished starting from **5** and alkyne **11** in *tert*-BuOH/H_2_O (2:1). The crude product was purified by column chromatography on silica gel with toluene/EtOH (9:1). Yield: 0.072 g, 36%; white crystals; m.p.: 185–189 °C; [α]_D_^20^ = +11.0 (*c* 0.27 MeOH); ^1^H NMR (500 MHz, DMSO) δ (ppm): 1.14 (d, *J* = 10.7 Hz, 1H), 1.17 (d, *J* = 13.5 Hz, 1H), 1.52–1.43 (m, 2H), 1.57 (d, *J* = 10.7 Hz, 1H), 1.73–1.64 (m, 2H), 2.06 (s, 3H), 2.19–2.11 (m, 1H), 2.60 (dd, *J* = 5.5, 11.8 Hz, 1H), 3.69 (s, 1H), 3.82 (t, *J* = 6.8 Hz, 1H), 4.09 (s, 1H), 4.25 (t, *J* = 5.6 Hz, 1H), 4.54 (dd, *J* = 7.5, 14.4 Hz, 1H), 4.72 (ddt, *J* = 5.9, 13.7, 12.9 Hz, 2H), 5.10 (dd, *J* = 6.3, 14.4 Hz, 1H), 6.84 (d, *J* = 7.5 Hz, 1H), 6.89 (d, *J* = 7.4 Hz, 1H), 7.03 (t, *J* = 7.4 Hz, 1H), 7.53 (d, *J* = 8.5 Hz, 2H), 7.76 (t, *J* = 5.8 Hz, 1H), 7.87 (d, *J* = 8.5 Hz, 2H), 8.03 (s, 1H), 8.11 (s, 1H), 9.59 (s, 1H); ^13^C-NMR (125 MHz, DMSO-d_6_) δ (ppm): 20.4, 22.3, 33.4, 35.3, 36.8, 45.4, 48.9 (2C), 51.4, 53.6, 63.4, 78.4, 79.2, 105.3, 118.5 (2C), 120.5, 120.9, 121.2, 124.4, 126.0 (d, *J* = 3.4 Hz, 2C), 127.0, 129.7, 133.5, 140.9, 144.8, 145.2, 145.6, 153.5, 157.8, 158.0; ^19^F-NMR (470 MHz, DMSO-d_6_): δ (ppm): −59.79; HRMS (ESI): *m*/*z* calcd. for C_32_H_34_ClF_3_N_7_O_3_ [M + H]^+^: 656.2363. Found: 656.2347.

##### (7*S*,8*S*,9a*R*,10*R*)-10-((4-(((5-Fluoro-2-((1-methyl-1H-pyrazol-4-yl)amino)pyrimidin-4-yl)amino)methyl)-1*H*-1,2,3-triazol-1-yl)methyl)-8-(hydroxymethyl)-1-methyl-4b,5,6,8,9,10-hexahydro-7*H*-7,9a-methanobenzo[a]azulene-7,8-diol (**30**)

The reaction was accomplished starting from **5** and alkyne **12** in *tert*-BuOH/H_2_O (2:1). Purification was conducted with column chromatography on silica gel with DCM/MeOH (15:1) increased to 100% MeOH. Yield: 0.121 g, 69%; white crystals; m.p.: 200–205 °C; [α]_D_^20^ = +5.42 (*c* 0.29 MeOH); ^1^H NMR (500 MHz, CDCl_3_) δ (ppm): 0.93 (d, *J* = 10.5 Hz, 1H), 1.19 (d, *J* = 10.7 Hz, 1H), 1.37 (d, *J* = 14.8 Hz, 1H), 1.58–1.48 (1H, m), 1.90–1.78 (2H, m), 2.30–2.22 (1H, m), 2.38 (s, 4H), 2.64 (dd, *J* = 5.5, 11.9 Hz, 1H), 3.55 (d, *J* = 11.4 Hz, 1H), 3.74 (d, *J* = 11.3 Hz, 1H), 3.81 (dd, *J* = 4.9, 11.6 Hz, 1H), 3.85 (s, 3H), 4.61 (dd, *J* = 11.8, 13.9 Hz, 1H), 4.80 (dt, *J* = 6.0, 16.1 Hz, 2H), 5.39 (dd, *J* = 4.8, 14.1 Hz, 1H), 5.77 (t, *J* = 6.1 Hz, 1H), 6.90 (d, *J* = 7.6 Hz, 1H), 6.94 (d, *J* = 7.6 Hz, 1H), 6.98 (s, 1H), 7.09 (t, *J* = 7.6 Hz, 1H), 7.53 (s, 1H), 7.69 (s, 1H), 7.78–7.75 (2H, m). ^13^C-NMR (125 MHz, CDCl_3_) δ (ppm): 21.4, 22.0, 29.6, 32.7, 34.7, 36.3, 39.1, 45.4, 49.5 (2C), 51.7, 53.1, 64.3, 78.6, 79.9, 120.5, 121.1, 123.5, 123.8, 127.3, 130.0, 133.4, 139.1, 139.2 (d, *J* = 19.4 Hz), 141.2 (d, *J* = 243.2 Hz) 144.7, 145.4, 152.5 (d, *J* = 12.0 Hz), 155.8. ^19^F-NMR (470 MHz, CDCl_3_) δ (ppm): −170.0; HRMS (ESI): *m*/*z* calcd. for C_29_H_35_FN_9_O_3_ [M + H]^+^: 576.2847. Found: 576.2832.

##### (7*S*,8*S*,9a*R*,10*R*)-10-((4-(((5-Chloro-2-((1-methyl-1′*H*-pyrazol-4-yl)amino)pyrimidin-4-yl)amino)methyl)-1*H*-1,2,3-triazol-1-yl)methyl)-8-(hydroxymethyl)-1-methyl-4b,5,6,8,9,10-hexahydro-7*H*-7,9a-methanobenzo[a]azulene-7,8-diol (**31**)

The reaction was accomplished starting from **5** and alkyne **13** in *tert*-BuOH/H_2_O (2:1). Column chromatography on silica gel with DCM/MeOH (6:1) gave compound **31**. Yield: 0.17 g, 95%; white crystals; m.p.: 166–170 °C; [α]_D_^20^ = +9.9 (*c* 0.19 MeOH); ^1^H NMR (500 MHz, CDCl_3_) δ (ppm): 0.95 (d, *J* = 11.1 Hz, 1H), 1.18 (d, *J* = 11.1 Hz, 1H), 1.39–1.32 (1H, m), 1.58–1.47 (1H, m), 1.90–1.77 (3H, m), 2.30–2.22 (1H, m), 2.37 (s, 3H), 2.64 (dd, *J* = 5.5, 11.9 Hz, 1H), 3.55 (d, *J* = 11.5 Hz, 1H), 3.73 (d, *J* = 11.5 Hz, 1H), 3.84–3.79 (1H, m), 3.85 (s, 3H), 4.61 (t, *J* = 13.0 Hz, 1H), 4.81 (d, *J* = 5.7 Hz, 2H), 5.39 (dd, *J* = 4.8, 14.0 Hz, 1H), 6.05 (s, 1H), 6.89 (d, *J* = 7.6 Hz, 1H), 6.94 (d, *J* = 7.6 Hz, 1H), 7.09 (t, *J* = 7.5 Hz, 1H), 7.38–7.29 (1H, m), 7.55 (s, 1H), 7.68 (s, 1H), 7.76 (s, 1H), 7.87 (s, 1H). ^13^C-NMR (125 MHz, CDCl_3_) δ (ppm): 21.4, 22.0, 32.8, 34.7, 36.8, 39.2, 45.4, 49.5 (2C), 51.7, 53.1, 64.3, 78.7, 79.9, 120.5 (2C), 121.4, 122.9, 123.9, 127.3, 130.0, 131.1 (2C), 133.4, 139.2, 144.7 (2C), 157.9 (2C); HRMS (ESI): *m*/*z* calcd. for C_29_H_35_ClN_9_O_3_ [M + H]^+^: 592.2551. Found: 592.2540.

##### Methyl 4-((4-(((1-(((7*S*,8*S*,9a*R*,10*R*)-7,8-Dihydroxy-8-(hydroxymethyl)-1-methyl-4b,6,7,8,9,10-hexahydro-5*H*-7,9a-methanobenzo[a]azulen-10-yl)methyl)-1*H*-1,2,3-triazol-4-yl)methyl)amino)-5-fluoropyrimidin-2-yl)amino)benzoate (**32**)

The reaction was accomplished starting from **5** and alkyne **14** in THF/H_2_O (2:1). Purification was conducted with column chromatography on silica gel with DCM/MeOH (19:1). Yield: 0.084 g, 44%; white crystals; m.p.: 251–253 °C; [α]_D_^20^ = +11.35 (*c* 0.13 DMSO); ^1^H NMR (500 MHz, DMSO) δ (ppm): 1.19–1.12 (m, 2H), 1.53–1.41 (m, 2H), 1.58 (d, *J* = 10.3 Hz, 1H), 1.73–1.63 (m, 2H), 2.07 (s, 3H), 2.19–2.11 (m, 1H), 2.60 (dd, *J* = 5.4, 11.9 Hz, 1H), 3.36 (d, *J* = 5.7 Hz, 2H), 3.71 (s, 1H), 3.83–3.78 (m, 4H), 4.07 (s, 1H), 4.22 (t, *J* = 5.7 Hz, 1H), 4.53 (dd, *J* = 7.4, 14.4 Hz, 1H), 4.70 (dq, *J* = 5.9, 14.0 Hz, 2H), 5.10 (dd, *J* = 6.3, 14.4 Hz, 1H), 6.84 (d, *J* = 7.5 Hz, 1H), 6.89 (d, *J* = 7.4 Hz, 1H), 7.03 (t, *J* = 7.4 Hz, 1H), 7.80 (s, 4H), 7.96 (d, *J* = 3.3 Hz, 1H), 8.03 (t, *J* = 5.8 Hz, 1H), 8.12 (s, 1H), 9.50 (s, 1H). ^13^C-NMR (125 MHz, DMSO-d_6_) δ (ppm): 20.5, 22.3, 33.5, 35.3, 36.2, 45.4, 48.9 (2C), 51.4, 52.0, 53.6, 63.4, 78.4, 79.3, 117.5 (2C), 120.5, 121.3, 124.5, 127.0, 129.7, 130.4 (2C), 133.6, 139.3 (d, *J* = 18.9 Hz), 141.0, 141.8 (d, *J* = 246.5 Hz), 145.1, 145.6, 146.2, 152.4 (d, *J* = 12.6 Hz), 155.6 (d, *J* = 2.8 Hz), 166.5; ^19^F-NMR (470 MHz, DMSO-d_6_) δ (ppm): −165.6; HRMS (ESI): *m*/*z* calcd. for C_33_H_37_FN7O_5_ [M + H]^+^: 630.2840. Found: 630.2825.

##### Methyl 4-((5-Chloro-4-(((1-(((7*S*,8*S*,9a*R*,10*R*)-7,8-dihydroxy-8-(hydroxymethyl)-1-methyl-4b,6,7,8,9,10-hexahydro-5*H*-7,9a-methanobenzo[a]azulen-10-yl)methyl)-1*H*-1,2,3-triazol-4-yl)methyl)amino)pyrimidin-2-yl)amino)benzoate (**33**)

The reaction was accomplished starting from **5** and alkyne **15** in *tert*-BuOH/H_2_O (2:1). Product **33** precipitated in EtOH was purified by filtration. Yield: 0.084 g, 43%; white crystals; m.p.: 283–285 °C; [α]_D_^20^ = +54.1 (*c* 0.13 DMSO); ^1^H NMR (500 MHz, DMSO-d_6_) δ (ppm): 1.20–1.12 (m, 2H), 1.53–1.41 (m, 2H), 1.60 (d, *J* = 10.4 Hz, 1H), 1.72–1.65 (m, 2H), 2.05 (s, 3H), 2.19–2.11 (m, 1H), 2.59 (dd, *J* = 5.4, 11.6 Hz, 1H), 3.36 (d, *J* = 5.6 Hz, 2H), 3.70 (s, 1H), 3.79 (s, 4H), 4.07 (s, 1H), 4.22 (t, *J* = 5.5 Hz, 1H), 4.52 (dd, *J* = 7.2, 14.2 Hz, 1H), 4.72 (dq, *J* = 5.8, 14.1 Hz, 2H), 5.08 (dd, *J* = 6.4, 14.4 Hz, 1H), 6.83 (d, *J* = 7.5 Hz, 1H), 6.88 (d, *J* = 7.4 Hz, 1H), 7.02 (t, *J* = 7.4 Hz, 1H), 7.83–7.74 (m, 5H), 8.04 (s, 1H), 8.09 (s, 1H), 9.63 (s, 1H); ^13^C-NMR (125 MHz, DMSO-d_6_) δ (ppm): 20.4, 22.4, 33.5, 35.3, 36.8, 45.4, 48.8 (2C), 51.4, 52.1, 53.6, 63.5, 78.4, 79.3, 105.3, 118.1 (2C), 120.5, 121.8, 124.4, 127.0, 129.7, 130.4 (2C), 133.6, 141.0, 145.2, 145.6, 145.8, 153.5, 157.8, 157.9, 166.5; HRMS (ESI): *m*/*z* calcd. for C_33_H_37_ClN_7_O_5_ [M + H]^+^: 646.2545. Found: 642.2534.

##### (7*S*,8*S*,9a*R*,10*R*)-10-((4-(((5-Fluoro-2-((4-morpholinophenyl)amino)pyrimidin-4-yl)amino)methyl)-1*H*-1,2,3-triazol-1-yl)methyl)-8-(hydroxymethyl)-1-methyl-4b,5,6,8,9,10-hexahydro-7*H*-7,9a-methanobenzo[a]azulene-7,8-diol (**34**)

The reaction was accomplished starting from **5** and alkyne **16** in *tert*-BuOH/H_2_O (2:1). The product was isolated after purification by column chromatography on silica gel with DCM/MeOH (19:1 increased to 9:1). Yield: 0.18 g, 92%; pale yellow crystals; m.p.: 121–124 °C; [α]_D_^20^ = –3.87 (*c* 0.18 MeOH); ^1^H NMR (500 MHz, DMSO-d_6_) δ (ppm): 1.20–1.13 (m, 2H), 1.52–1.42 (m, 2H), 1.57 (d, *J* = 10.3 Hz, 1H), 1.72–1.62 (m, 2H), 2.10 (s, 3H), 2.20–2.12 (m, 1H), 2.62 (dd, *J* = 5.2, 11.8 Hz, 1H), 2.98–2.94 (m, 4H), 3.36 (d, *J* = 5.8 Hz, 2H), 3.71–3.67 (m, 5H), 3.80 (t, *J* = 6.8 Hz, 1H), 4.08 (s, 1H), 4.22 (t, *J* = 5.8 Hz, 1H), 4.53 (dd, *J* = 7.5, 14.3 Hz, 1H), 4.65 (dq, *J* = 5.8, 14.0 Hz, 2H), 5.09 (dd, *J* = 6.3, 14.3 Hz, 1H), 6.80 (d, *J* = 8.7 Hz, 2H), 6.87 (d, *J* = 7.3 Hz, 1H), 6.90 (d, *J* = 7.3 Hz, 1H), 7.04 (t, *J* = 7.4 Hz, 1H), 7.51 (d, *J* = 8.7 Hz, 2H), 7.77 (t, *J* = 5.8 Hz, 1H), 7.84 (d, *J* = 3.5 Hz, 1H), 8.06 (s, 1H), 8.72 (s, 1H). ^13^C-NMR (125 MHz, DMSO-d_6_) δ (ppm): 20.5, 22.4, 33.5, 35.3, 36.0, 45.4, 48.8 (2C), 49.9 (2C), 53.6, 63.5, 66.6 (2C), 78.5, 79.3, 116.1 (2C), 120.2, 120.5 (2C), 124.6, 127.1, 129.8, 133.6, 134.3, 139.4 (d, *J* = 18.3 Hz), 141.0, 141,2 (d, *J* = 243.6 Hz), 145.2, 145.7 (2C), 146.1, 152.2 (d, *J* = 12.2 Hz), 156.5 (d, *J* = 2.8). ^19^F-NMR (470 MHz, DMSO-d_6_) δ (ppm): −168.5; HRMS (ESI): *m*/*z* calcd. for C_35_H_42_FN_8_O_4_ [M + H]^+^: 657.3313. Found: 657.3294.

### 3.4. Antimicrobial Analyses

The synthesized compounds were dissolved in DMSO and diluted with H_2_O to reach concentration levels of up to 100 μg/mL and 10 μg/mL with a final DMSO content of 1%. Then, the resulting solutions were investigated in a microdilution assay using bacterial and yeast strains including *Bacillus subtilis* SZMC 0209 (Gram-positive), *Staphylococcus aureus* SZMC 14,611 (Gram-positive), *Escherichia coli* SZMC 6271 (Gram-negative), and *Pseudomonas aeruginosa* SZMC 23,290 (Gram-negative) as well as *Candida albicans* SZMC 1533 and *C. krusei* SZMC 1352, respectively, according to the M07-A10 CLSI guideline [39] and our previous work [47,48]. The microbe suspensions were prepared from fresh cultures cultivated overnight in different fermentation broths for both bacteria (10 g/L peptone, 5 g/L NaCl, 5 g/L yeast extract) and yeast (20 g/L peptone, 10 g/L yeast extract, 20 g/L glucose) at 37 °C, and their concentrations were set to 4 × 10^5^ cells/mL with sterile media. After that, 100 μL of suspension containing the bacterial or yeast cells and 100 μL of the test solutions were dispensed into each well of 96-well plates and incubated for 24 h at 37 °C and 32 °C for bacteria and yeasts, respectively. The blank sample was a mixture of 100 μL broth and 100 μL of a test solution used for background correction, while 100 μL of microbial suspension supplemented with 100 μL of 1% DMSO was applied as a negative control. For the positive control, ampicillin (Sigma, Kawasaki, Japan) or nystatin (Sigma) was applied for bacteria or fungi, respectively, at two final concentration levels (50 μg/mL and 5 μg/mL). The inhibitory effects were calculated as the percentage of the negative control after blank correction using the following formula: inhibitory effect (%) = 100 − ((OD_test_ − OD_blank_)/OD_negative_) × 100). Here, OD_test_ represents the OD values of the test solution, OD_negative_ represents the OD values of the negative control, and OD_blank_ represents the OD values of blank samples.

### 3.5. General Procedure for Molecular Docking

Protein structures were obtained from the Protein Data Bank (PDB code: 6M1S, resolution 2.25 Å). Protein preparation steps, including the removal of water molecules, addition of polar hydrogens, and assignment of Kollman charges, were carried out using AutoDock Tools 1.5.7 [49]. The energy minimization of the protein structures was performed using Chimera 1.18. The grid box was centered on the co-crystallized native ligand’s coordinates (X = 22.036, Y = 1.330, Z = 41.041) with dimensions of 30 × 30 × 30 Å. Ligand structures were drawn in ChemDraw 16.0, optimized, and prepared using AutoDock Tools. Docking simulations were conducted using AutoDock Vina [40] with the protein kept rigid and the ligand flexible. Validation involved redocking the native co-crystallized ligand, achieving RMSD values below 3.0 Å for the top 5 scored compounds [50]. The docking results were visualized and analyzed using Discovery Studio (version 16.0, BIOVIA, San Diego, CA, USA, Dassault Systèmes), which also generated 3D and 2D interaction diagrams.

## 4. Conclusions

A series of gibberellic acid-based 2,4-diaminopyrimidines was designed and synthesized from commercially available gibberellic acid. The antimicrobial activity of the prepared compounds was explored in *B. subtilis*, *S. aureus*, *E. coli*, and *P. aeruginosa* bacteria, as well as in *C. krusei* and *C. albicans* fungi. The best diaminopyrimidine derivative **29** with 5-chloro and 2-((4-(trifluoromethyl)phenyl)amino substituents showed reliable antimicrobial activities against both *P. aeruginosa* and *C. krusei* and was proven to be an excellent lead molecule for further investigation. Similarly, compound **20** expressed a selective antibacterial effect against *P. aeruginosa*, while compound **28** was effectively selective against fungus. The *P. aeruginosa* DNA gyrase inhibitor activity of compounds **20** and **29**, as a possible pathway, was examined by molecular interactions between the active compounds and their potential targets using molecular docking studies, which were conducted using the AutoDock Vina program.

## Figures and Tables

**Table 1:** Antimicrobial activities of the synthesized compounds.

				Inhibitory Effect (%) ± RSD (%)			
		Gram-Positive		Gram-Negative		Yeast	
Compound	Conc. (μg/mL)	*B. subtilis*	*S. aureus*	*E. coli*	*P. aeruginosa*	*C. krusei*	*C. albicans*
**Ampicillin**	50	99.7 ± 2.2	99.1 ± 1.2	99.1 ± 1.2	99.1 ± 1.2	-	-
	5	100.3 ± 0.2	100.0 ± 2.1	100.0 ± 2.1	100.0 ± 2.1	-	**-**
**Nystatine**	50	-	-	-	-	100.0 ± 4.6	100.0 ± 1.0
	5	-	-	-	-	77.5 ± 6.4	100.0 ± 2.0
**4**	50	100.0 ± 5.9	100.0 ± 1.9	100.0 ± 2.6	44.5 ± 1.6	86.6 ± 22.0	48.3 ± 5.5
	5	28.7 ± 9.4	19.8 ± 7.9	-	18.3 ± 3.8	95.0 ± 6.3	32.5 ± 2.5
**5**	50	-	3.2 ± 3.0	15.4 ± 1.2	15.8 ± 1.9	-	4.6 ± 7.1
	5	15.6 ± 5.3	8.5 ± 4.4	-	14.9 ± 3.9	62.1 ± 2.2	3.9 ± 2.2
**17**	50	58.7 ± 3.1	12.3 ± 6.0	82.6 ± 2.1	22.8 ± 5.5	59.3 ± 4.6	7.7 ± 2.2
	5	69.3 ± 9.4	45.1 ± 6.7	42.4 ± 4.8	50.0 ± 7.7	41.8 ± 4.0	-
**18**	50	-	43.4 ± 5.3	-	31.0 ± 5.0	58.8 ± 9.3	13.8 ± 3.1
	5	11.7 ± 7.2	14.2 ± 4.0	-	49.4 ± 3.0	50.9 ± 17.8	10.6 ± 2.2
**19**	50	82.2 ± 4.8	50.6 ± 3.3	18.8 ± 2.3	19.8 ± 3.8	46.0 ± 8.5	-
	5	-	17.9 ± 1.9	13.3 ± 1.4	18.5 ± 2.0	-	-
**20**	50	23.4 ± 3.7	26.1 ± 4.6	17.2 ± 4.9	81.6 ± 4.2	12.4 ± 3.0	4.9 ± 2.9
	5	-	15.8 ± 4.0	9.5 ± 2.2	100.0 ± 1.4	12.9 ± 2.8	3.2 ± 3.3
**21**	50	-	23.6 ± 2.8	27.2 ± 5.6	7.6 ± 1.2	79.2 ± 1.6	71.8 ± 1.1
	5	-	17.0 ± 4.5	-	12.0 ± 4.1	39.6 ± 8.5	37.3 ± 1.1
**22**	50	-	9.5 ± 1.9	32.1 ± 2.4	-	58.6 ± 3.2	47.1 ± 0.6
	5	-	2.4 ± 2.5	-	9.3 ± 8.7	29.2 ± 10.3	32.1 ± 0.6
**23**	50	-	-	48.1 ± 2.5	-	57.4 ± 5.7	49.8 ± 1.1
	5	-	20.1 ± 6.8	-	4.6 ± 1.9	20.2 ± 2.3	21.4 ± 1.3
**24**	50	-	-	-	-	56.8 ± 9.0	58.1 ± 1.1
	5	-	5.9 ± 1.1	-	10.6 ± 1.7	-	35.3 ± 0.5
**25**	50	-	-	-	10.1 ± 2.2	42.3 ± 0.7	27.0 ± 0.3
	5	-	24.1 ± 5.4	-	3.4 ± 0.7	-	22.5 ± 0.3
**26**	50	-	-	17.5 ± 6.5	15.5 ± 3.1	18.5 ± 13.6	5.2 ± 1.3
	5	-	16.7 ± 6.4	-	42.3 ± 12.9	-	-
**27**	50	-	-	22.7 ± 1.3	30.2 ± 2.5	61.3 ± 10.8	-
	5	39.5 ± 7.7	19.8 ± 6.6	-	21.9 ± 5.5	100.0 ± 21.3	56.3 ± 2.0
**28**	50	96.5 ± 5.2	94.7 ± 1.6	97.5 ± 1.4	33.6 ± 3.9	26.7 ± 8.1	7.6 ± 1.8
	5	-	-	-	21.9 ± 2.5	100.0 ± 3.0	56.1 ± 0.7
**29**	50	100.7 ± 1.8	50.1 ± 5.5	32.4 ± 12.7	93.2 ± 3.4	84.0 ± 5.2	19.1 ± 0.6
	5	-	-	5.7 ± 5.1	100.0 ± 1.4	43.7 ± 18.9	-
**30**	50	-	17.0 ± 8.3	-	-	41.7 ± 2.7	48.1 ± 0.9
	5	-	-	-	-	17.7 ± 6.5	34.5 ± 2.3
**31**	50	-	100.0 ± 8.1	61.2 ± 3.3	49.3 ± 2.5	54.5 ± 4.4	60.2 ± 0.4
	5	-	17.0 ± 2.8	27.5 ± 13.9	-	27.0 ± 4.2	18.9 ± 0.5
**32**	50	-	45.9 ± 6.1	40.1 ± 7.8	15.9 ± 0.6	53.8 ± 2.7	60.1 ± 2.1
	5	-	2.7 ± 2.2	12.3 ± 2.1	7.4 ± 4.9	29.2 ± 10.3	28.6 ± 0.8
**33**	50	-	100.0 ± 2.8	60.7 ± 18	38.4 ± 0.8	57.0 ± 6.9	67.3 ± 0.9
	5	-	11.1 ± 4.0	22.3 ± 1.3	12.4 ± 3.1	-	27.0 ± 0.6
**34**	50	-	21.7 ± 1.4	18.4 ± 2.1	-	50.7 ± 3.2	29.6 ± 0.3
	5	13.1 ± 7.6	25.8 ± 3.0	-	-	26.1 ± 9.4	31.6 ± 0.3

## Data Availability

The original contributions presented in this study are included in the article and Supplementary Material. Further inquiries can be directed to the corresponding author.

## Supplementary Materials

The following supporting information can be downloaded at https://www.mdpi.com/article/10.3390/ph18020168/s1: Figures S1–S131: ^1^H, ^13^C, ^19^F NMR, COSY, NOESY, HSQC, and HMBC spectra of new compounds; Figures S132–S134 and Tables S1–S4: docking study; Figure S135: 2D-diagram showing interactions between the best pose of compound **30** and *P. aeruginosa* DNA gyrase (PDB code: 6M1S); Figure S136: 3D presentation showing the binding location and accumulated poses predicted by the software of the original ligand and *P. aeruginosa* DNA gyrase (PDB code: 6M1S); Figure S137: 3D presentation showing the binding location and accumulated poses predicted by the software of compounds **20** and *P. aeruginosa* DNA gyrase (PDB code: 6M1S); Figure S138: 3D presentation showing the binding location and accumulated poses predicted by the software of compounds **29** and *P. aeruginosa* DNA gyrase (PDB code: 6M1S); Figure S139: 3D presentation showing the binding location and accumulated poses predicted by the software of compounds **30** and *P. aeruginosa* DNA gyrase (PDB code: 6M1S).

## Abbreviations

The following abbreviations are used in this manuscript:

MDPI	Multidisciplinary Digital Publishing Institute
DOAJ	Directory of open access journals
TLA	Three-letter acronym
LD	Linear dichroism
CuAAC reaction	Copper-catalyzed azide–alkyne cycloaddition
DCM	Dichloromethane
DIPEA	N,N-Diisopropylethylamine
DMAP	4-(Dimethylamino)pyridine
DMSO	Dimethyl sulfoxide
MW	Microwave
NMO	4-Methylmorpholine N-oxide
NOE	Nuclear Overhauser effect
SZMC	Szeged Microbiology Collection
TEA	Triethylamine
