# Supplementary material for: Stereoselective Synthesis and Antimicrobial Studies of Allo-Gibberic Acid-Based 2,4-Diaminopyrimidine Chimeras
Source: Pharmaceuticals (Basel). 2025 Jan 26;18(2):168. doi: 10.3390/ph18020168 (PMC11860145; doi:10.3390/ph18020168)

Supplementary data

Stereoselective synthesis and antimicrobial studies of *allo*-gibberic acid-based 2,4-diaminopyrimidine chimeras

Dima Depp [1], Noémi Regina Sebők [2], András Szekeres [2] and Zsolt Szakonyi [1*]

1 Institute of Pharmaceutical Chemistry, University of Szeged, Eötvös utca 6, H-6720 Szeged, Hungary; e-mail@e-mail.com

2 Department of Microbiology, University of Szeged, Közép fasor 52. H-6726 Szeged Hungary

*** Correspondence: szakonyi.zsolt@szte.hu; Tel.: +36-62-546809**

Content

^{1}H, ^{13}C, ^{19}F NMR, COSY, NOESY, HSQC, HMBC spectra of new compounds S3-S137

Docking study S138- S149

NMR Spectra of new compounds

(7S,8S,9aR,10R)-10-(Azidomethyl)-8-(hydroxymethyl)-1-methyl-4b,5,6,8,9,10-hexahydro-7H-7,9a-methanobenzo[a]azulene-7,8-diol (**5**)

Figure S1. ^{1}H-NMR of compound **5**

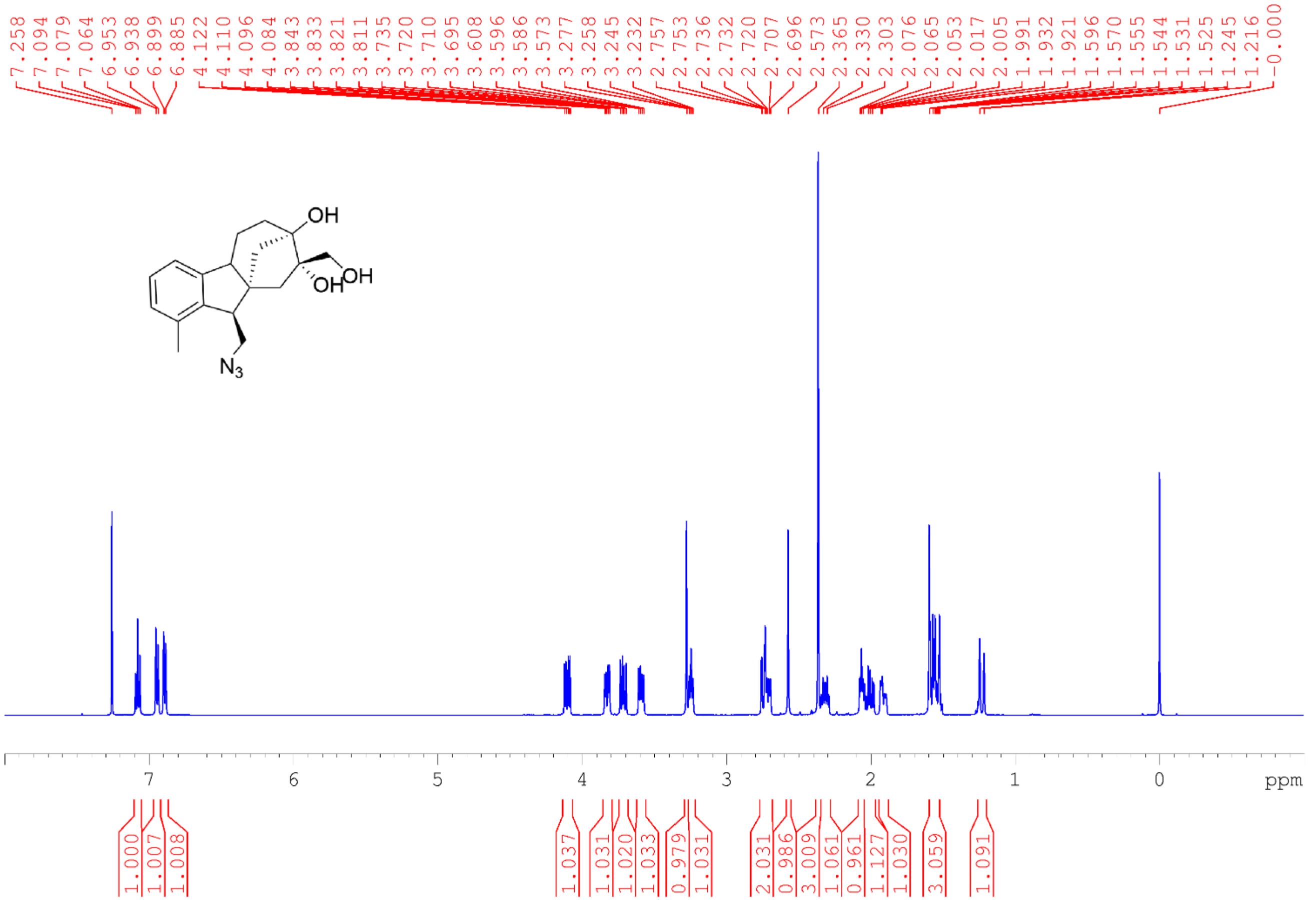

Figure S2.^{13}C-NMR of compound **5**

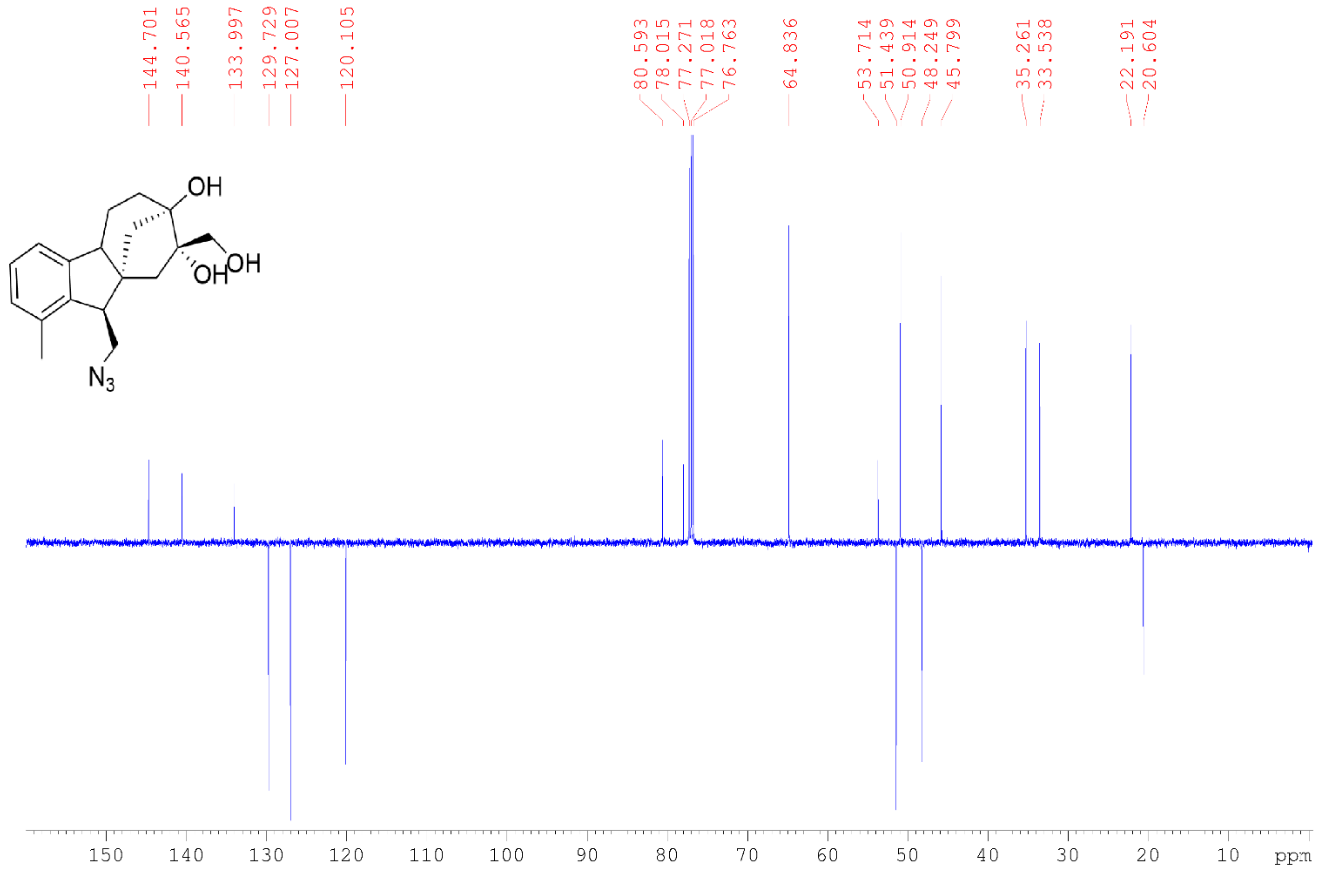

Figure S3. COSY-NMR of compound **5**

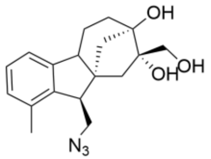

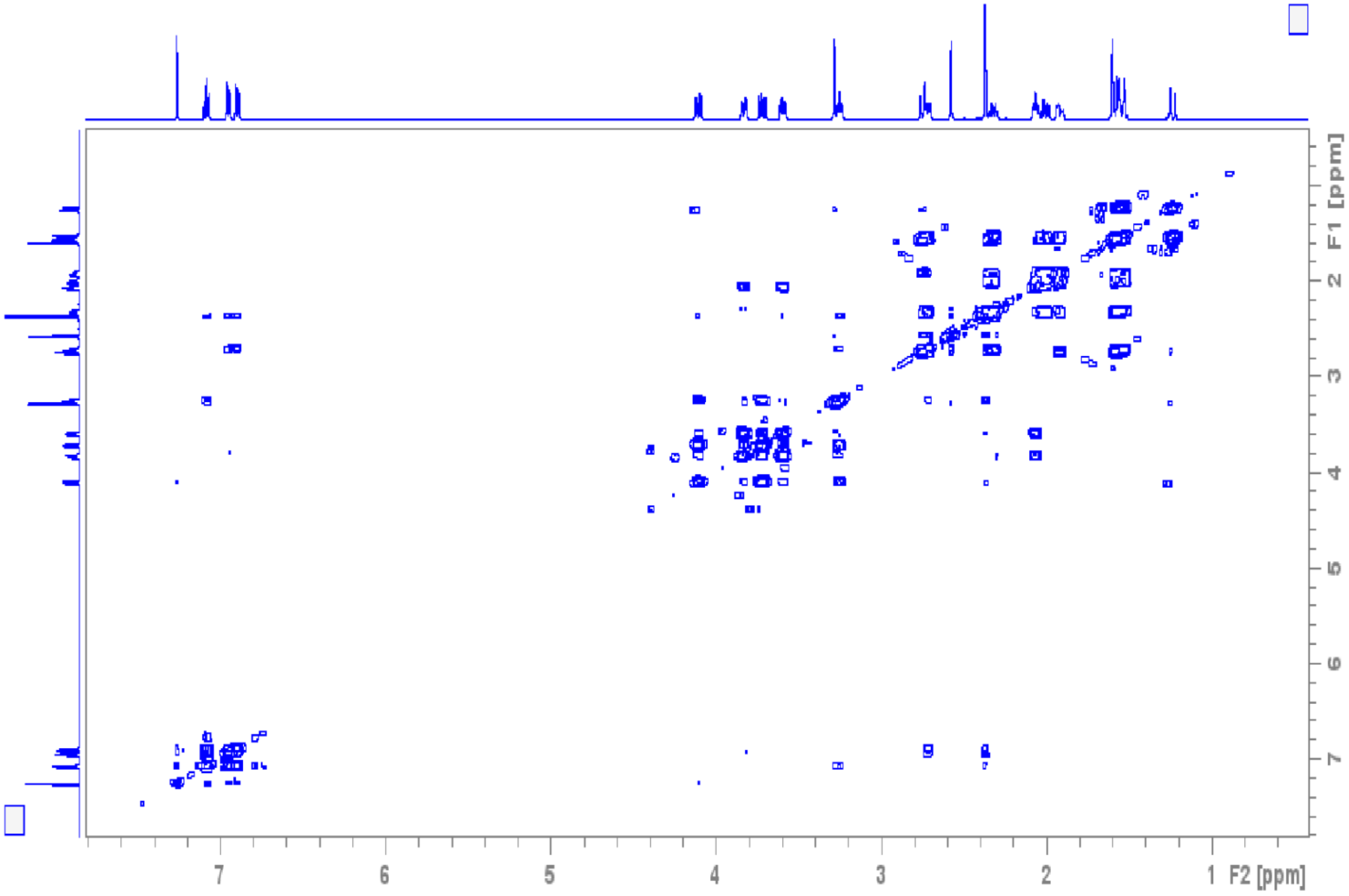

Figure S4. NOESY-NMR of compound **5**

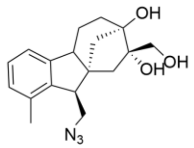

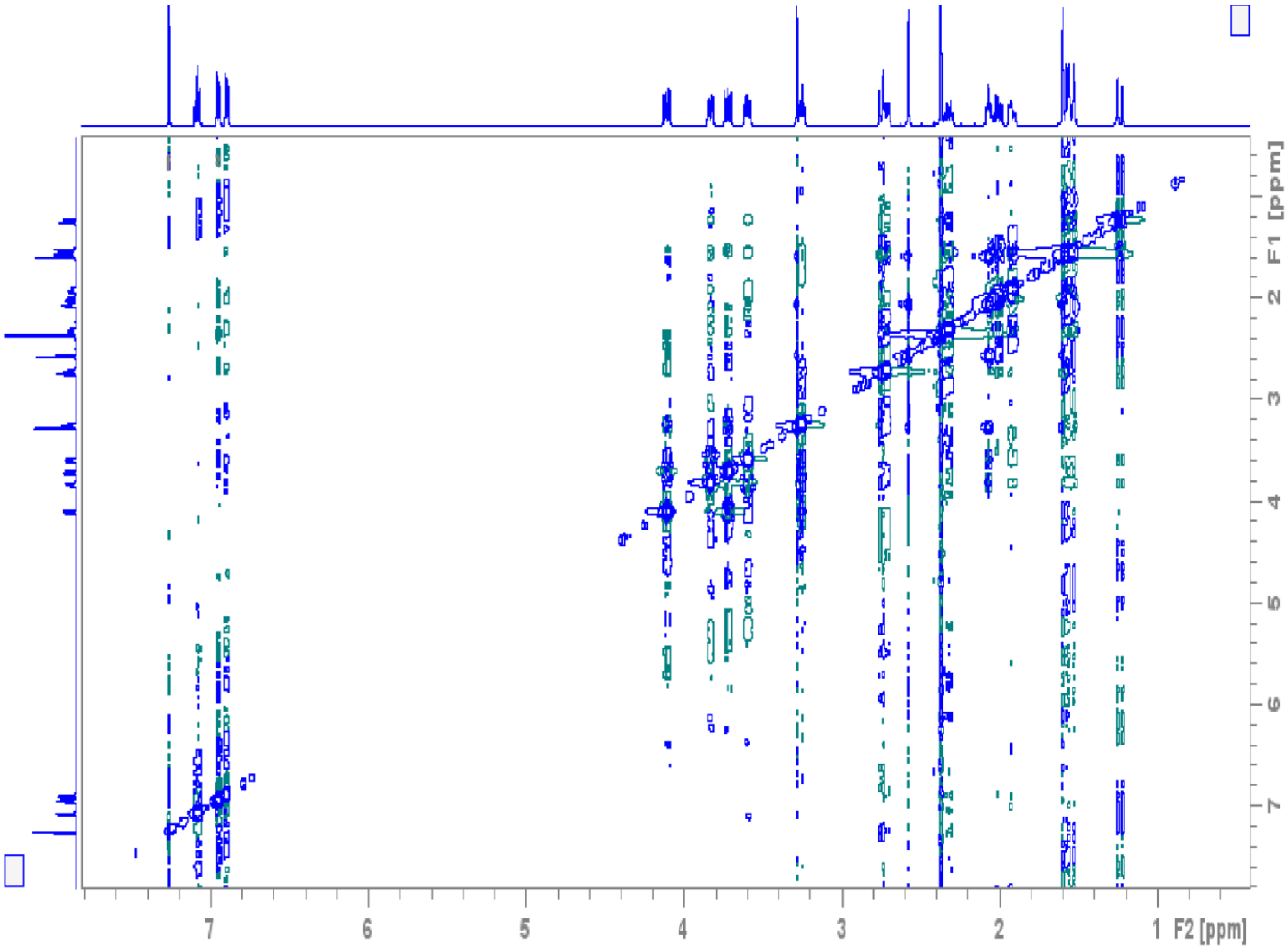

Figure S5. HSQC-NMR of compound **5**

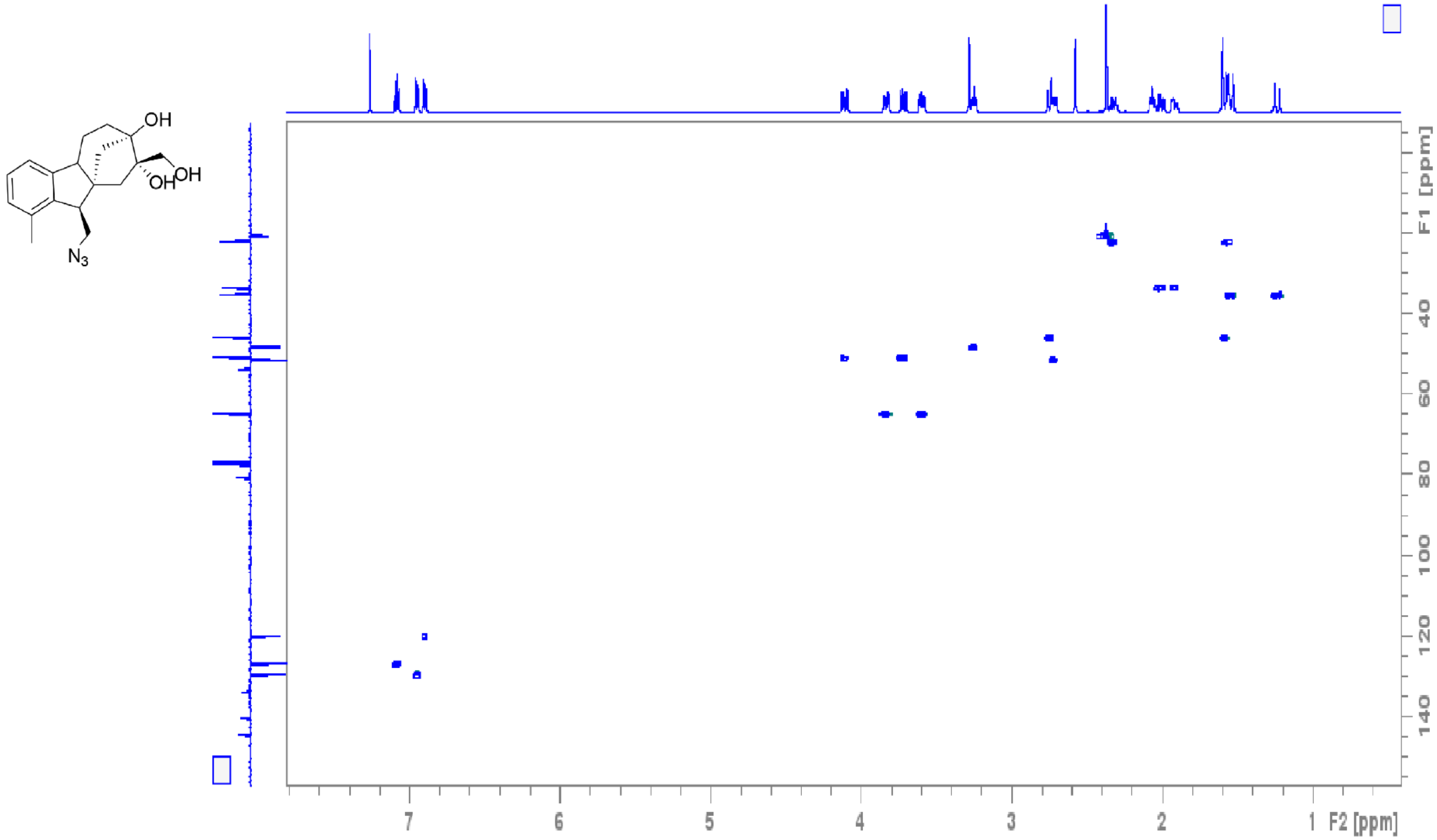

Figure S6. HMBC-NMR of compound **5**

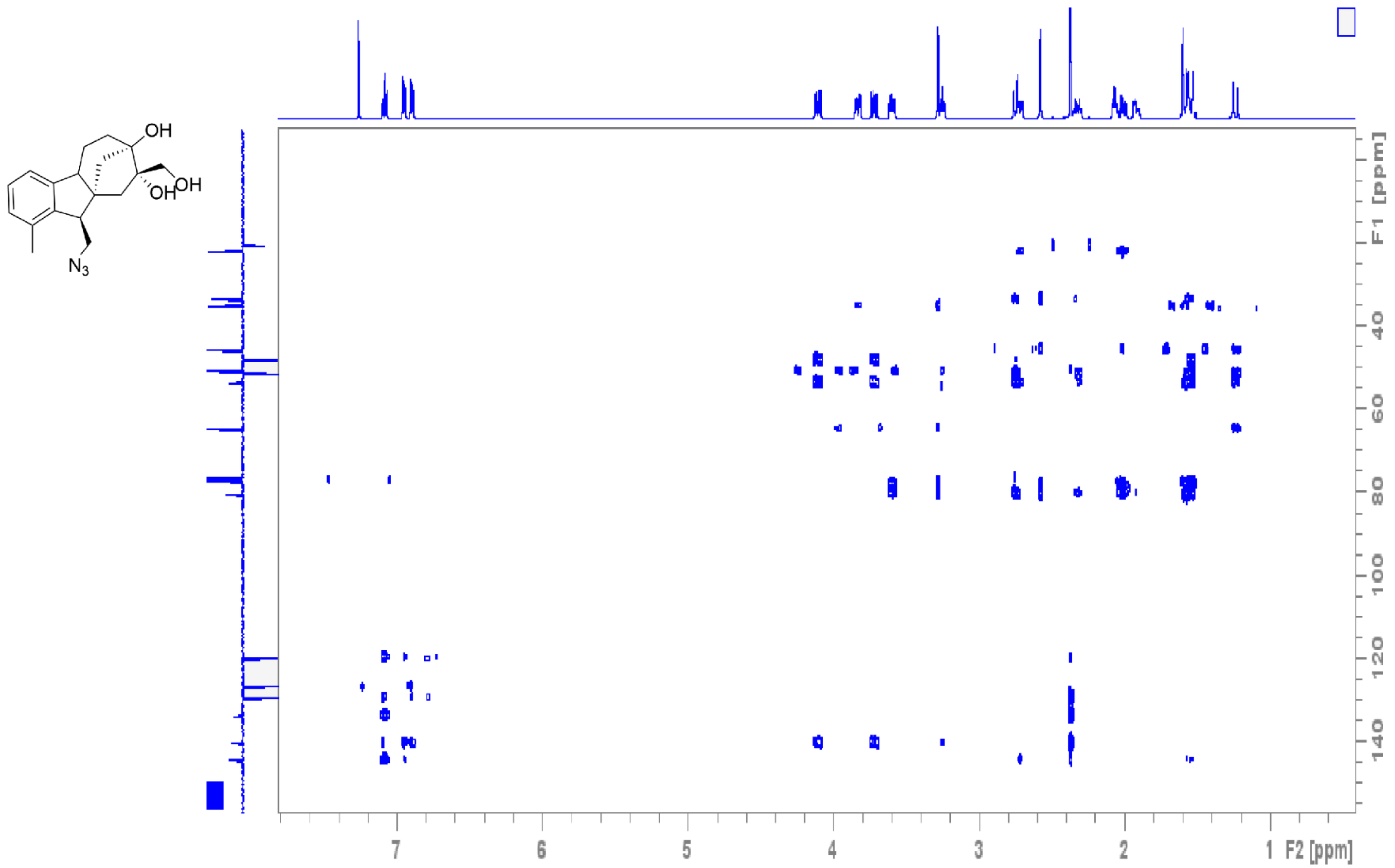

Methyl 4-((5-fluoro-4-(prop-2-yn-1-ylamino)pyrimidin-2-yl)amino)benzoate (**14**)

Figure. S7. ^{1}H-NMR of compound **14**

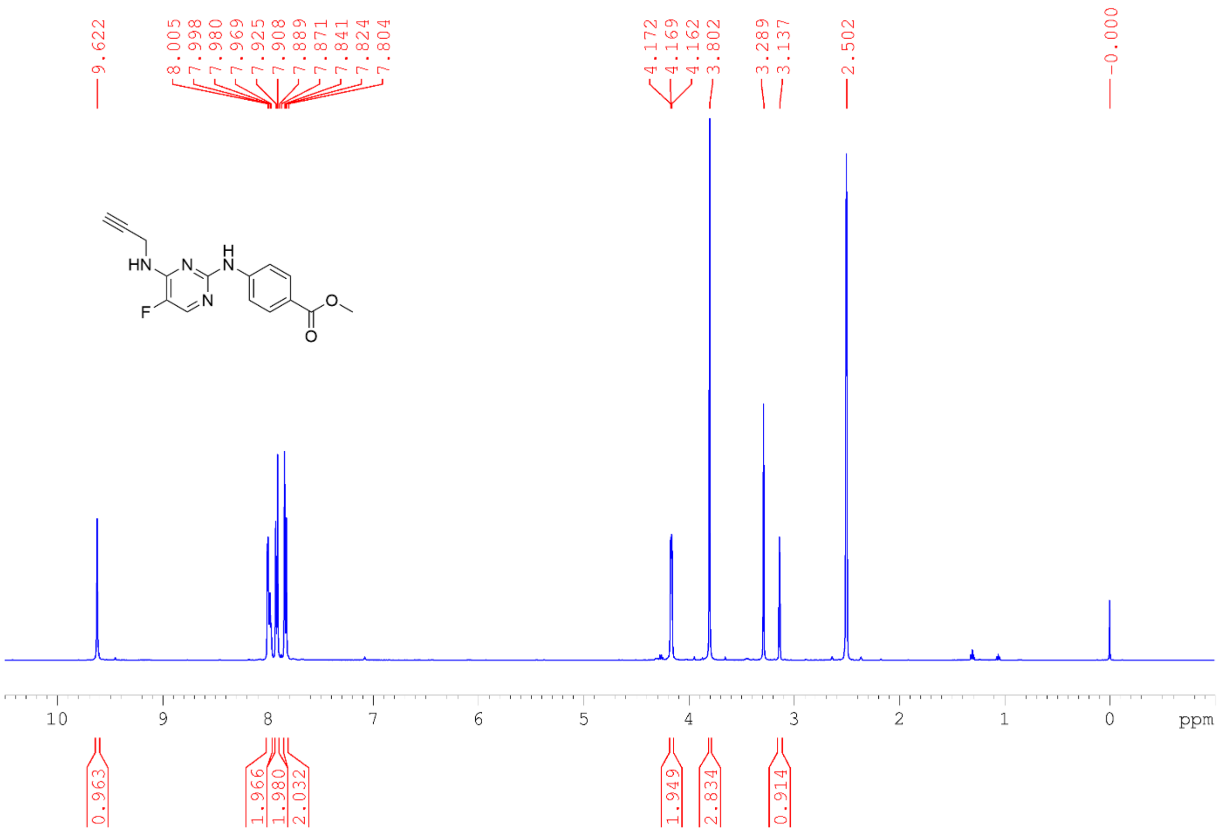

Figure. S8. ^{13}C-NMR of compound **14**

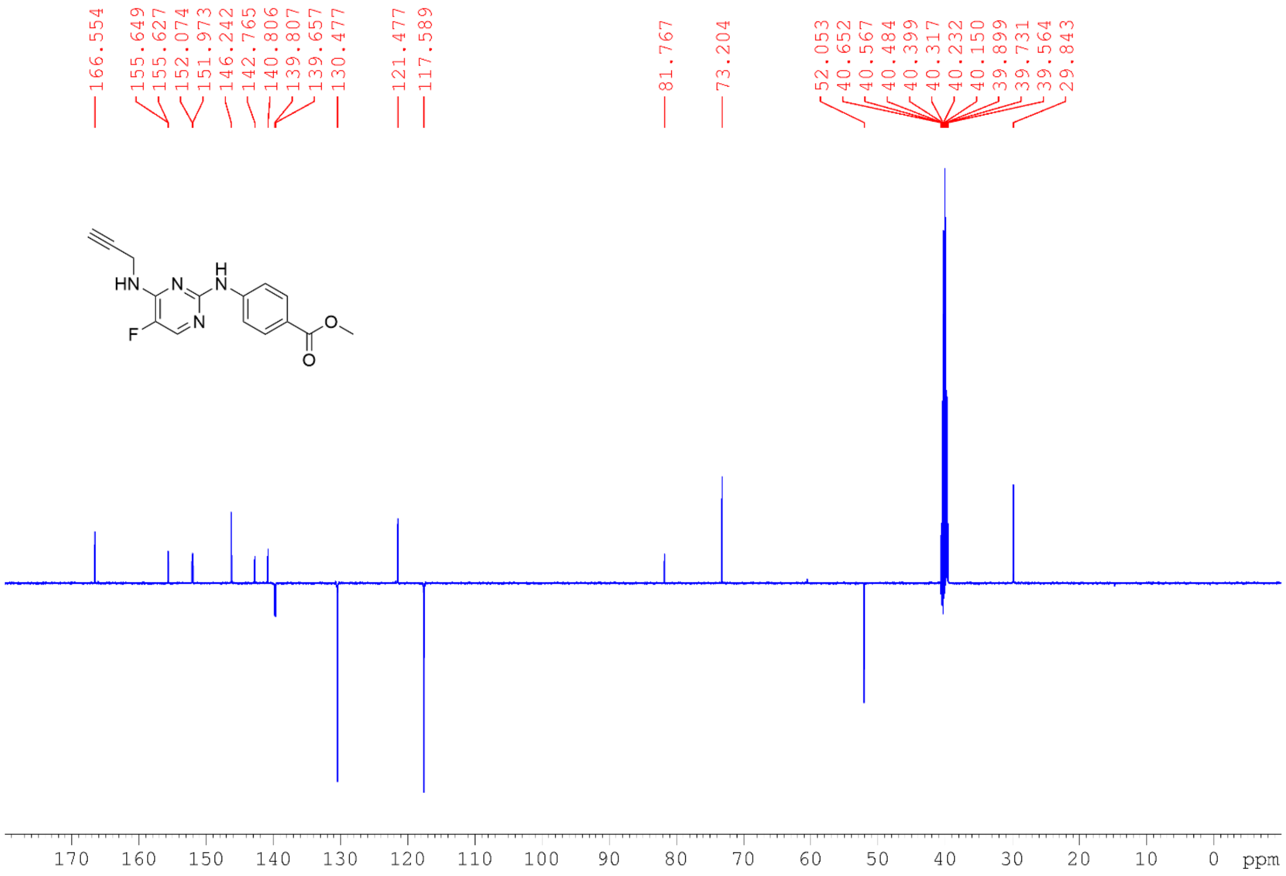

Figure. S9. ^{19}F-NMR of compound **14**

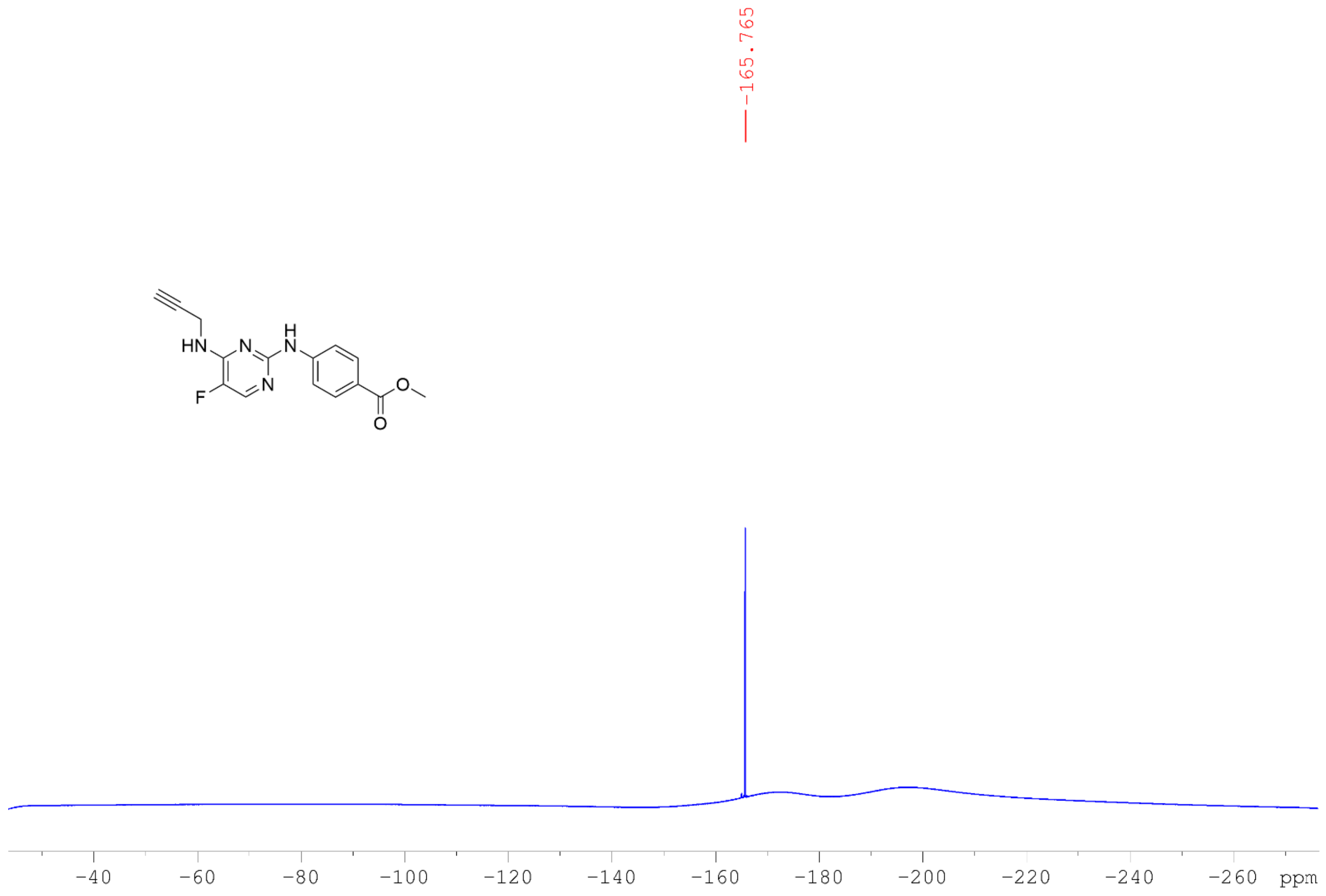

Methyl 4-((5-chloro-4-(prop-2-yn-1-ylamino)pyrimidin-2-yl)amino)benzoate (**15**)

Figure S10. ^{1}H-NMR of compound **15**

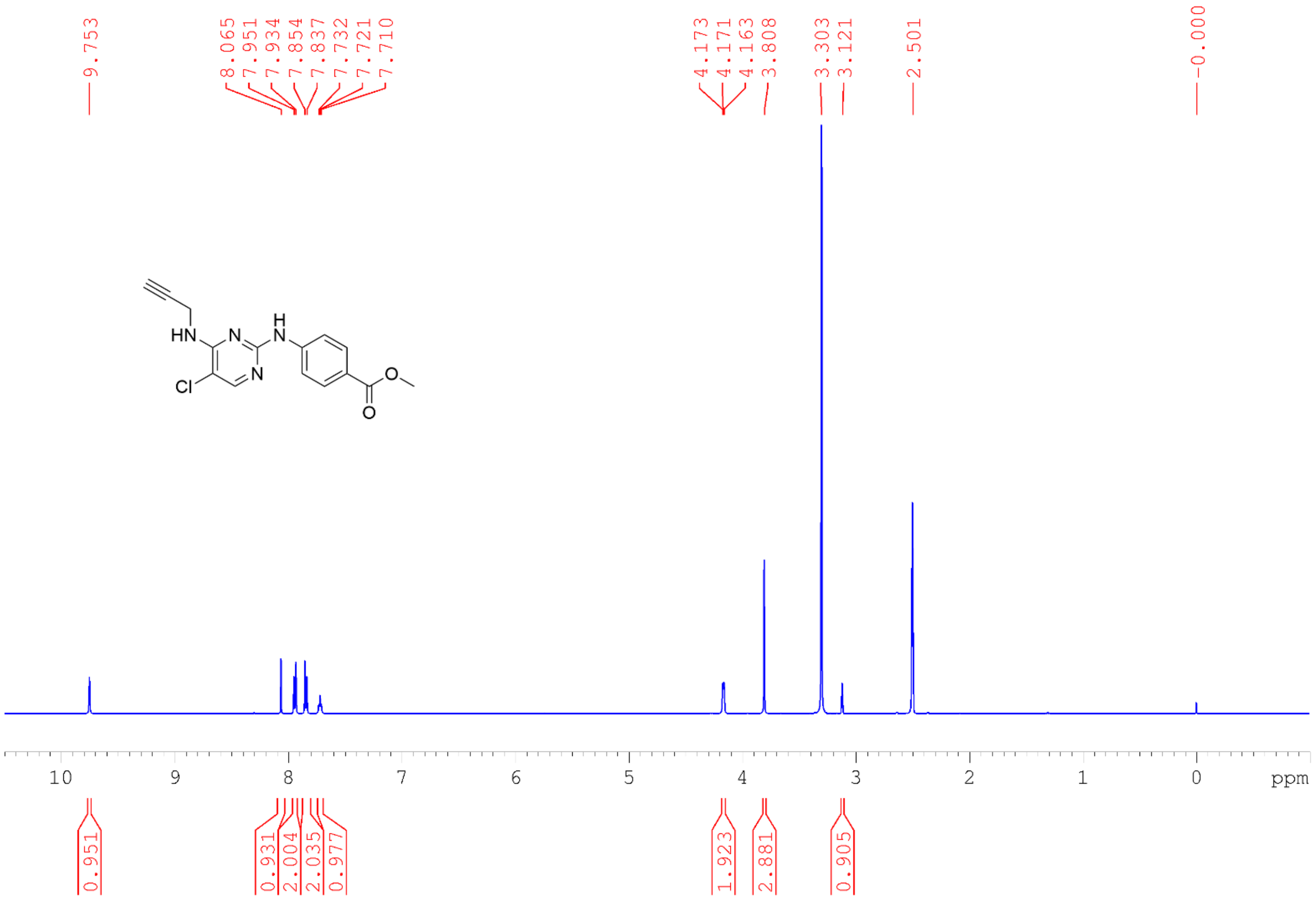

Figure S11. ^{13}C-NMR of compound **15**

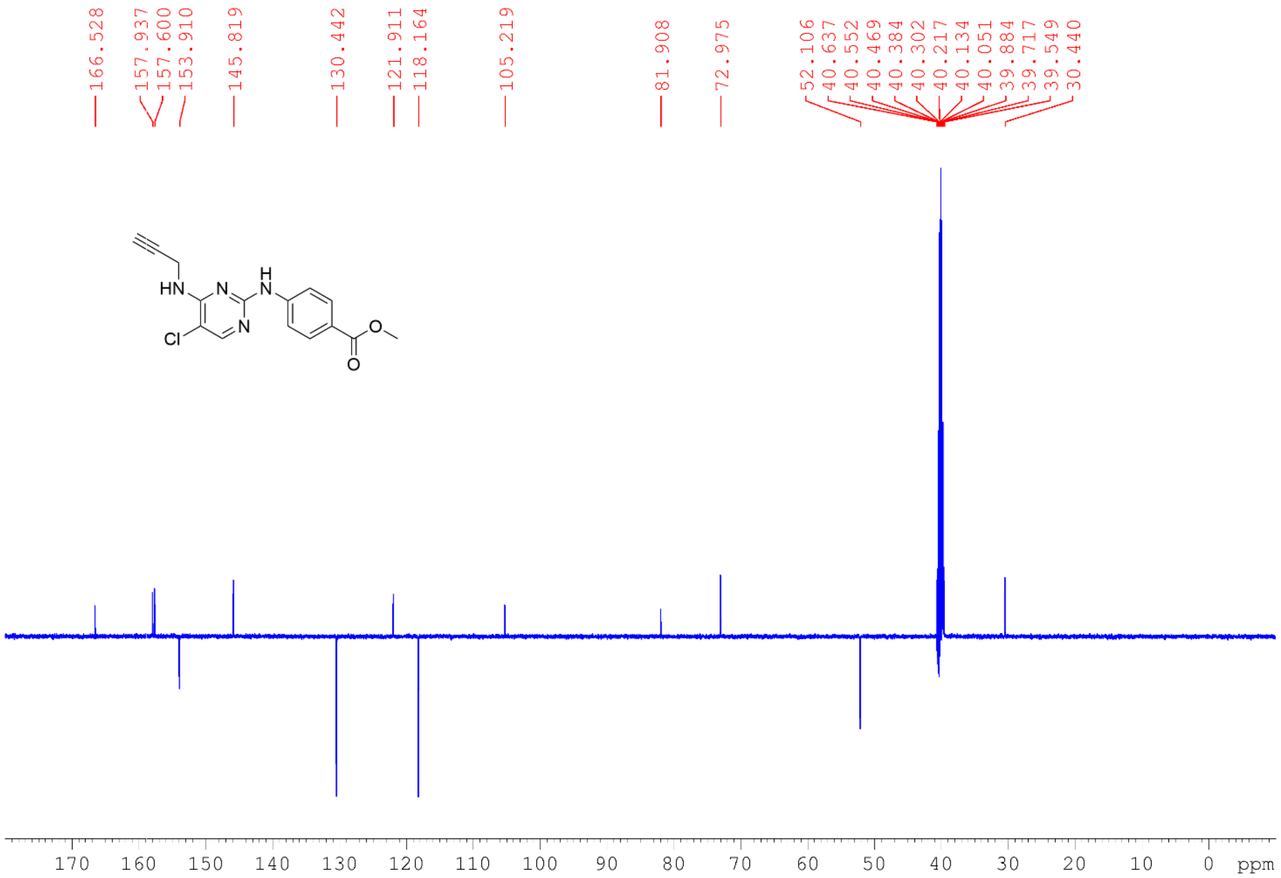

5-Fluoro-N2-(4-morpholinophenyl)-N4-(prop-2-yn-1-yl)pyrimidine-2,4-diamine (**16**)

Figure S12. ^{1}H-NMR of compound **16**

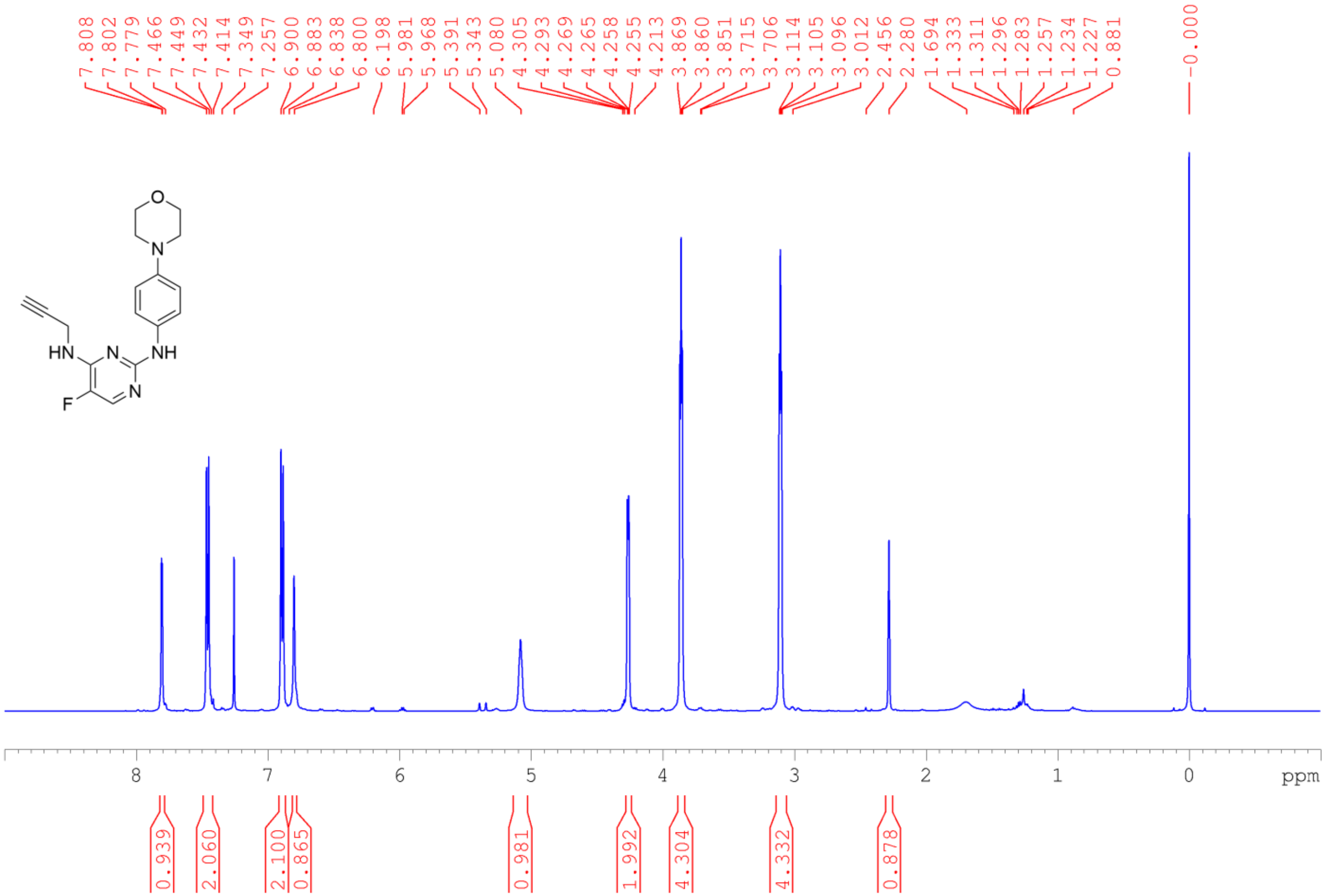

Figure S13. ^{13}C-NMR of compound **16**

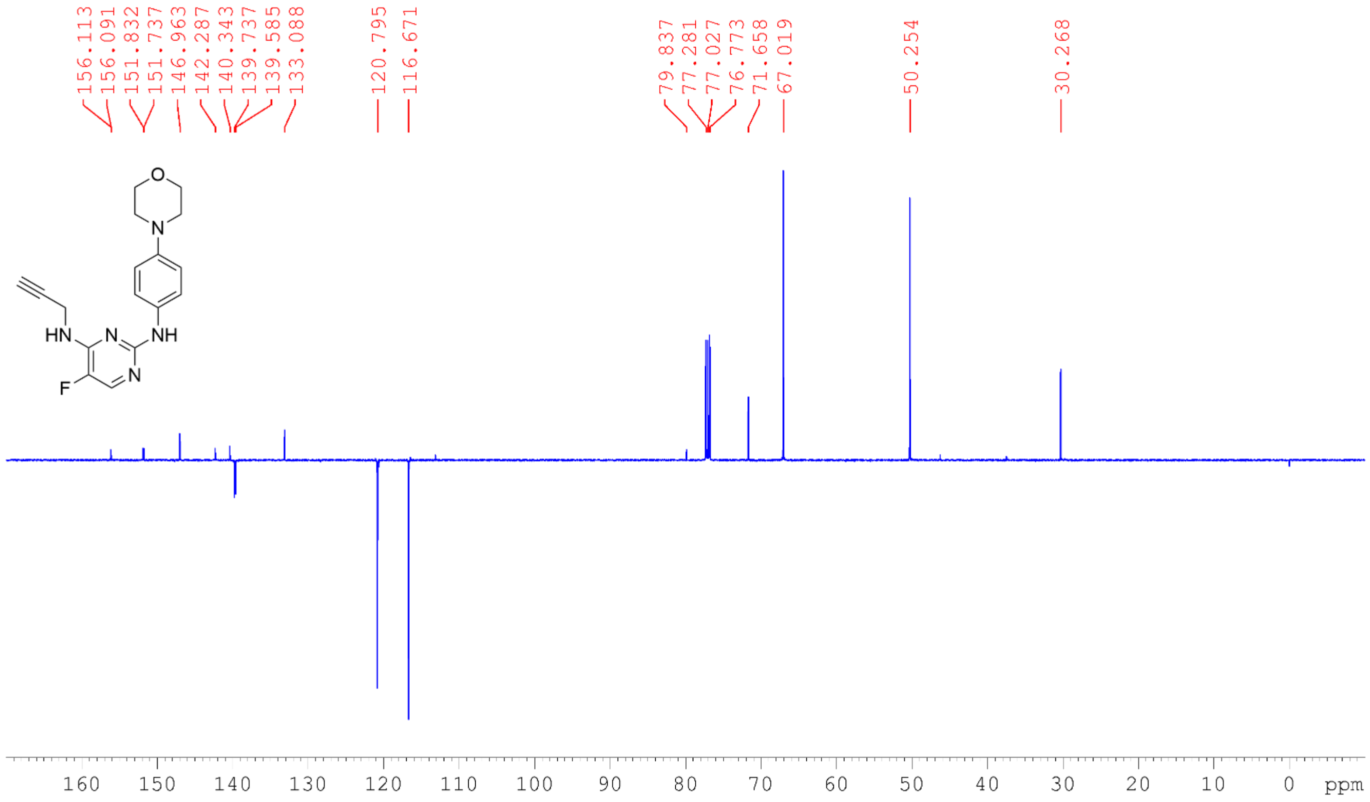

Figure S14. ^{19}F-NMR of compound **16**

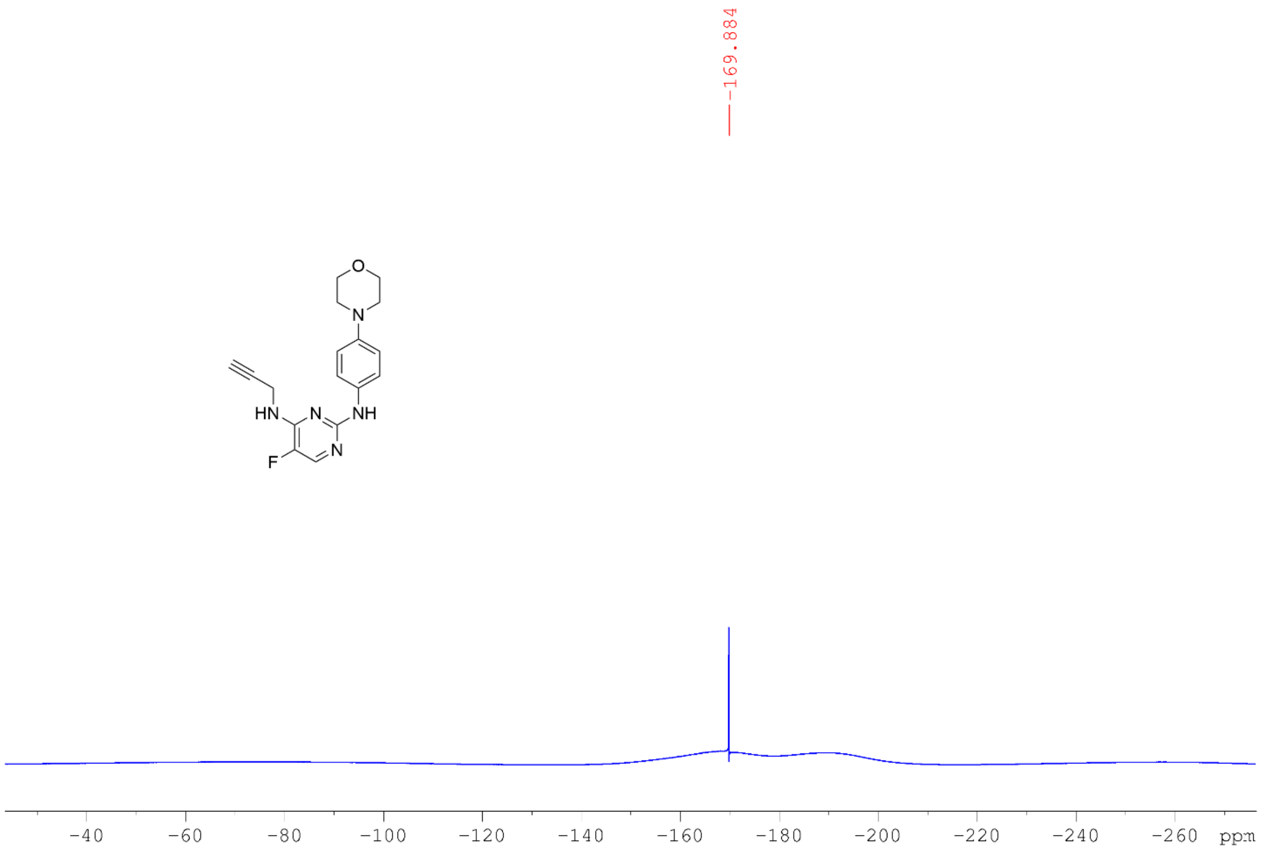

(7S,9aS,10R)-10-((4-(((2-Chloro-5-fluoropyrimidin-4-yl)amino)methyl)-1H-1,2,3-triazol-1-yl)me-thyl)-1-methyl-8-methylene-4b,5,6,8,9,10-hexahydro-7H-7,9a-methanobenzo[a]azulen-7-ol (**17**)

Figure S15. ^{1}H-NMR of compound **17**

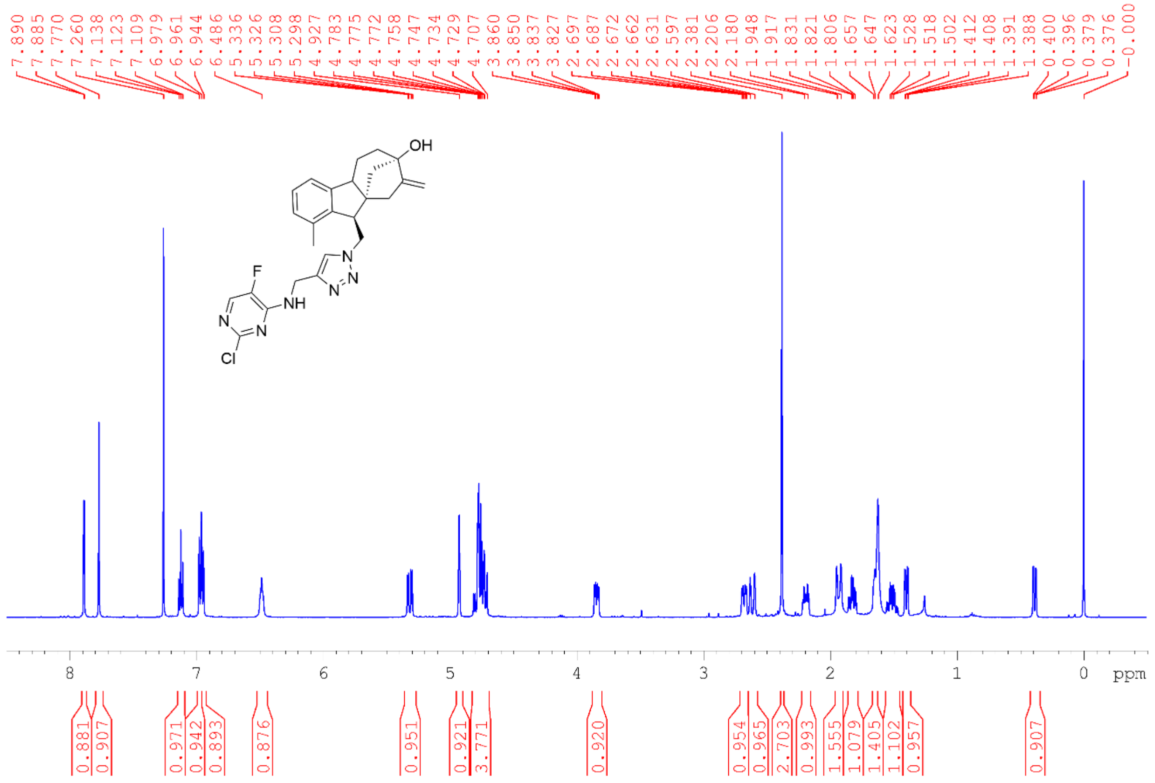

Figure S16. ^{13}C-NMR of compound **17**

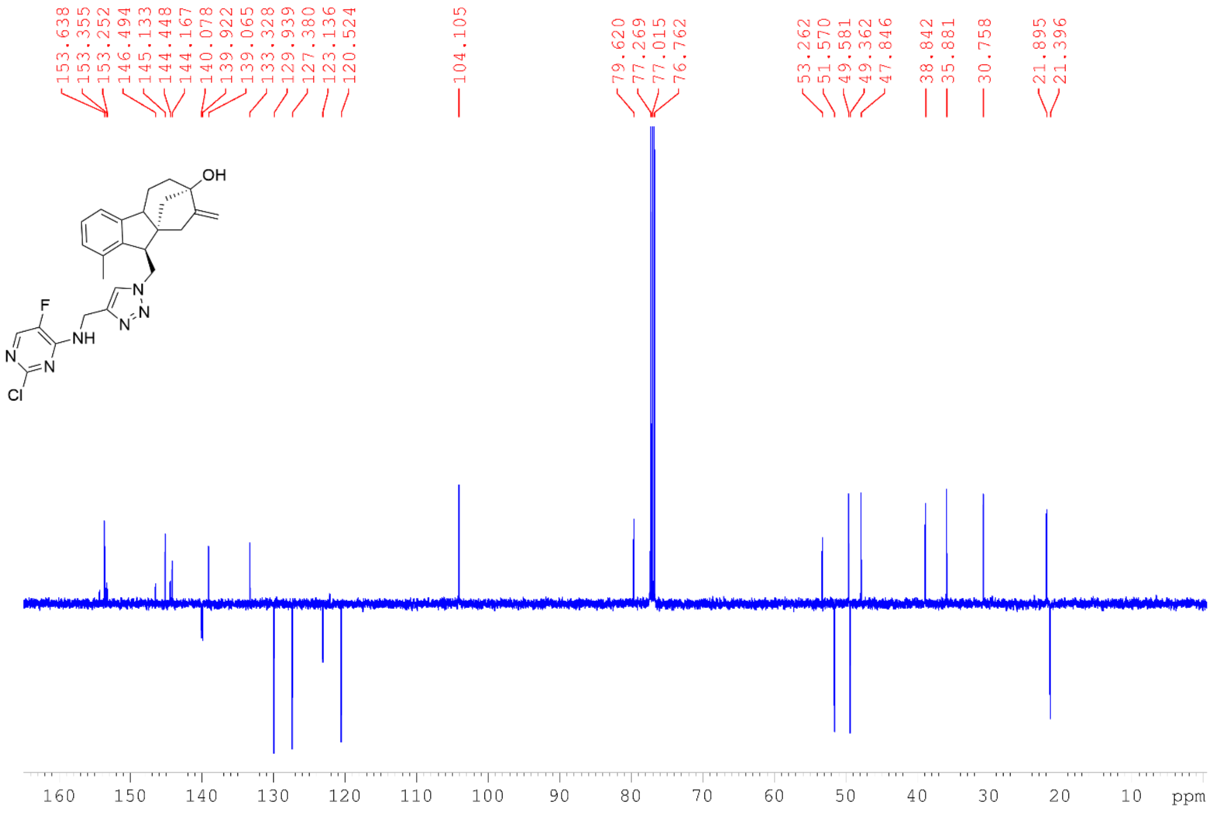

Figure S17. ^{19}F-NMR of compound **17**

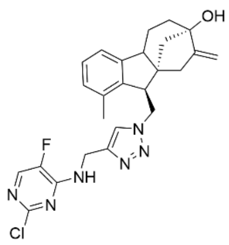

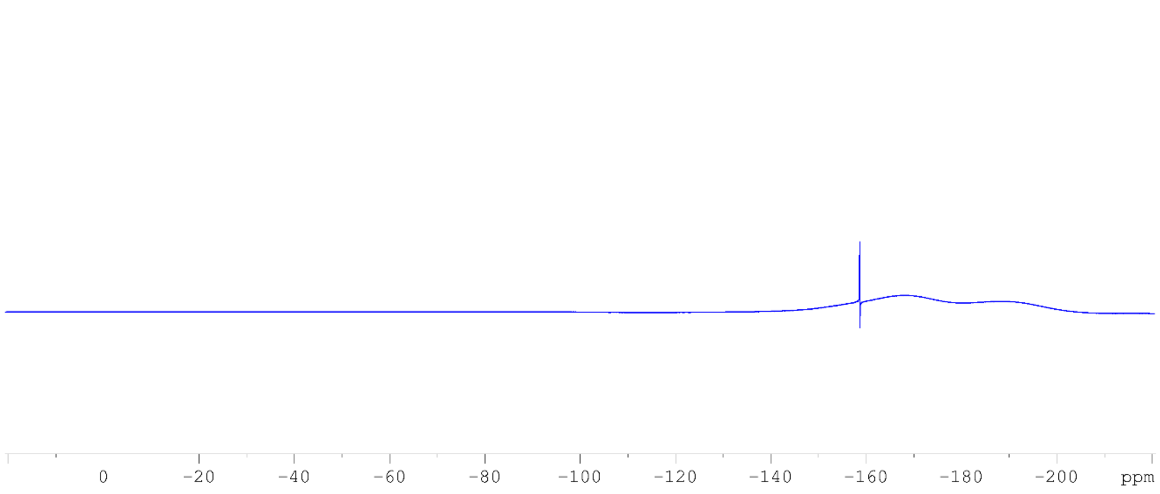

Figure S18. COSY-NMR of compound **17**

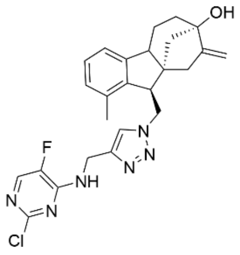

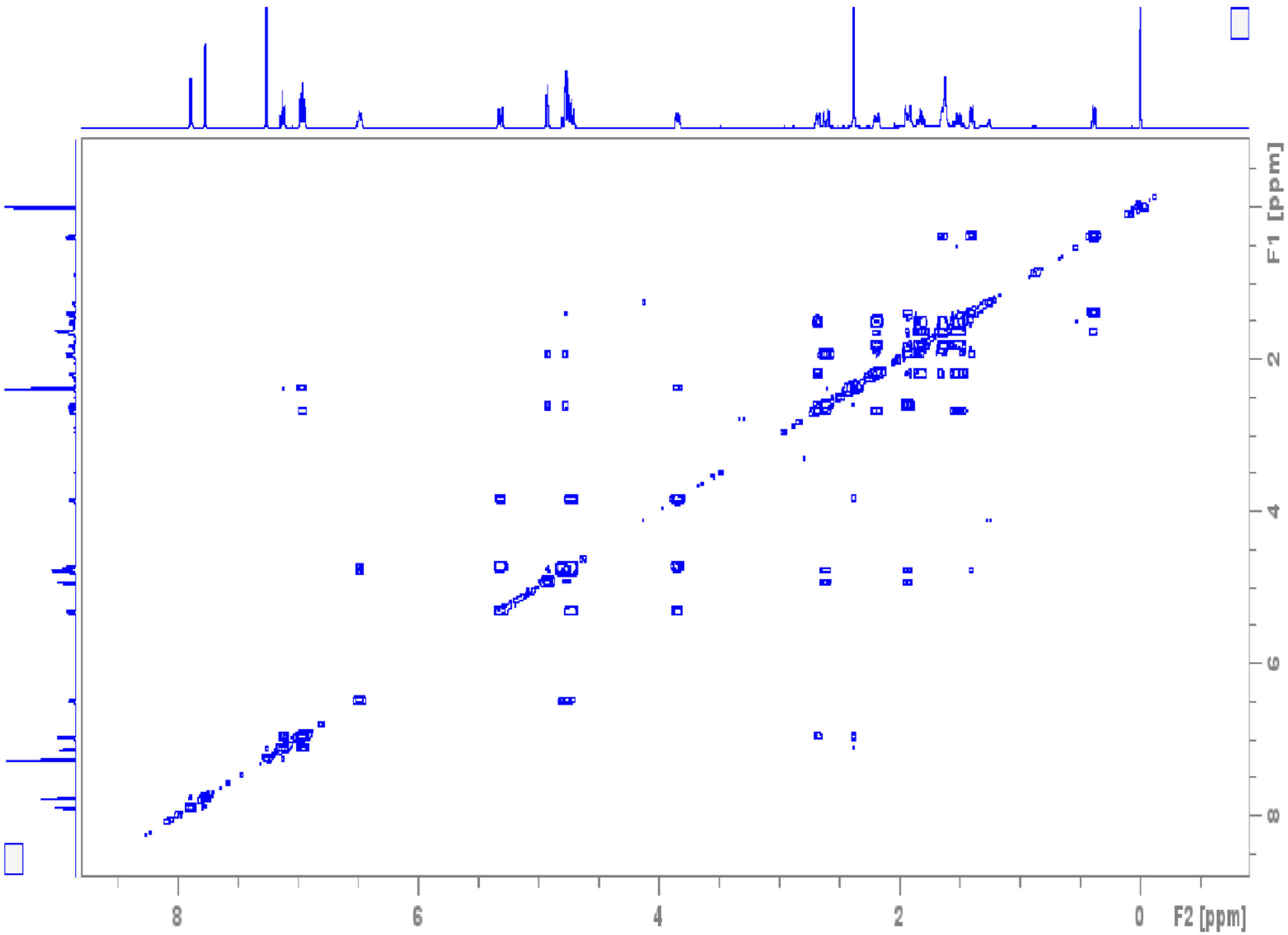

Figure S19. NOESY-NMR of compound **17**

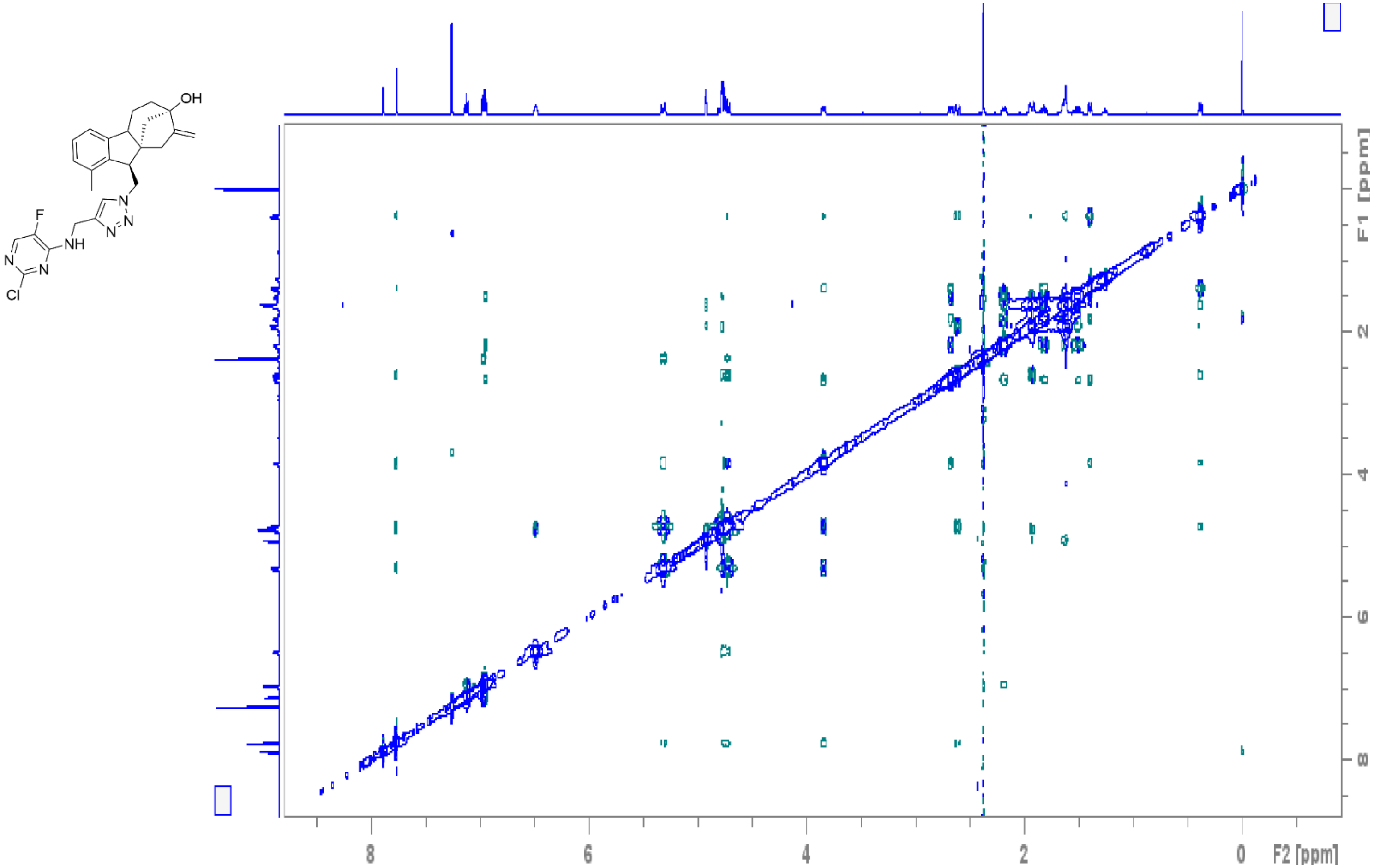

Figure S20. HSQC-NMR of compound **17**

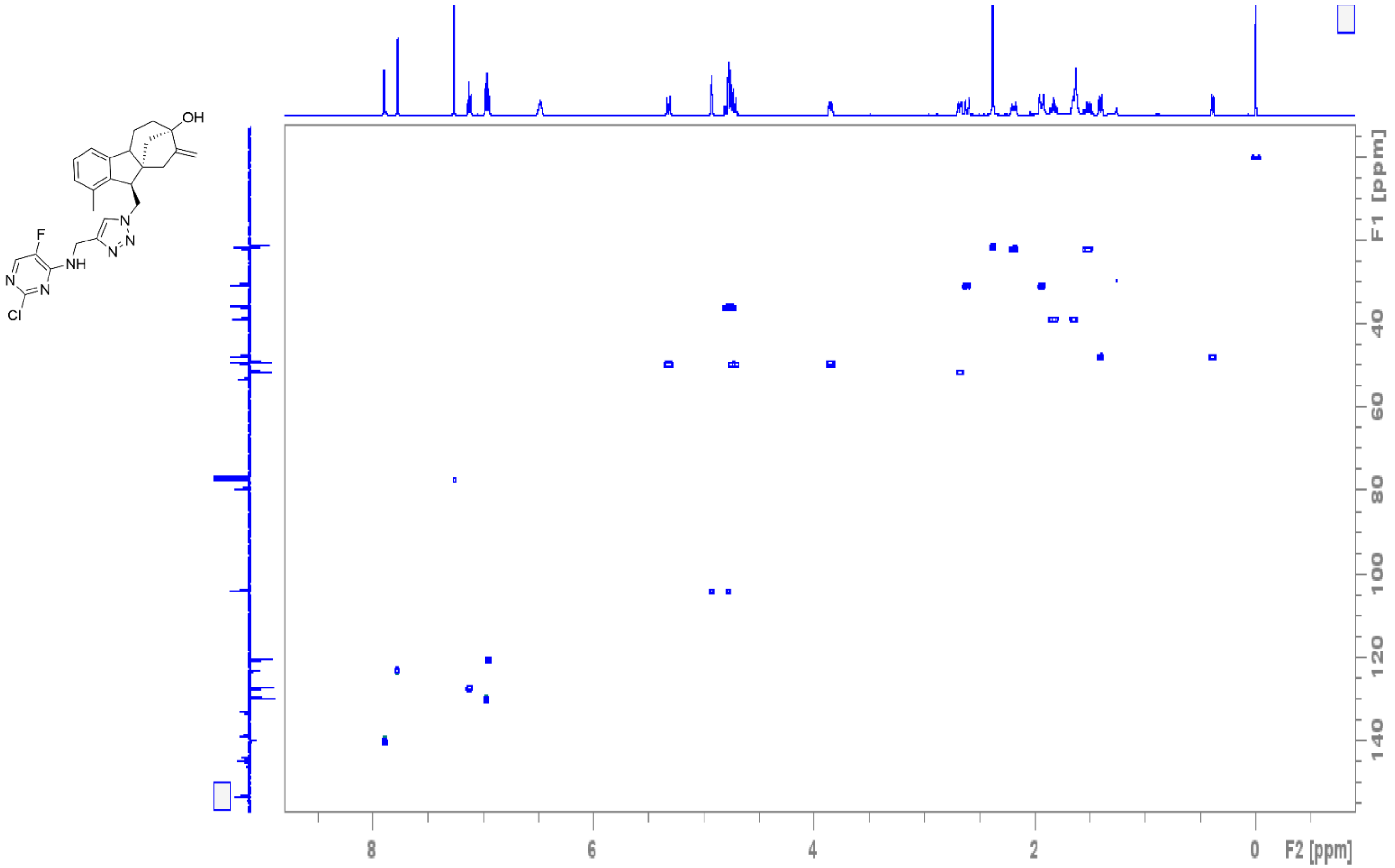

Figure S21. HMBC-NMR of compound **17**

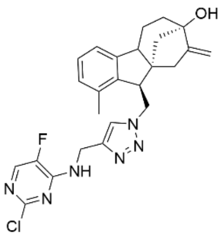

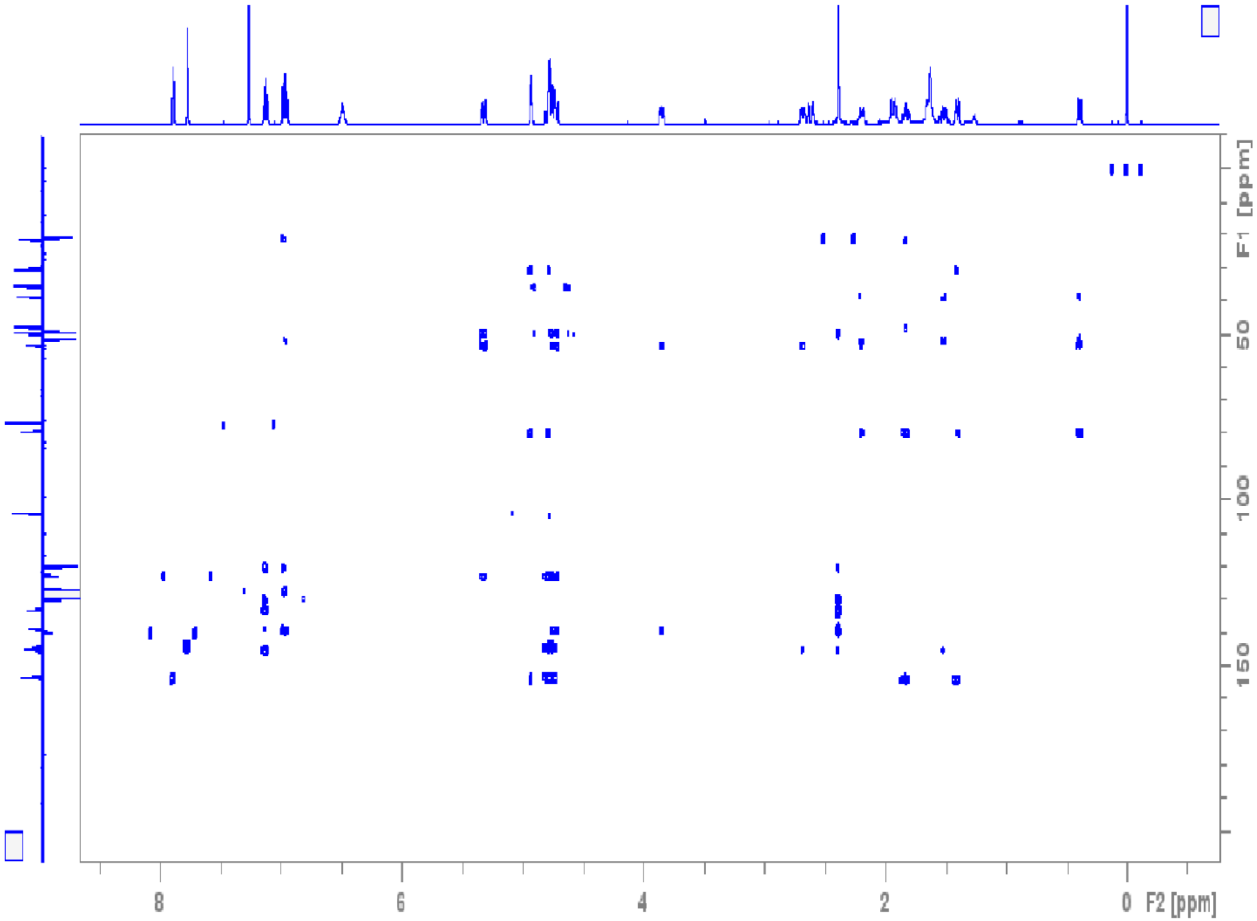

(7S,9aS,10R)-10-((4-(((2,5-Dichloropyrimidin-4-yl)amino)methyl)-1H-1,2,3-triazol-1-yl)methyl)-1-methyl-8-methylene-4b,5,6,8,9,10-hexahydro-7H-7,9a-methanobenzo[a]azulen-7-ol (**18**)

Figure S22. ^{1}H-NMR of compound **18**

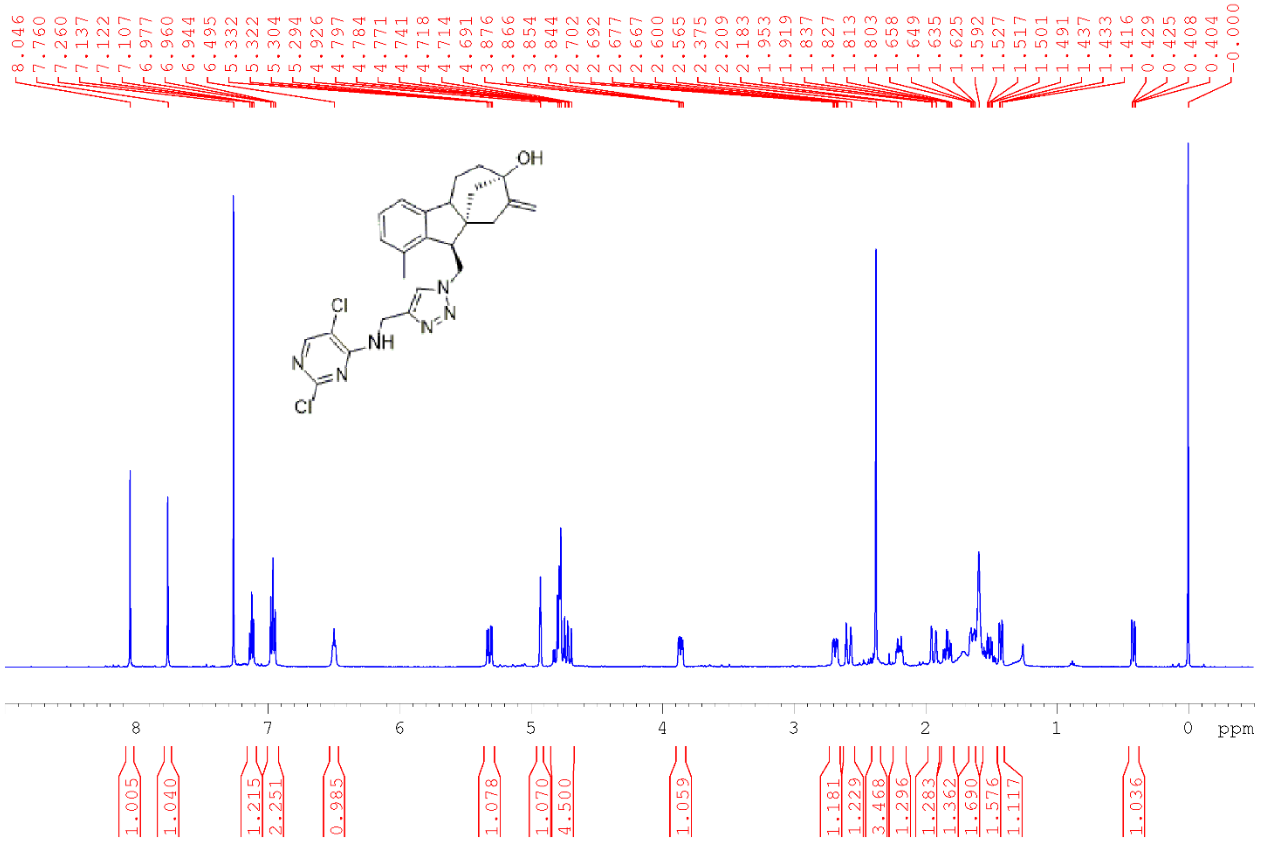

Figure S23. ^{13}C-NMR of compound **18**

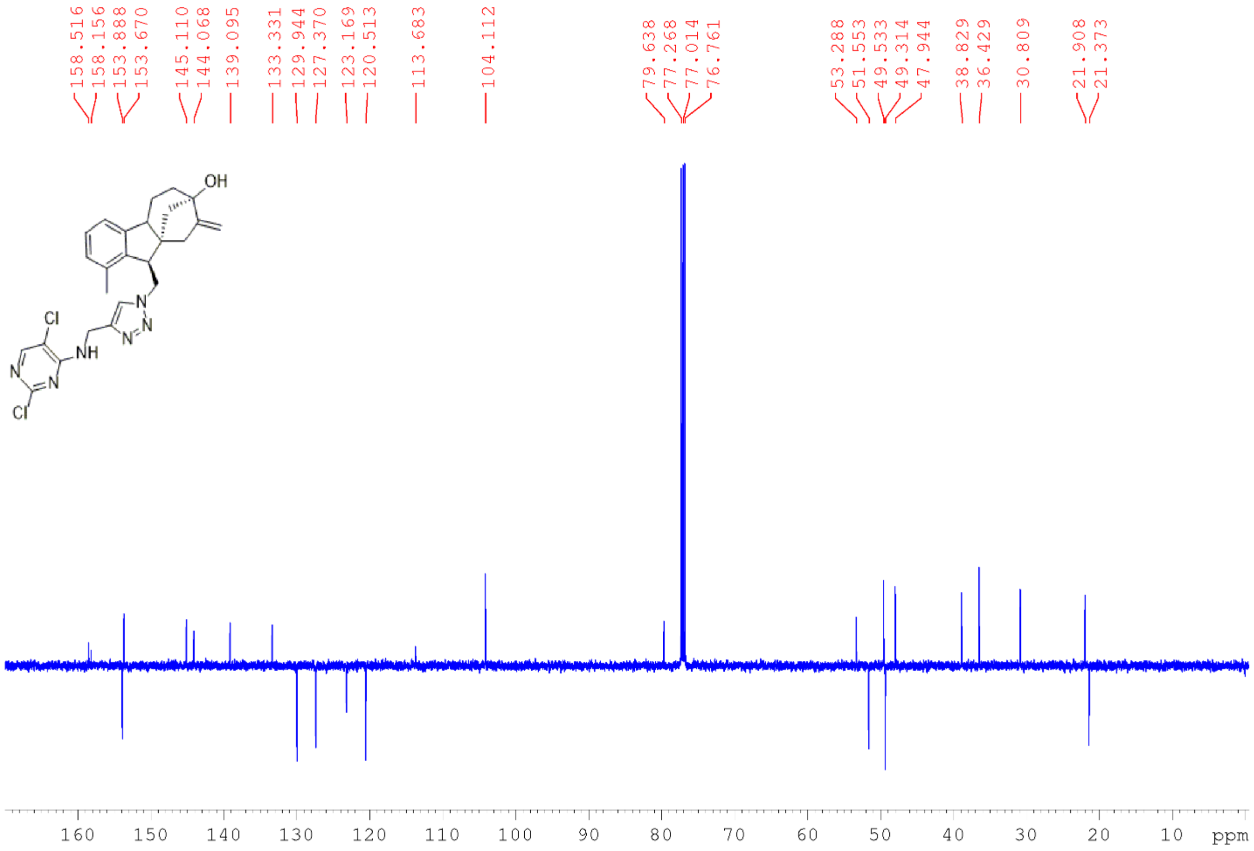

Figure S24. COSY-NMR of compound **18**

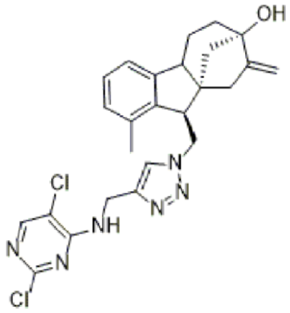

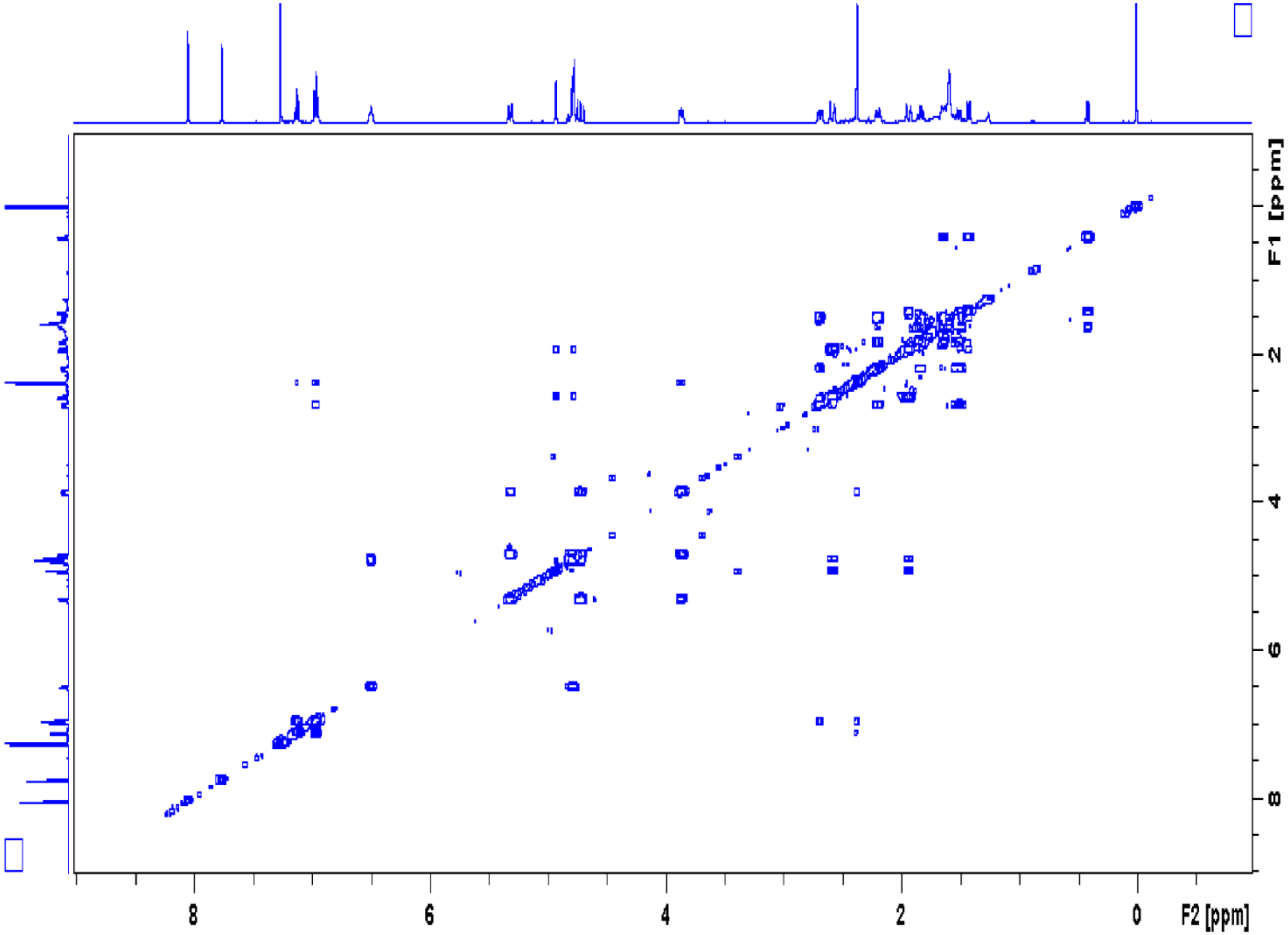

Figure S25. NOESY-NMR of compound **18**

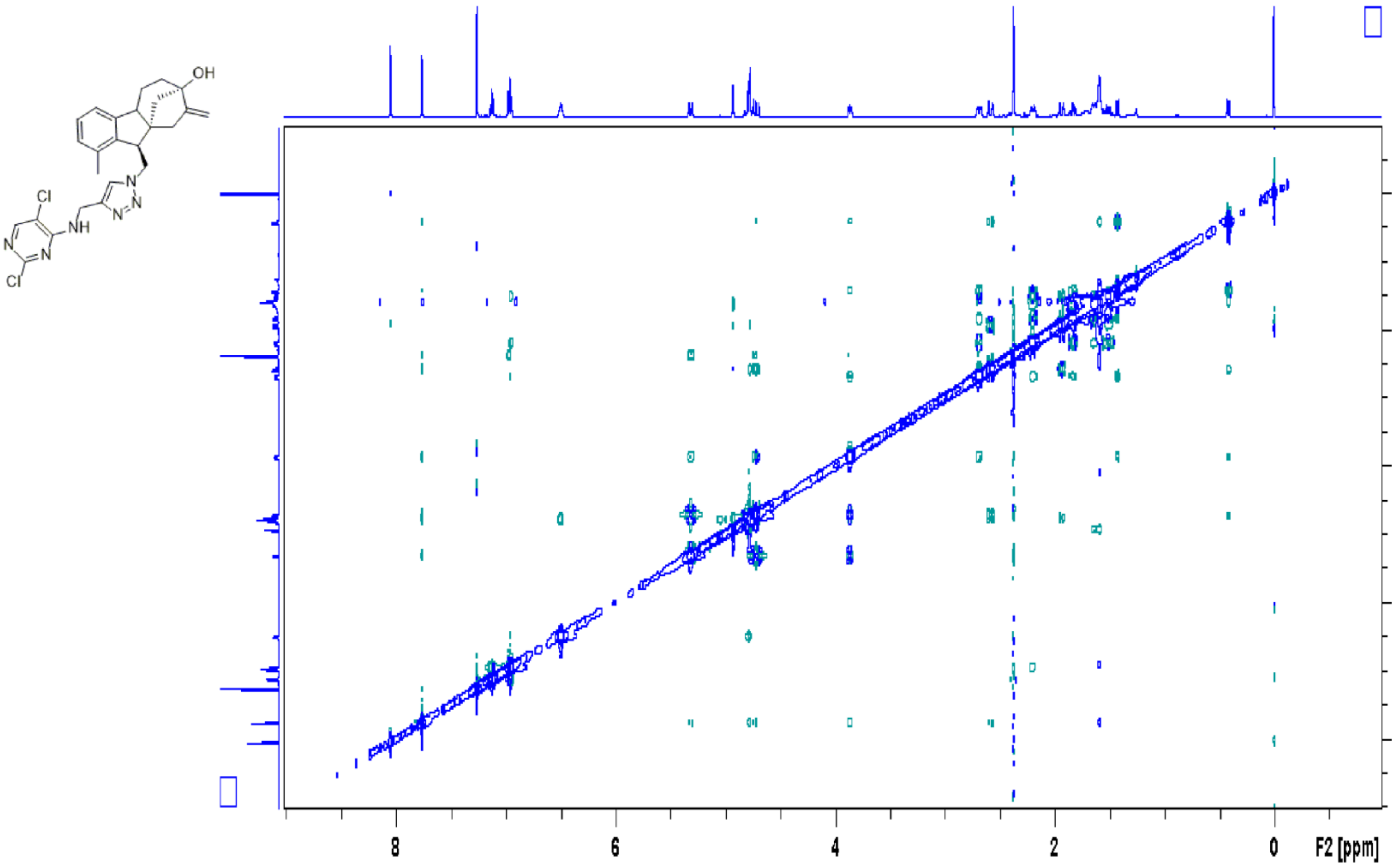

Figure S26. HSQC-NMR of compound **18**

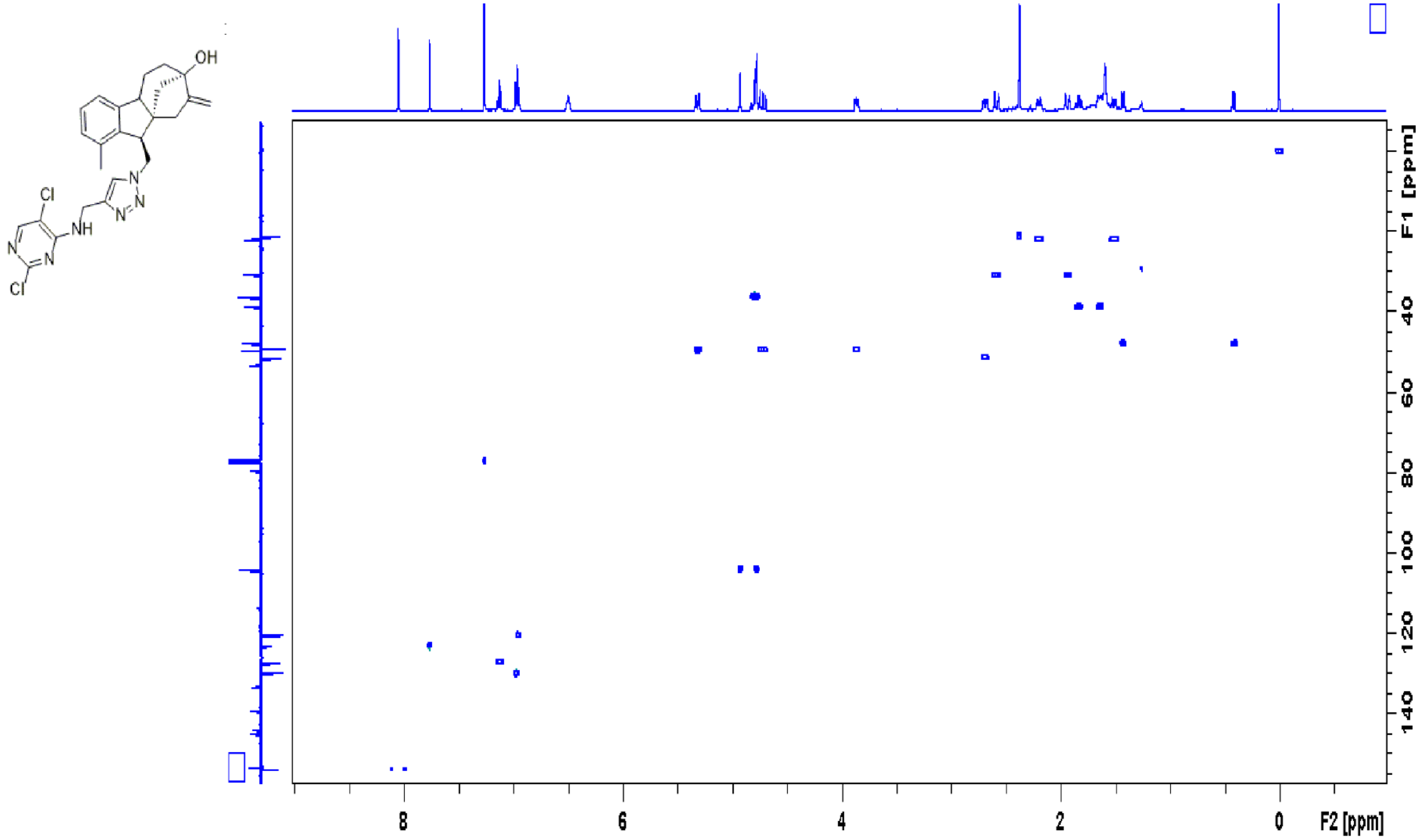

Figure S27. HMBC-NMR of compound **18**

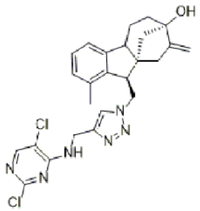

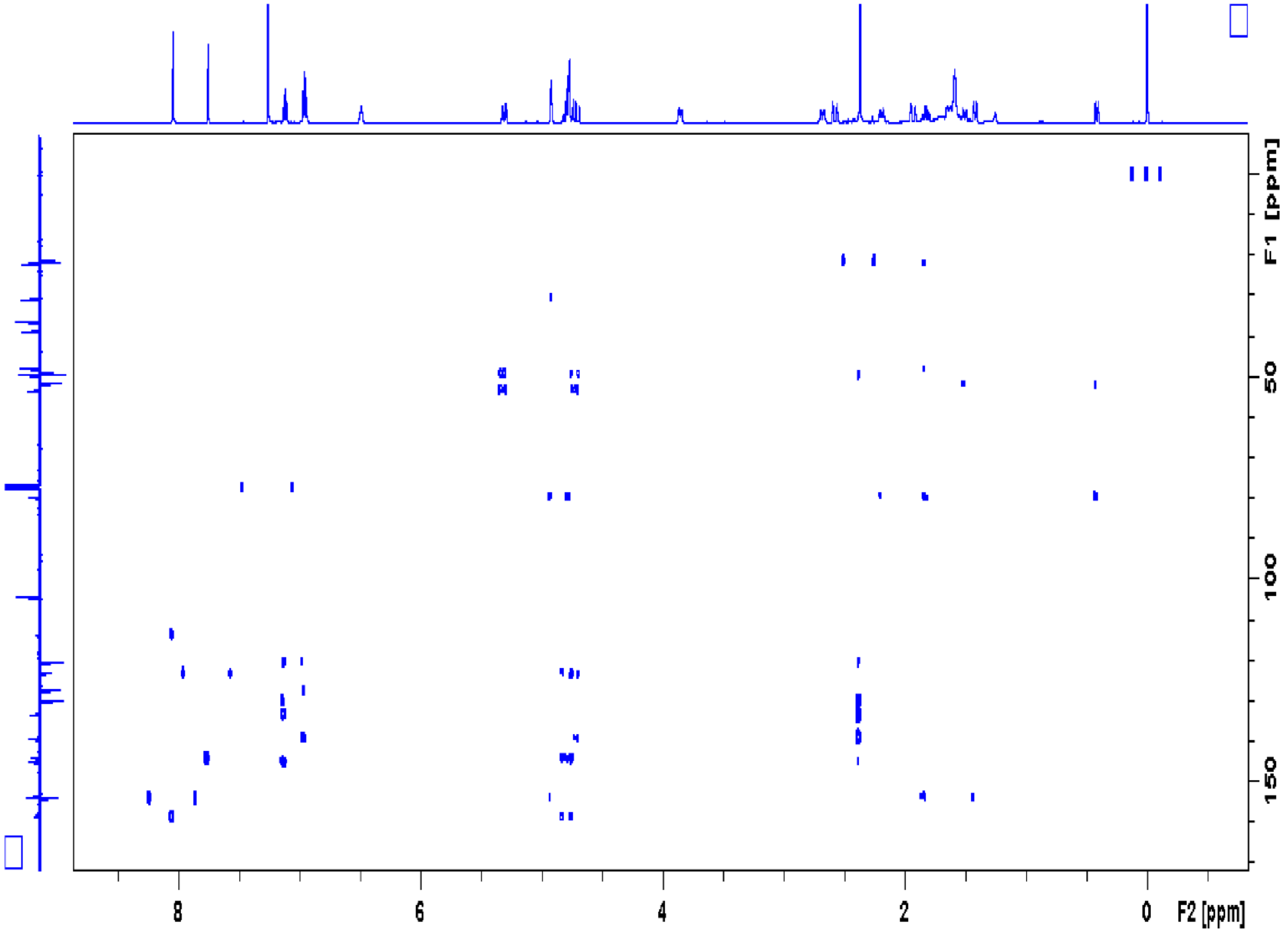

(7S,9aS,10R)-10-((4-(((5-Fluoro-2-((4-(trifluoromethyl)phenyl)amino)pyrimidin-4-yl)amino)me-thyl)-1H-1,2,3-triazol-1-yl)methyl)-1-methyl-8-methylene-4b,5,6,8,9,10-hexahydro-7H-7,9a-methanobenzo[a]azulen-7-ol (**19**)

Figure S28. ^{1}H-NMR of compound **19**

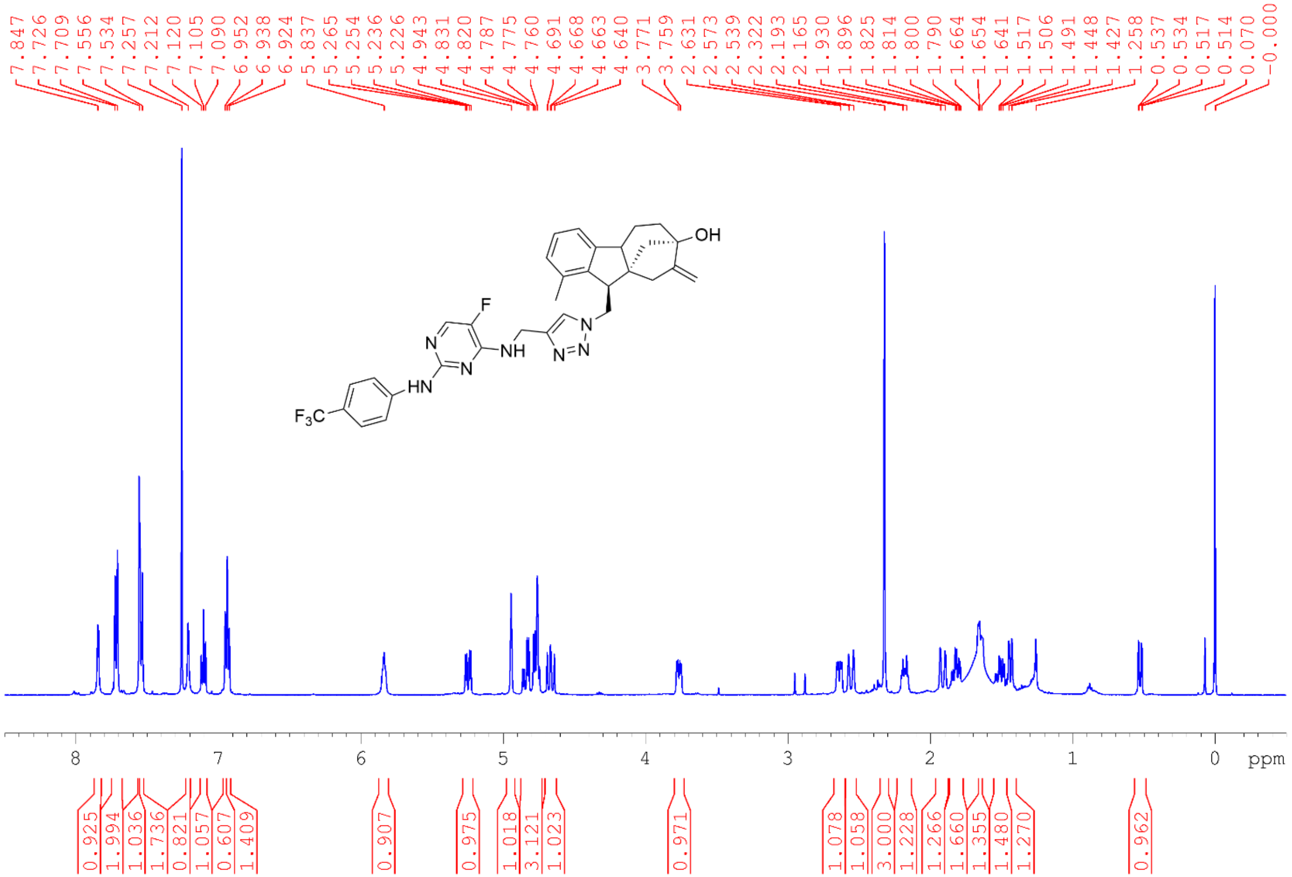

Figure S29. ^{13}C-NMR of compound **19**

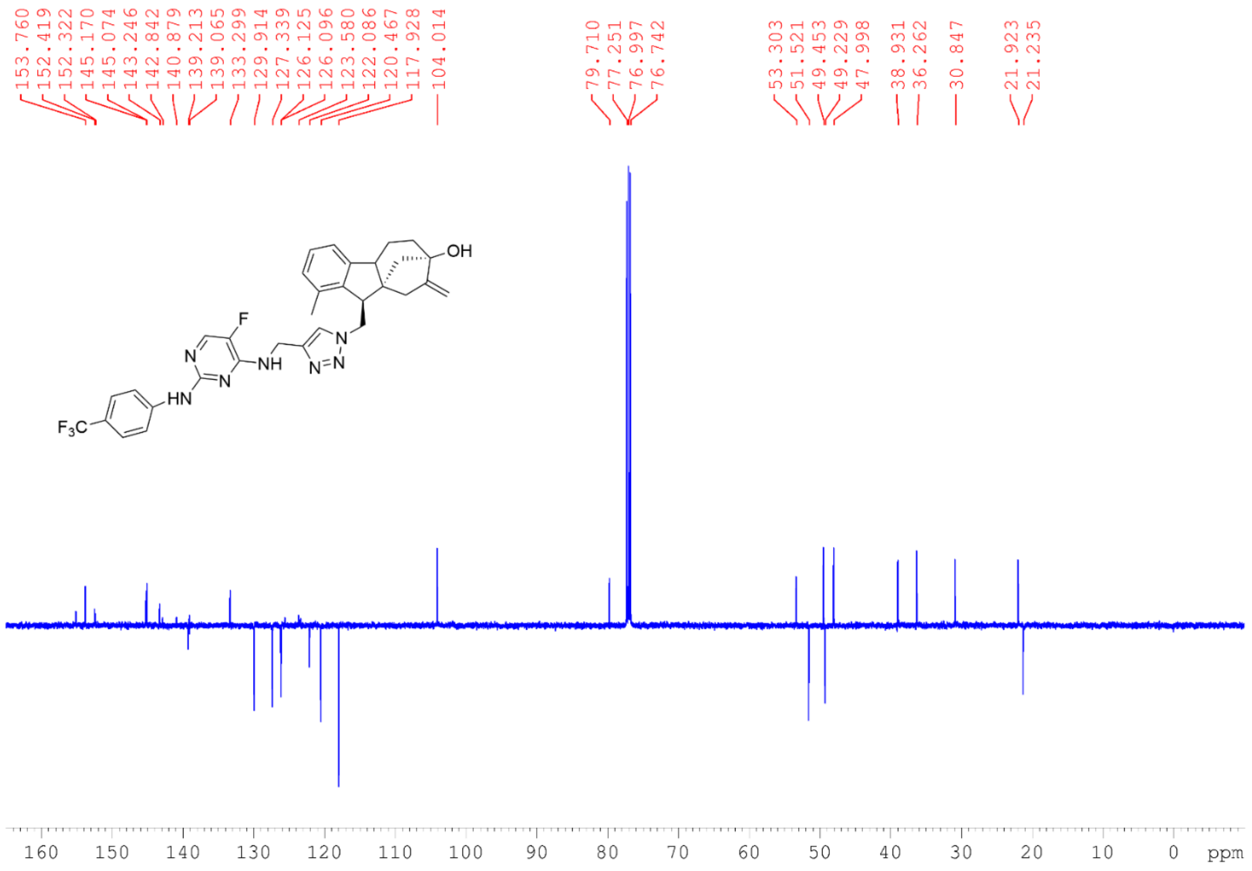

Figure S30. ^{19}F-NMR of compound **19**

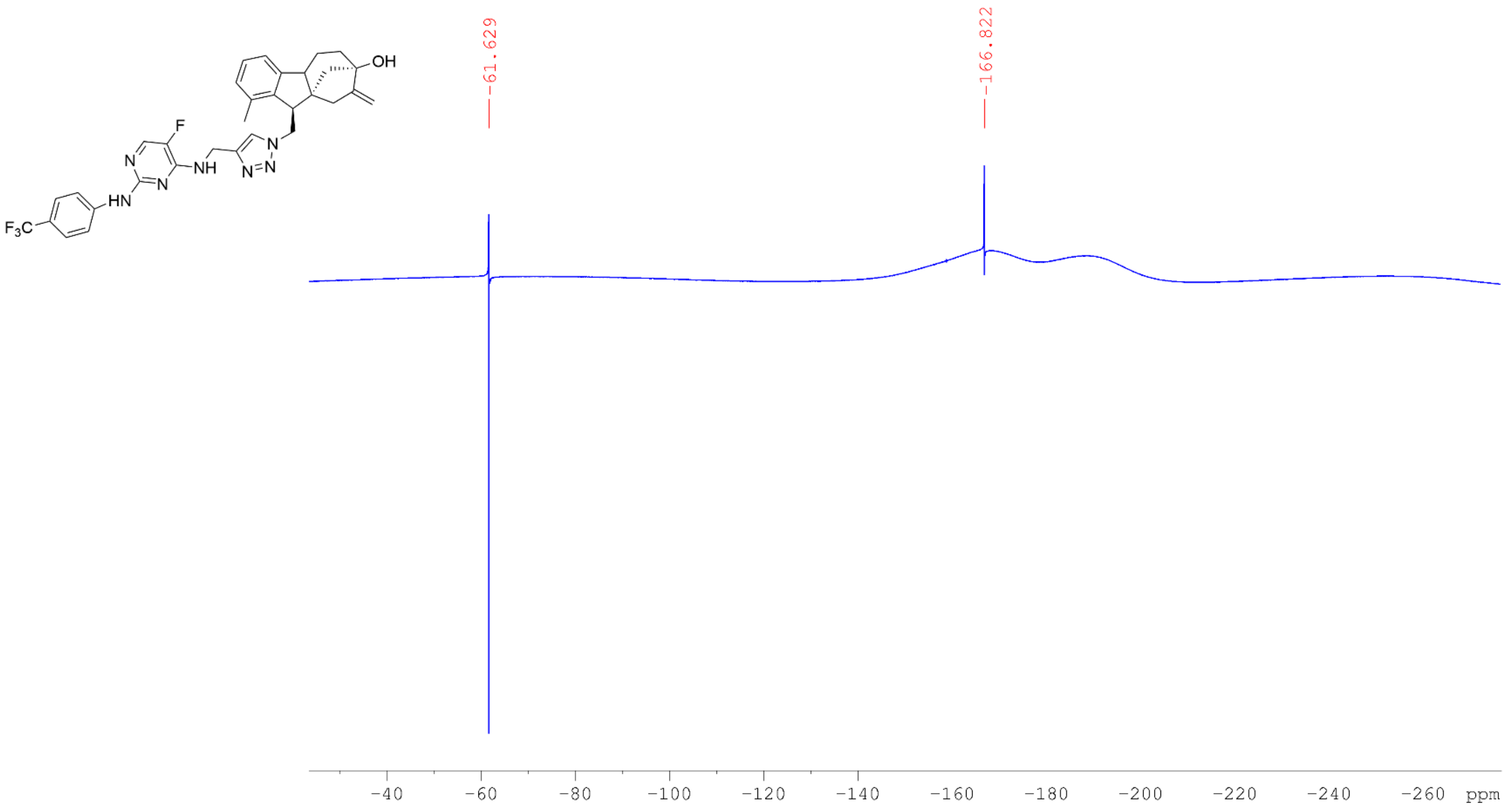

Figure S31. COSY-NMR of compound **19**

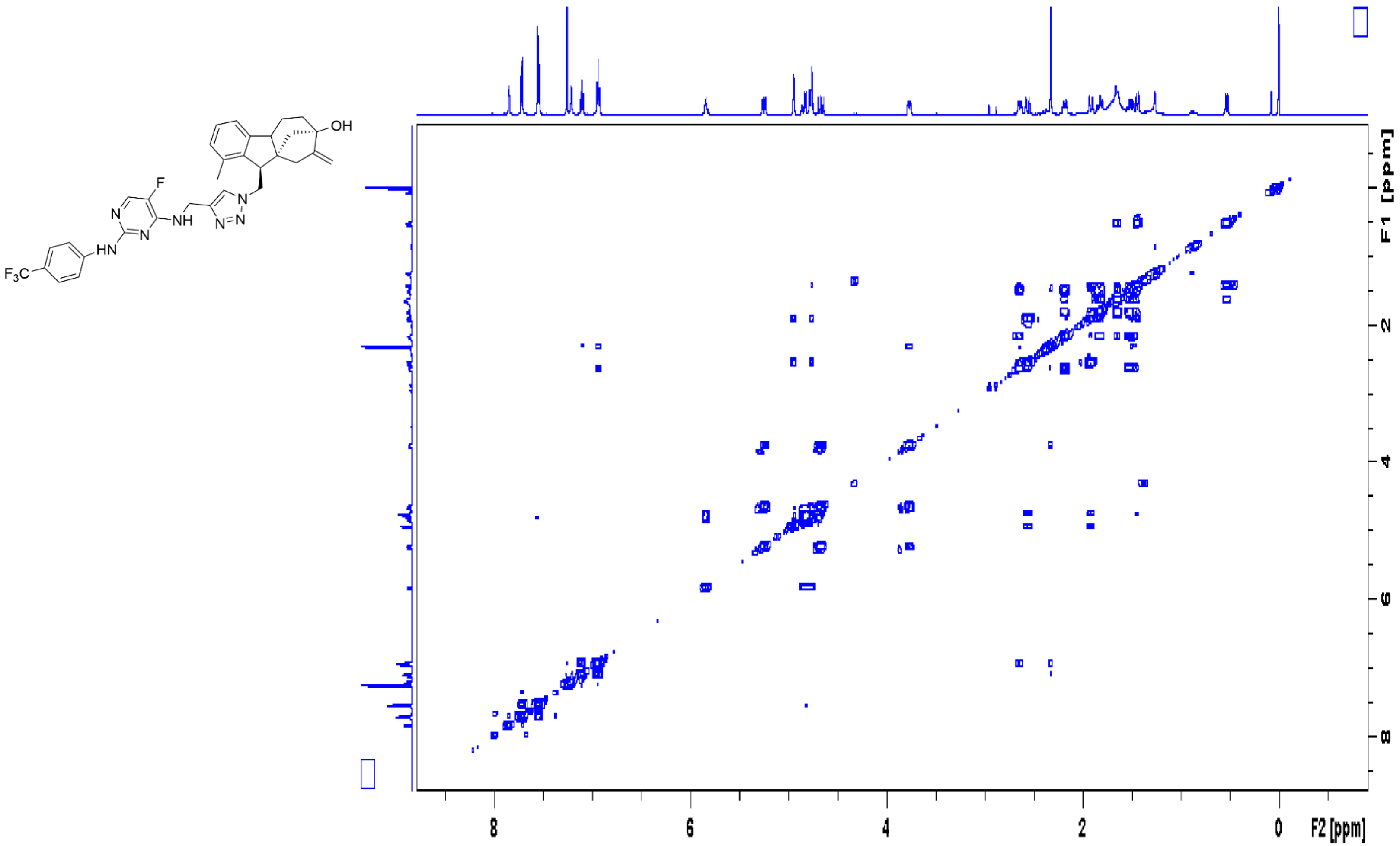

Figure S32. NOESY-NMR of compound **19**

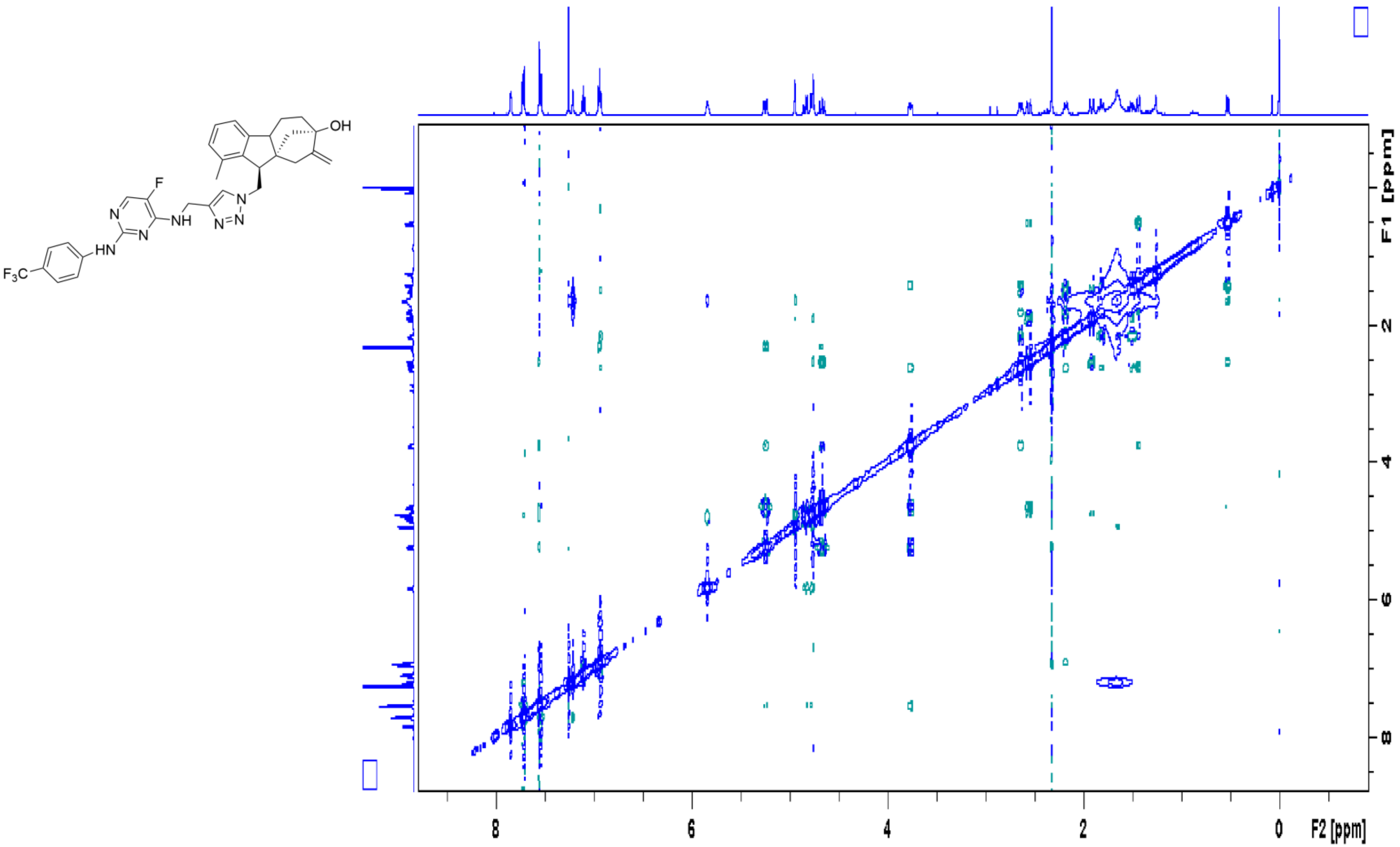

Figure S33. HSQC-NMR of compound **19**

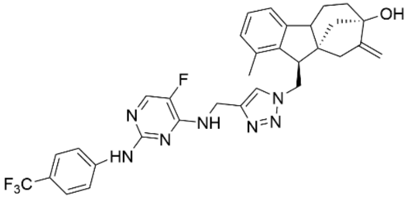

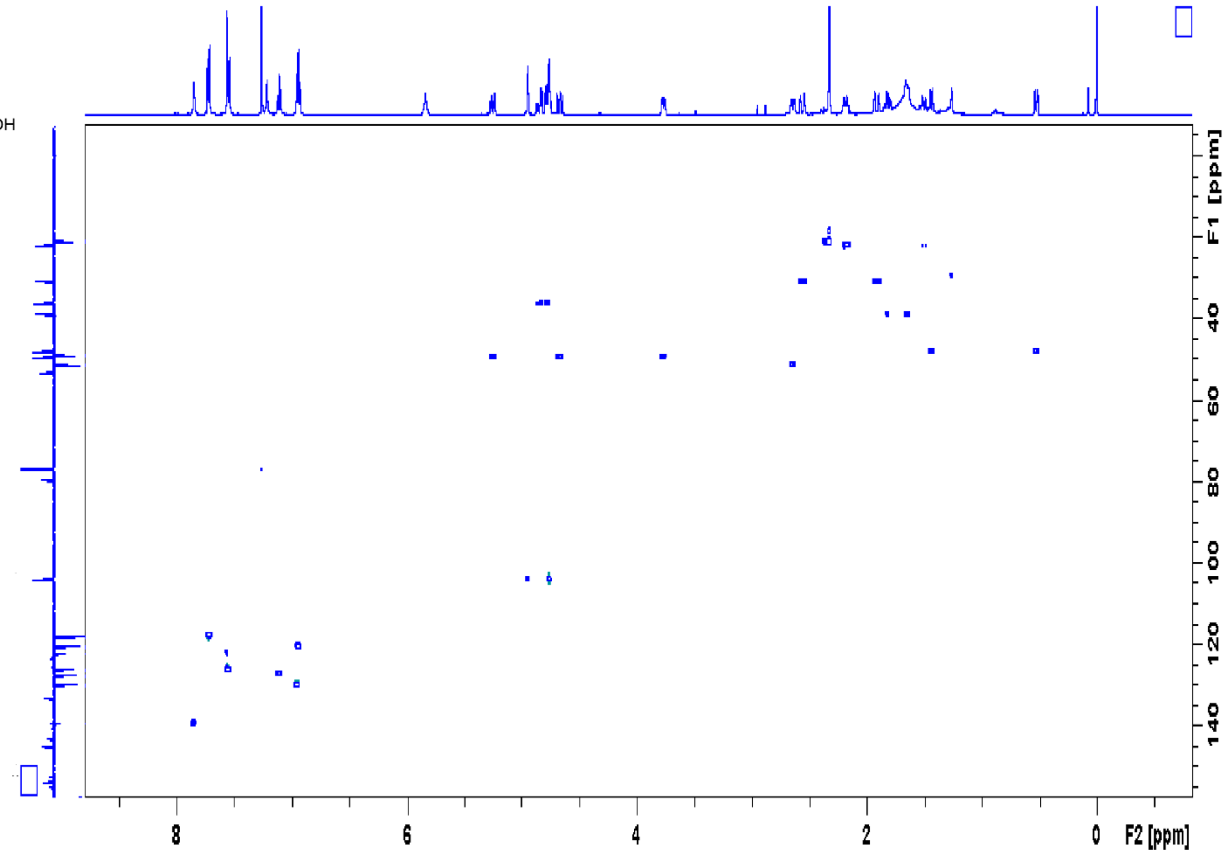

Figure S34. HMBC-NMR of compound **19**

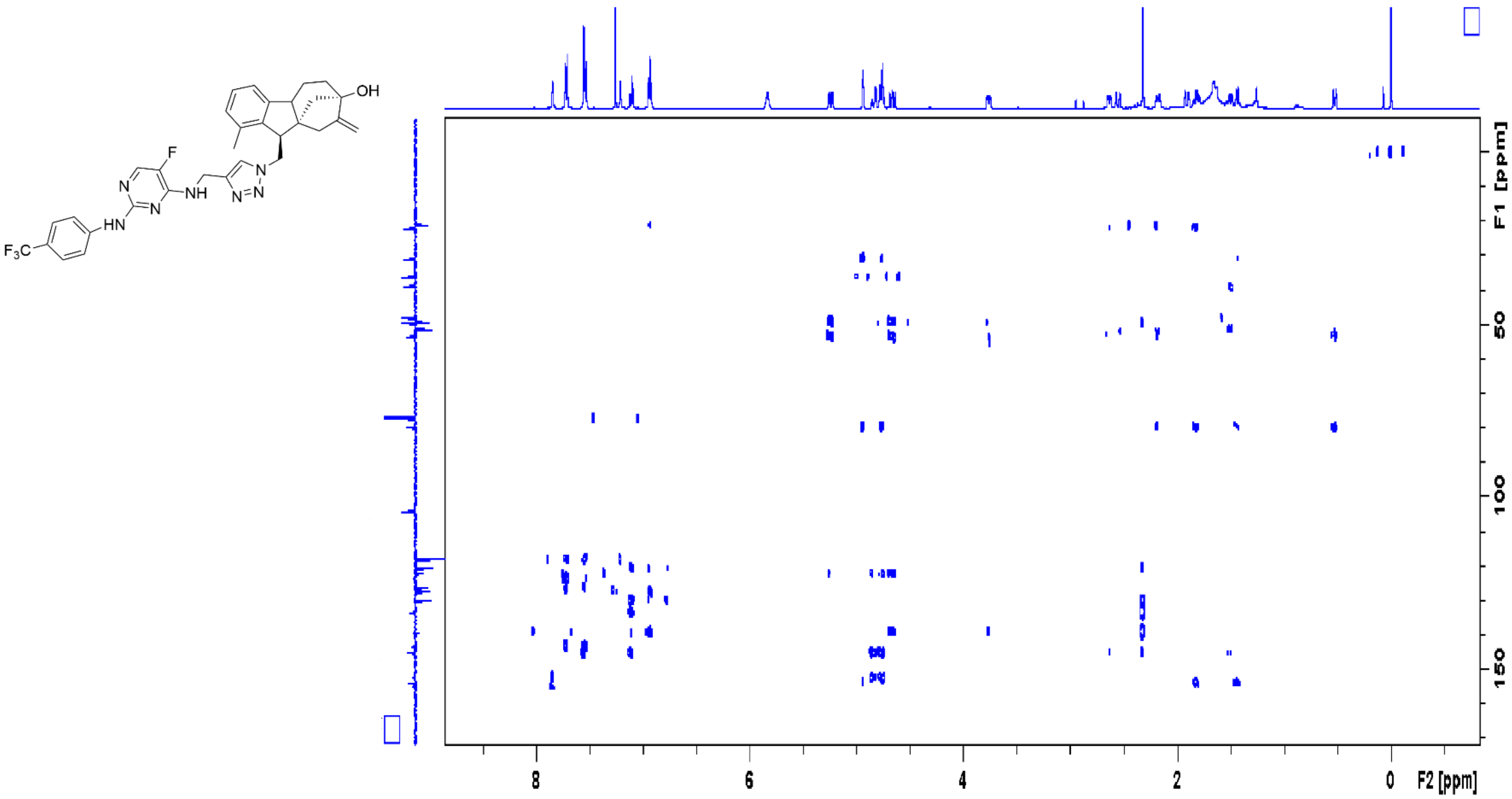

(7S,9aS,10R)-10-((4-(((5-Chloro-2-((4-(trifluoromethyl)phenyl)amino)pyrimidin-4-yl)amino)me-thyl)-1H-1,2,3-triazol-1-yl)methyl)-1-methyl-8-methylene-4b,5,6,8,9,10-hexahydro-7H-7,9a-methanobenzo[a]azulen-7-ol (**20**)

Figure S35. ^{1}H-NMR of compound **20**

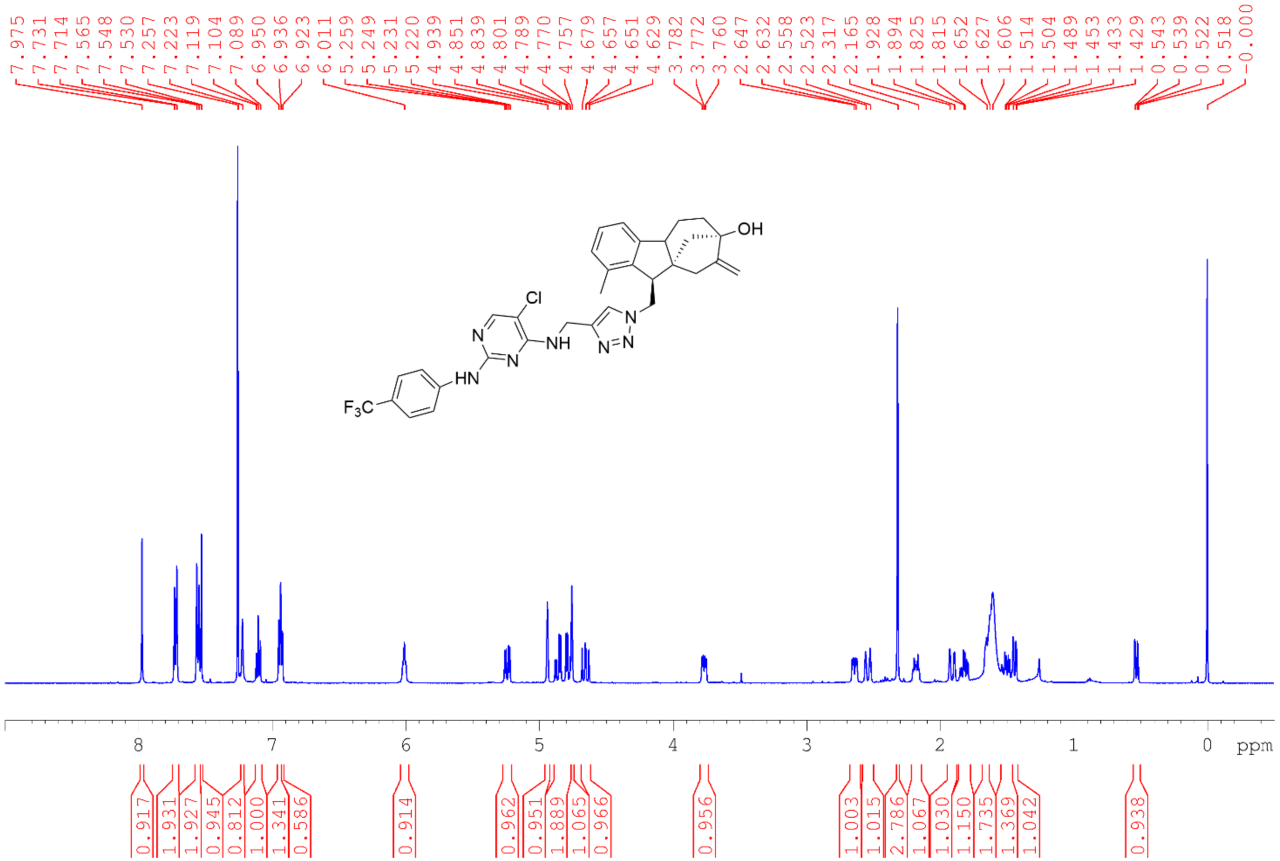

Figure S36. ^{13}C-NMR of compound **20**

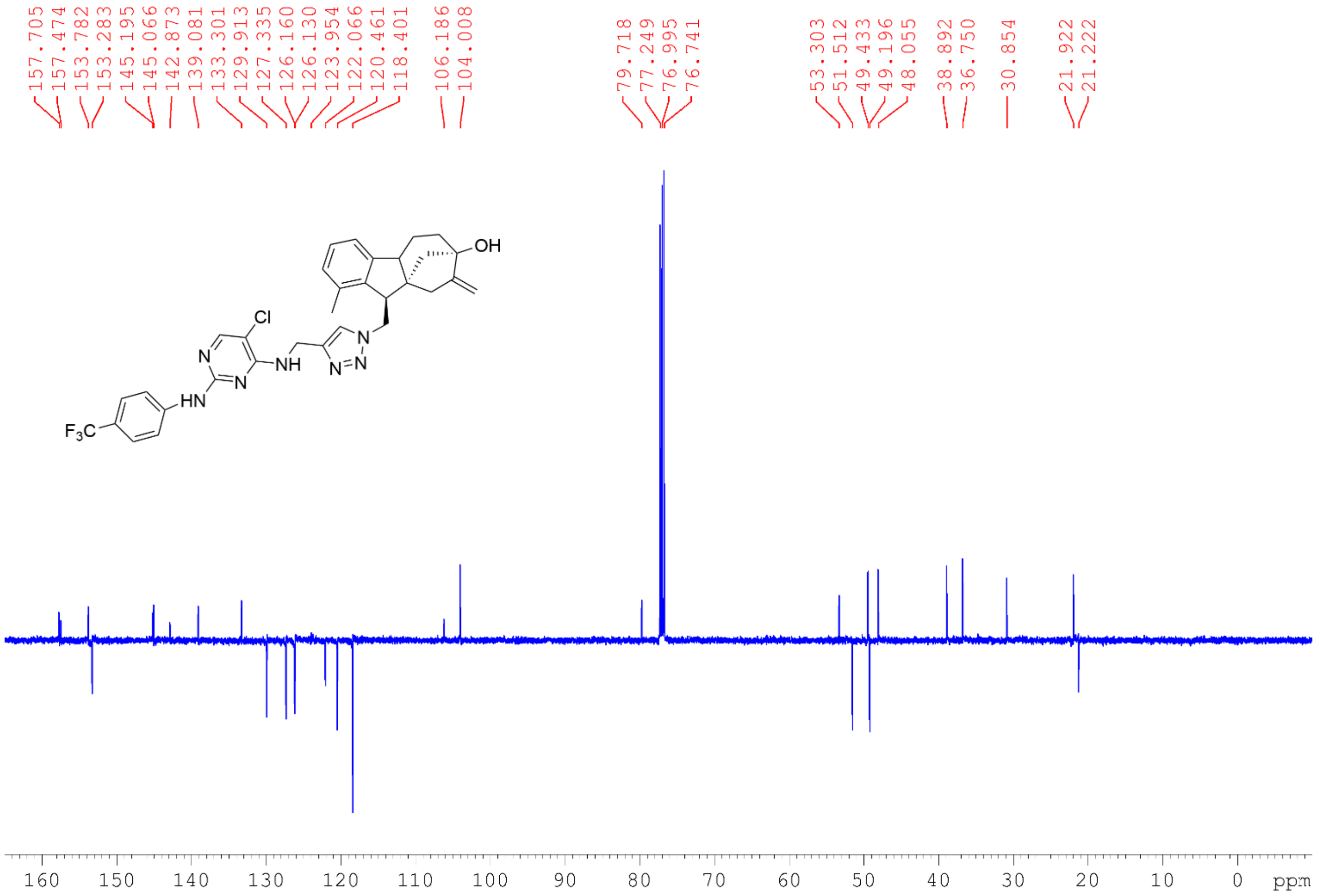

Figure S37. ^{19}F-NMR of compound **20**

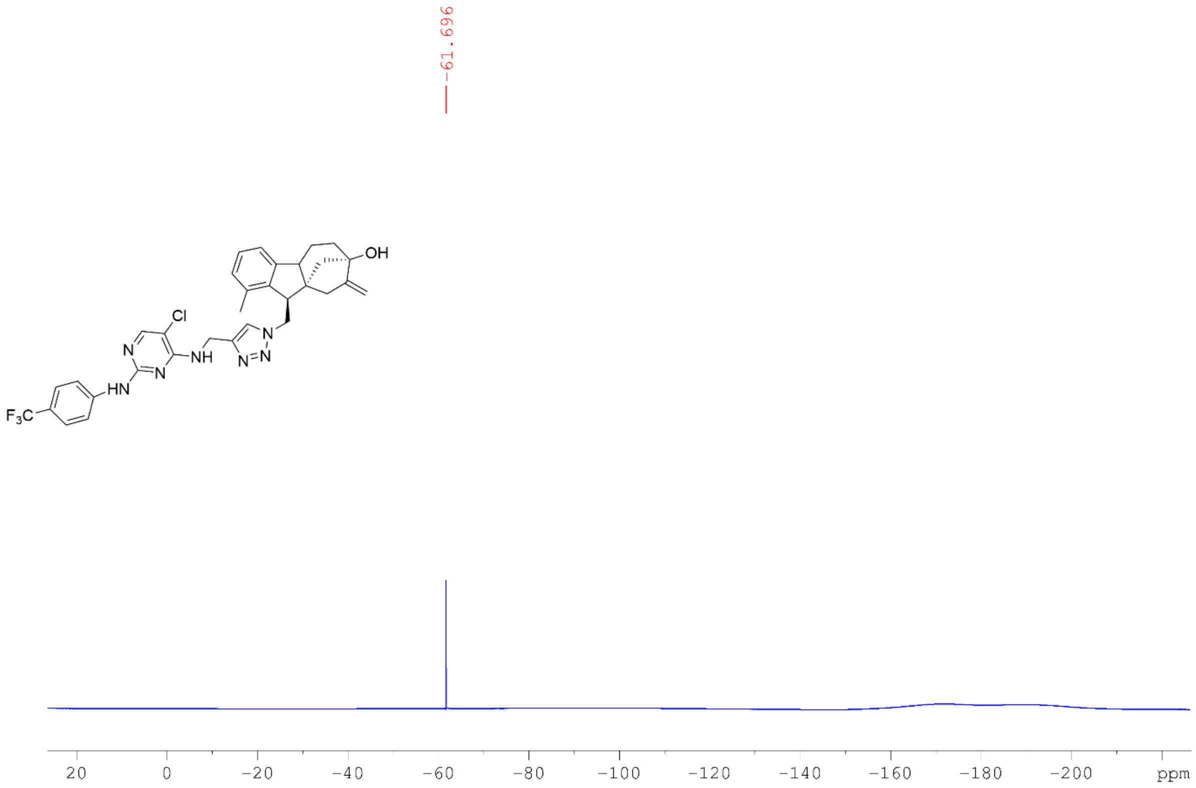

Figure S38. COSY-NMR of compound **20**

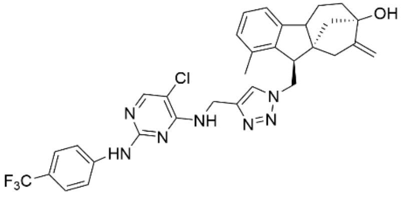

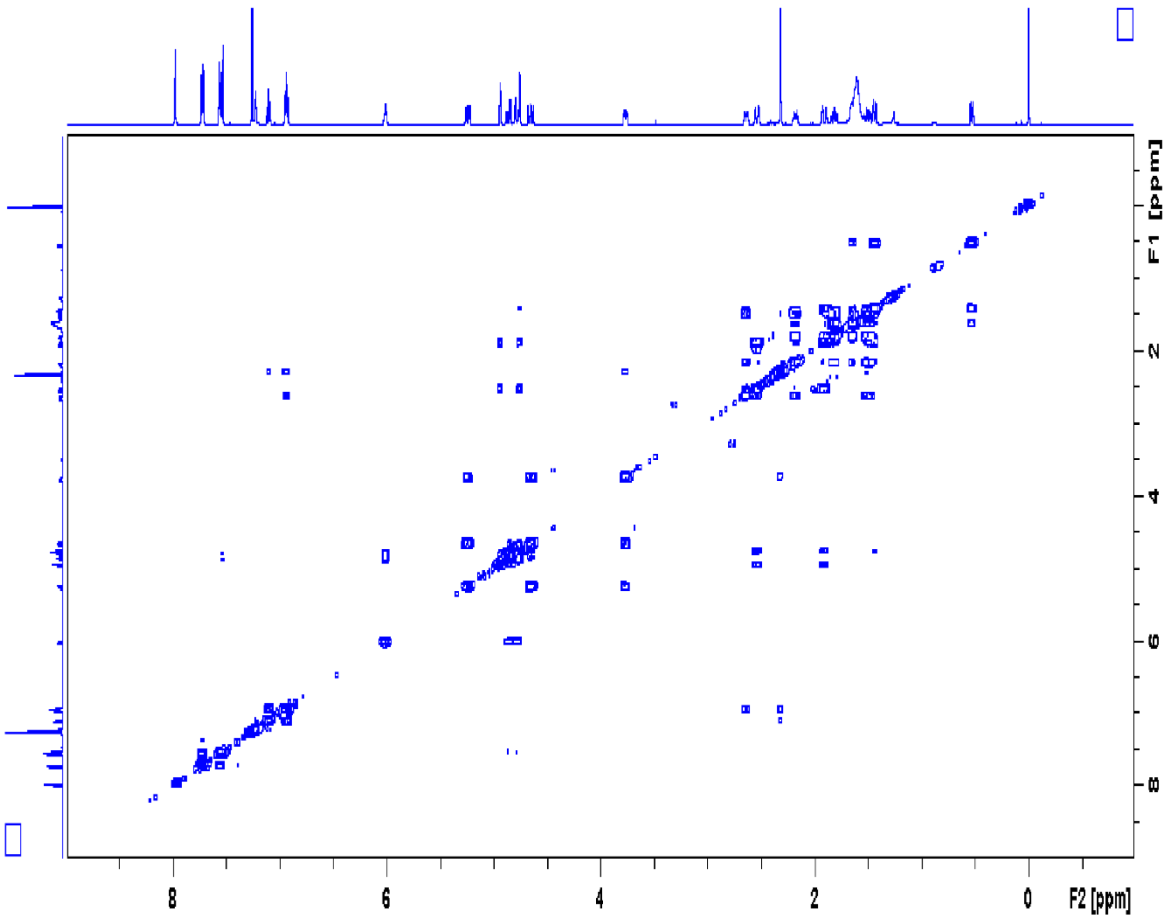

Figure S39. NOESY-NMR of compound **20**

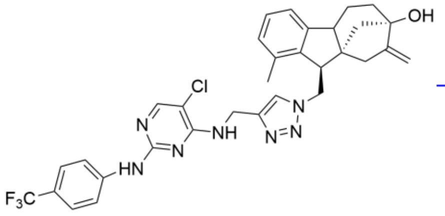

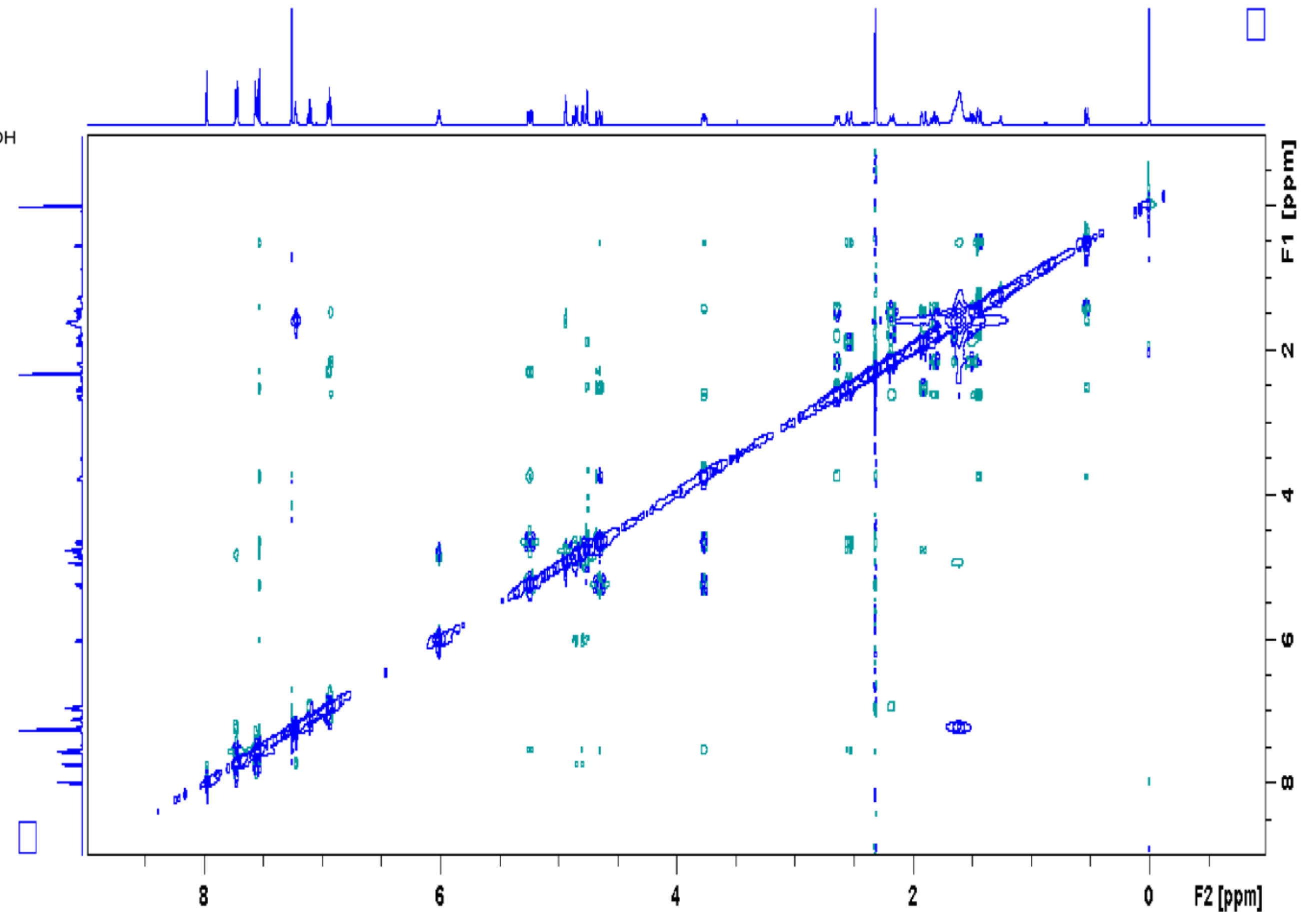

Figure S40. HSQC-NMR of compound **20**

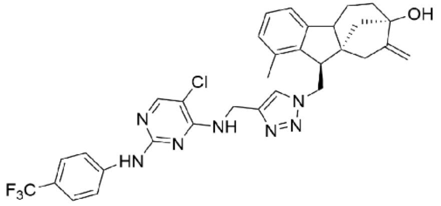

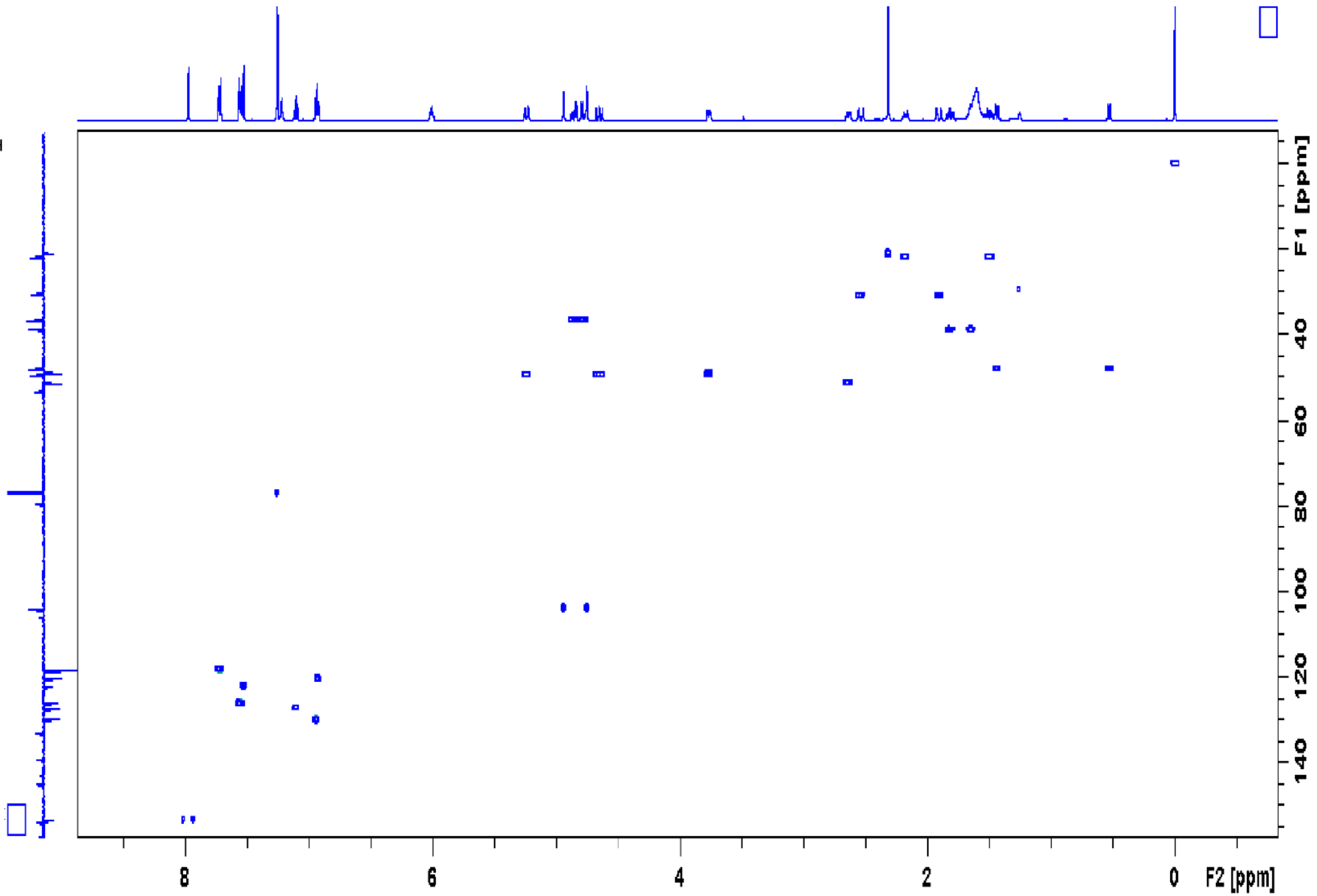

Figure S41. HMBC-NMR of compound **20**

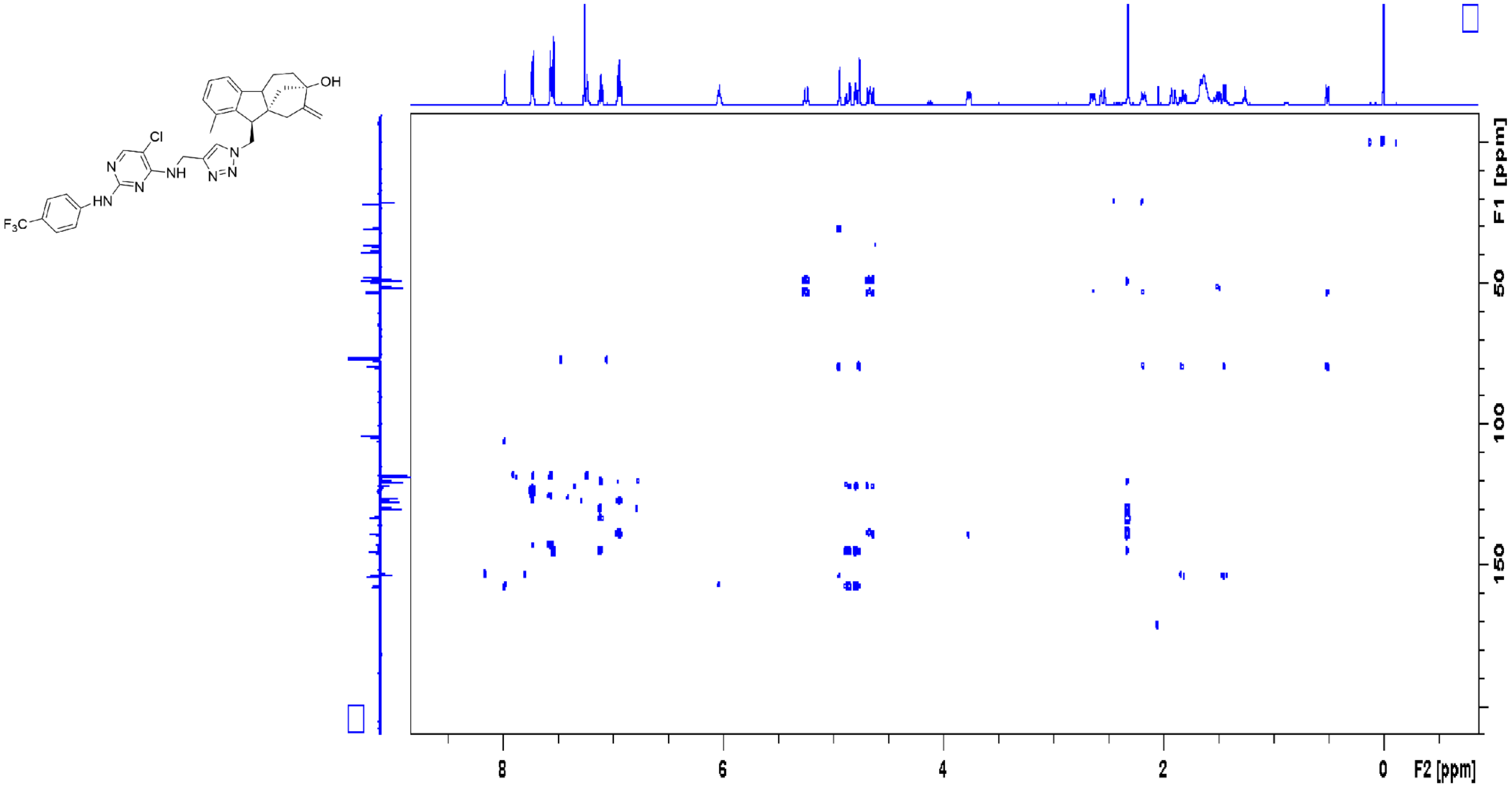

(7S,9aS,10R)-10-((4-(((5-Fluoro-2-((1-methyl-1H-pyrazol-4-yl)amino)pyrimidin-4-yl)amino)me-thyl)-1H-1,2,3-triazol-1-yl)methyl)-1-methyl-8-methylene-4b,5,6,8,9,10-hexahydro-7H-7,9a-methanobenzo[a]azulen-7-ol (**21**)

Figure S42. ^{1}H-NMR of compound **21**

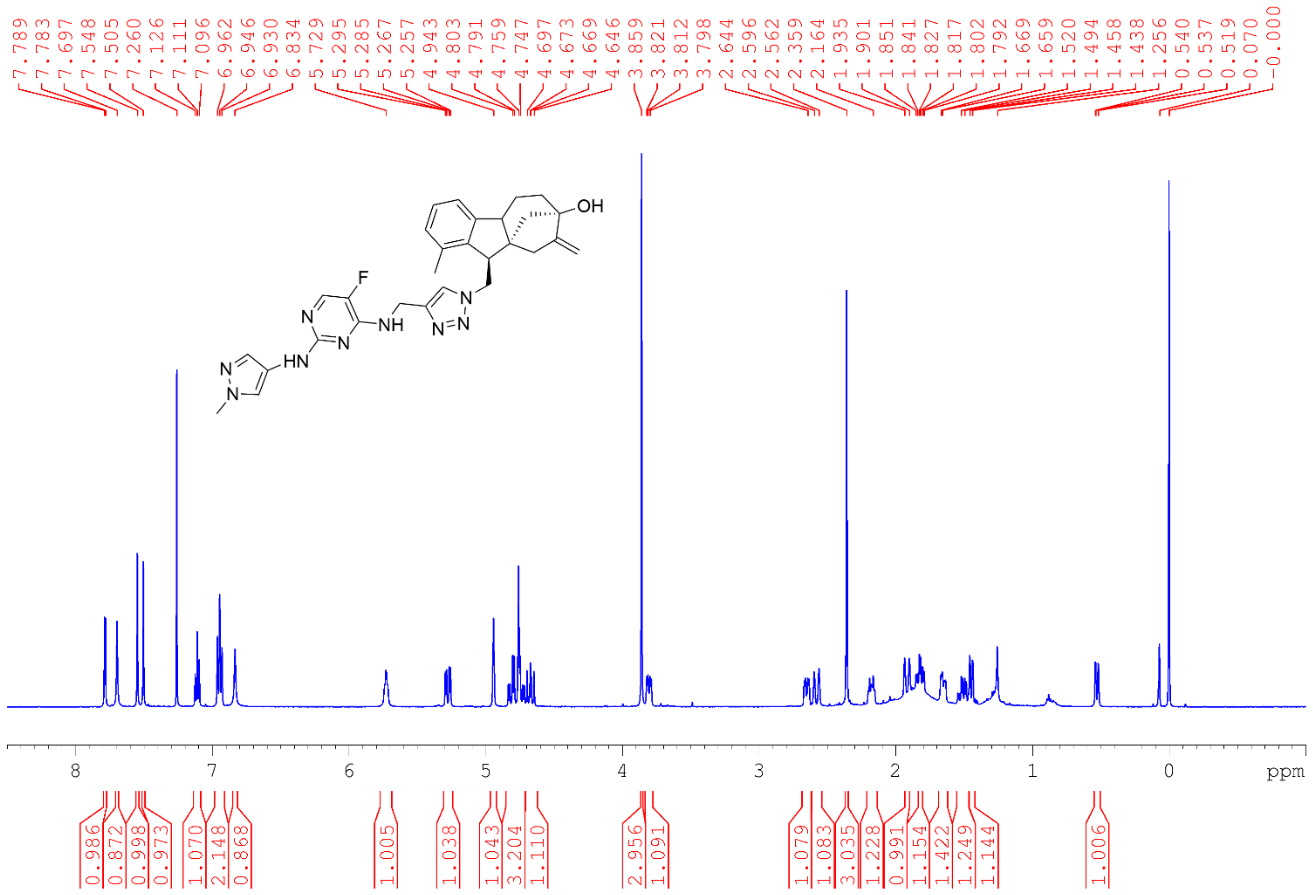

Figure S43. ^{13}C-NMR of compound **21**

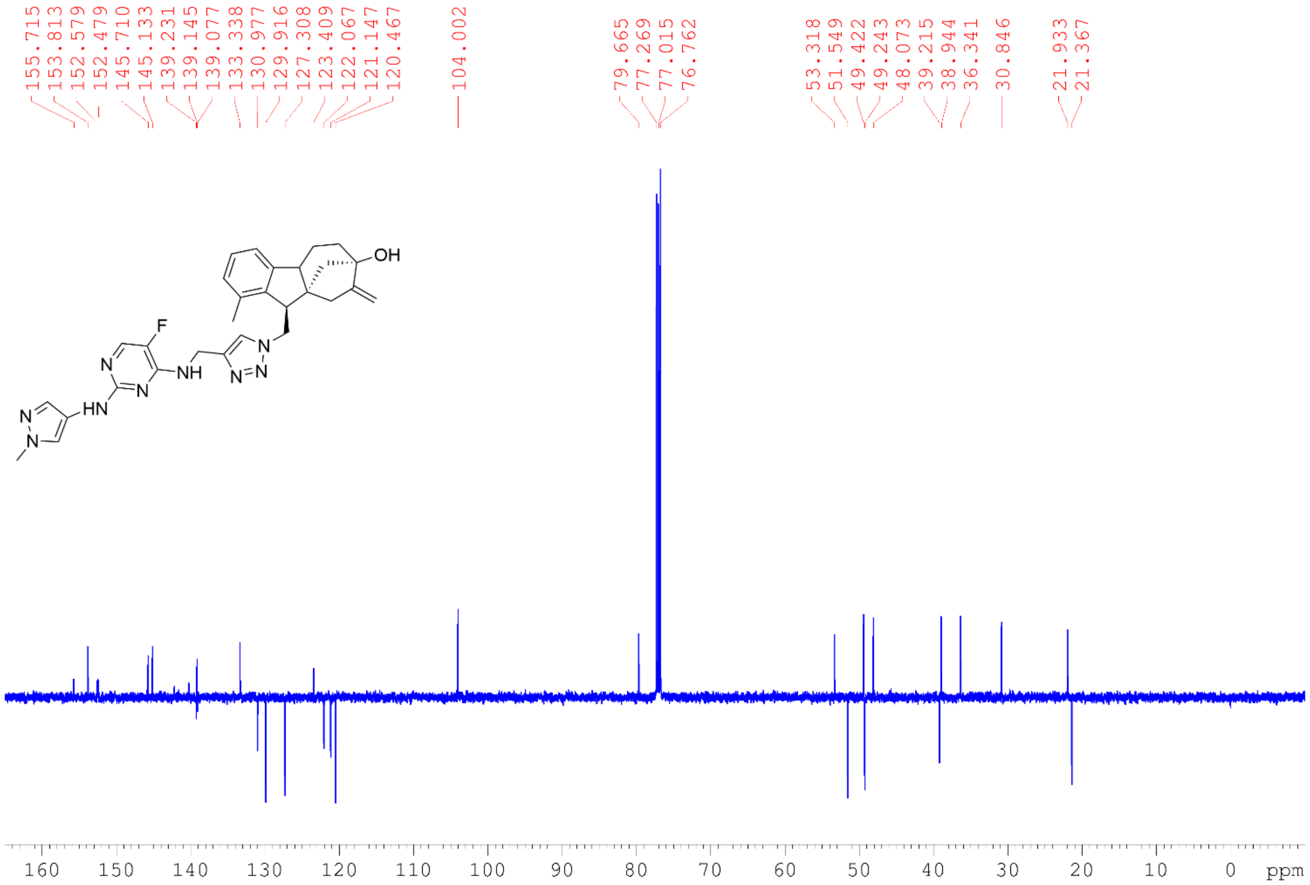

Figure S44. ^{19}F-NMR of compound **21**

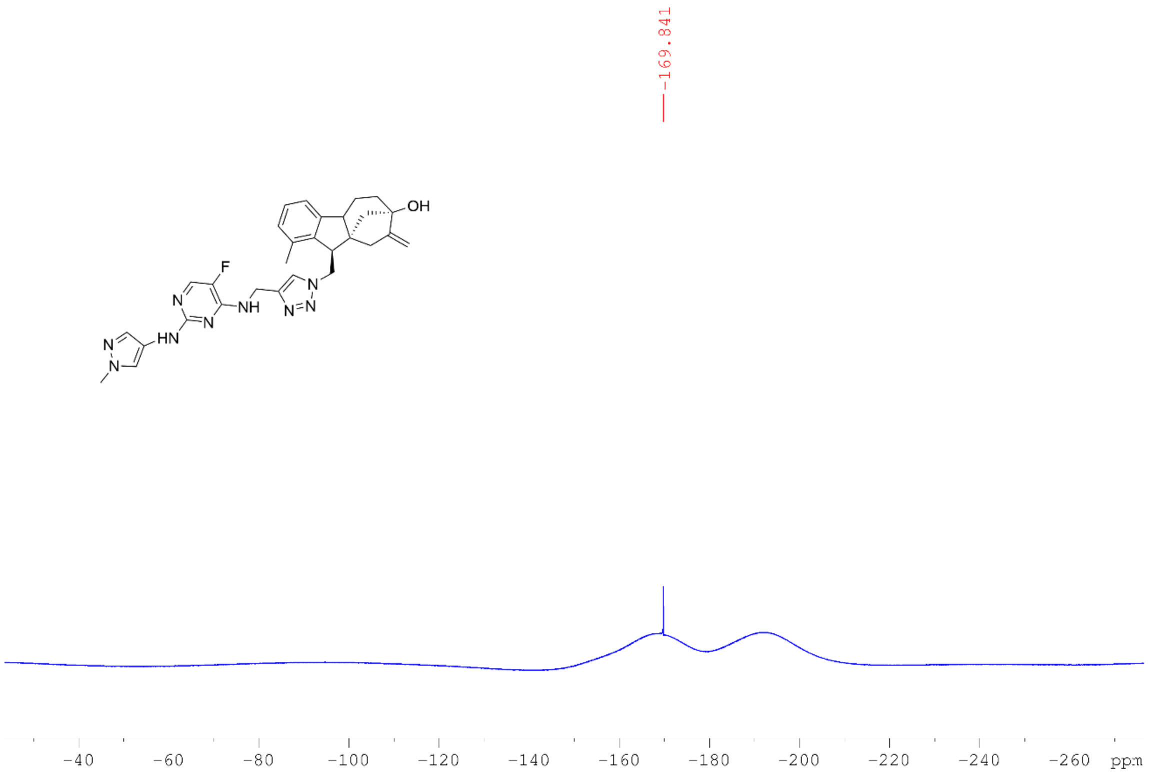

Figure S45. COSY-NMR of compound **21**

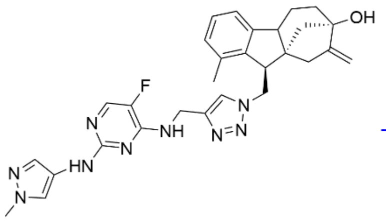

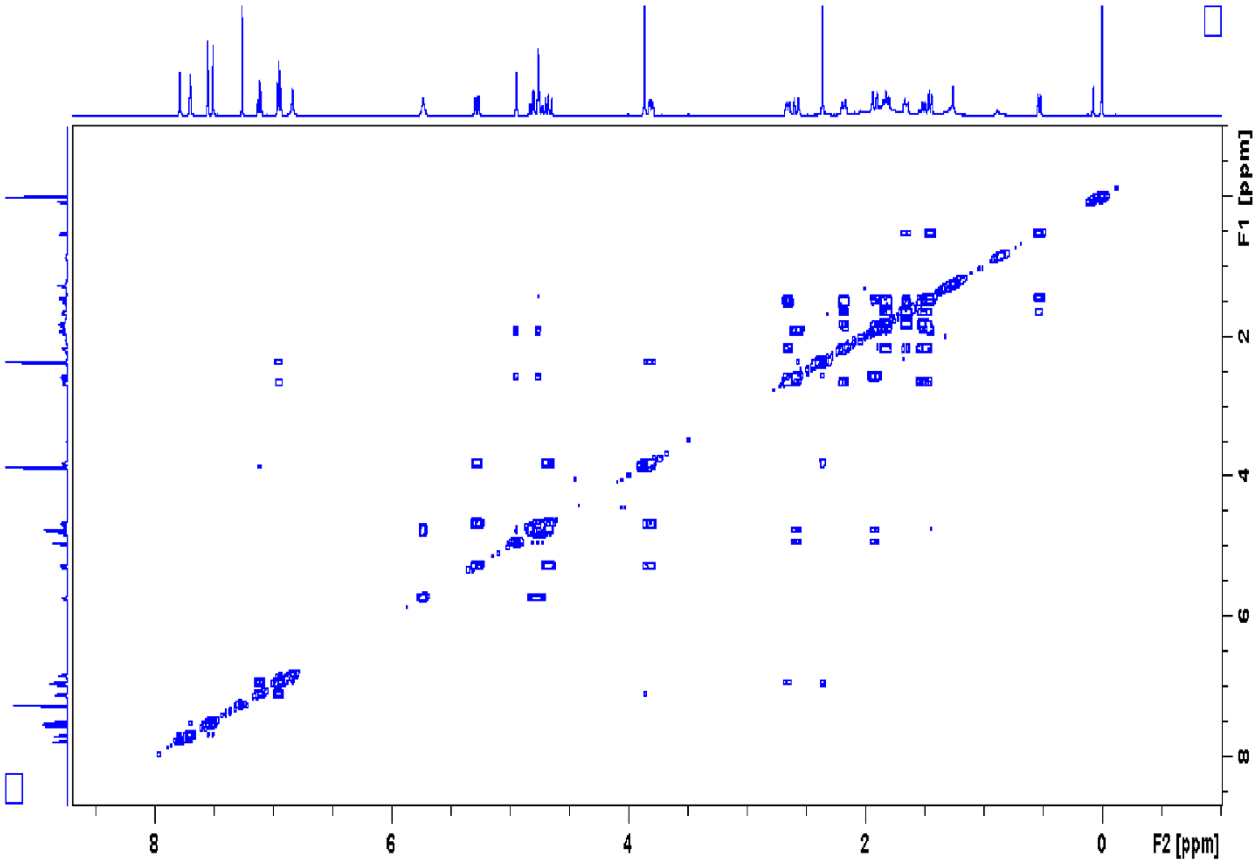

Figure S46. NOESY-NMR of compound **21**

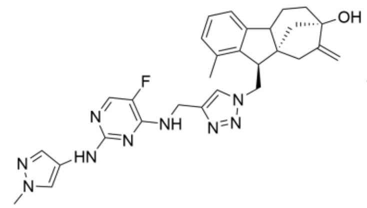

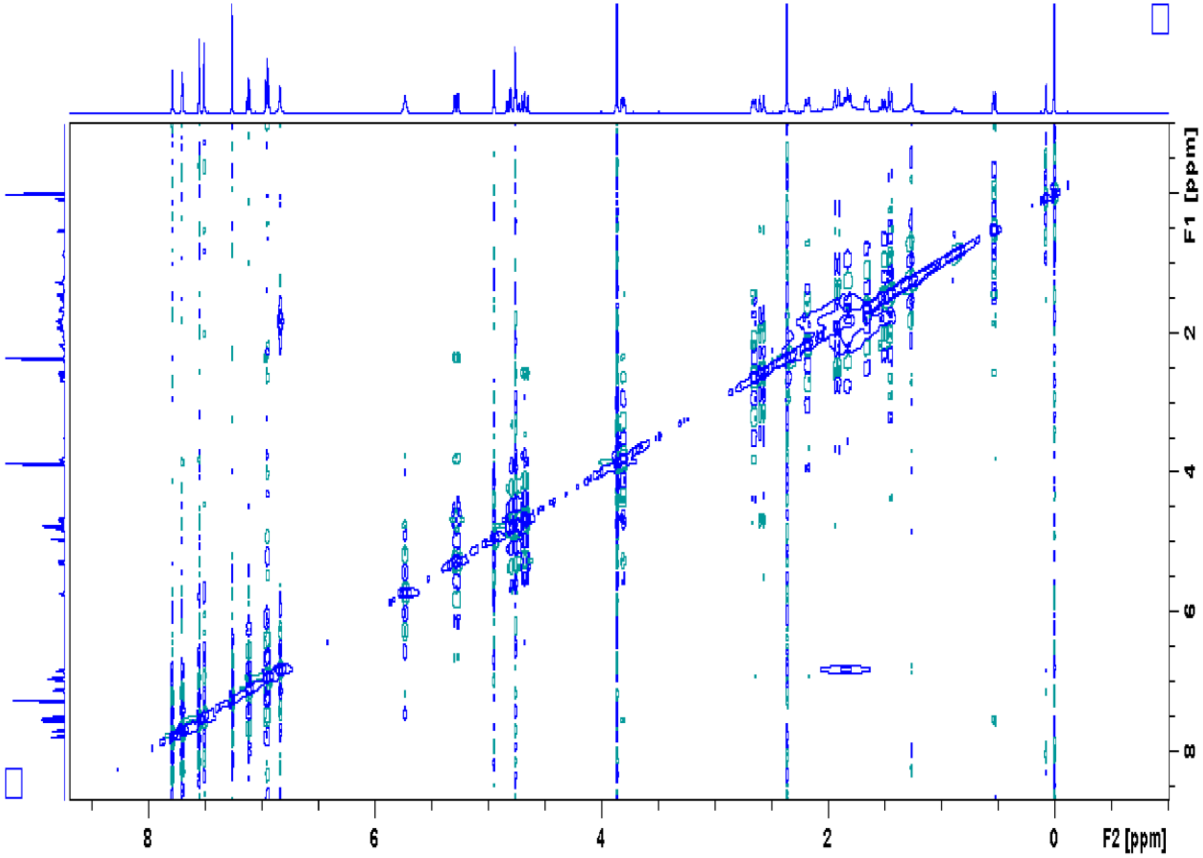

Figure S47. HSQC-NMR of compound **21**

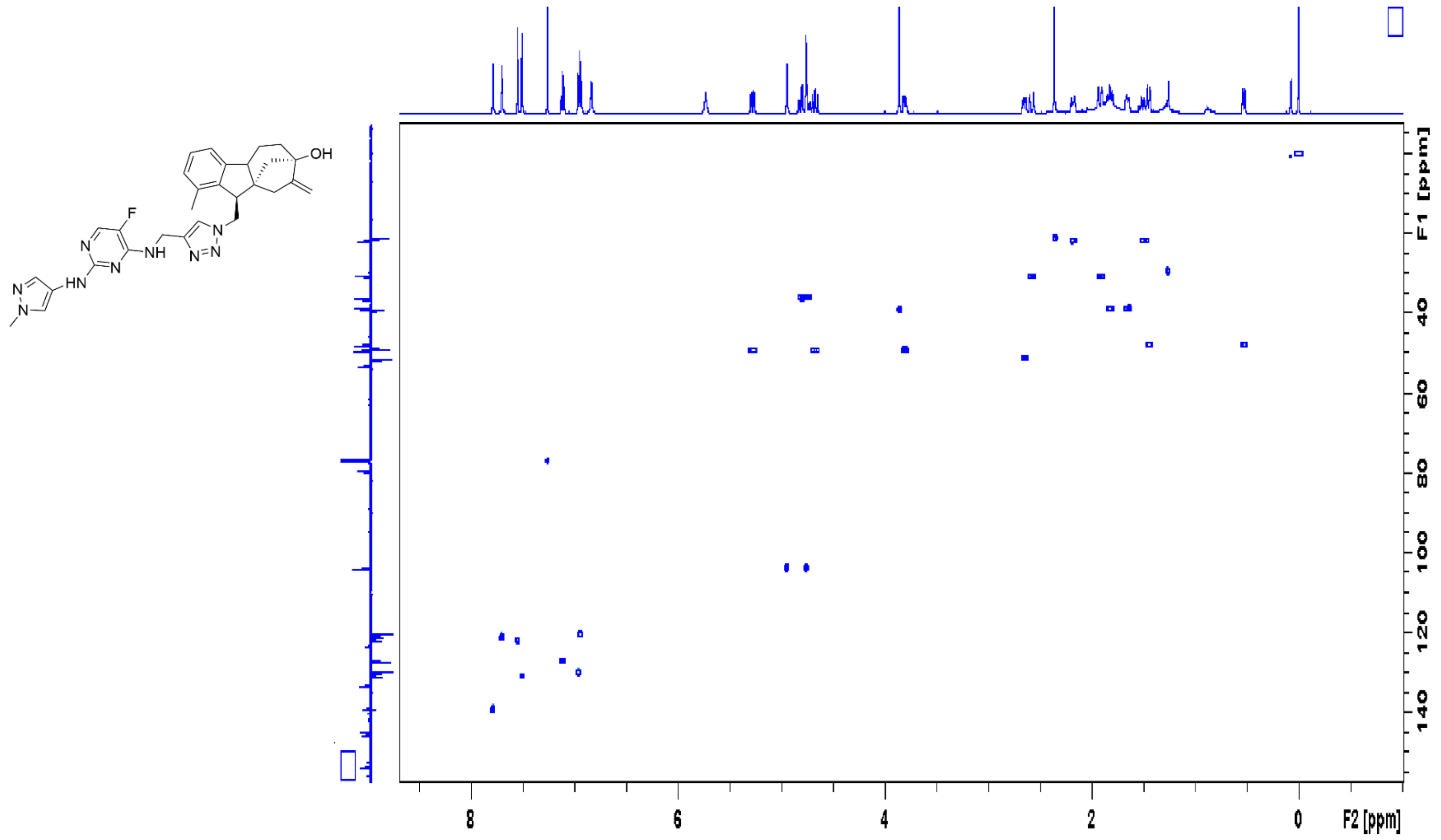

Figure S48. HMBC-NMR of compound **21**

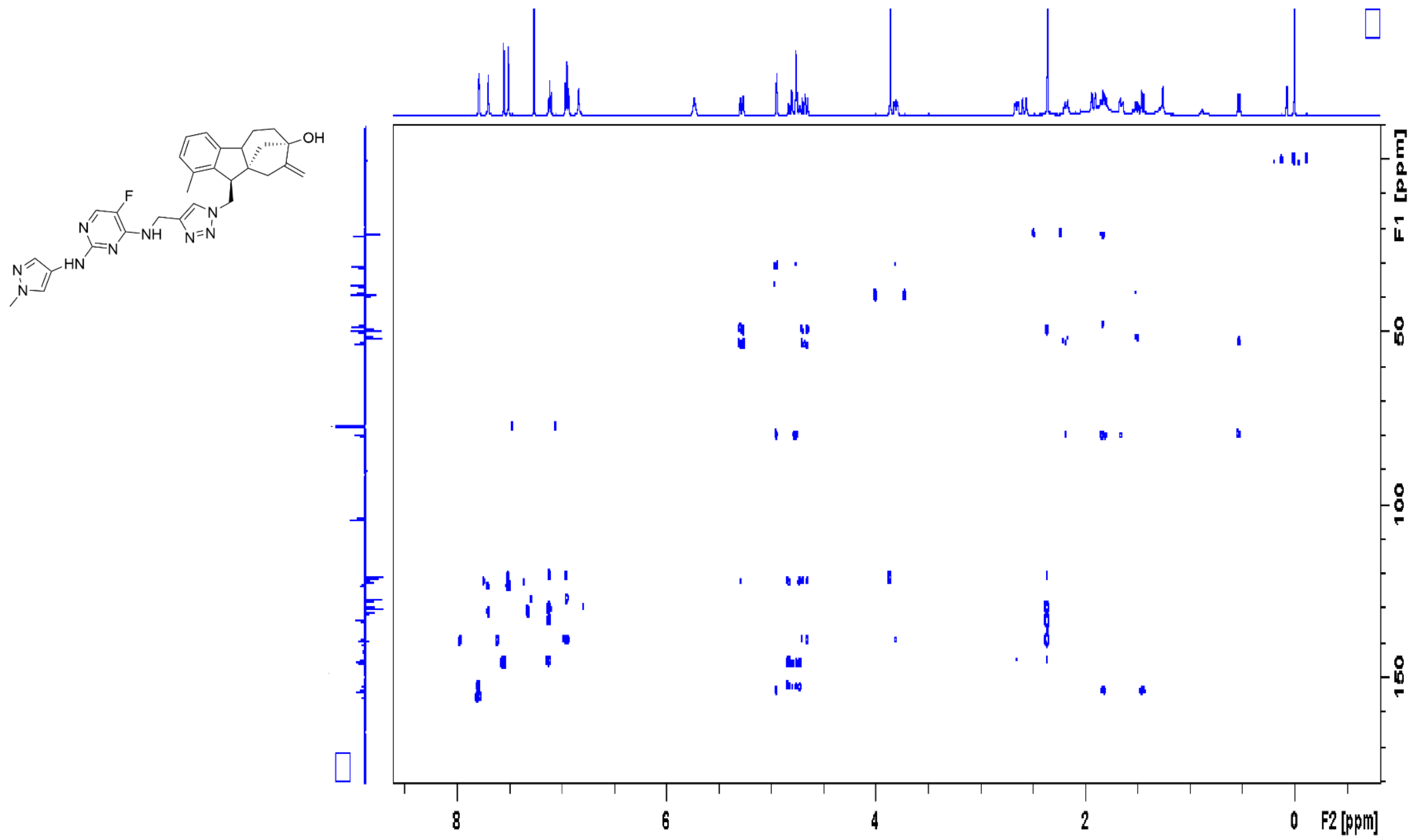

(7S,9aS,10R)-10-((4-(((5-Chloro-2-((1-methyl-1H-pyrazol-4-yl)amino)pyrimidin-4-yl)amino)me-thyl)-1H-1,2,3-triazol-1-yl)methyl)-1-methyl-8-methylene-4b,5,6,8,9,10-hexahydro-7H-7,9a-methanobenzo[a]azulen-7-ol (**22**)

Figure S49. ^{1}H-NMR of compound **22**

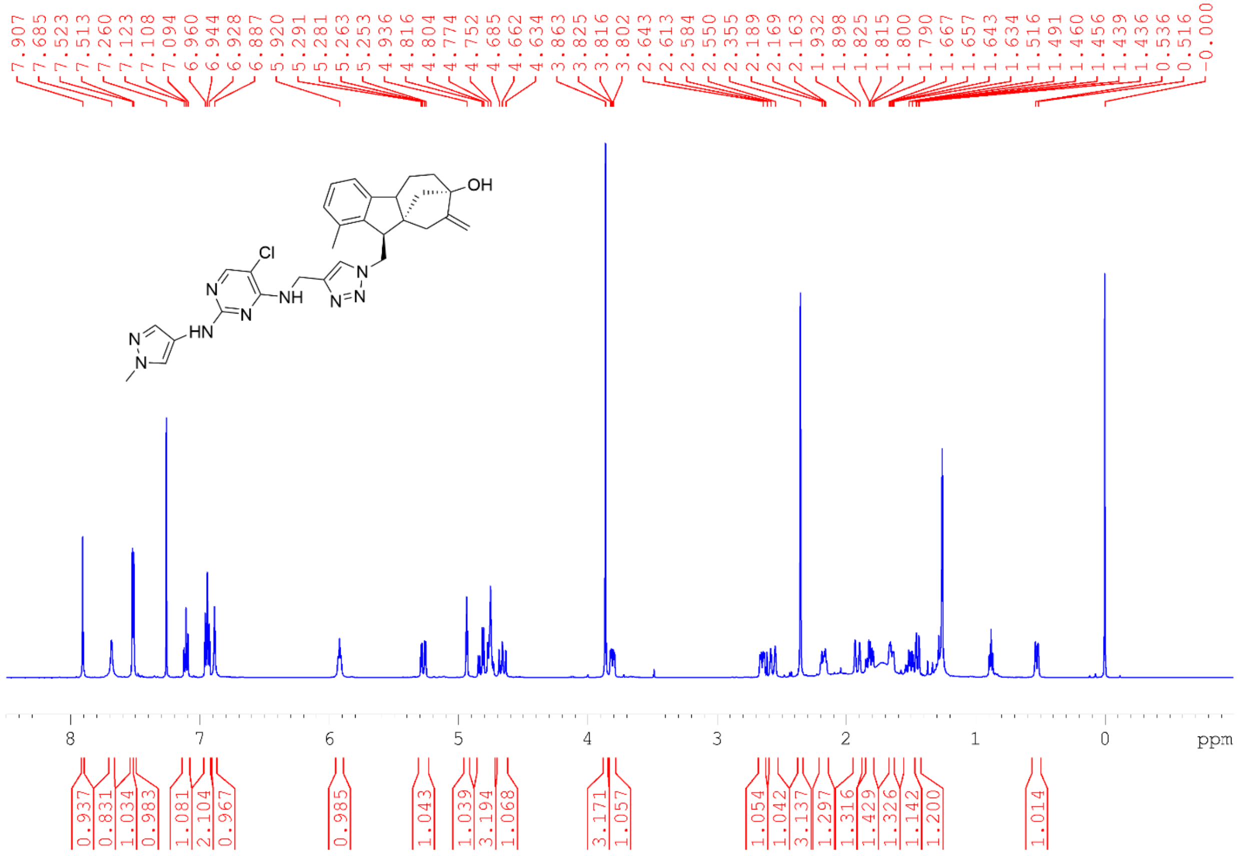

Figure S50. ^{13}C-NMR of compound **22**

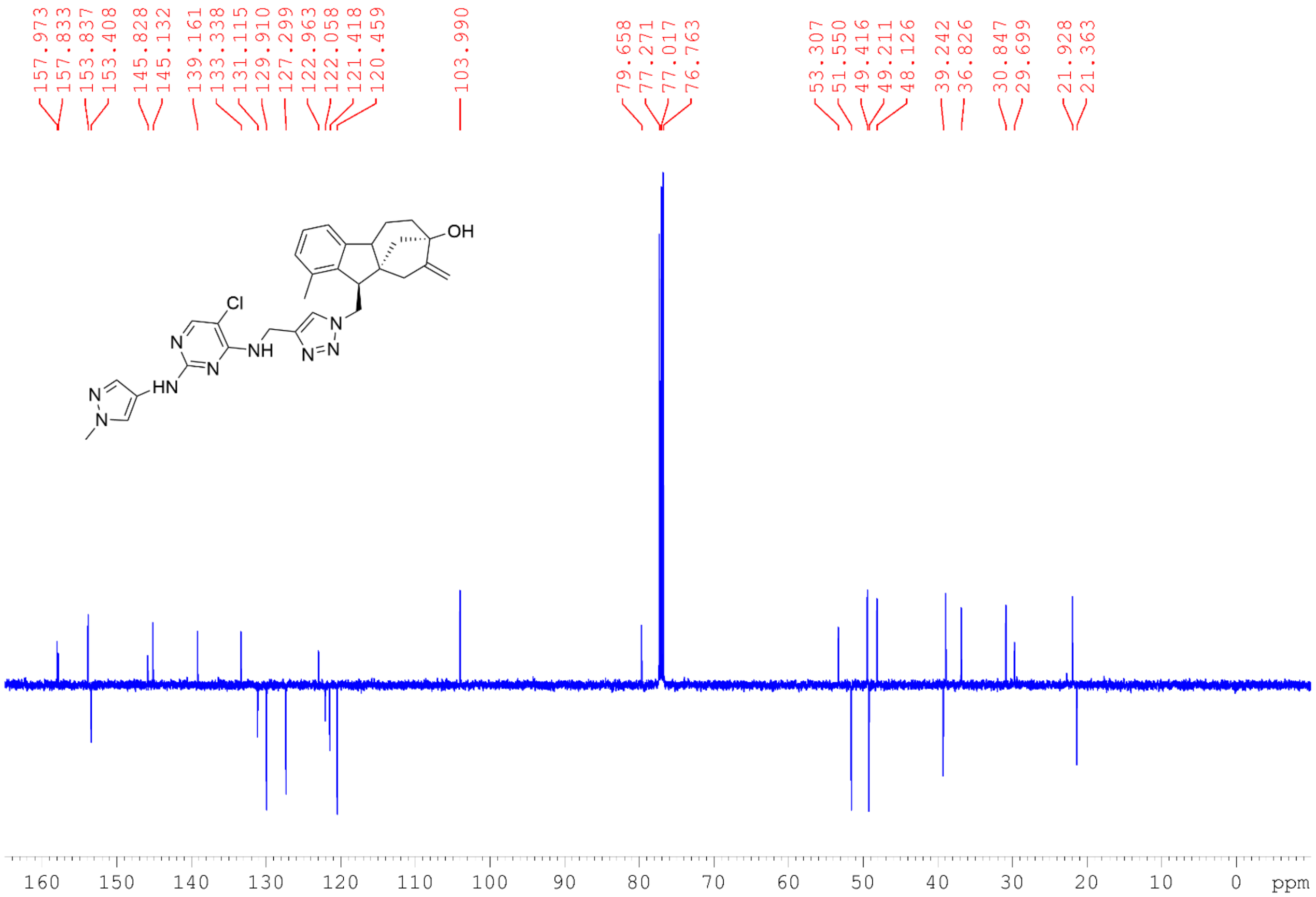

Figure S51. COSY-NMR of compound **22**

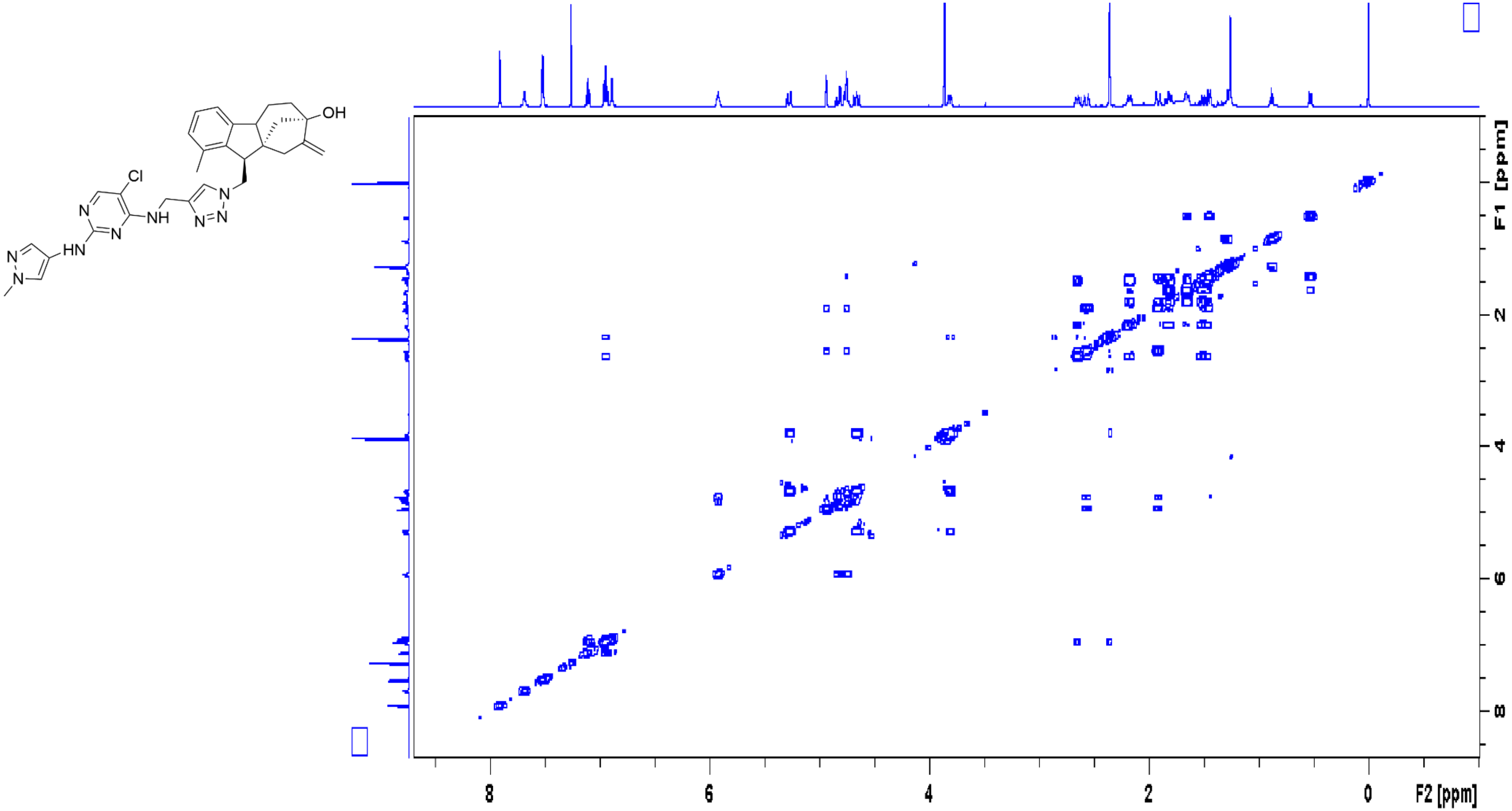

Figure S52. NOESY-NMR of compound **22**

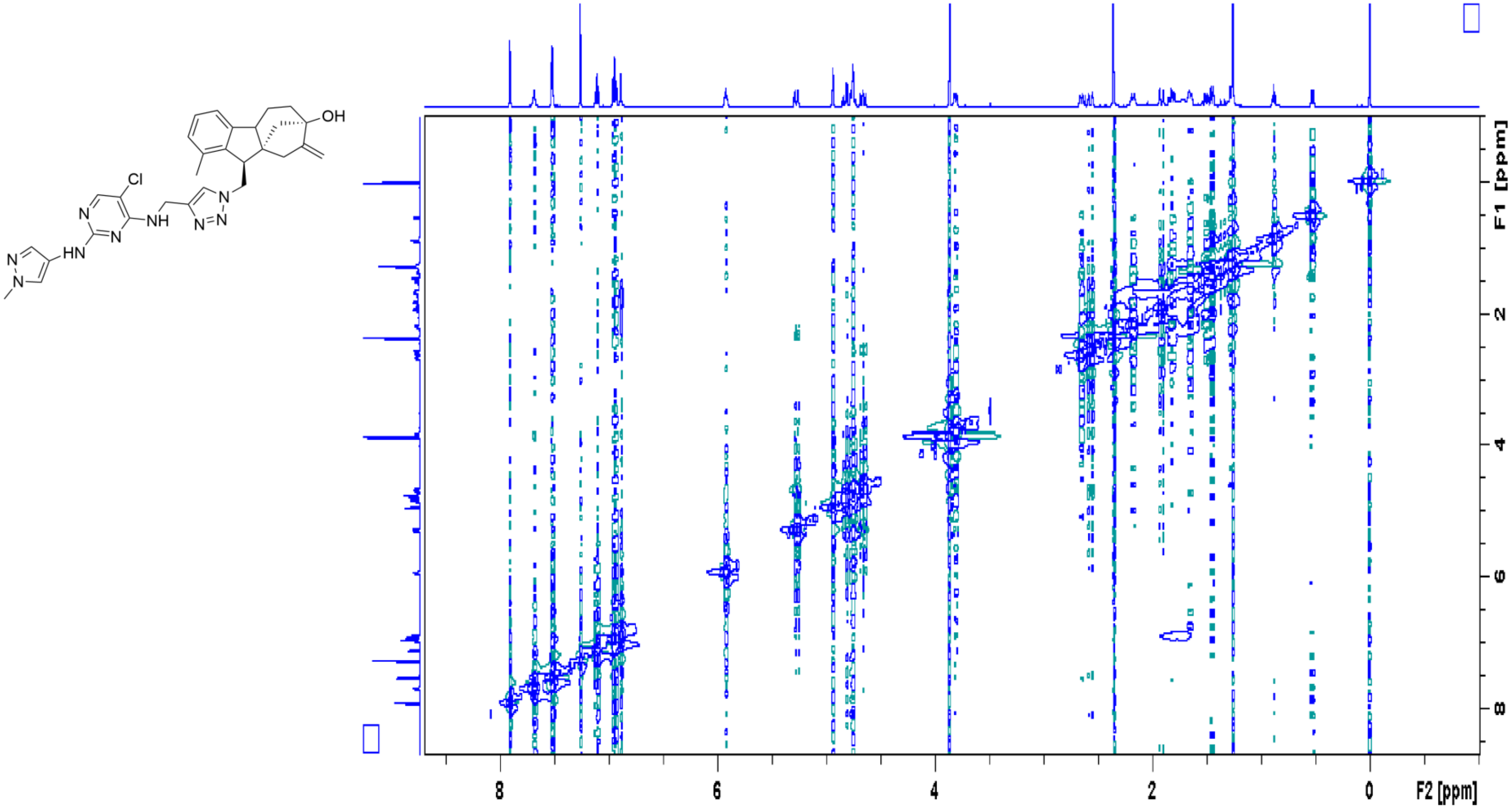

Figure S53. HSQC-NMR of compound **22**

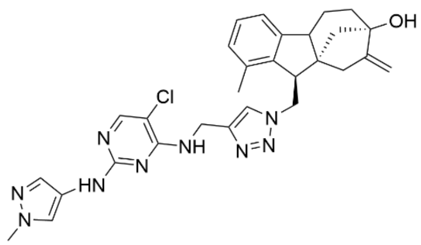

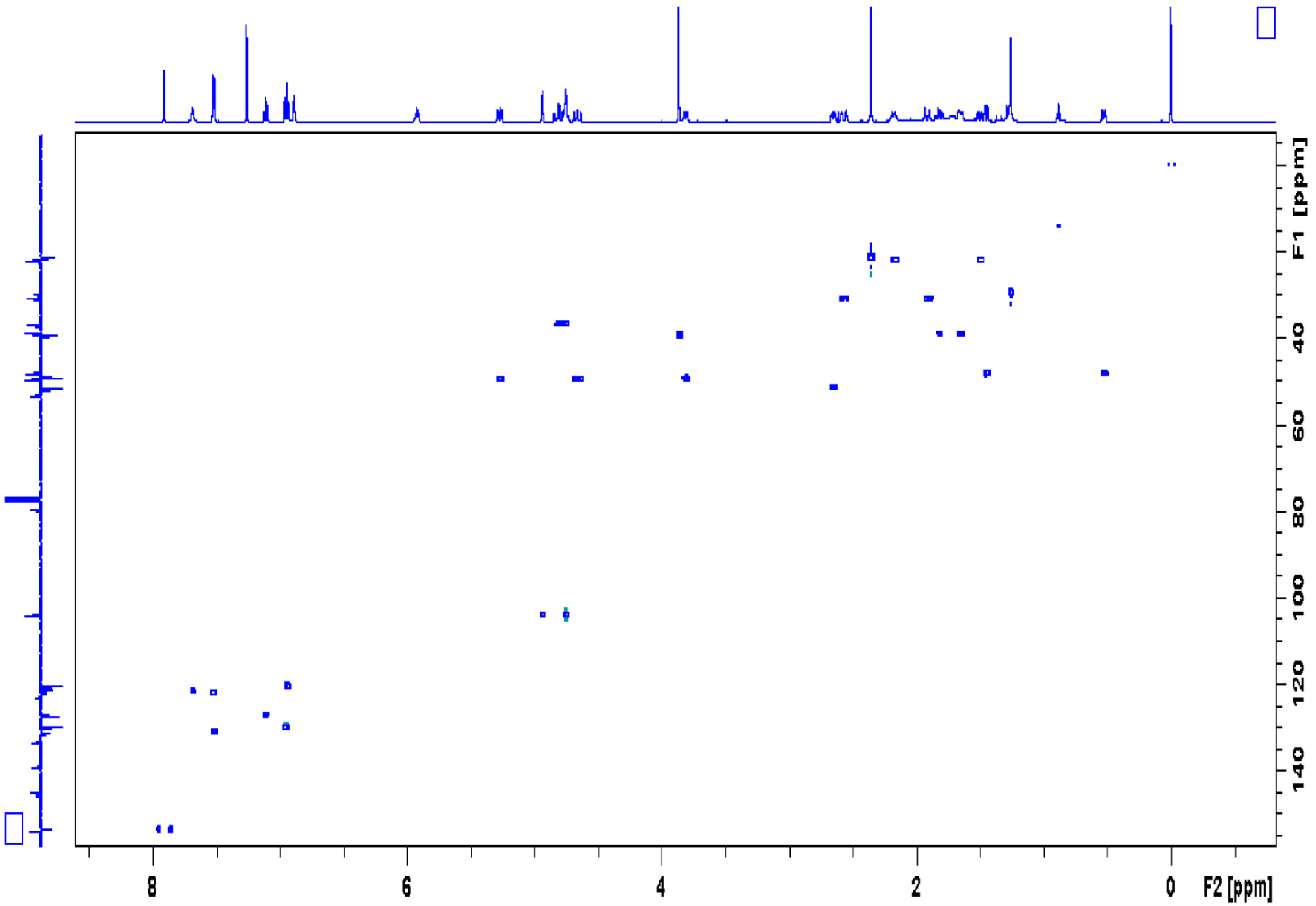

Figure S54. HMBC-NMR of compound **22**

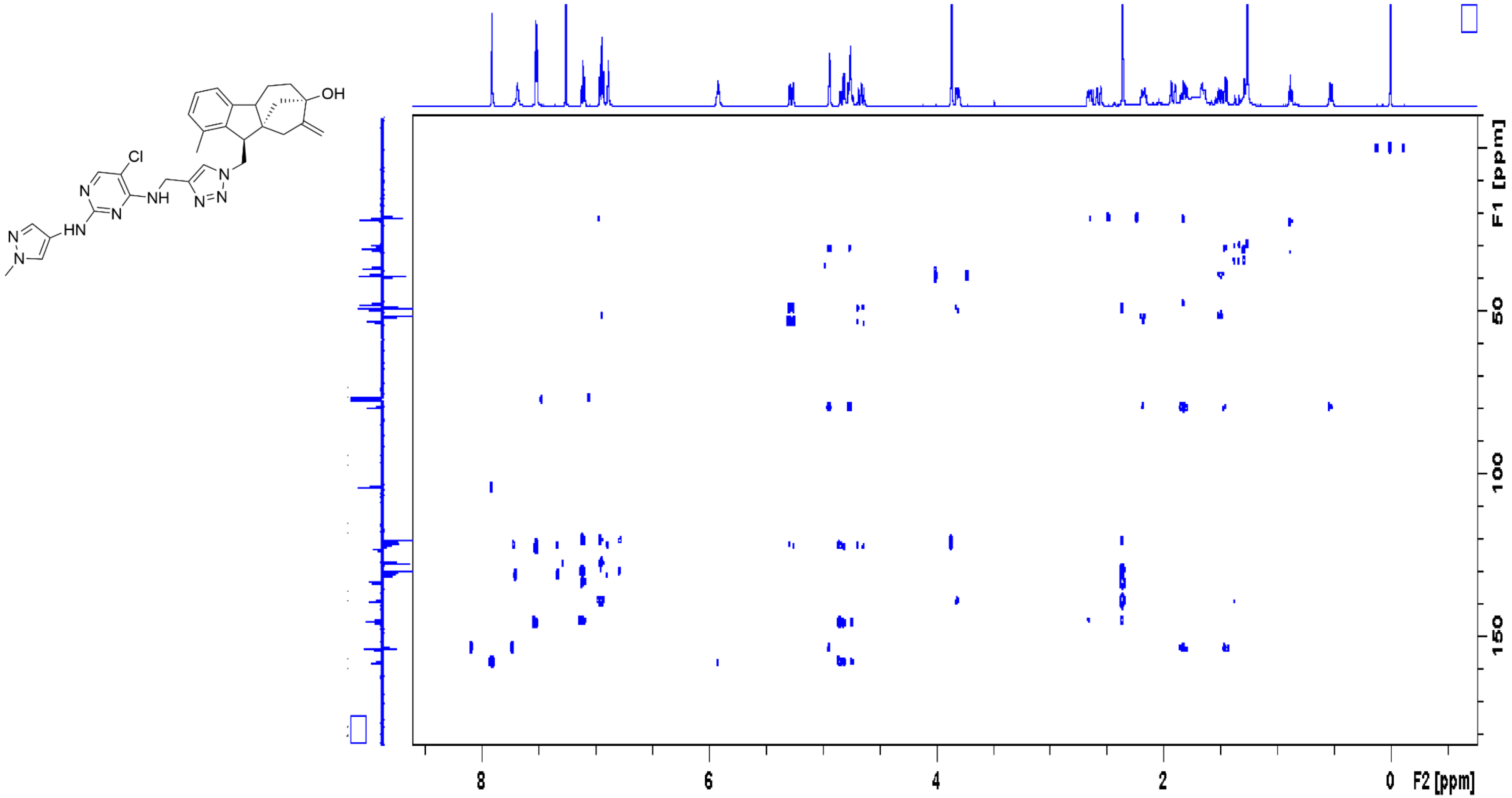

Methyl 4-((5-fluoro-4-(((1-(((7S,9aS,10R)-7-hydroxy-1-methyl-8-methylene-5,6,7,8,9,10-hexahydro-4bH-7,9a-methanobenzo[a]azulen-10-yl)methyl)-1H-1,2,3-triazol-4-yl)methyl)amino)pyrimidin-2-yl)amino)benzoate (**23**)

Figure S55. ^{1}H-NMR of compound **23**

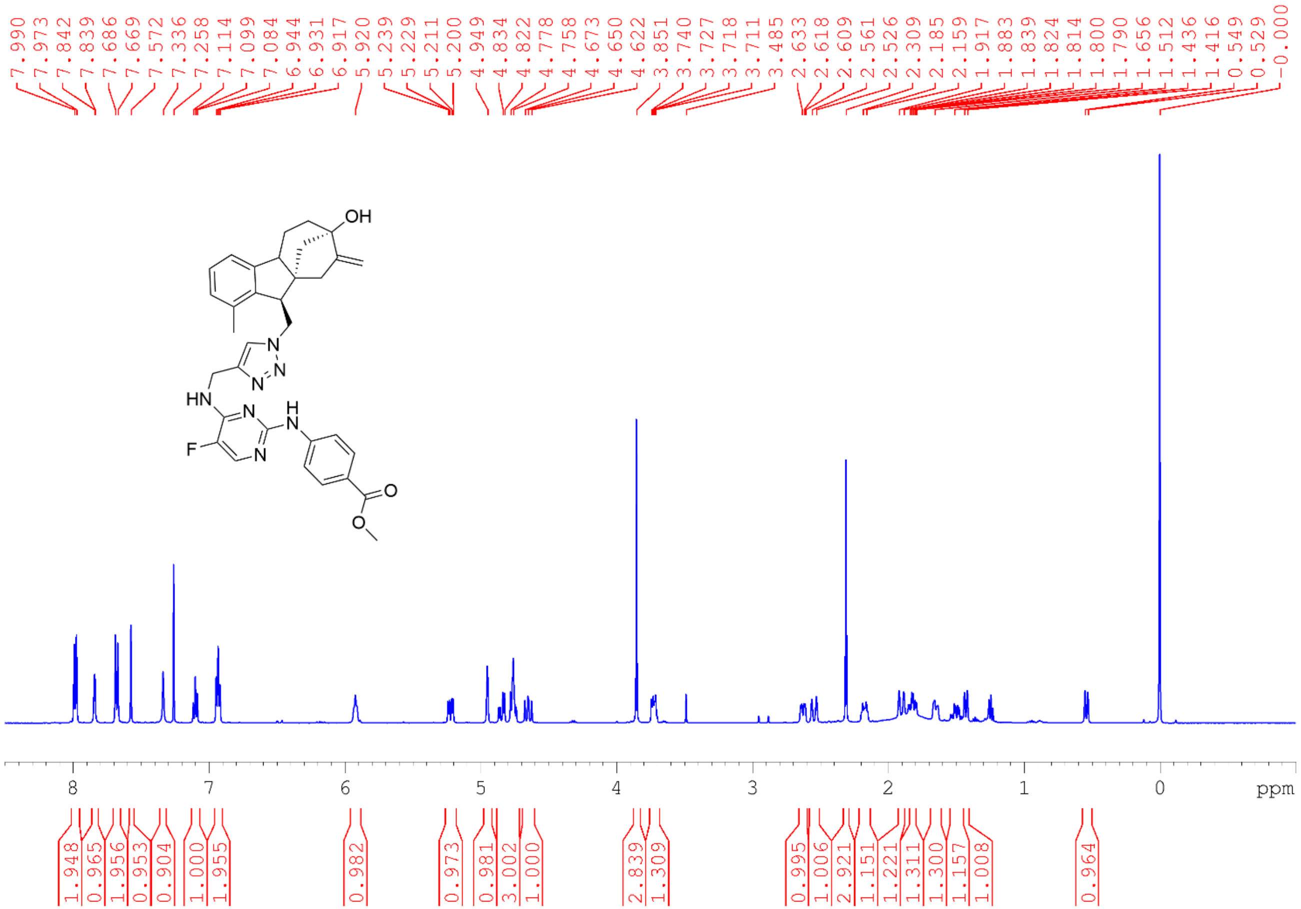

Figure S56. ^{13}C-NMR of compound **23**

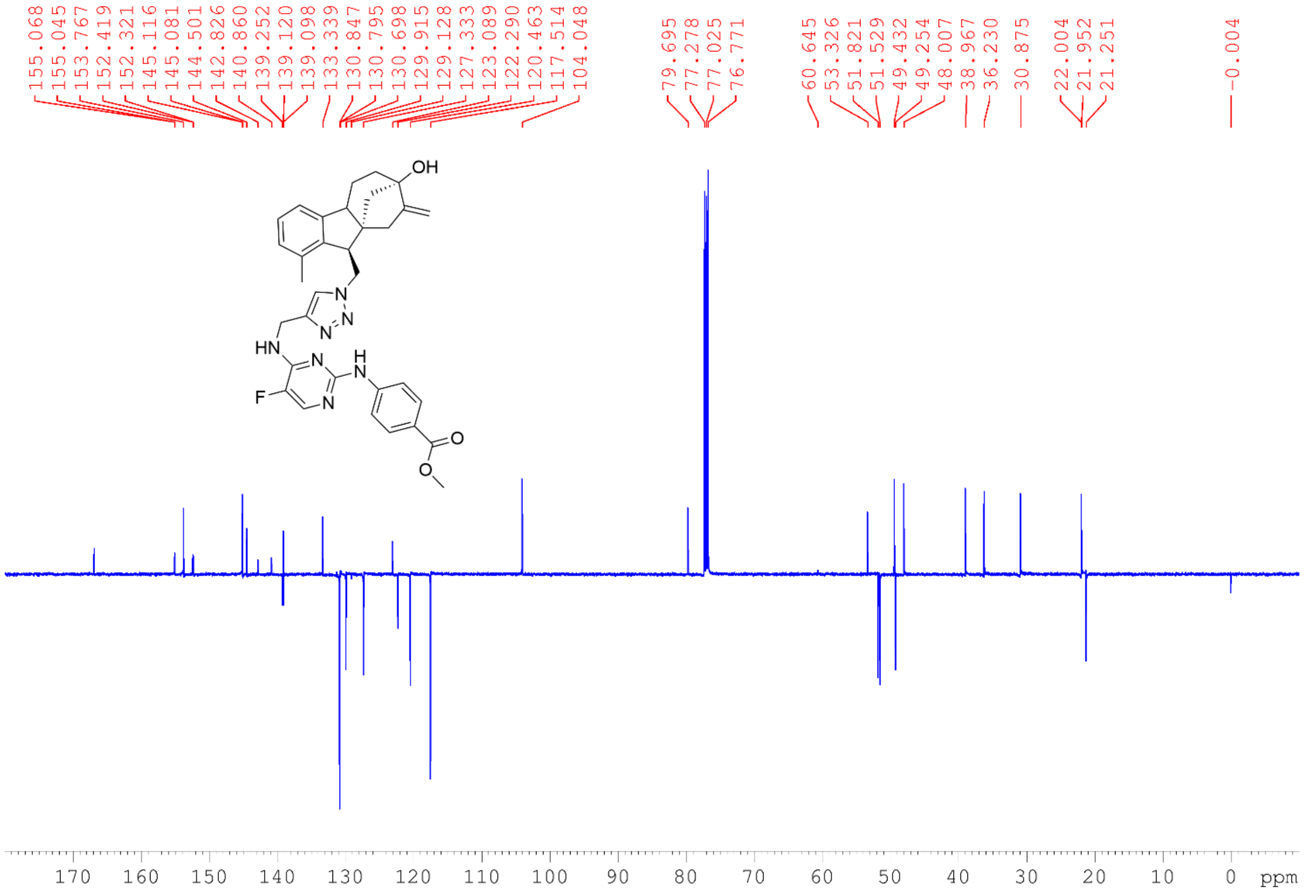

Figure S57. ^{19}F-NMR of compound **23**

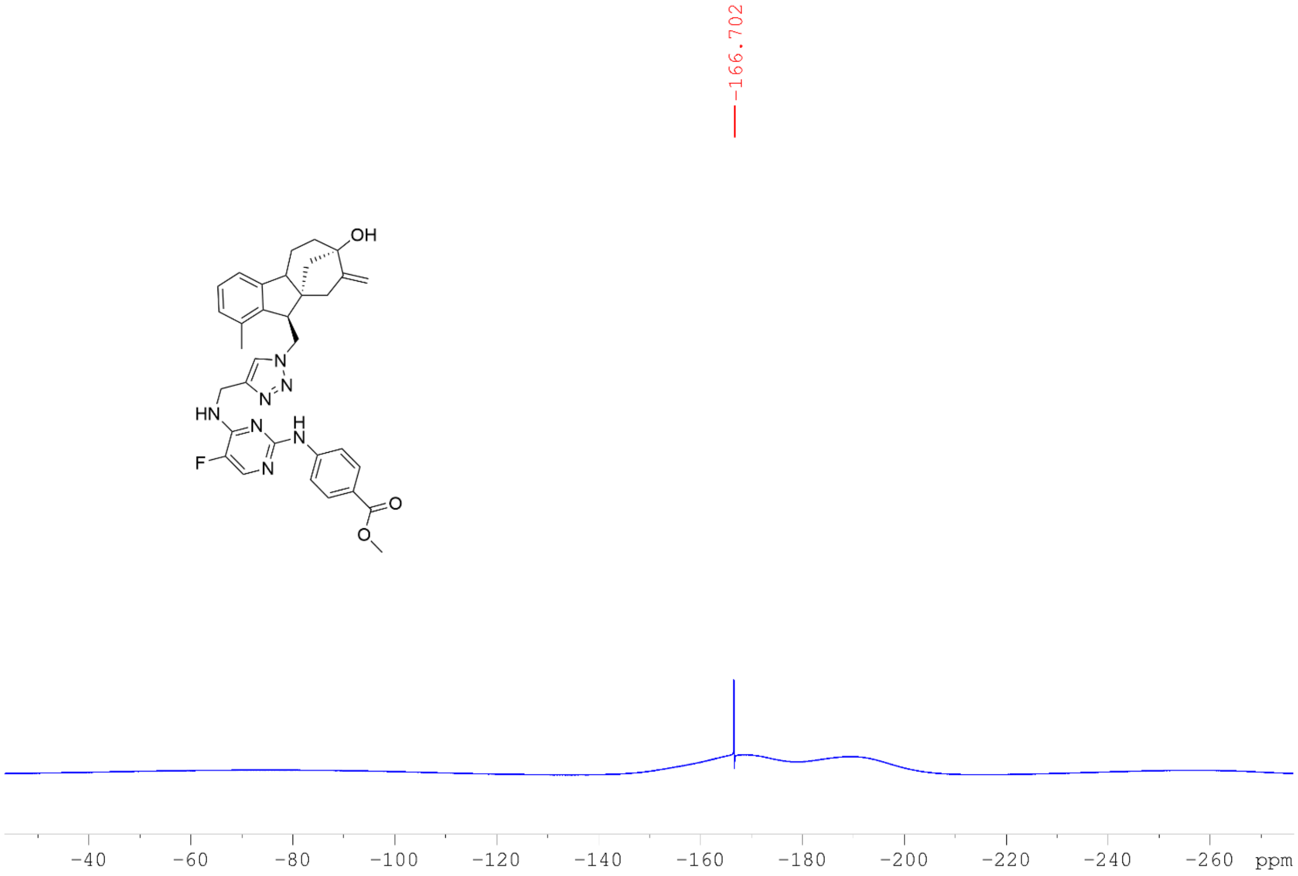

Figure S58. COSY-NMR of compound **23**

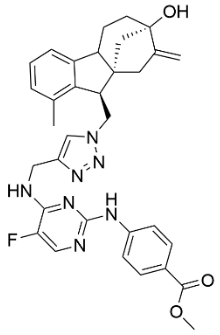

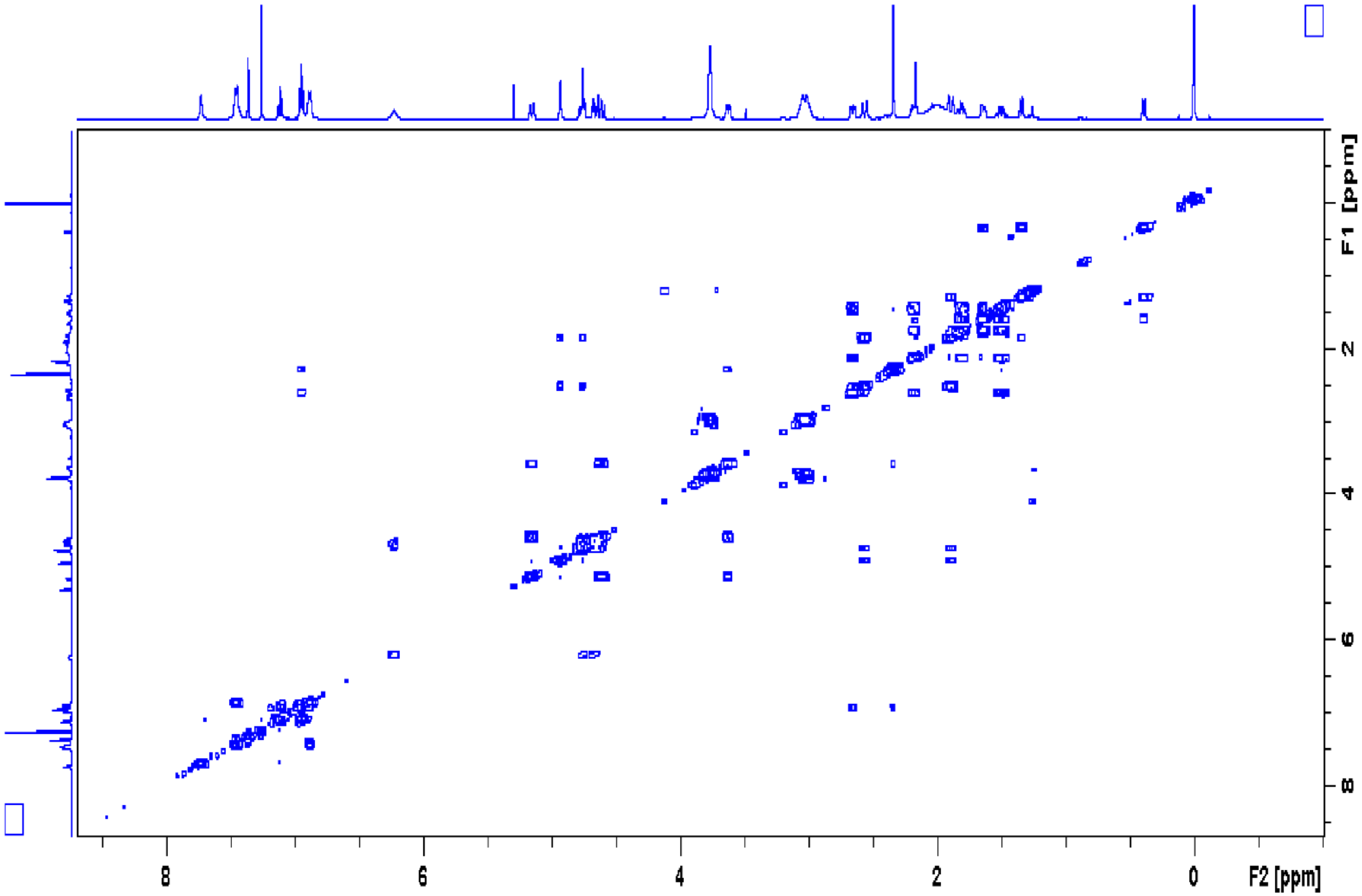

Figure S59. NOESY-NMR of compound **23**

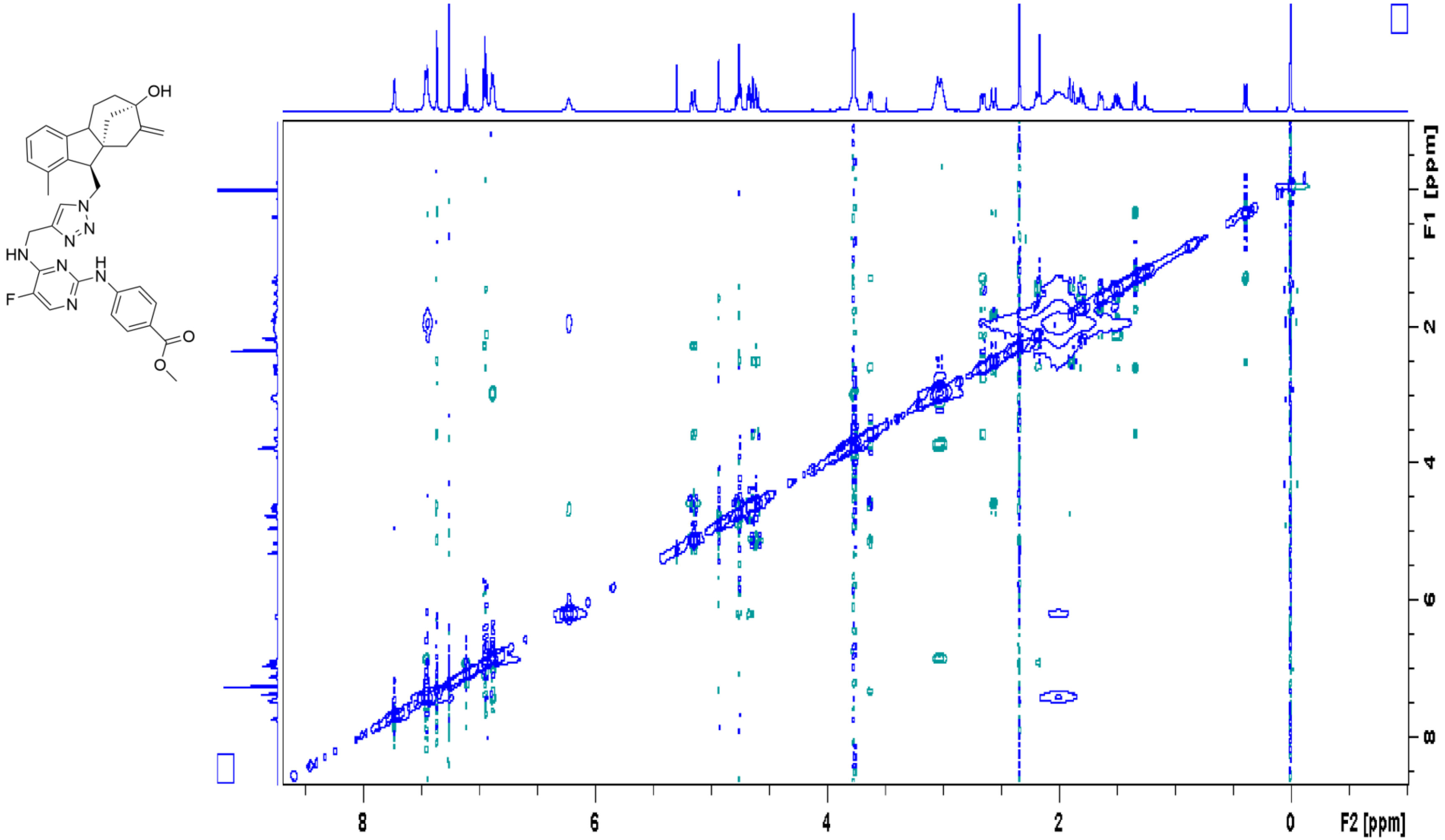

Figure S60. HSQC-NMR of compound **23**

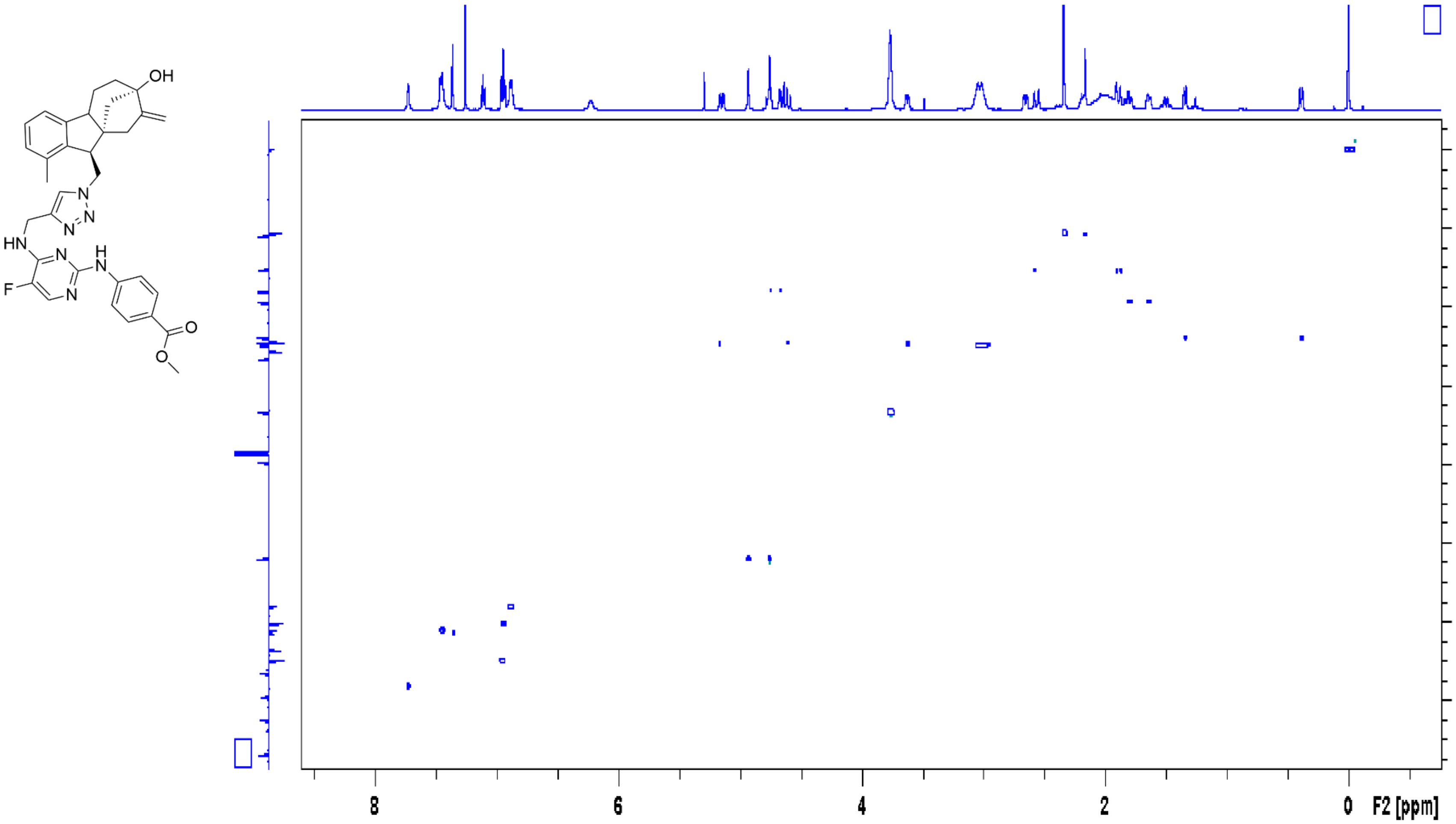

Figure S61. HMBC-NMR of compound **23**

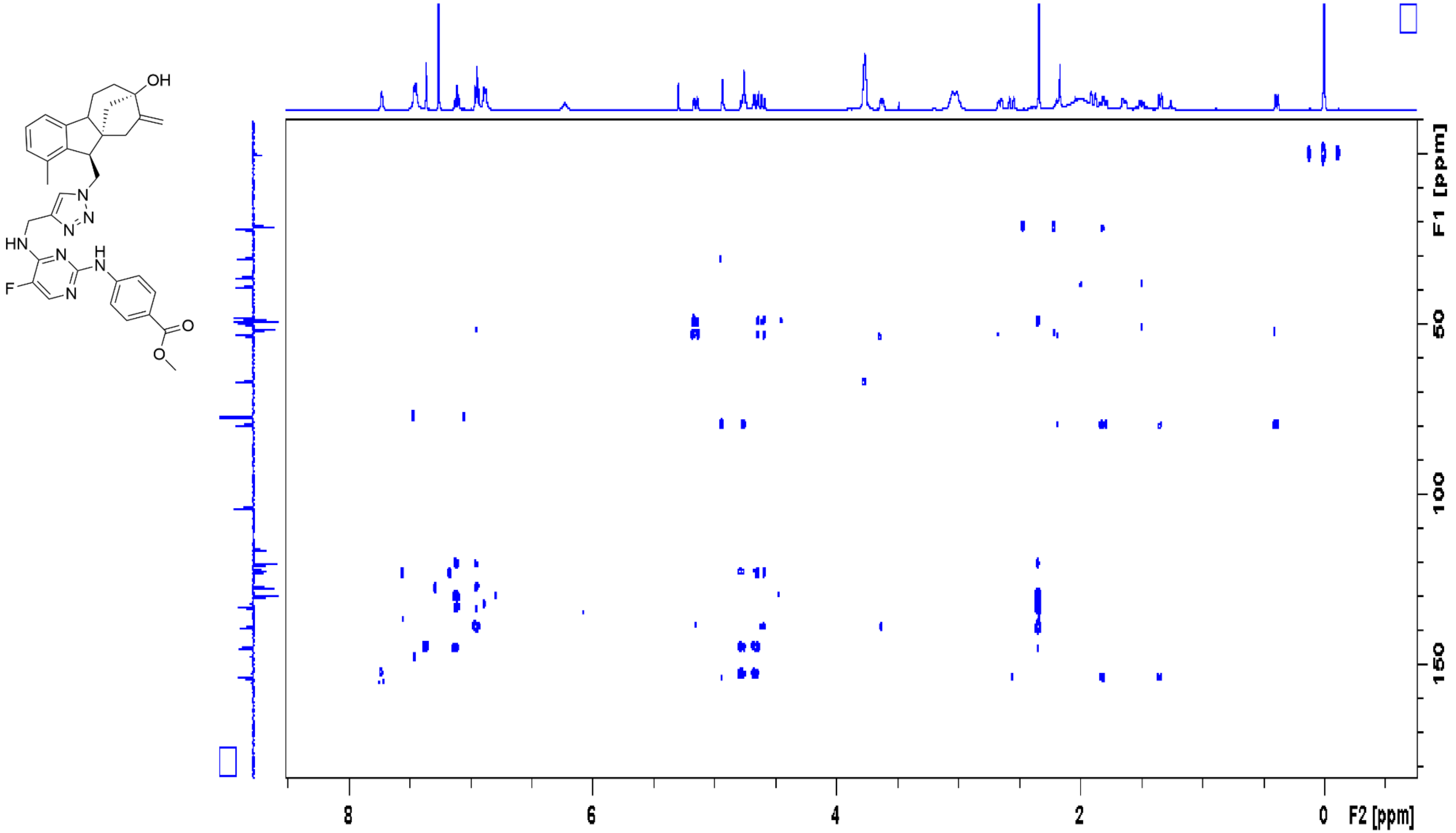

Methyl 4-((5-chloro-4-(((1-(((7S,9aS,10R)-7-hydroxy-1-methyl-8-methylene-4b,6,7,8,9,10-hexahydro-5H-7,9a-methanobenzo[a]azulen-10-yl)methyl)-1H-1,2,3-triazol-4-yl)methyl)amino)pyrimidin-2-yl)amino)benzoate (**24**)

Figure S62. ^{1}H-NMR of compound **24**

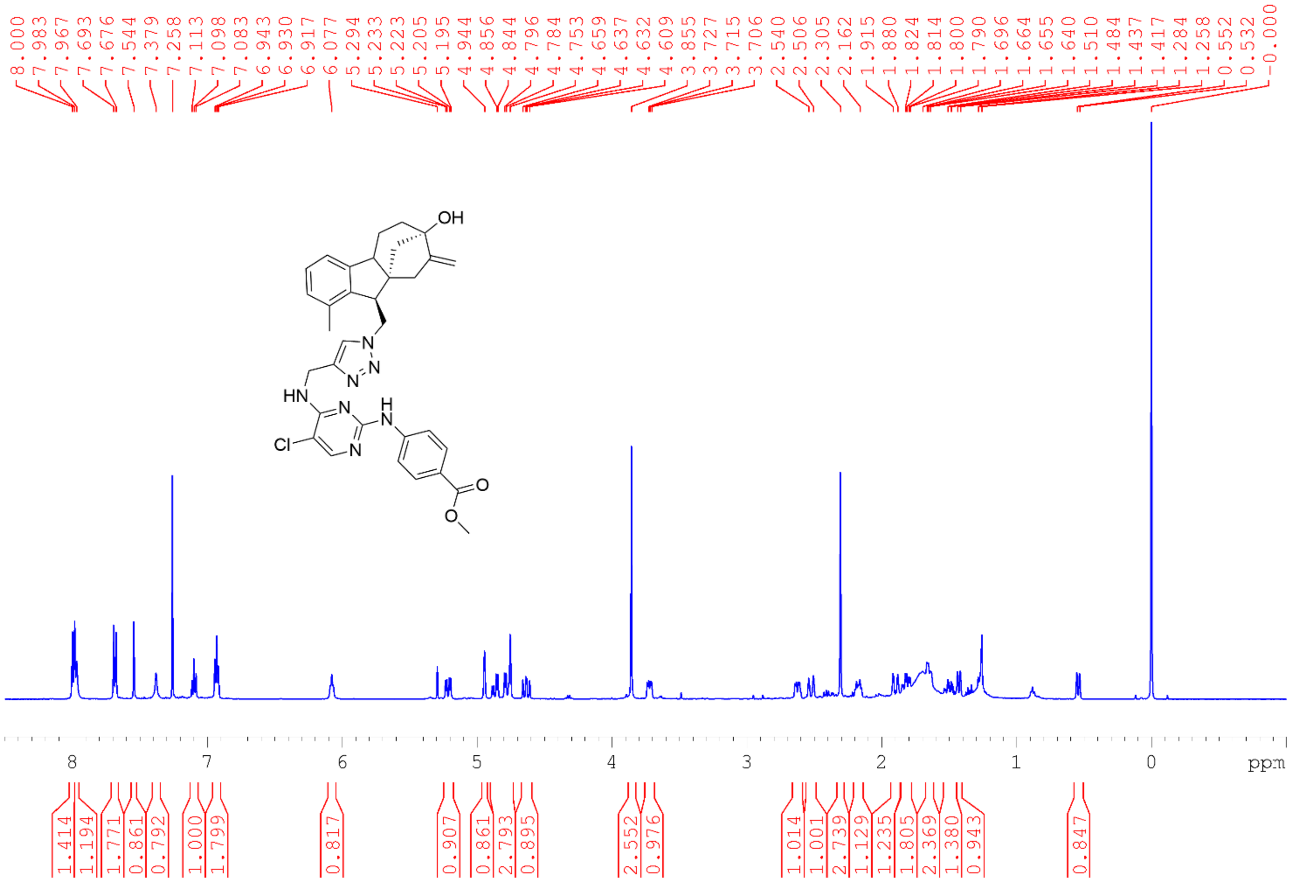

Figure S63. ^{13}C-NMR of compound **24**

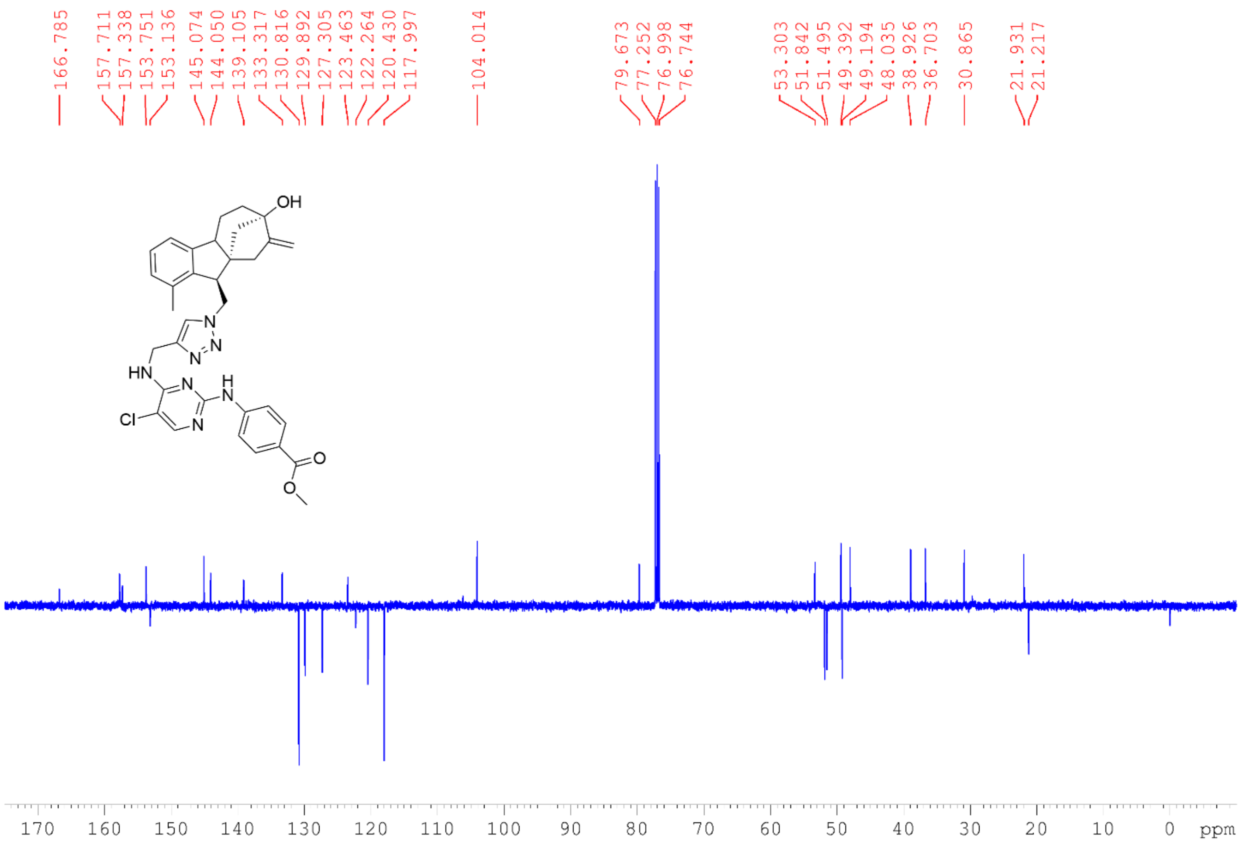

Figure S64. COSY-NMR of compound **24**

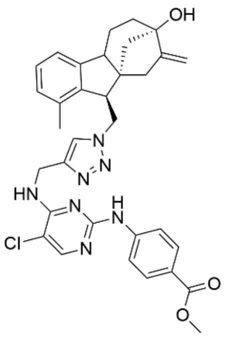

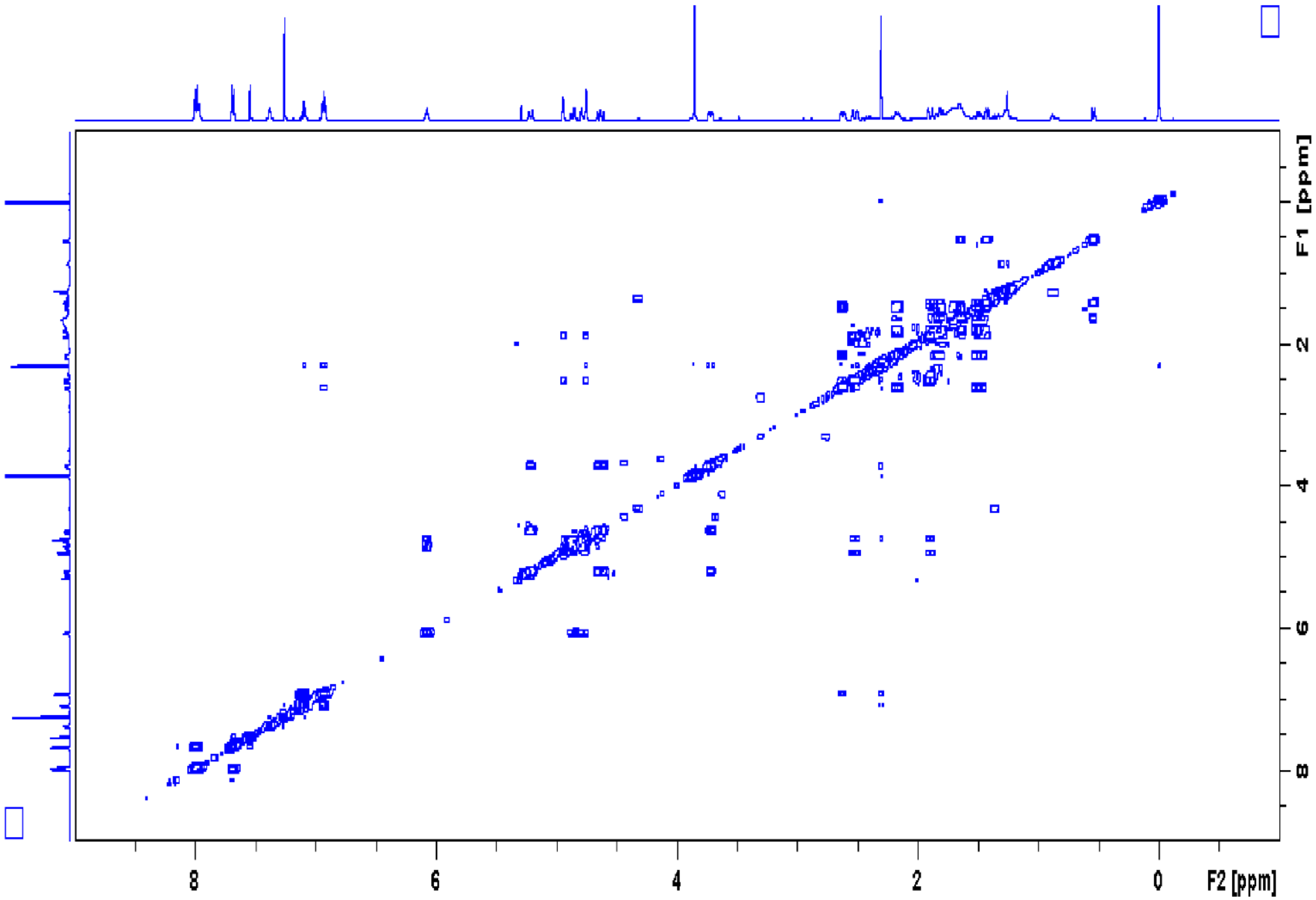

Figure S65. NOESY-NMR of compound **24**

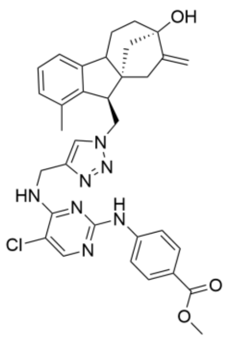

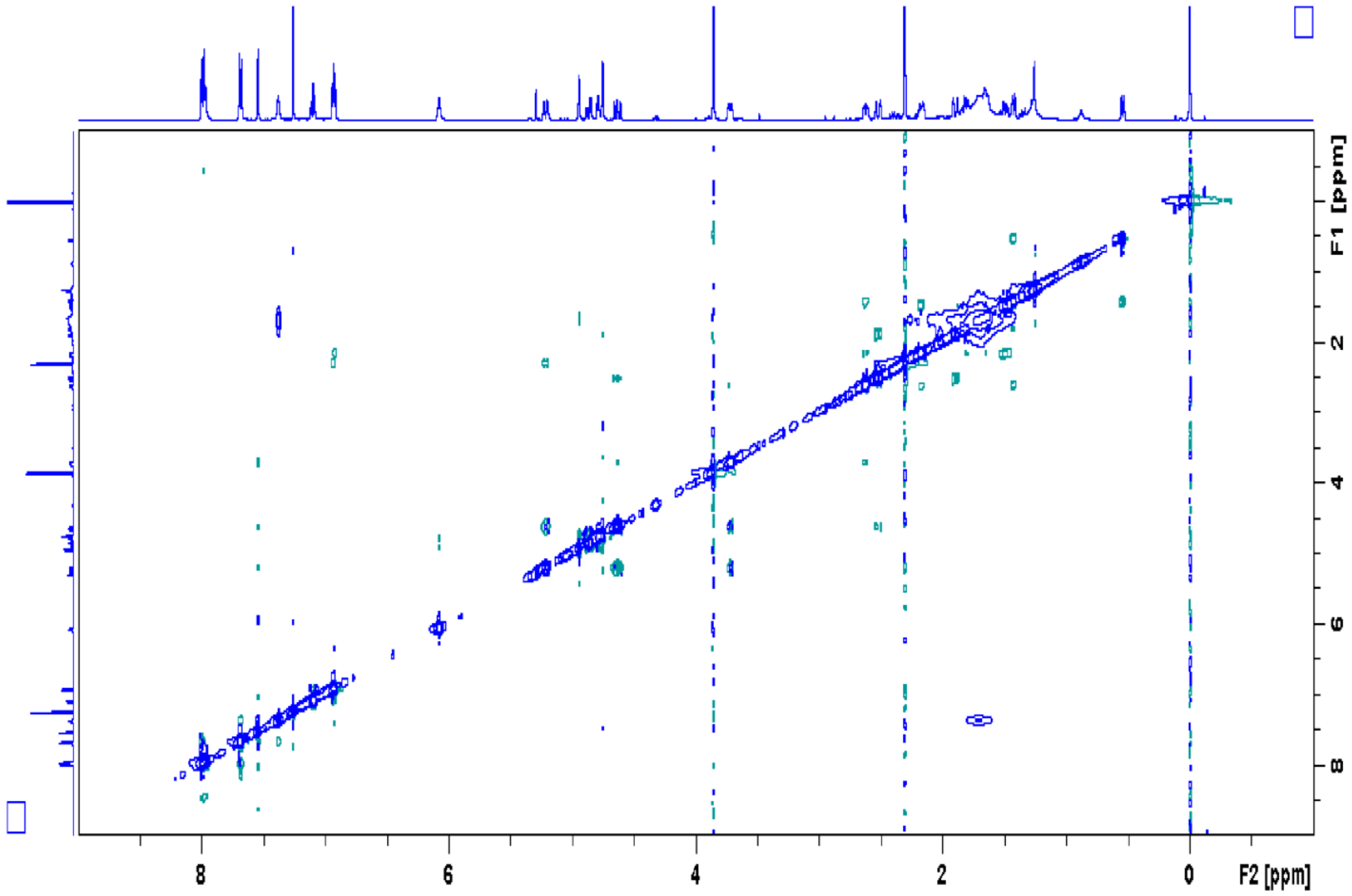

Figure S66. HSQC-NMR of compound **24**

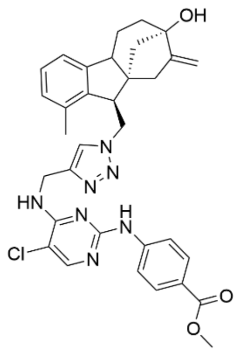

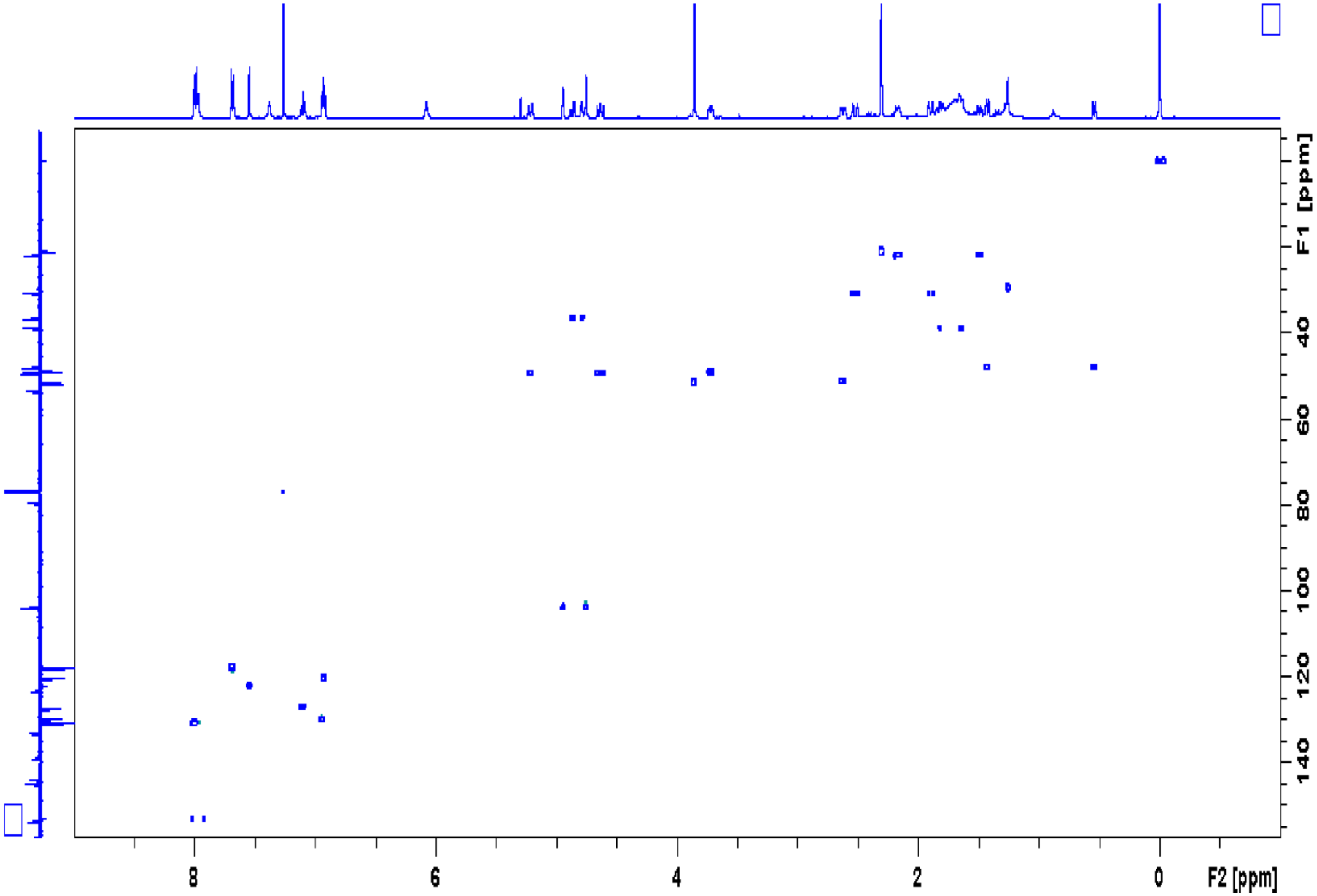

Figure S67. HMBC-NMR of compound **24**

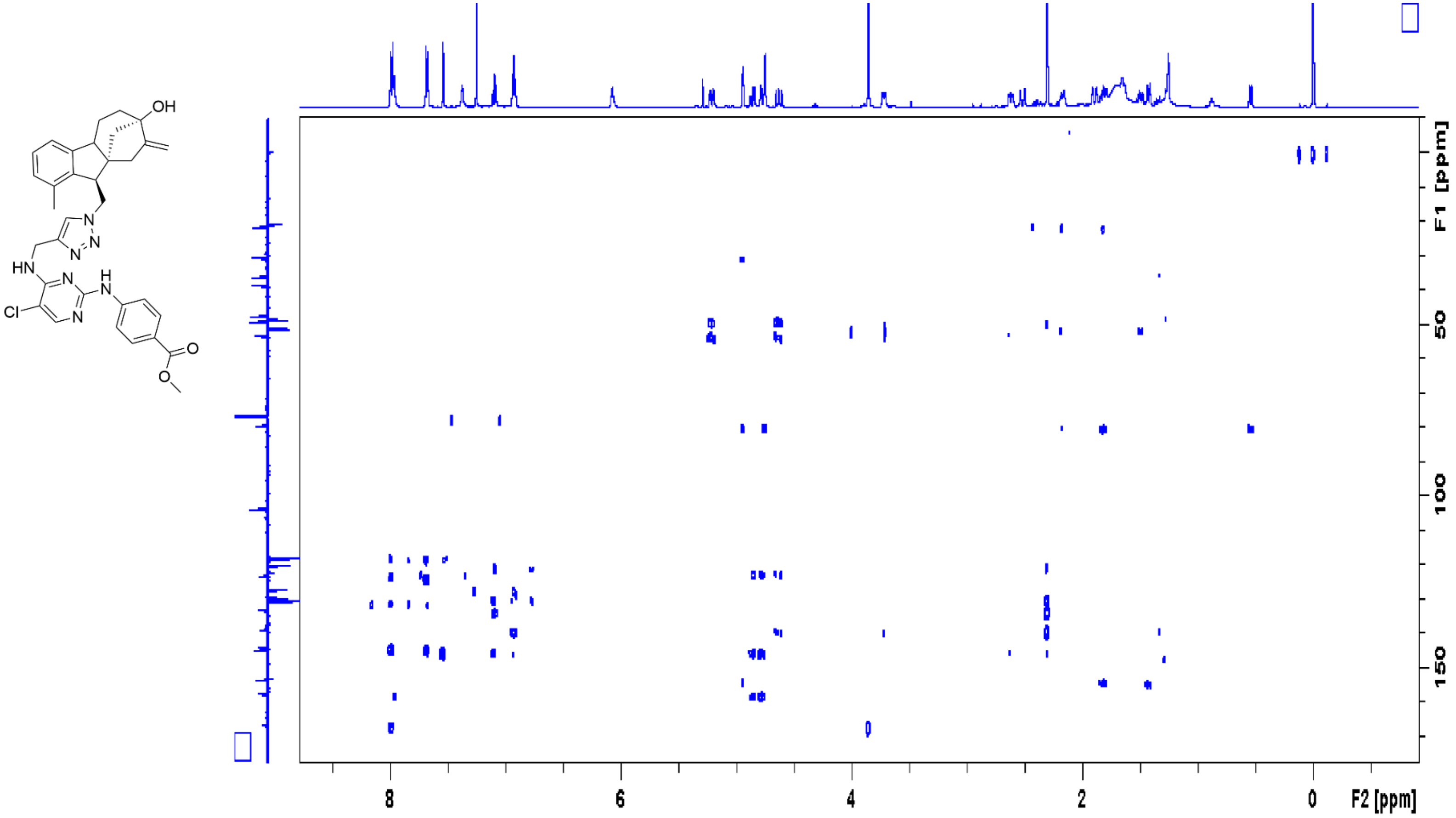

(7S,9aS,10R)-10-((4-(((5-Fluoro-2-((4-morpholinophenyl)amino)pyrimidin-4-yl)amino)methyl)-1H-1,2,3-triazol-1-yl)methyl)-1-methyl-8-methylene-4b,5,6,8,9,10-hexahydro-7H-7,9a-methanobenzo[a]azulen-7-ol (**25**)

Figure S68. ^{1}H-NMR of compound **25**

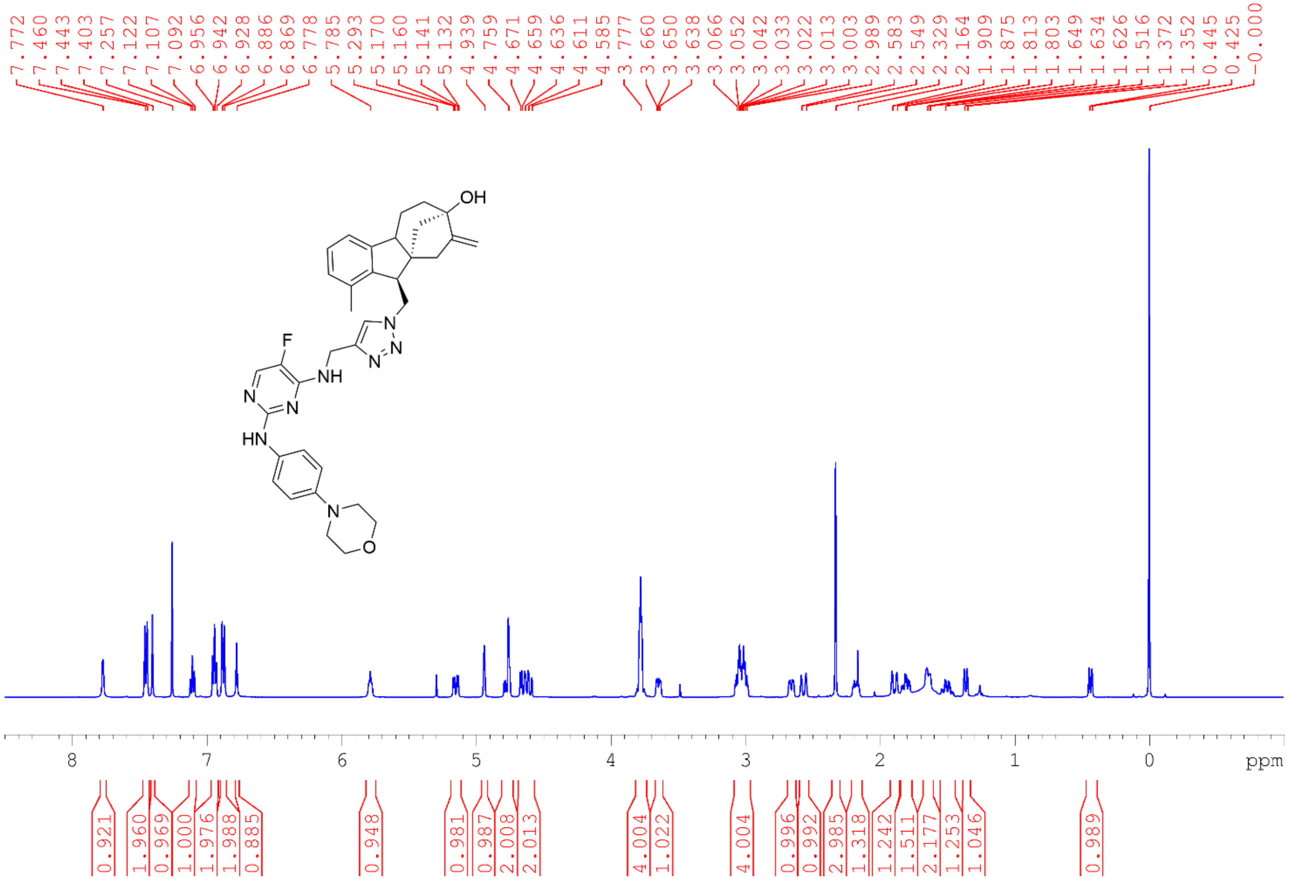

Figure S69. ^{13}C-NMR of compound **25**

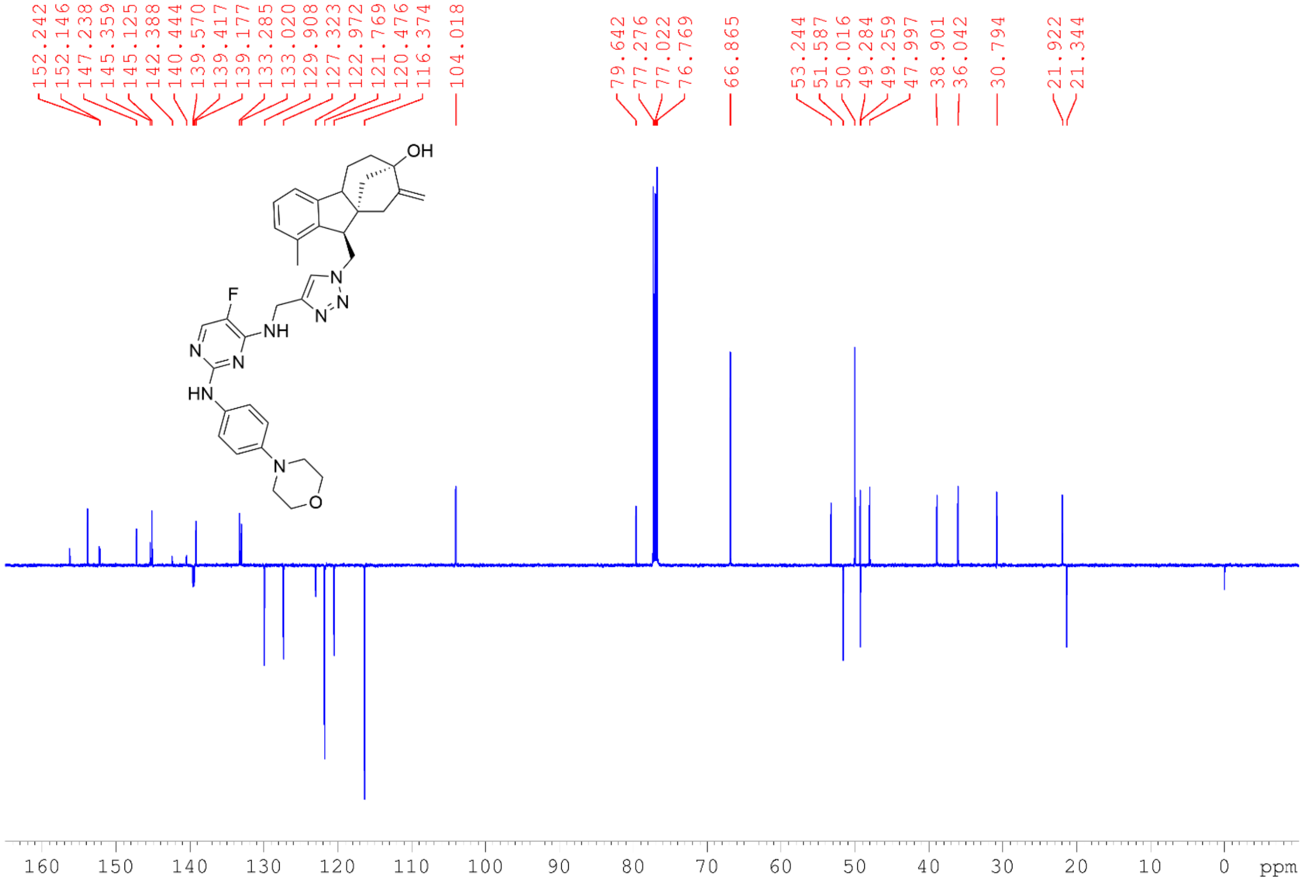

Figure S70. ^{19}F-NMR of compound **25**

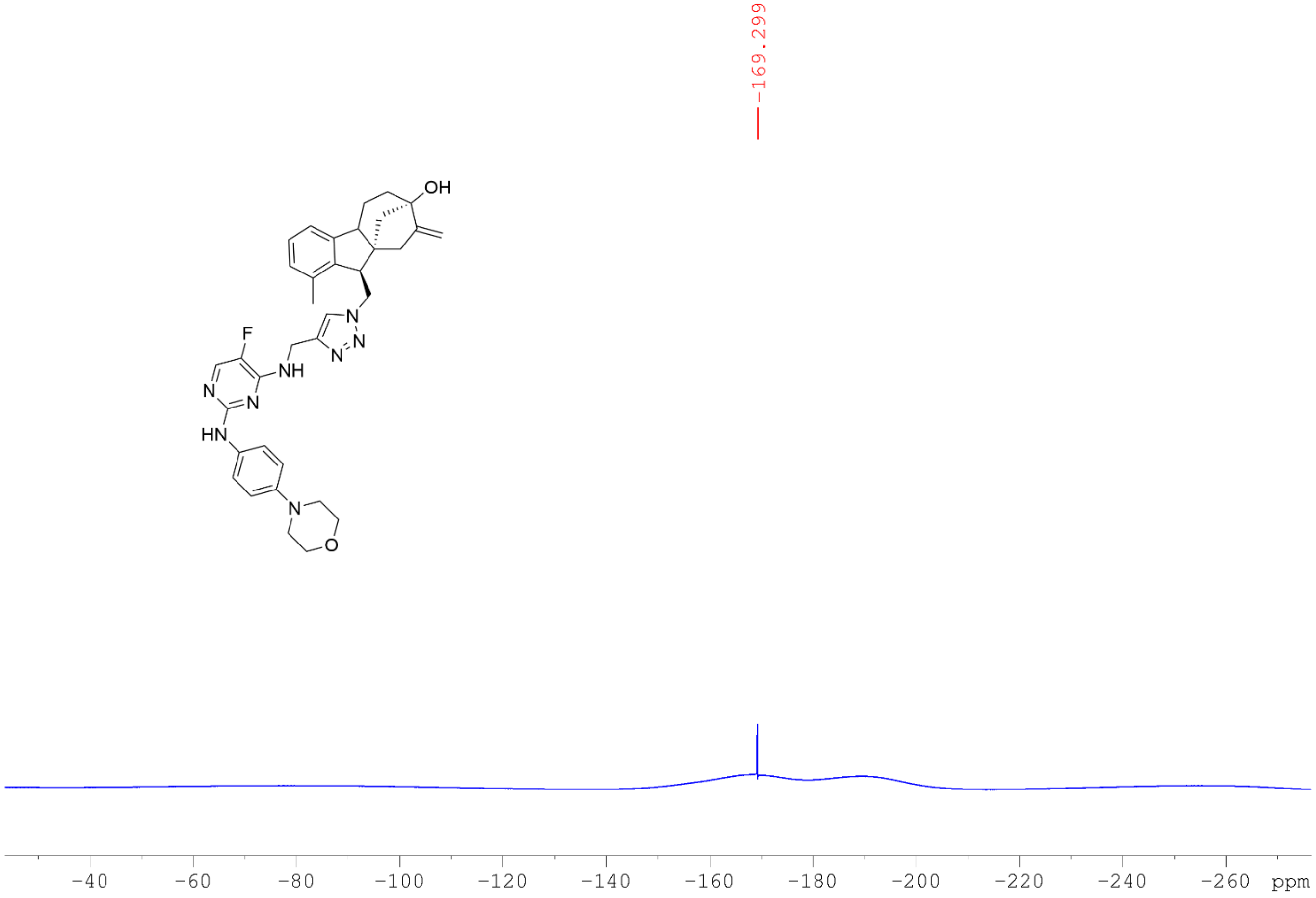

Figure S71. COSY-NMR of compound **25**

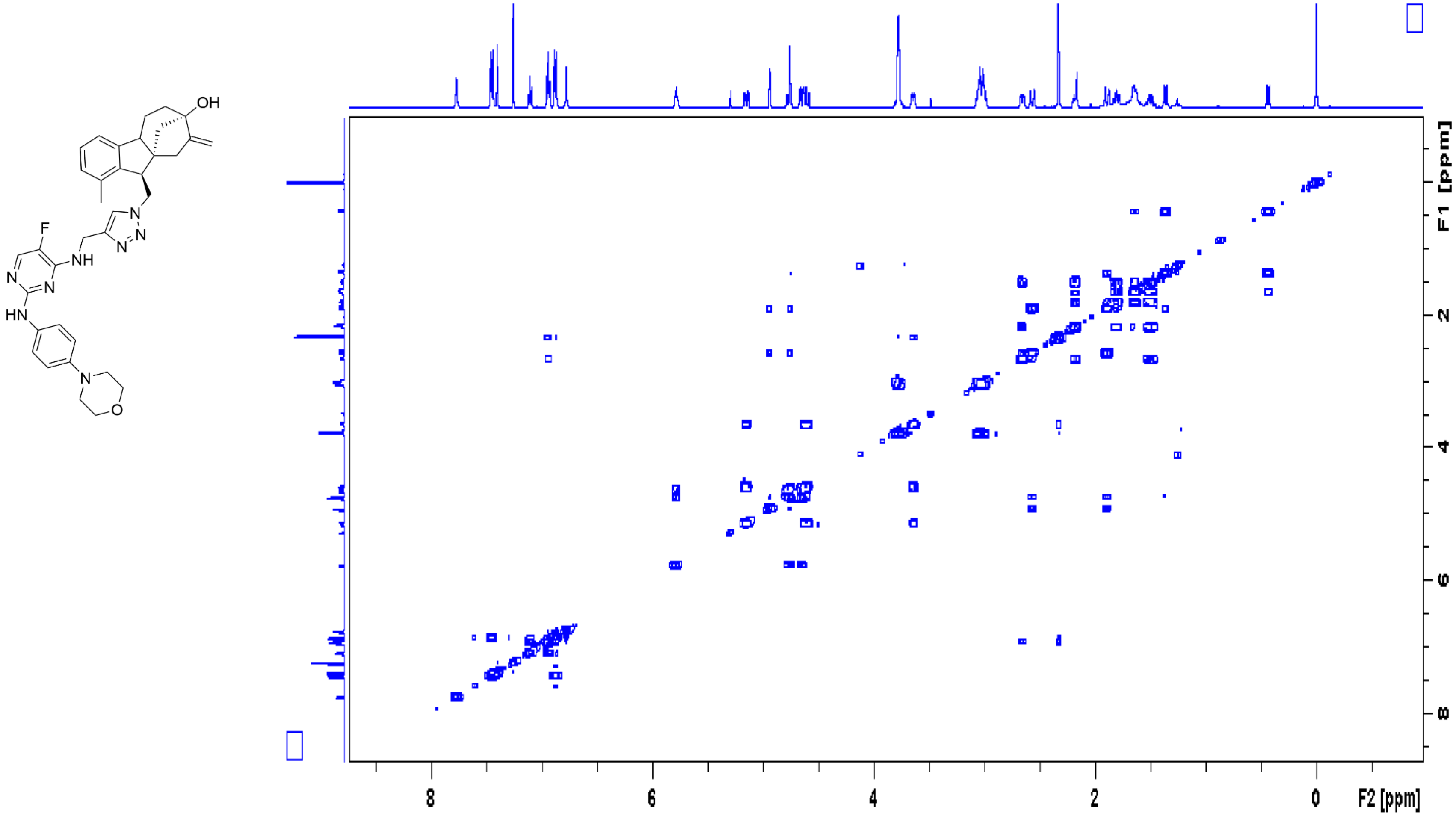

Figure S72. NOESY-NMR of compound **25**

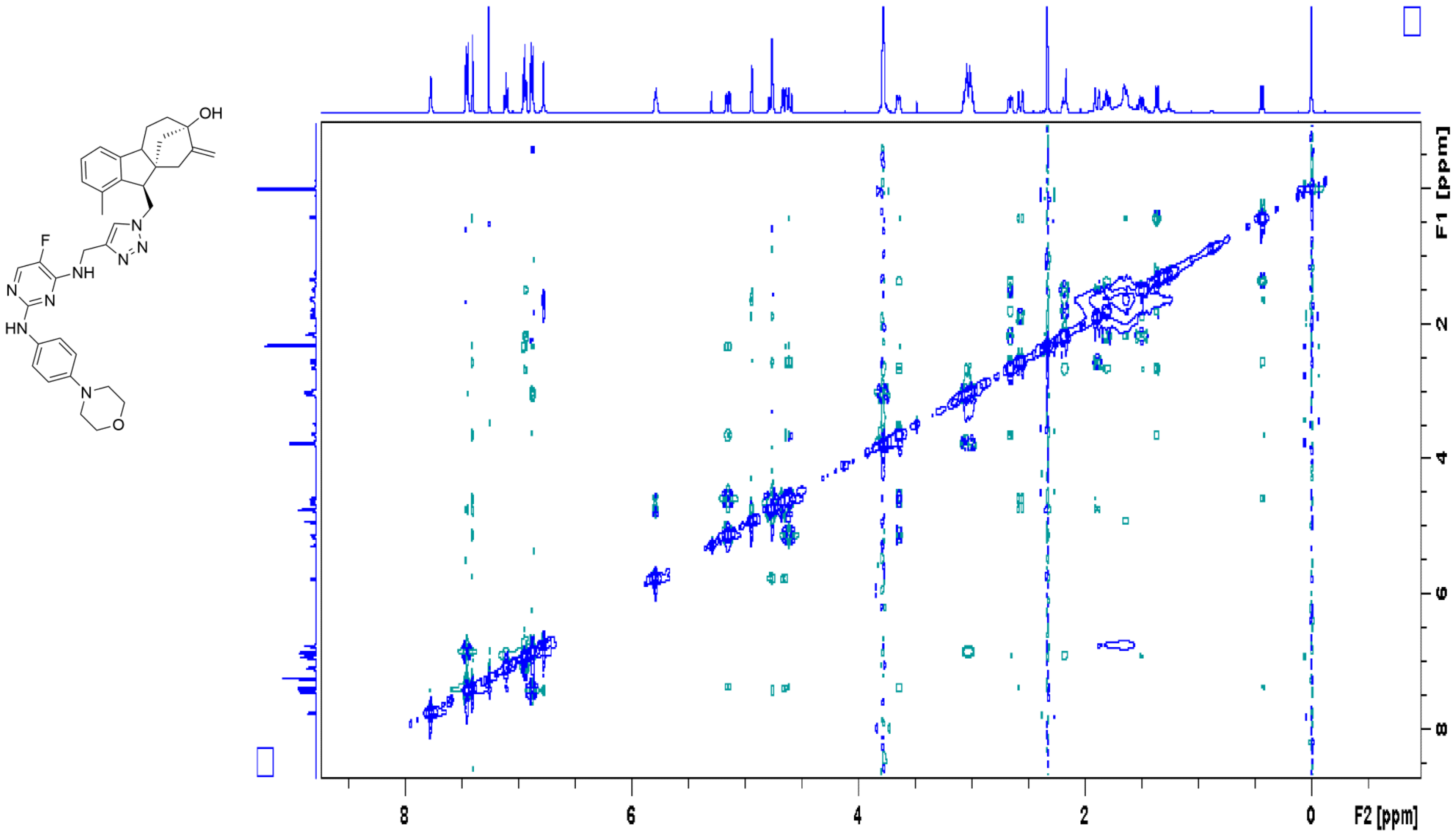

Figure S73. HSQC-NMR of compound **25**

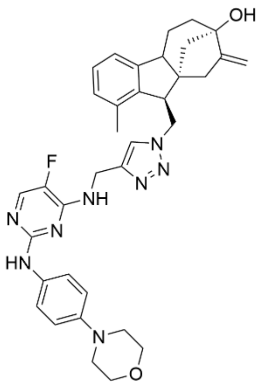

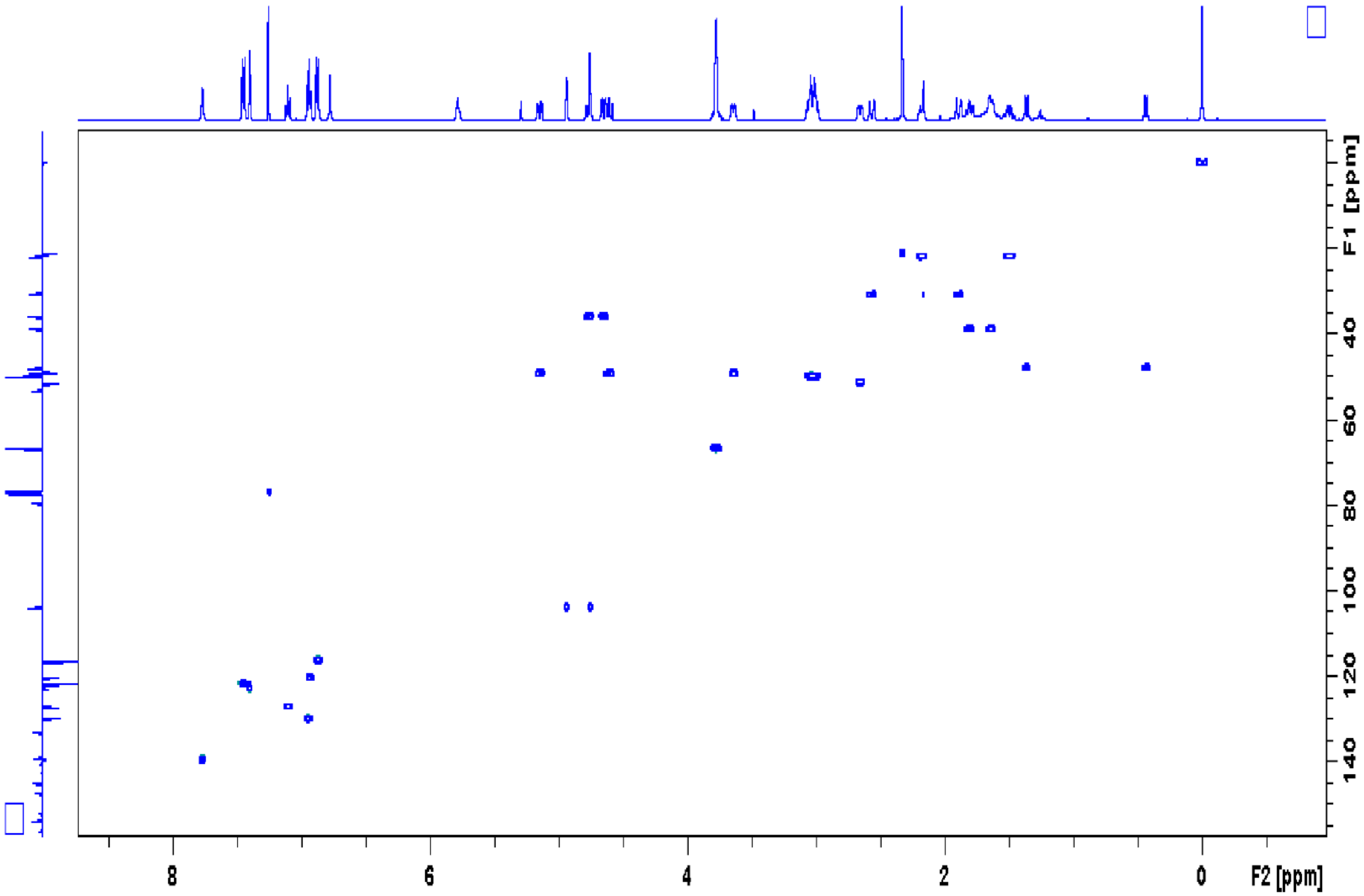

Figure S74. HMBC-NMR of compound **25**

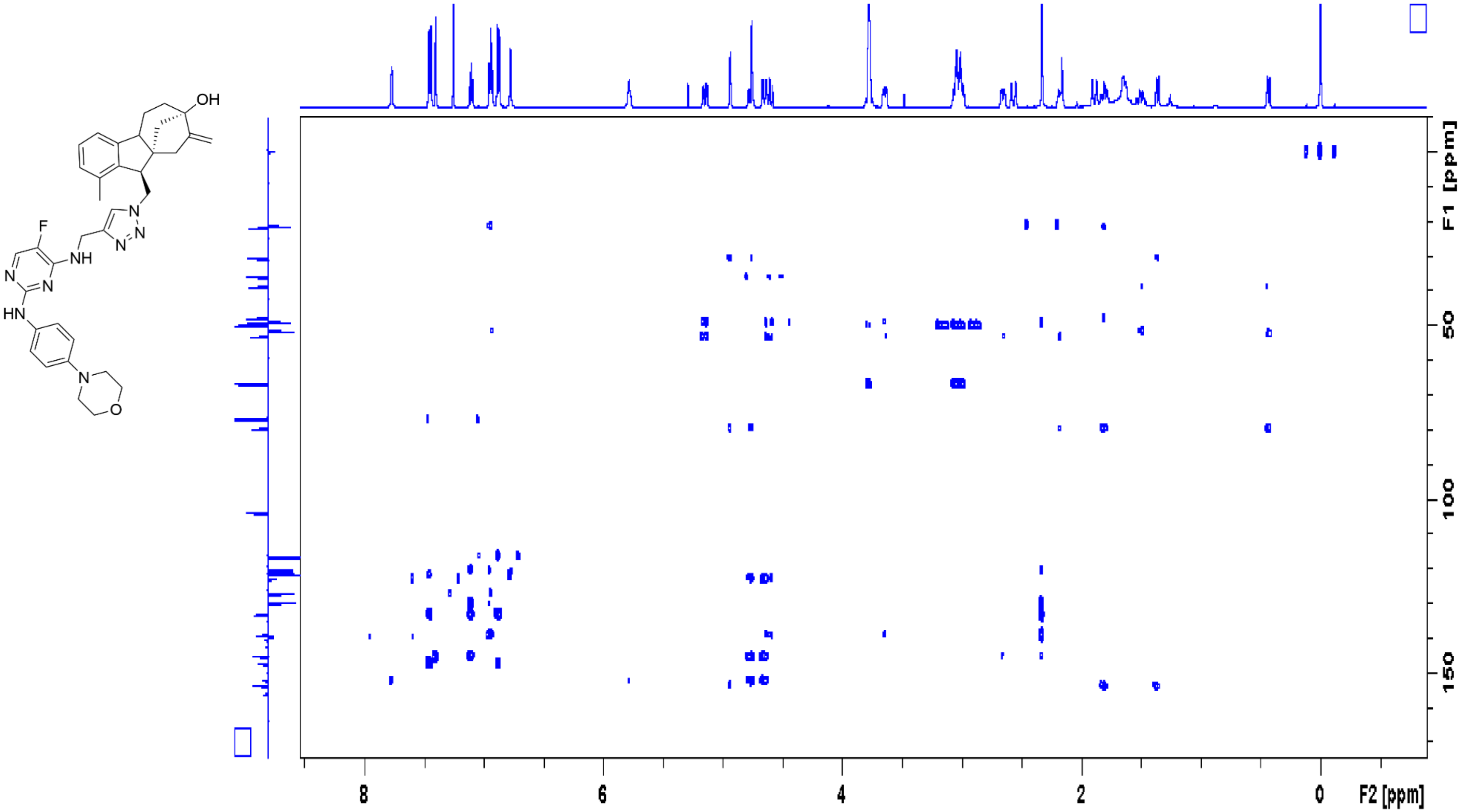

(7S,8S,9aR,10R)-10-((4-(((2-Chloro-5-fluoropyrimidin-4-yl)amino)methyl)-1H-1,2,3-triazol-1-yl)methyl)-8-(hydroxymethyl)-1-methyl-4b,5,6,8,9,10-hexahydro-7H-7,9a-methanobenzo[a]azulene-7,8-diol (**26**)

Figure S75. ^{1}H-NMR of compound **26**

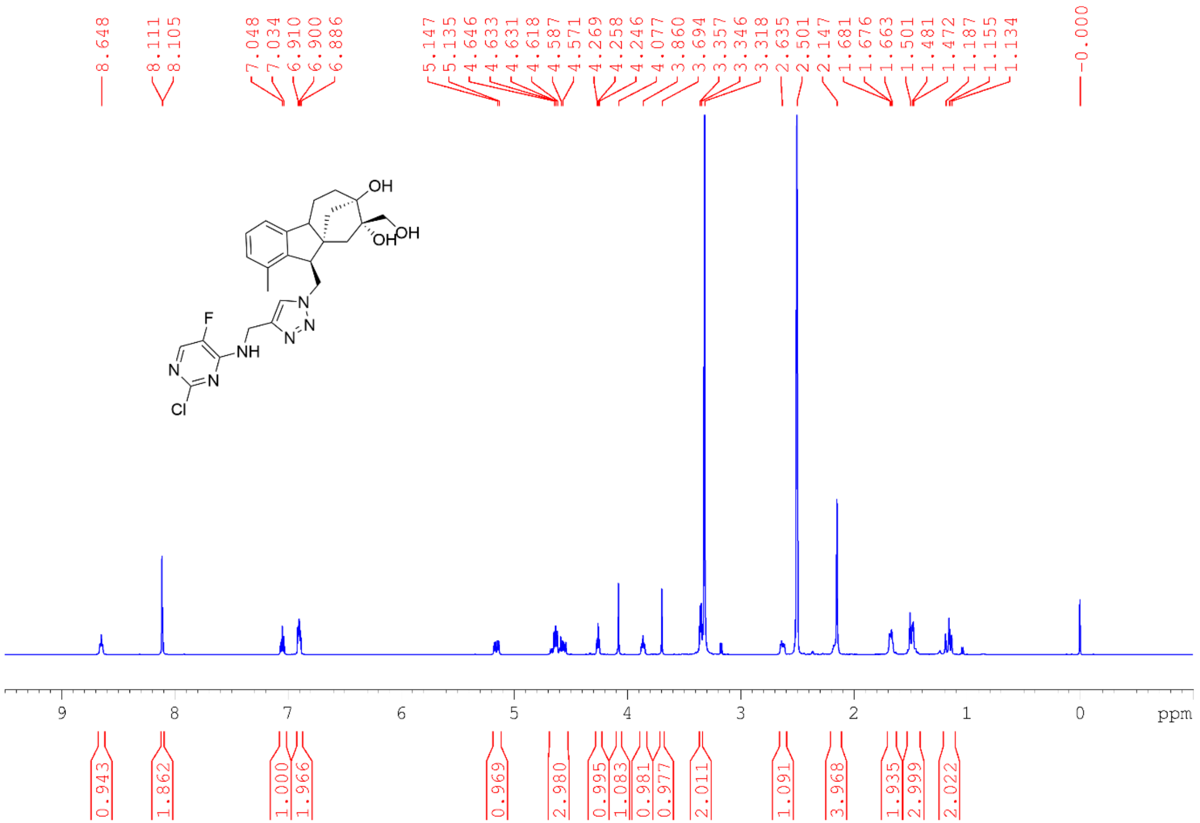

Figure S76. ^{13}C-NMR of compound **26**

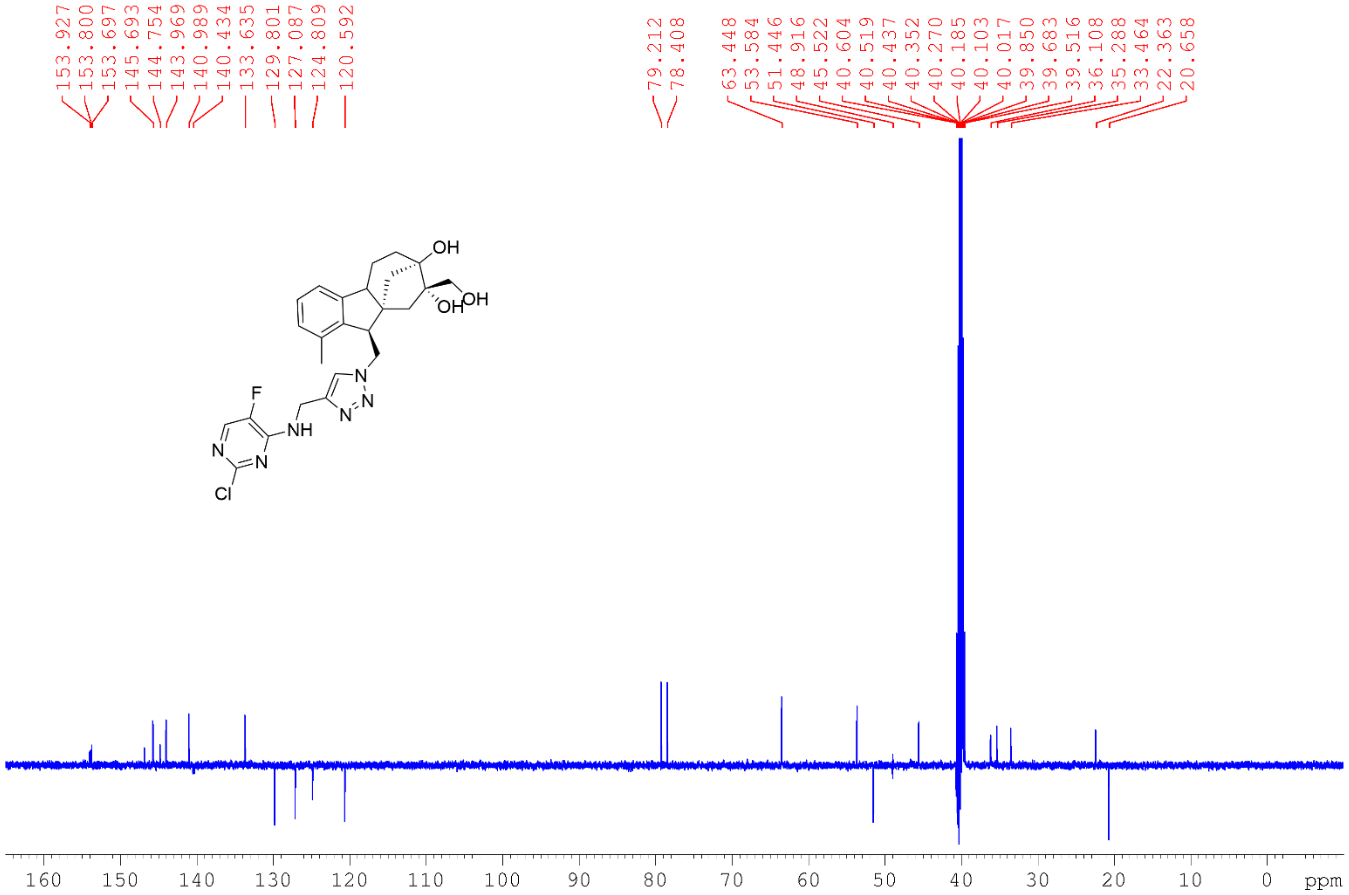

Figure S77. ^{19}F-NMR of compound **26**

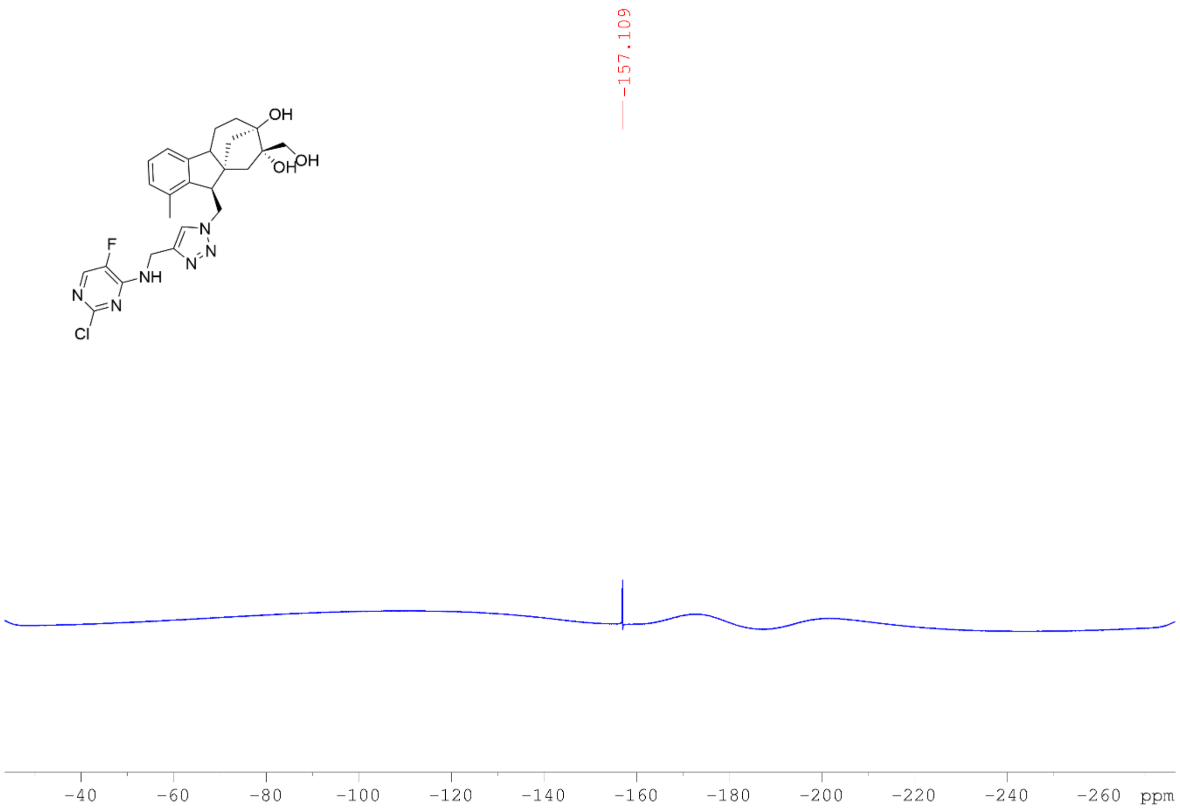

Figure S78. COSY-NMR of compound **26**

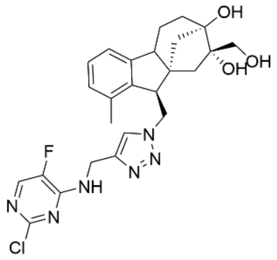

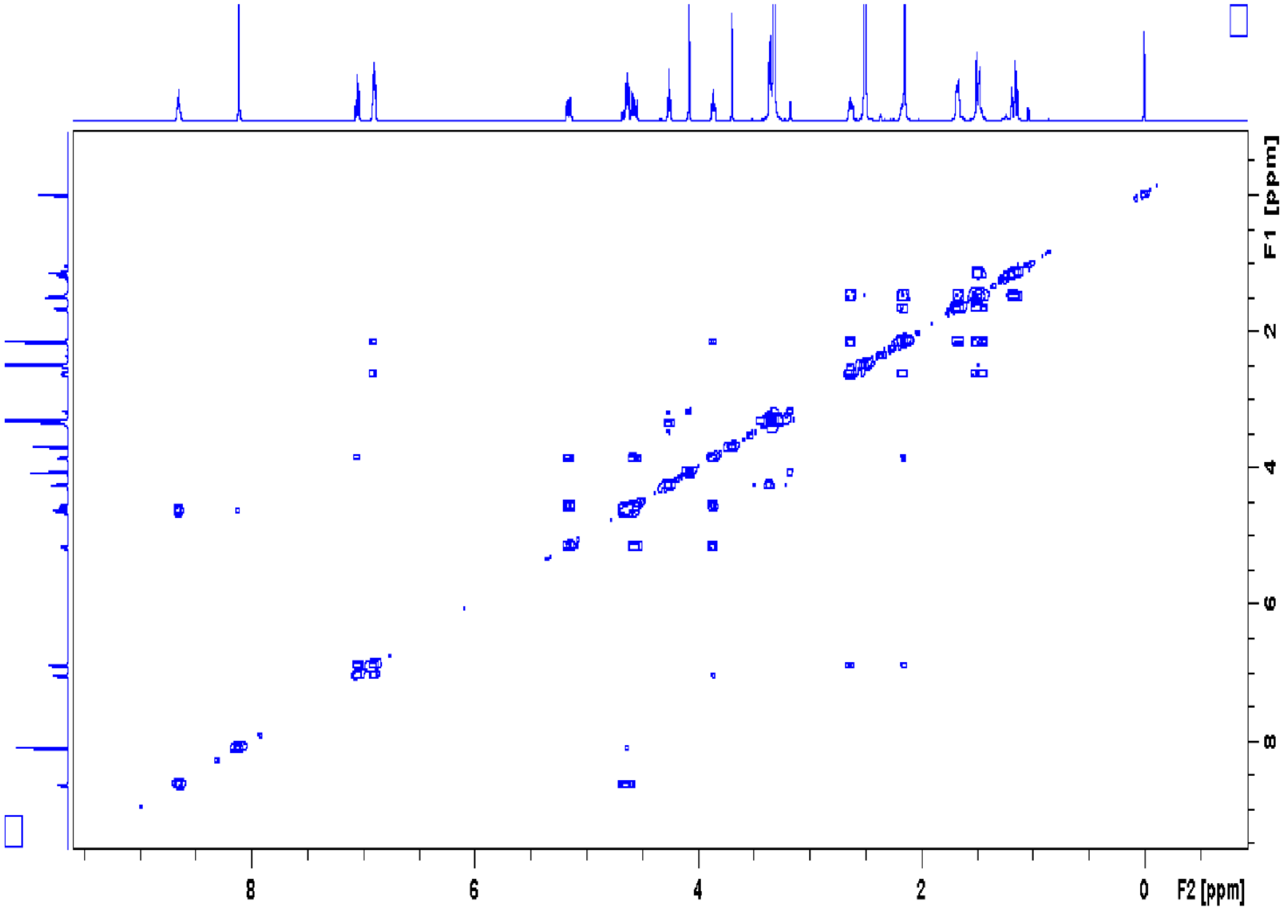

Figure S79. NOESY-NMR of compound **26**

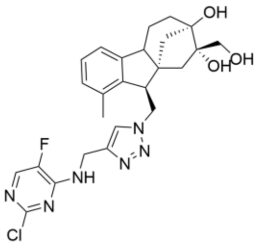

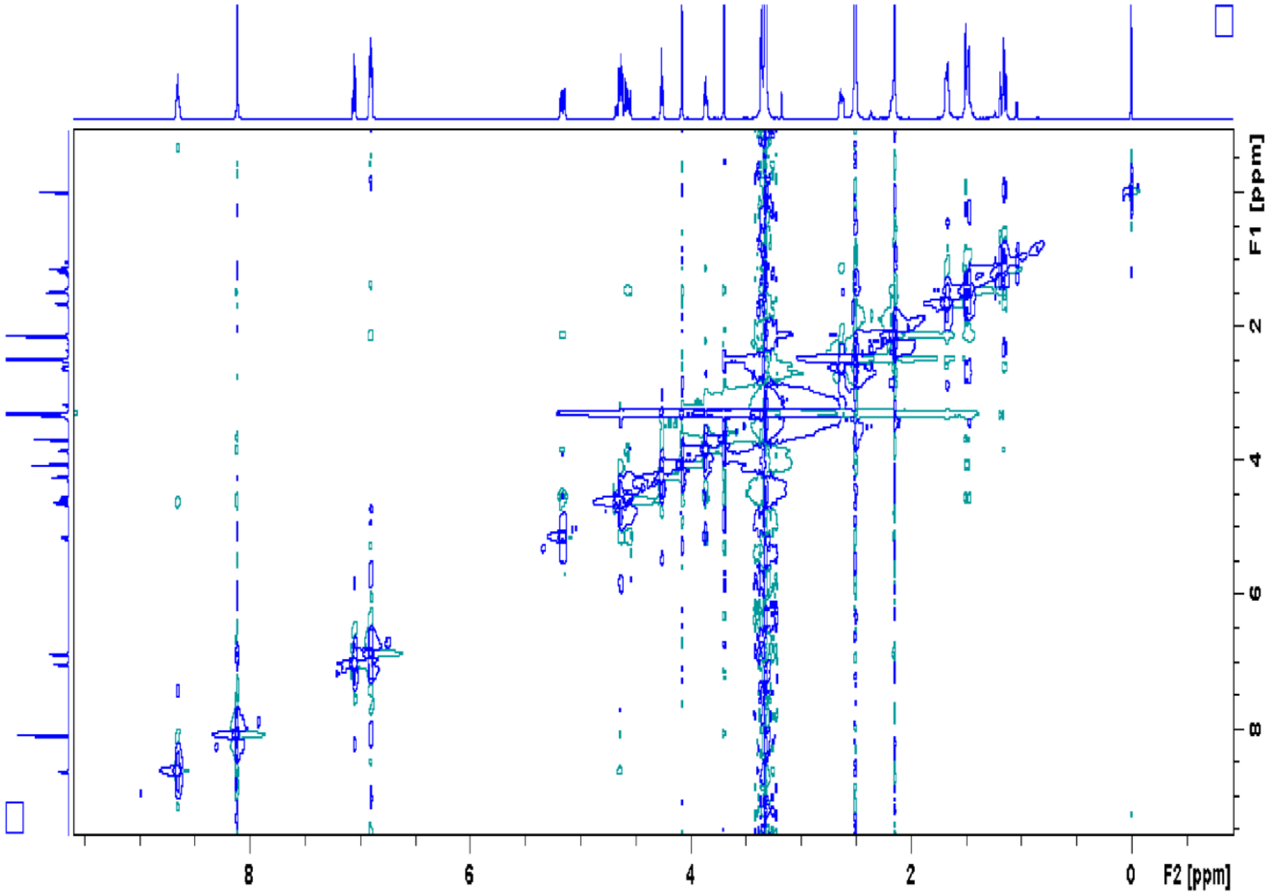

Figure S80. HSQC-NMR of compound **26**

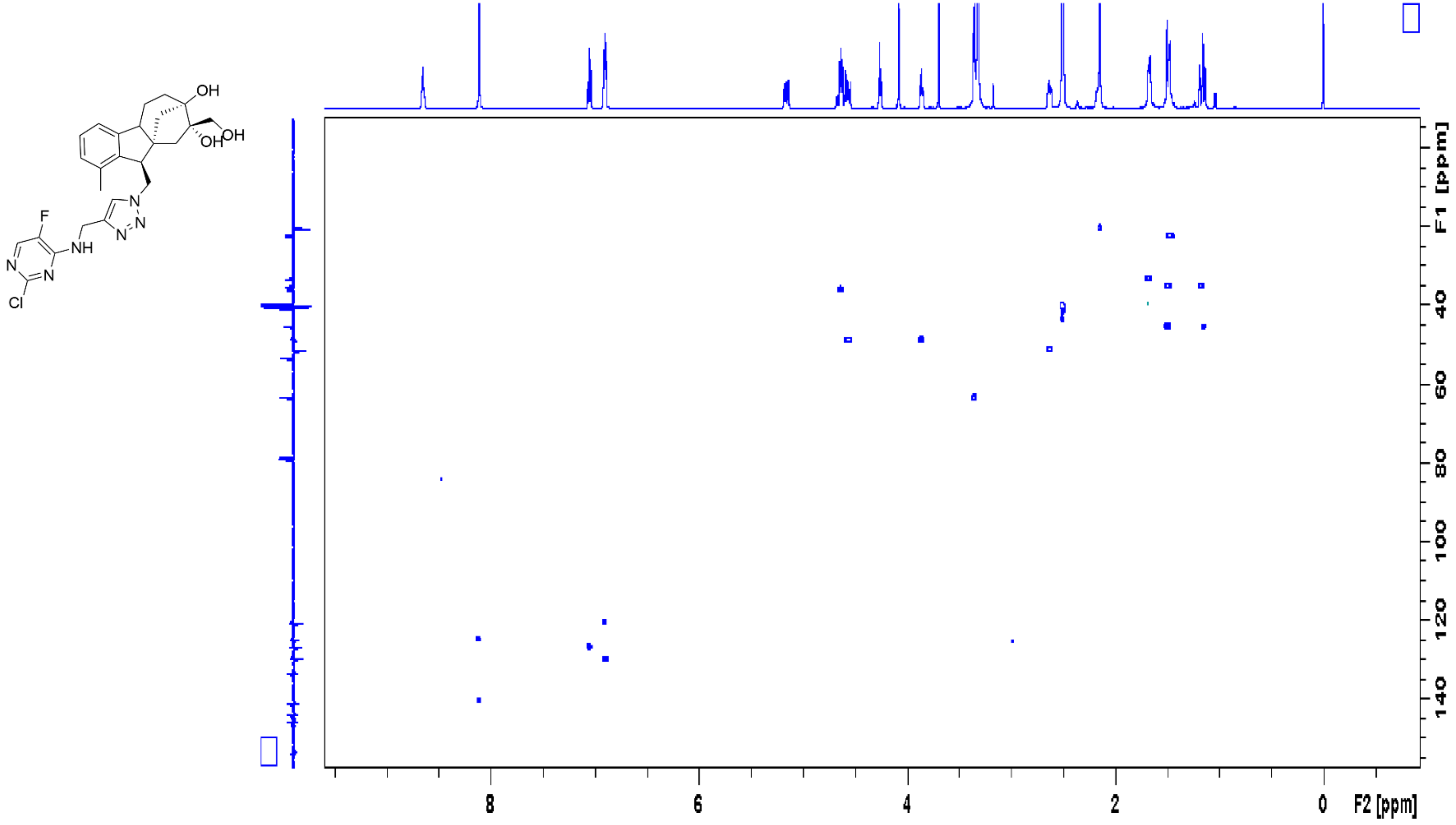

Figure S81. HMBC-NMR of compound **26**

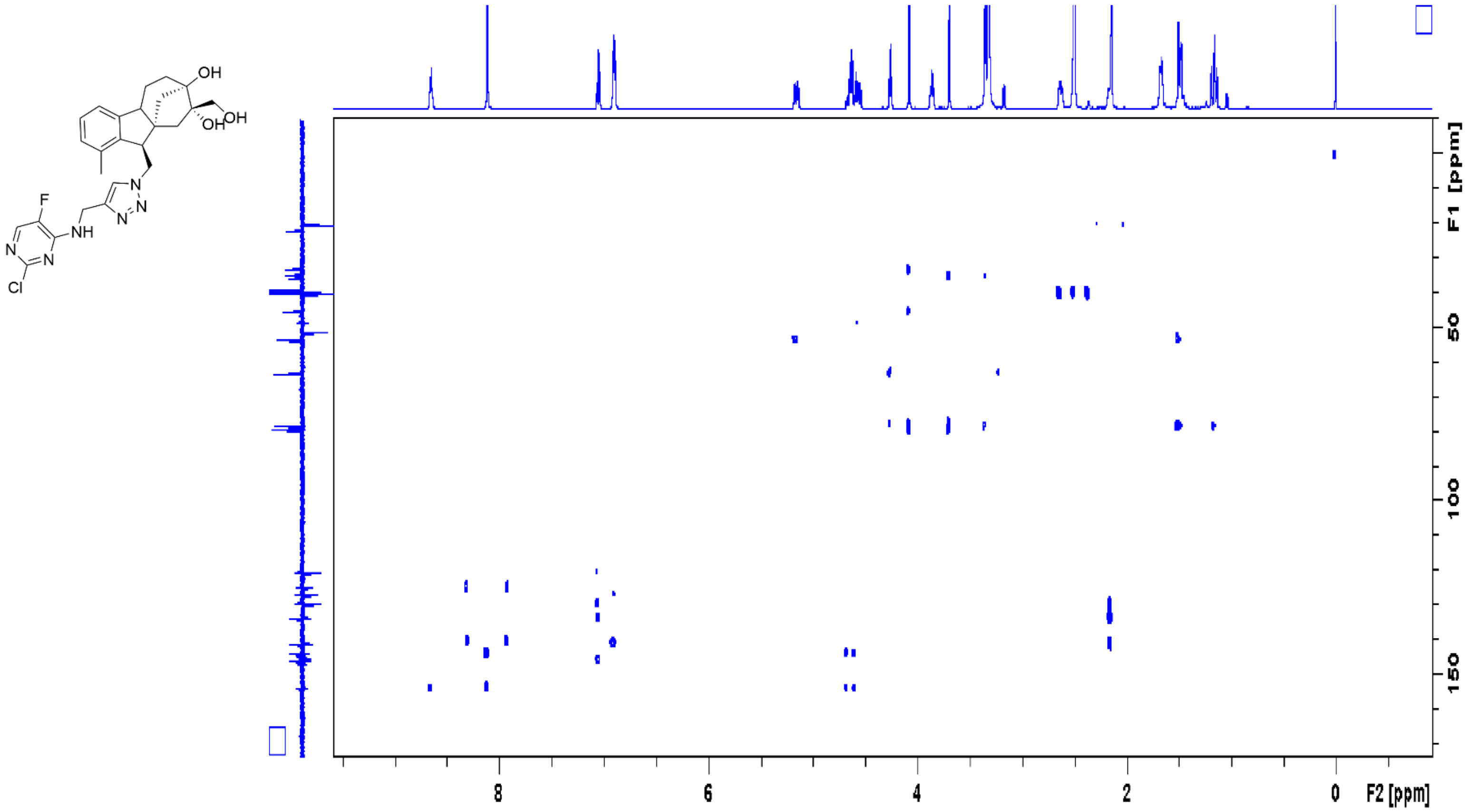

(7S,8S,9aR,10R)-10-((4-(((2,5-Dichloropyrimidin-4-yl)amino)methyl)-1H-1,2,3-triazol-1-yl)me-thyl)-8-(hydroxymethyl)-1-methyl-4b,5,6,8,9,10-hexahydro-7H-7,9a-methanobenzo[a]azulene-7,8-diol (**27**)

Figure S82. ^{1}H-NMR of compound **27**

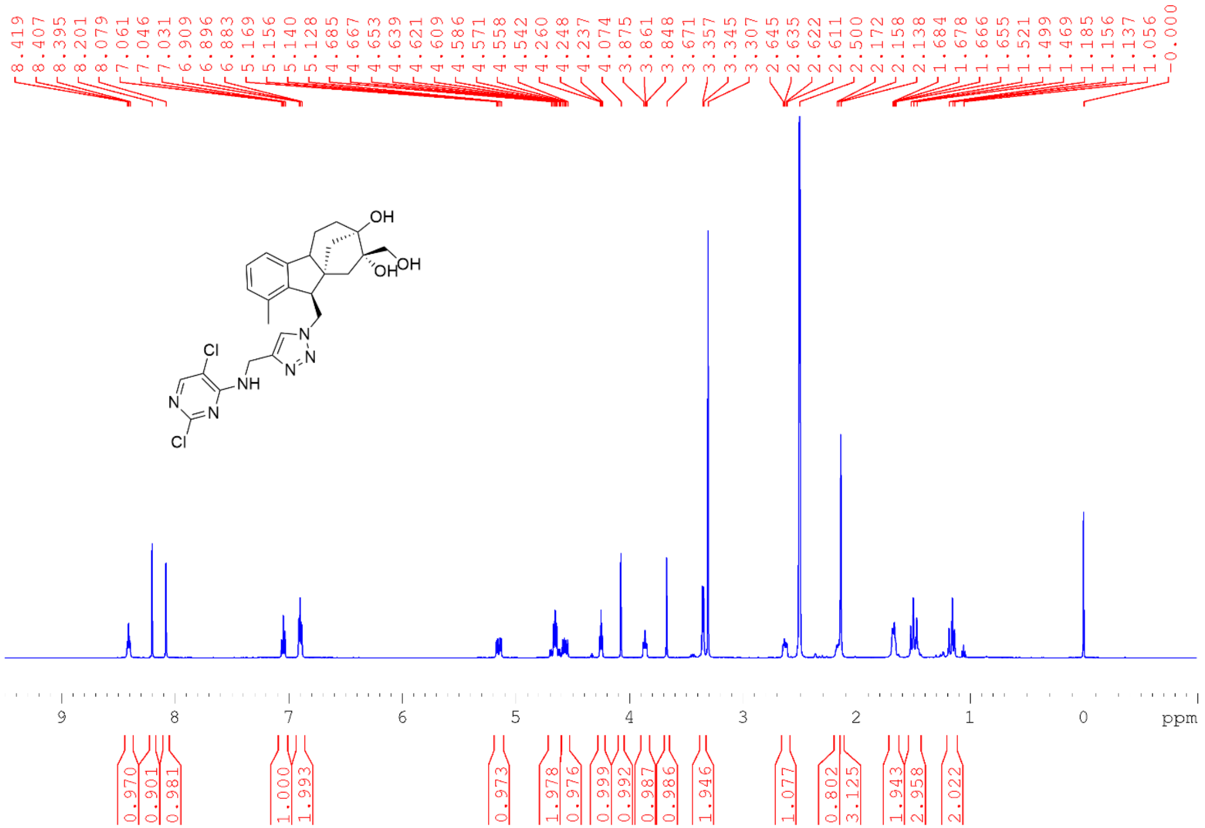

Figure S83. ^{13}C-NMR of compound **27**

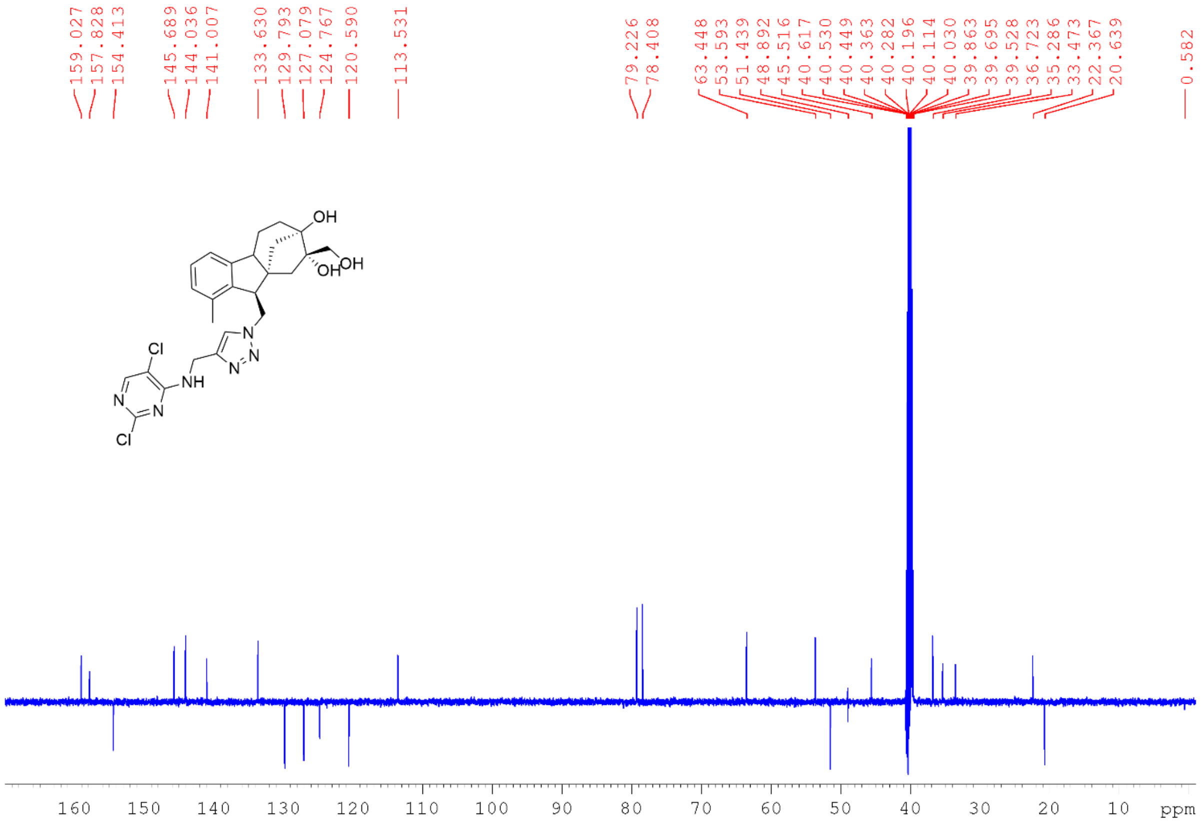

Figure S84. COSY-NMR of compound **27**

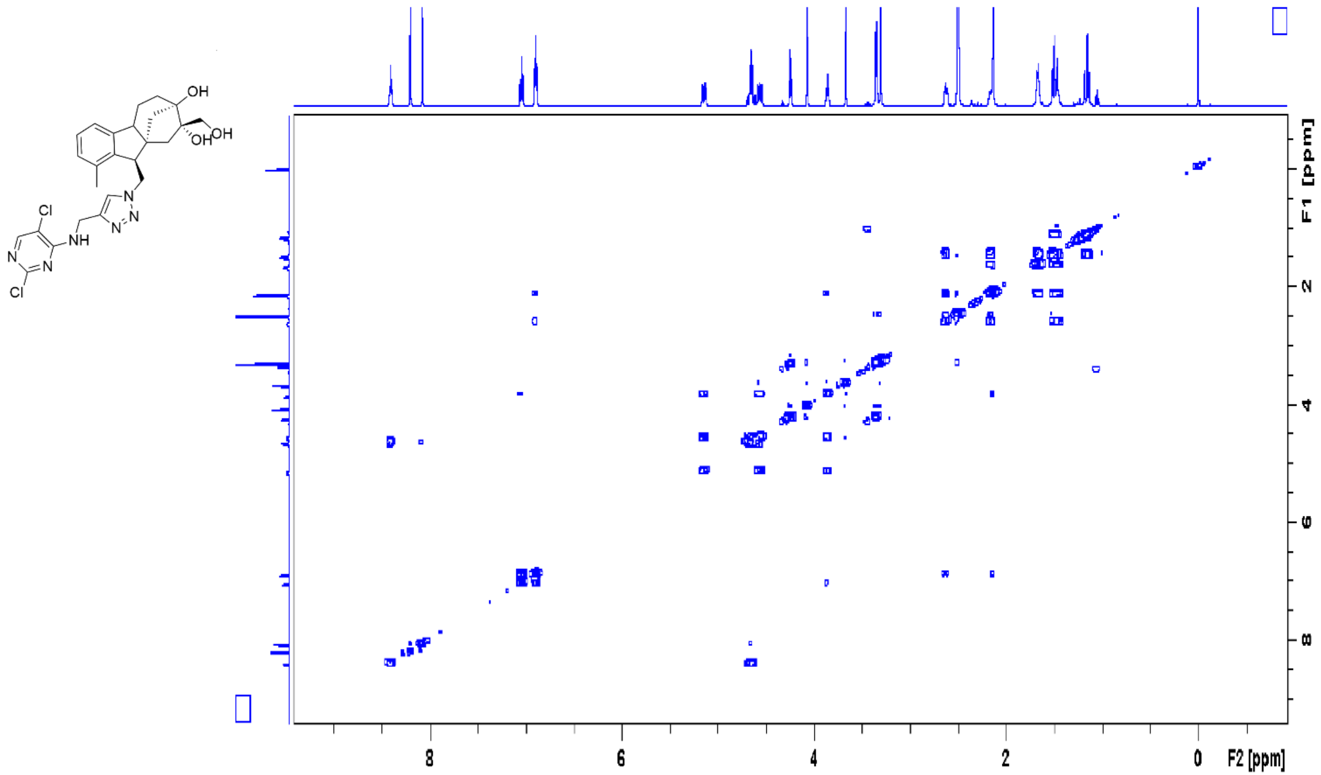

Figure S85. NOESY-NMR of compound **27**

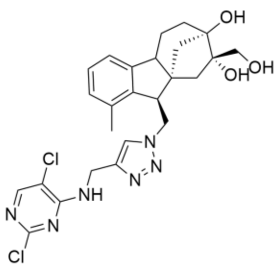

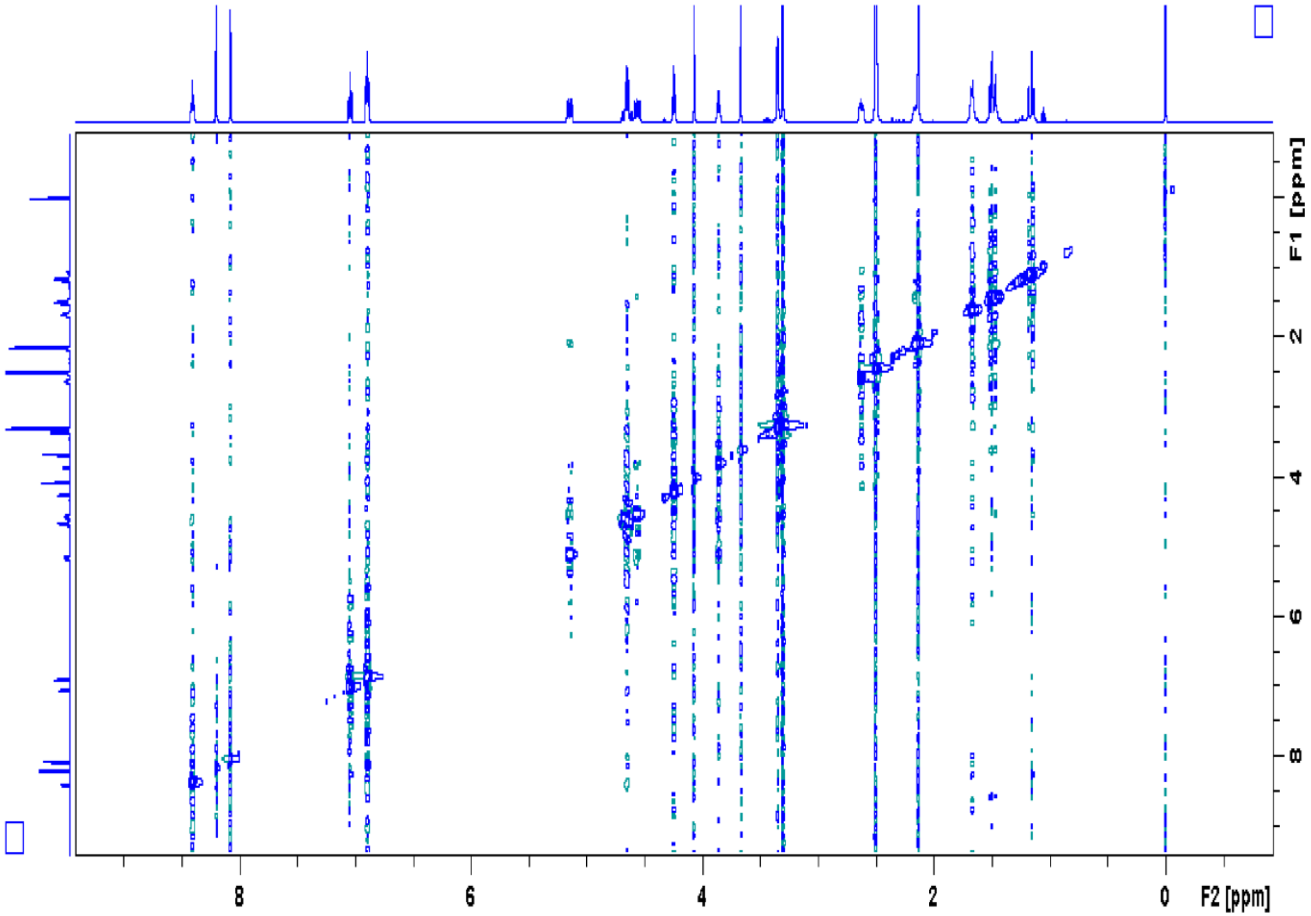

Figure S86. HSQC-NMR of compound **27**

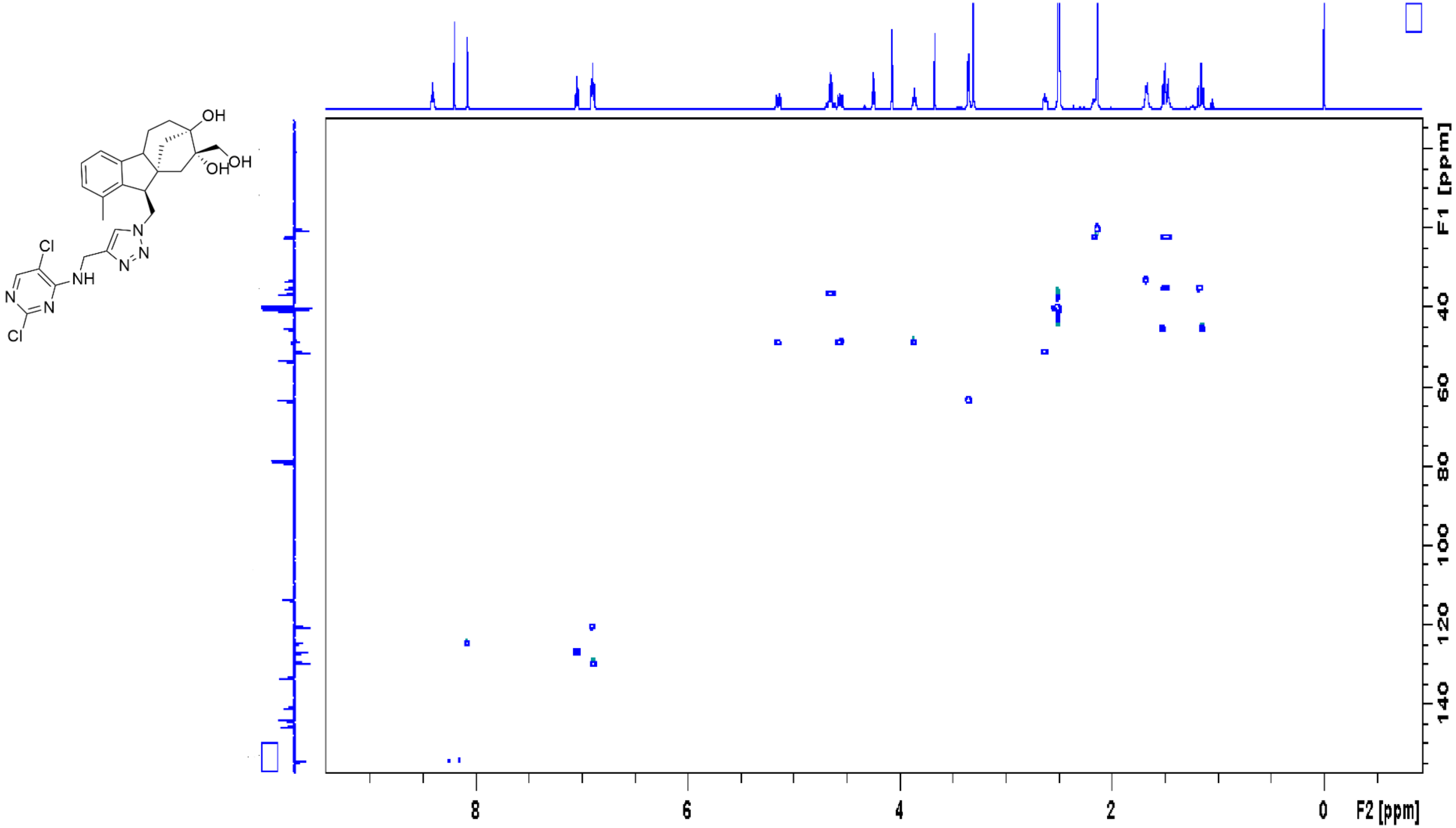

Figure S87. HMBC-NMR of compound **27**

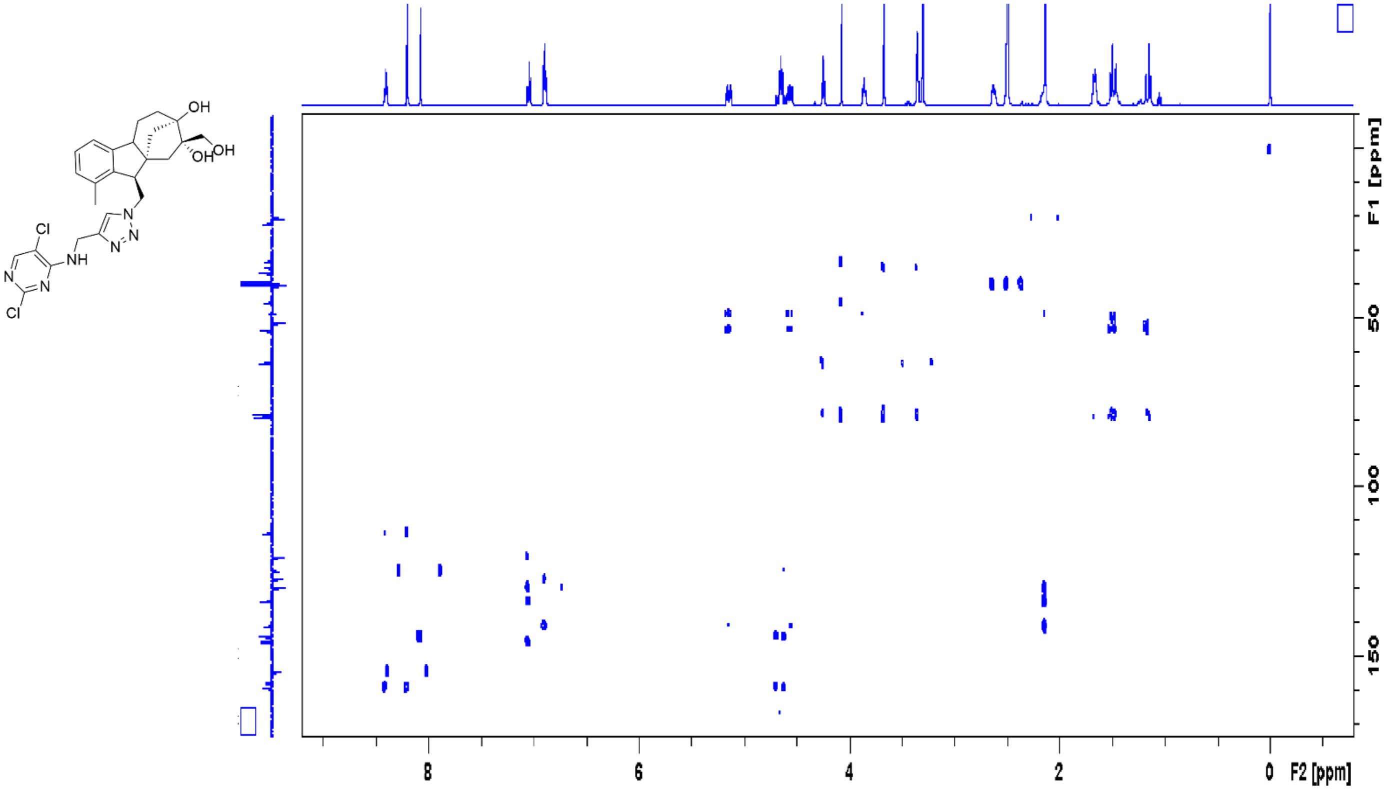

(7S,8S,9aR,10R)-10-((4-(((5-Fluoro-2-((4-(trifluoromethyl)phenyl)amino)pyrimidin-4-yl)amino)methyl)-1H-1,2,3-triazol-1-yl)methyl)-8-(hydroxymethyl)-1-methyl-4b,5,6,8,9,10-hexahydro-7H-7,9a-methanobenzo[a]azulene-7,8-diol (**28**)

Figure S88. ^{1}H-NMR of compound **28**

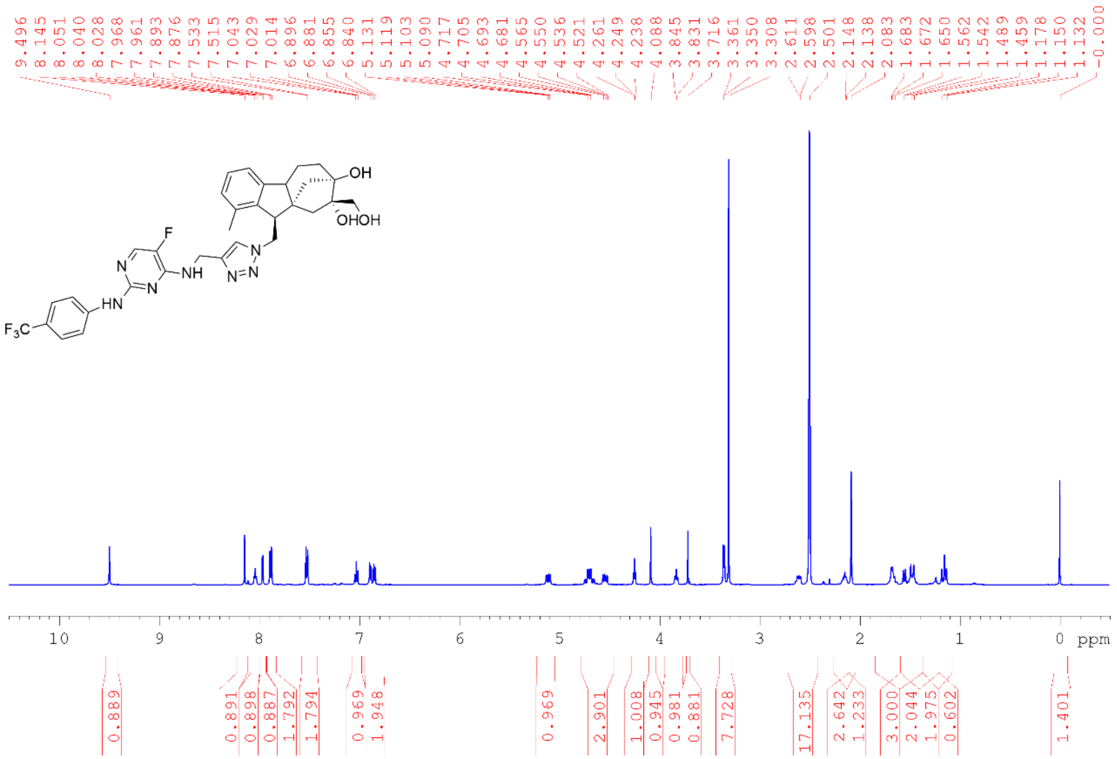

Figure S89. ^{13}C-NMR of compound **28**

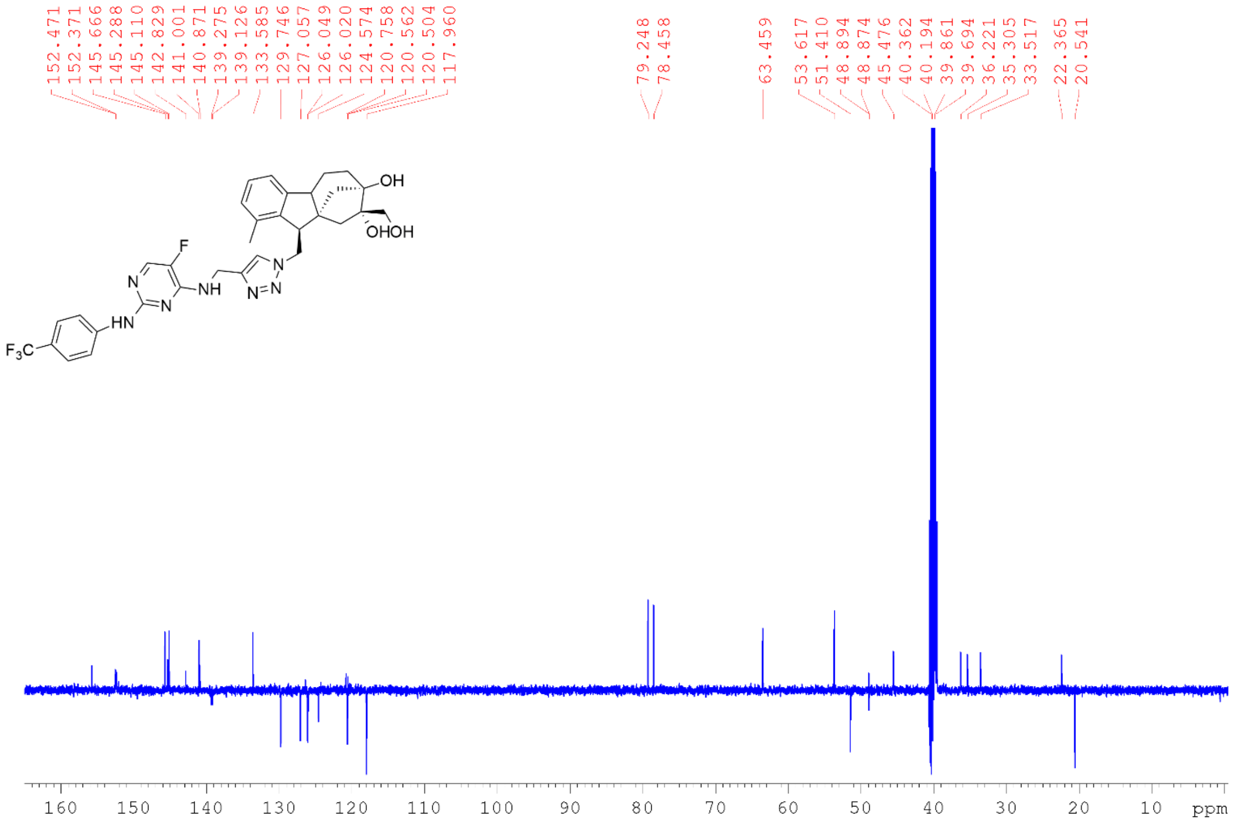

Figure S90. ^{19}F-NMR of compound **28**

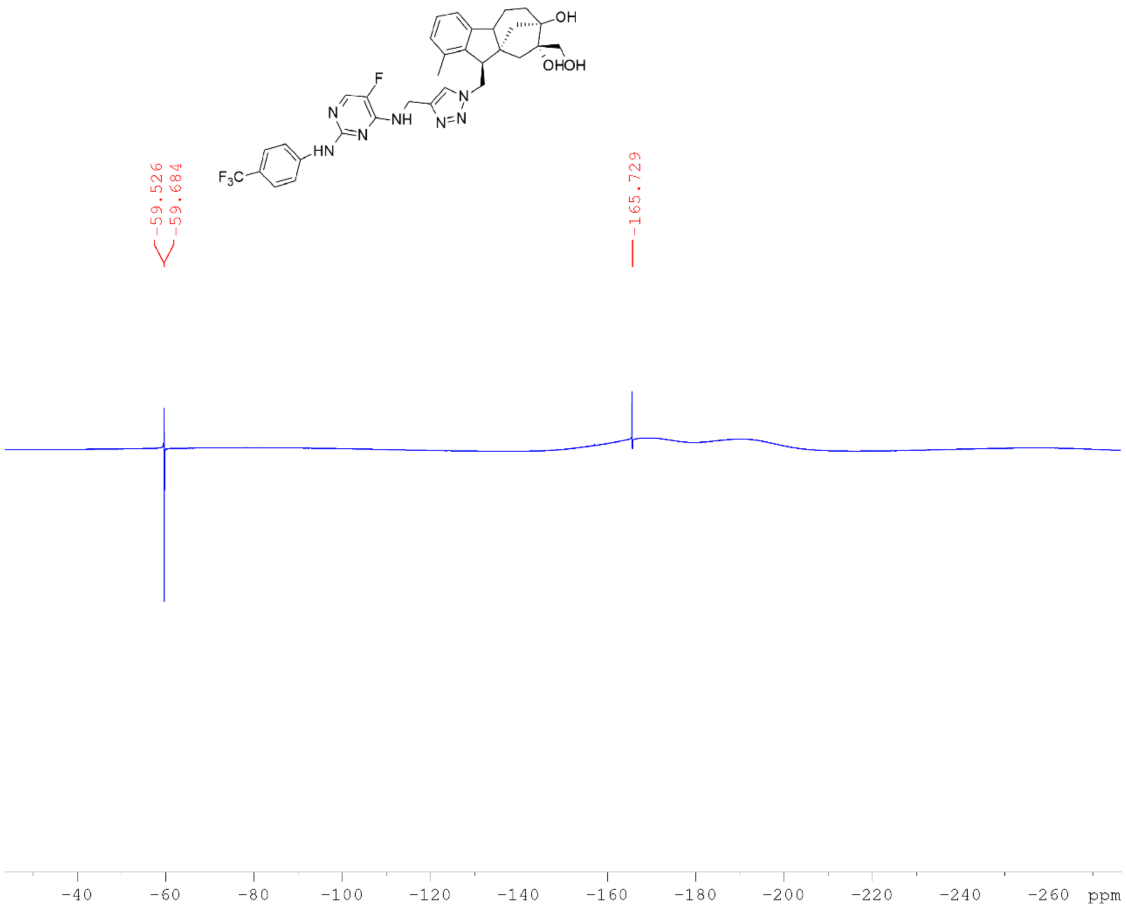

Figure S91. COSY-NMR of compound **28**

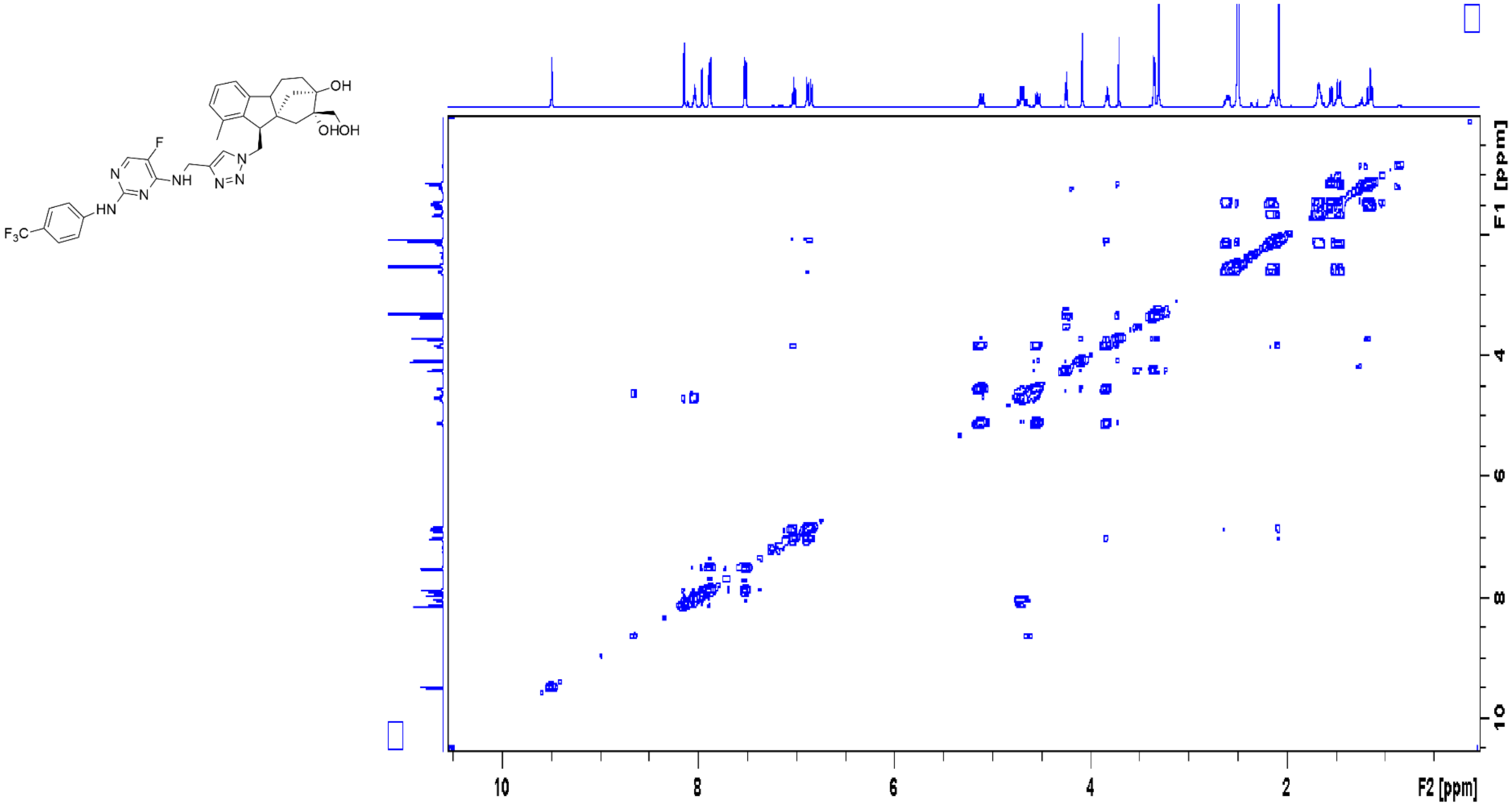

Figure S92. NOESY-NMR of compound **28**

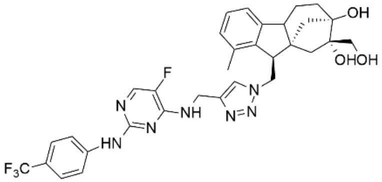

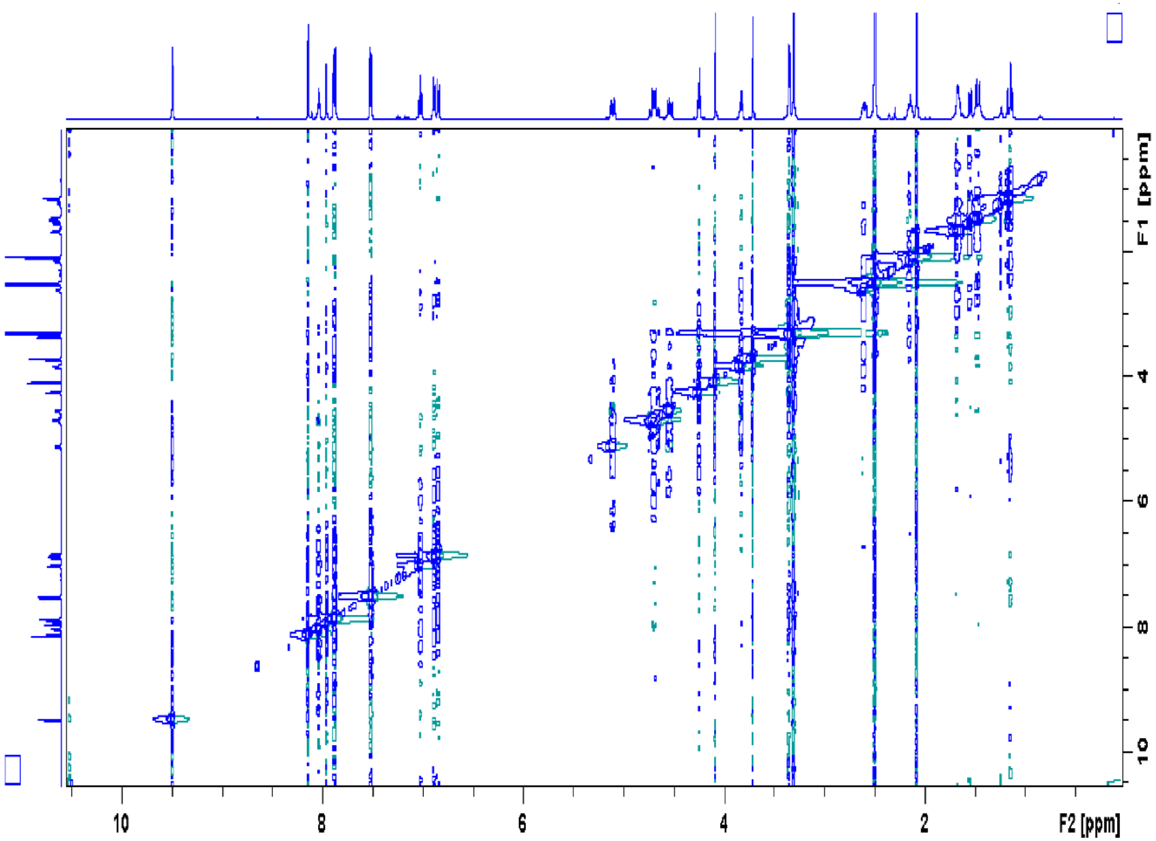

Figure S93. HSQC-NMR of compound **28**

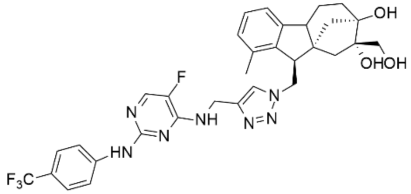

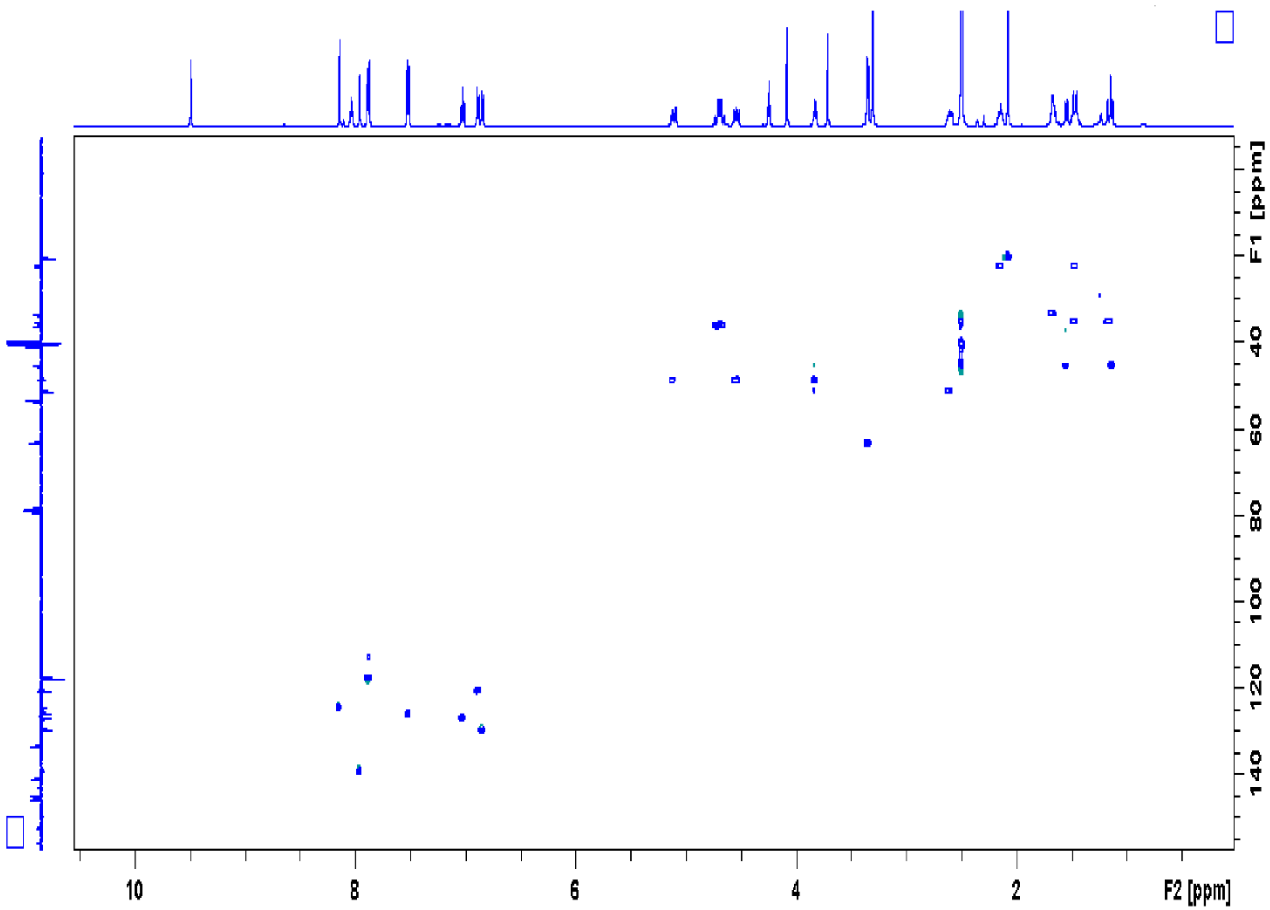

Figure S93. HMBC-NMR of compound **28**

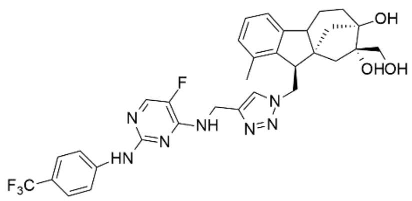

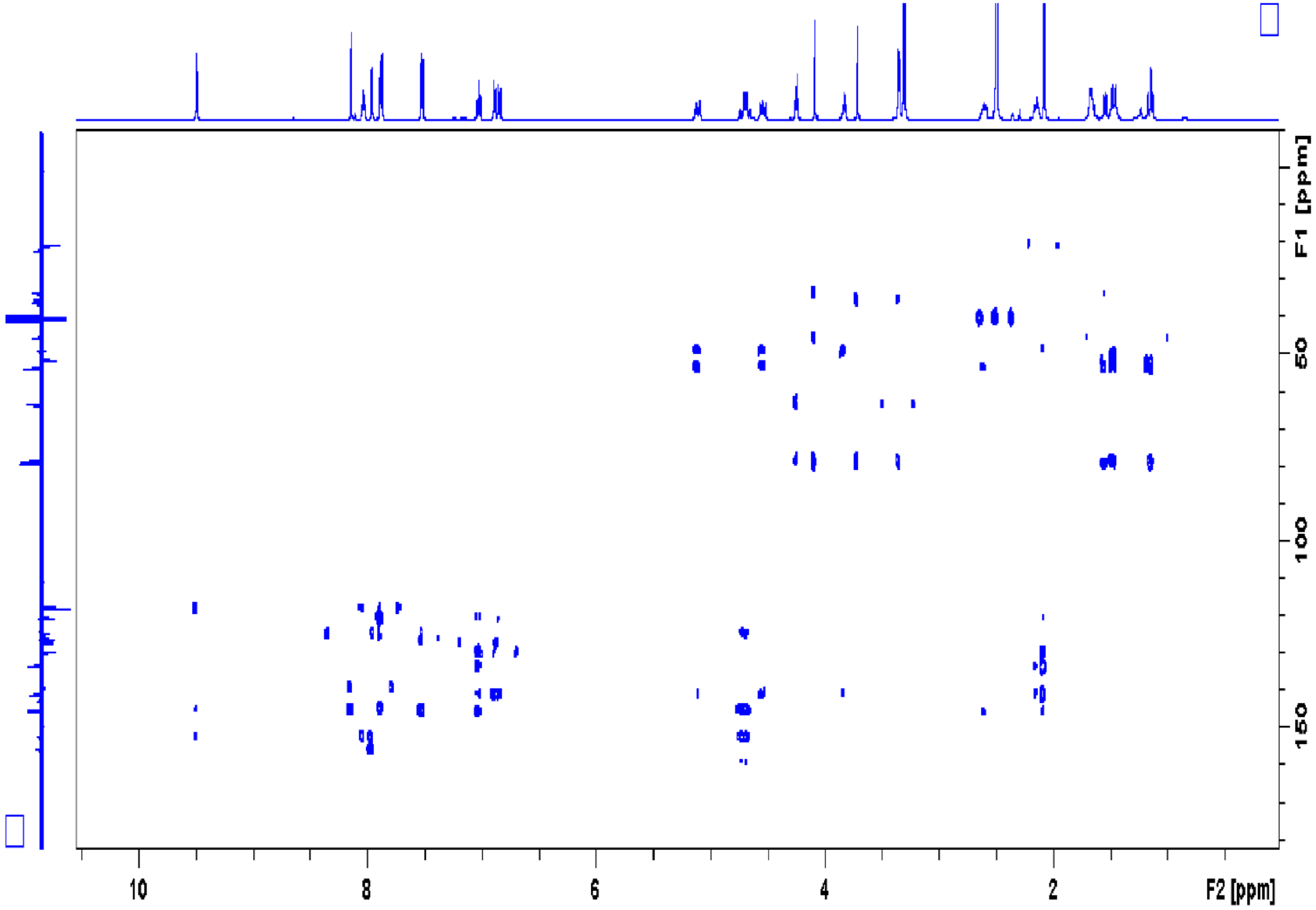

(7S,8S,9aR,10R)-10-((4-(((5-Chloro-2-((4-(trifluoromethyl)phenyl)amino)pyrimidin-4-yl)amino)methyl)-1H-1,2,3-triazol-1-yl)methyl)-8-(hydroxymethyl)-1-methyl-4b,5,6,8,9,10-hexahydro-7H-7,9a-methanobenzo[a]azulene-7,8-diol (**29**)

Figure S94. ^{1}H-NMR of compound **29**

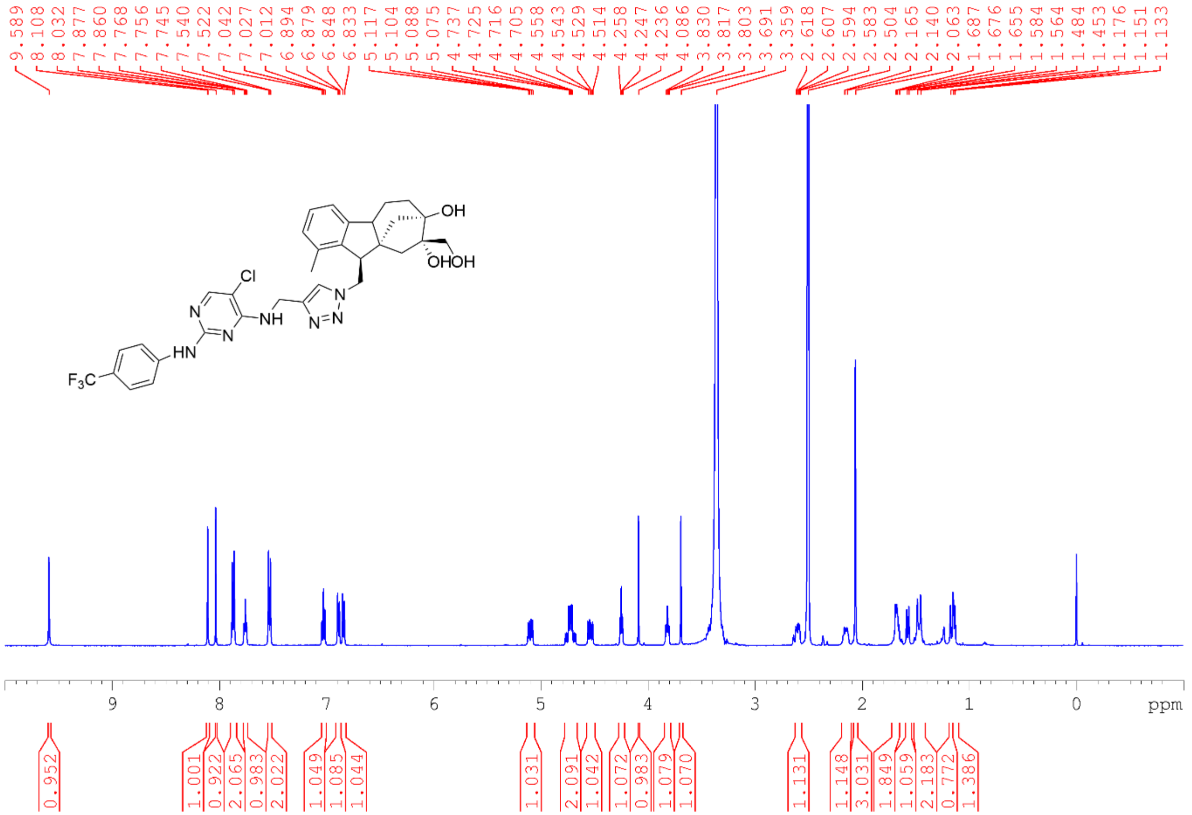

Figure S95. ^{13}C-NMR of compound **29**

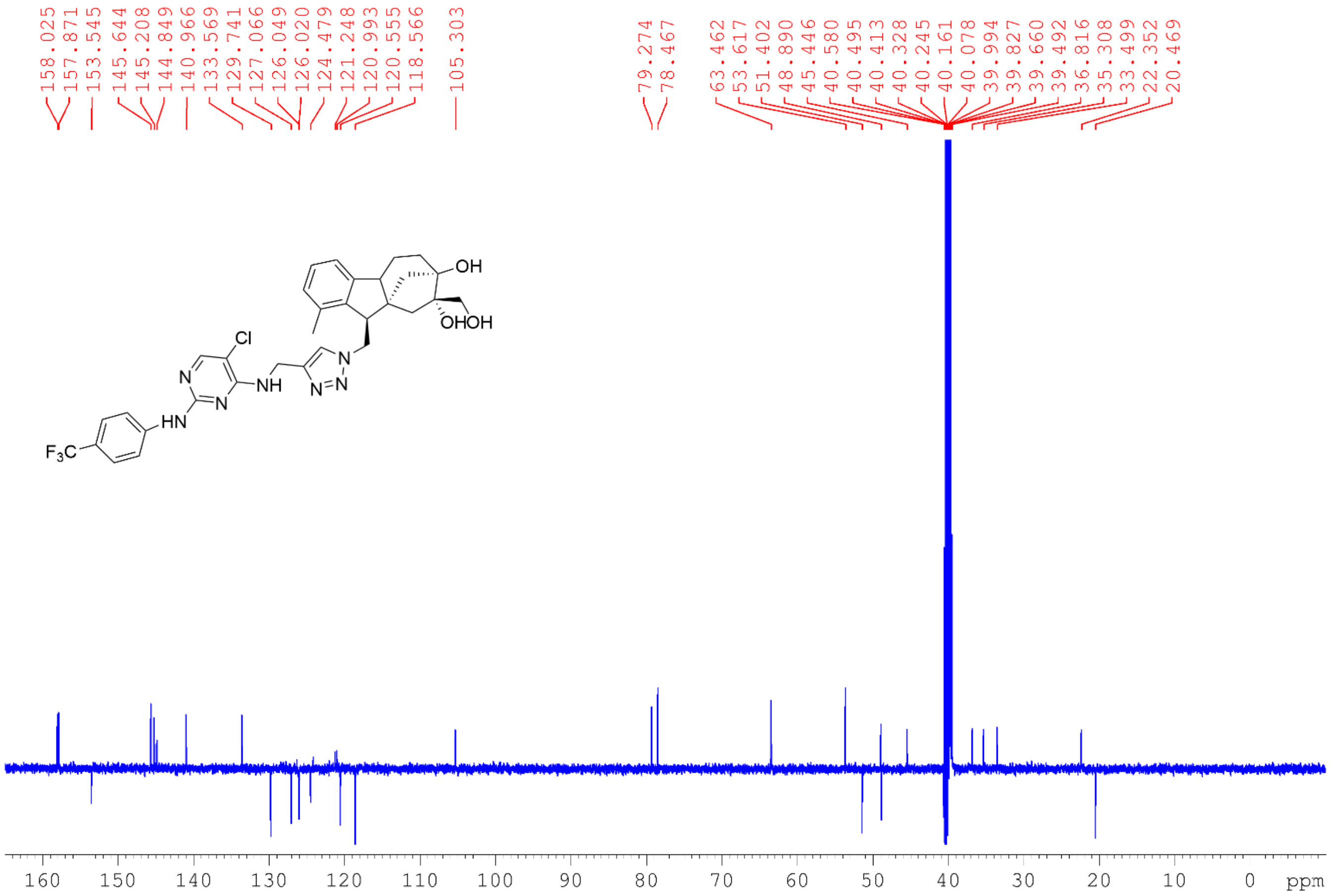

Figure S96. ^19^F-NMR of compound **29**

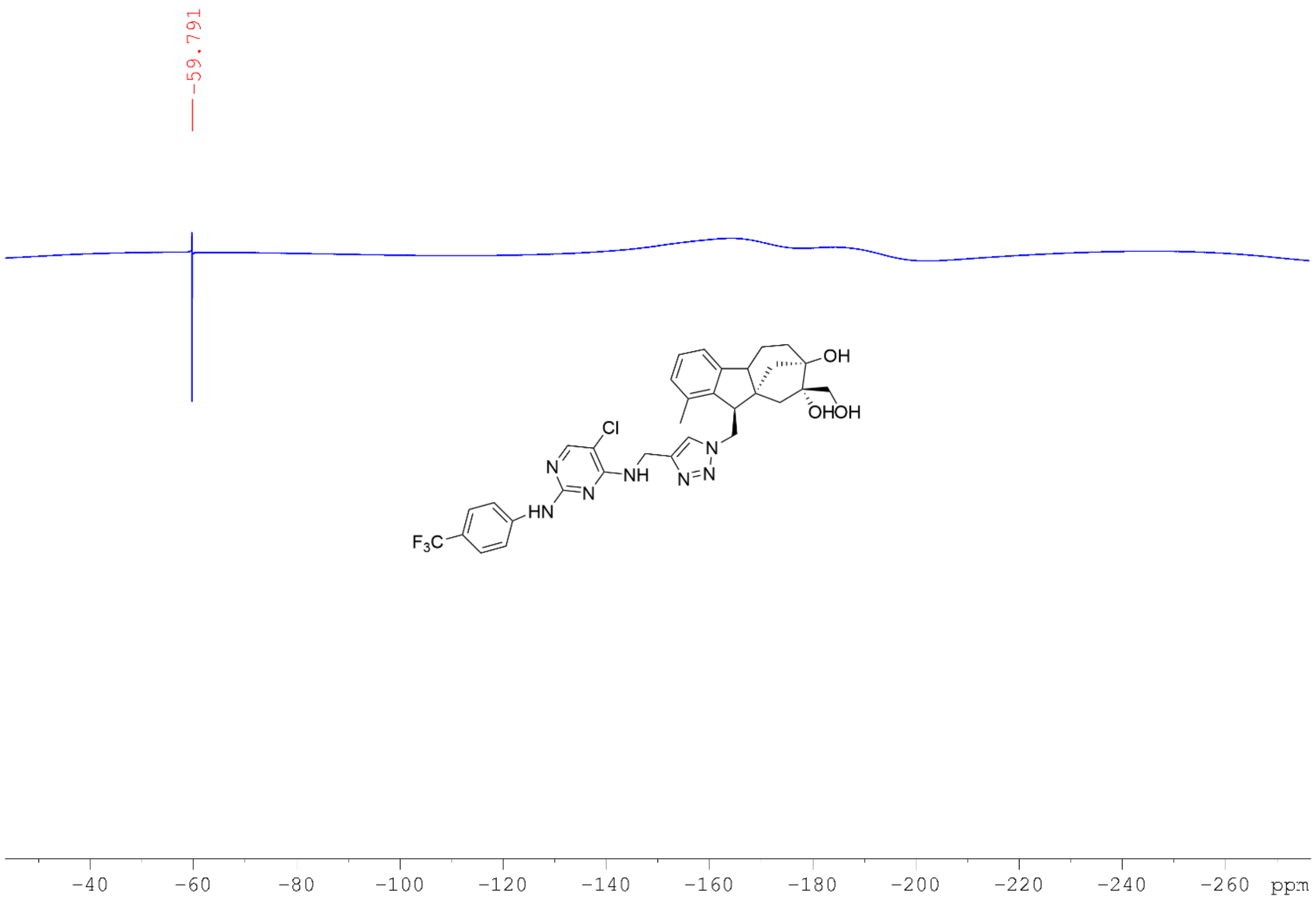

Figure S97. COSY-NMR of compound **29**

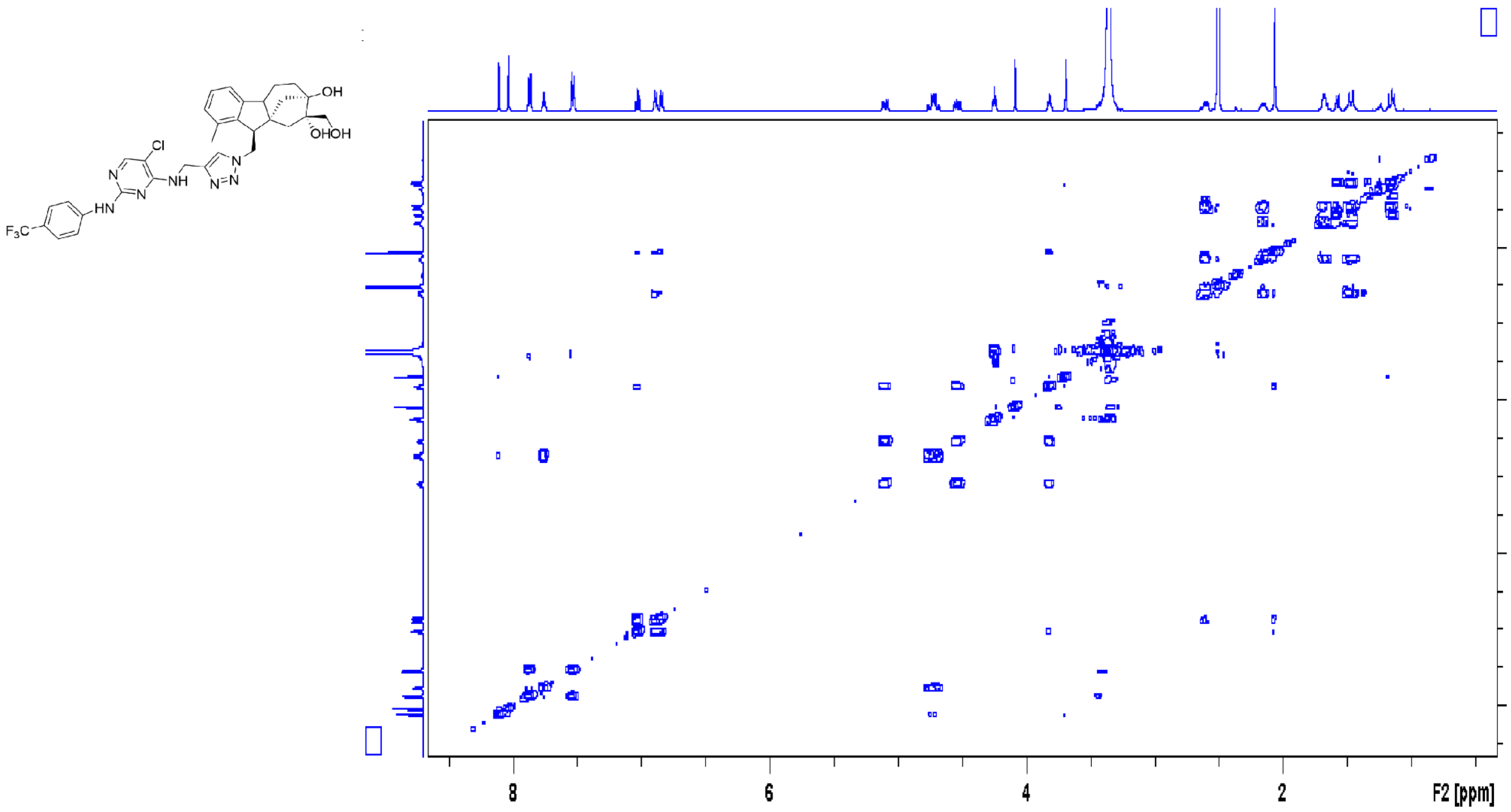

Figure S98. NOESY-NMR of compound **29**

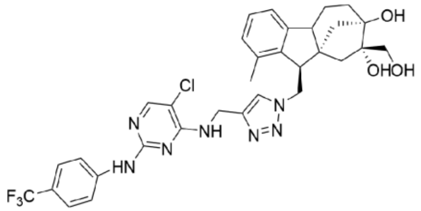

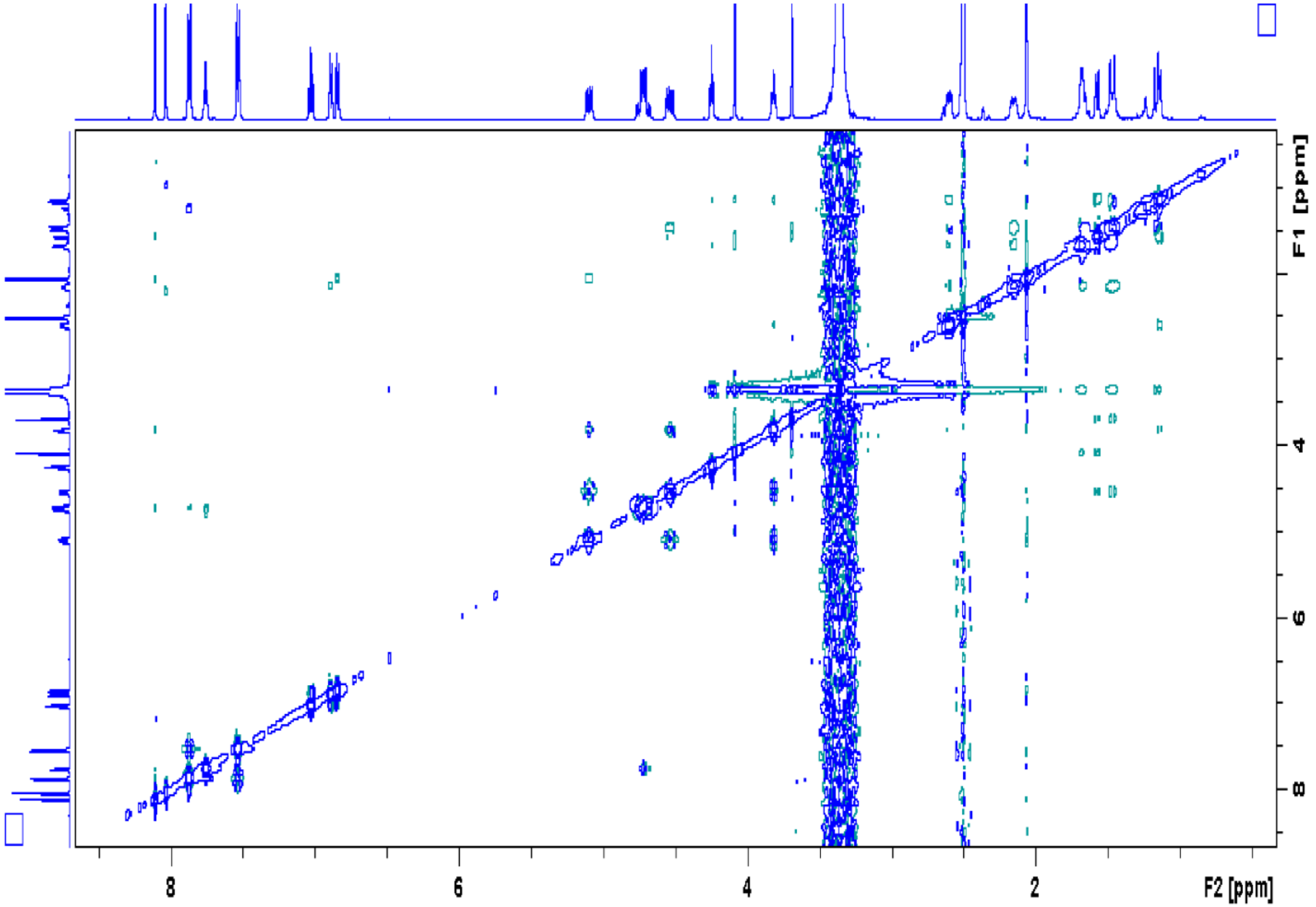

Figure S99. HSQC-NMR of compound **29**

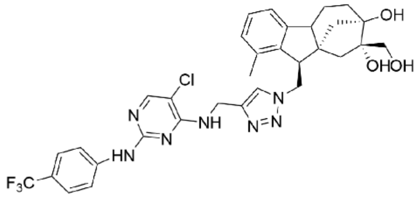

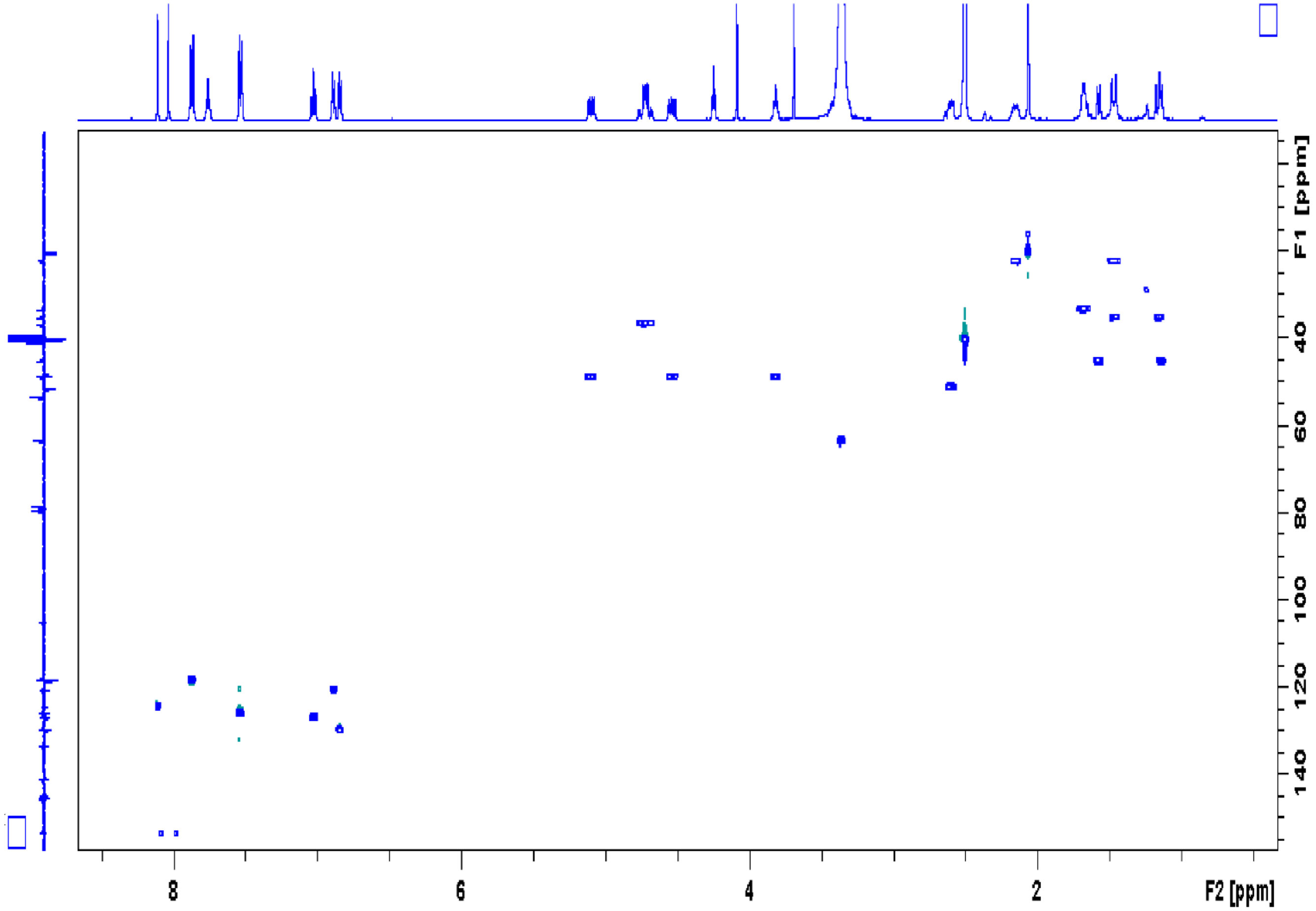

Figure S100. HMBC-NMR of compound **29**

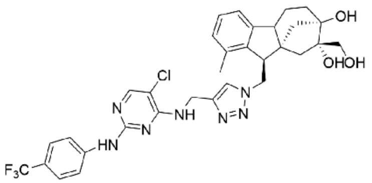

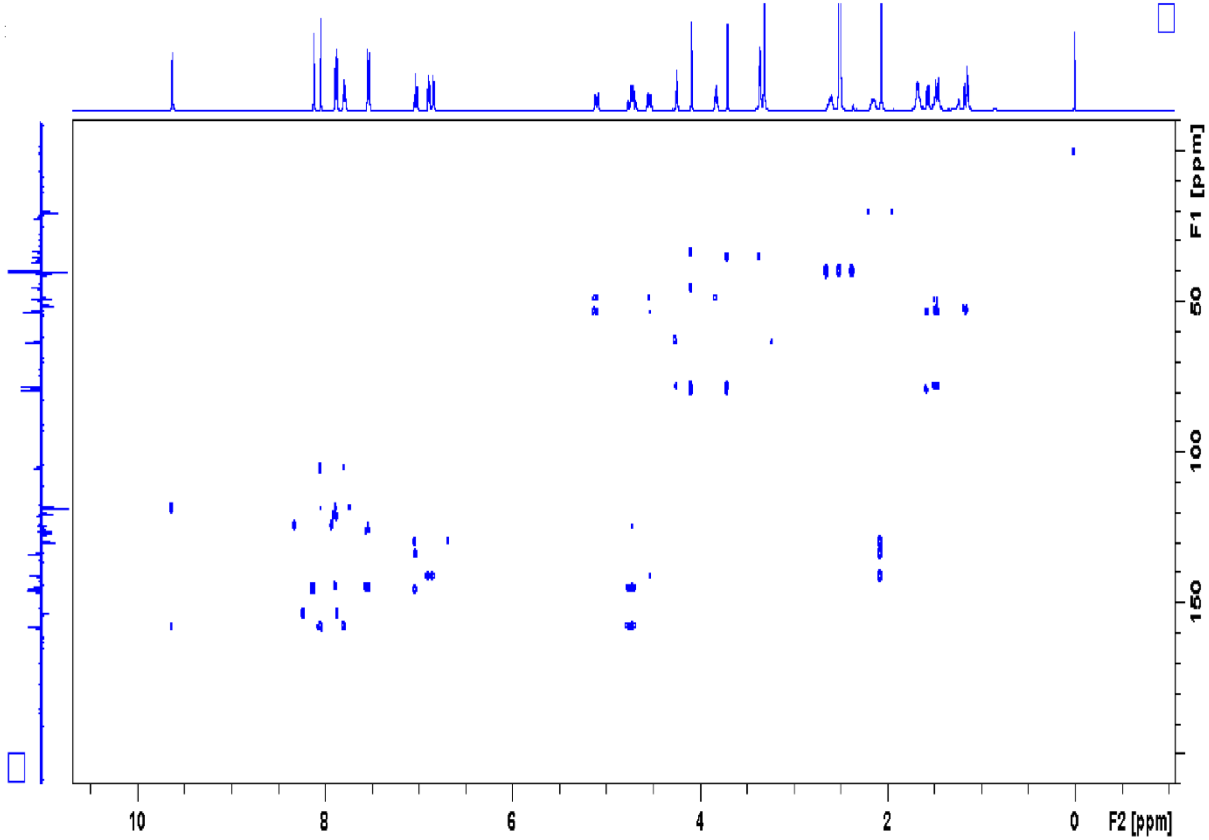

(7S,8S,9aR,10R)-10-((4-(((5-Fluoro-2-((1-methyl-1H-pyrazol-4-yl)amino)pyrimidin-4-yl)amino)methyl)-1H-1,2,3-triazol-1-yl)methyl)-8-(hydroxymethyl)-1-methyl-4b,5,6,8,9,10-hexahydro-7H-7,9a-methanobenzo[a]azulene-7,8-diol (**30**)

Figure S101. ^{1}H-NMR of compound **30**

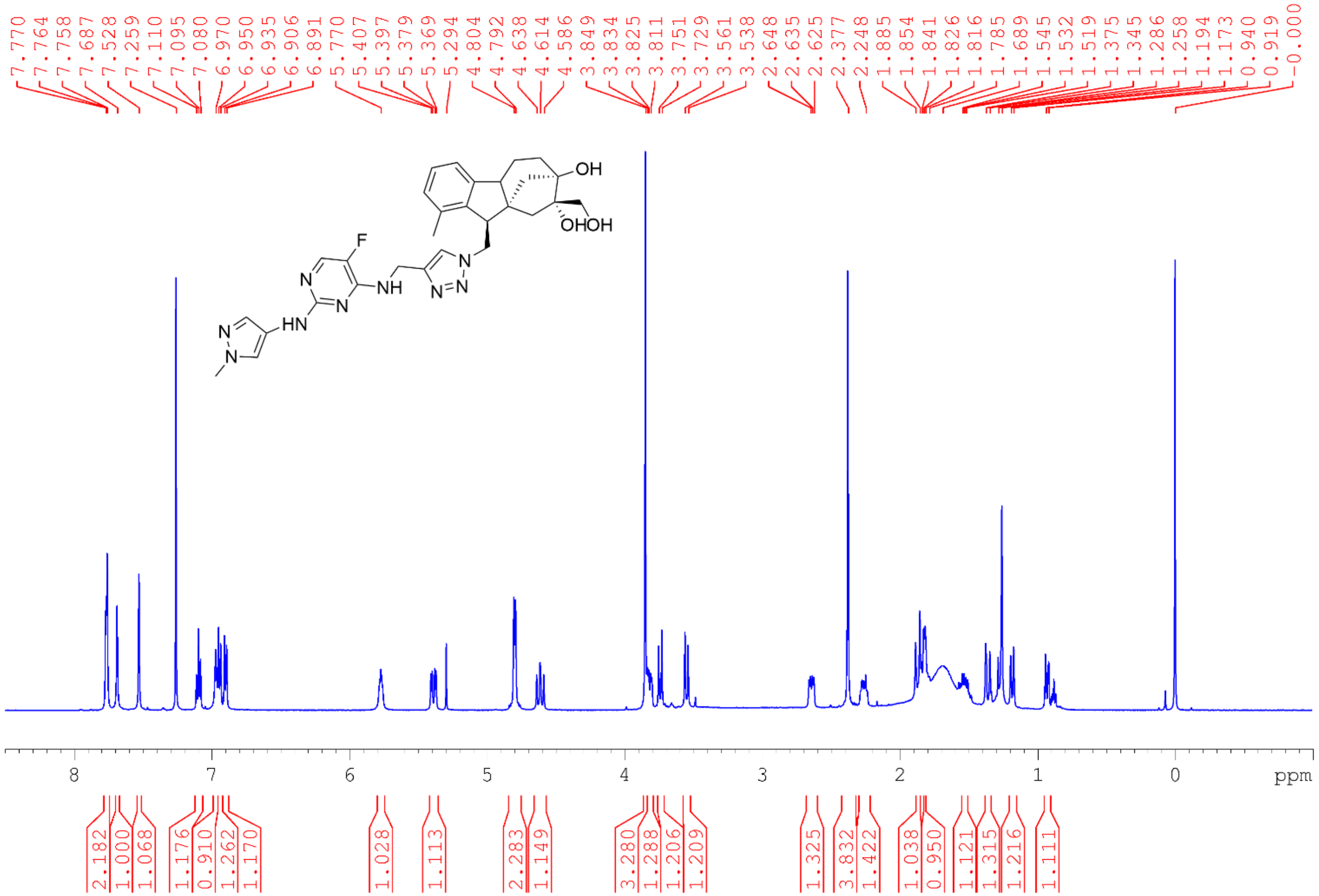

Figure S102. ^{13}C-NMR of compound **30**

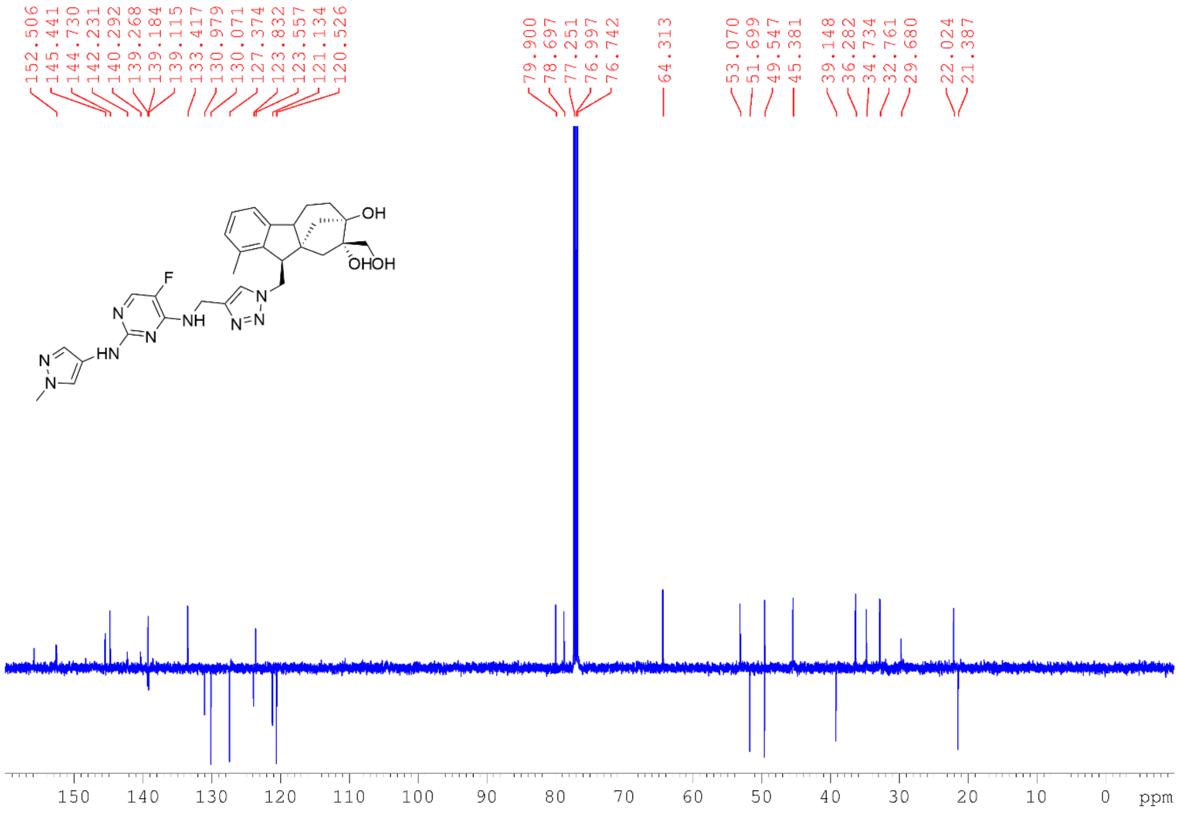

Figure S103. ^{19}F-NMR of compound **30**

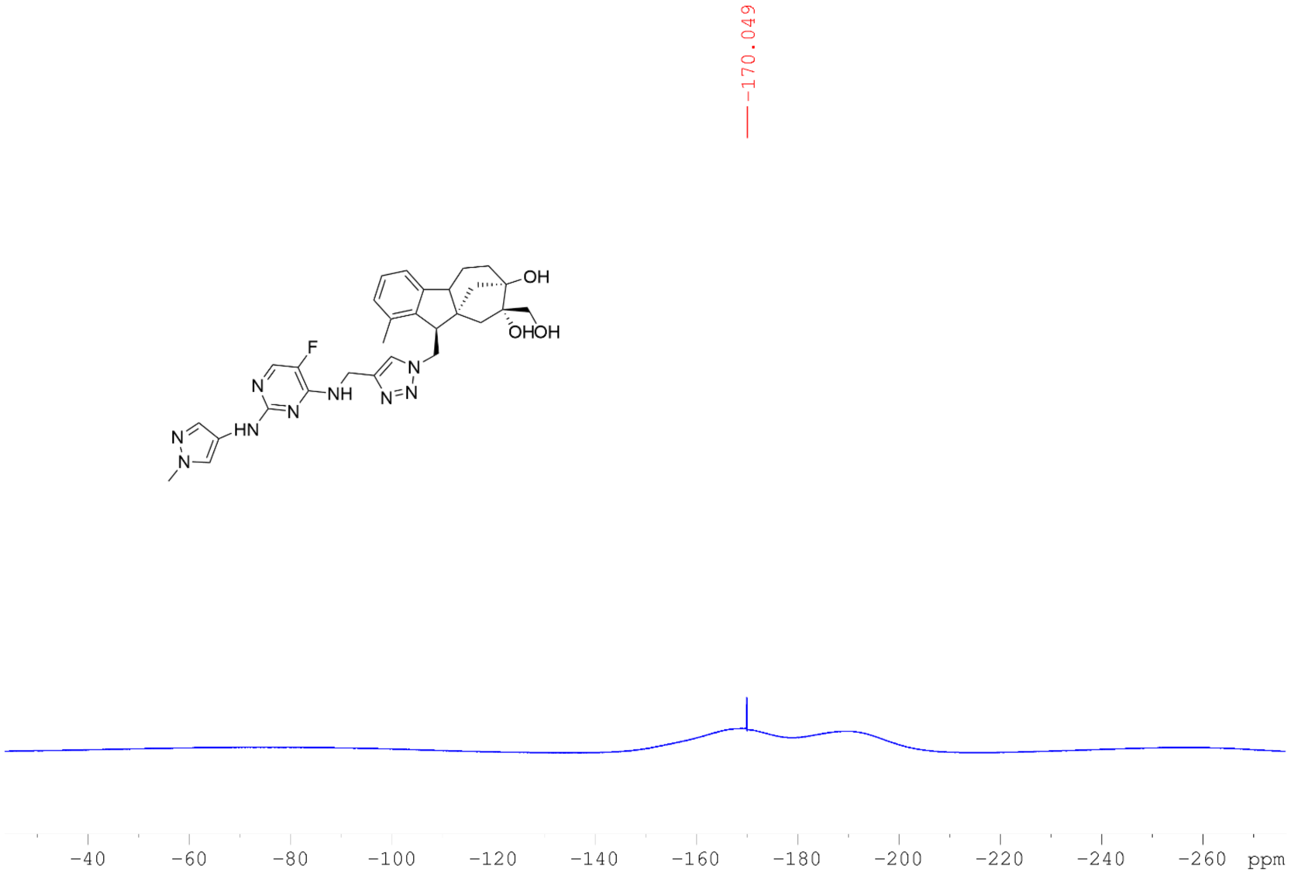

Figure S104. COSY-NMR of compound **30**

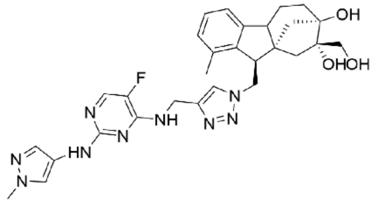

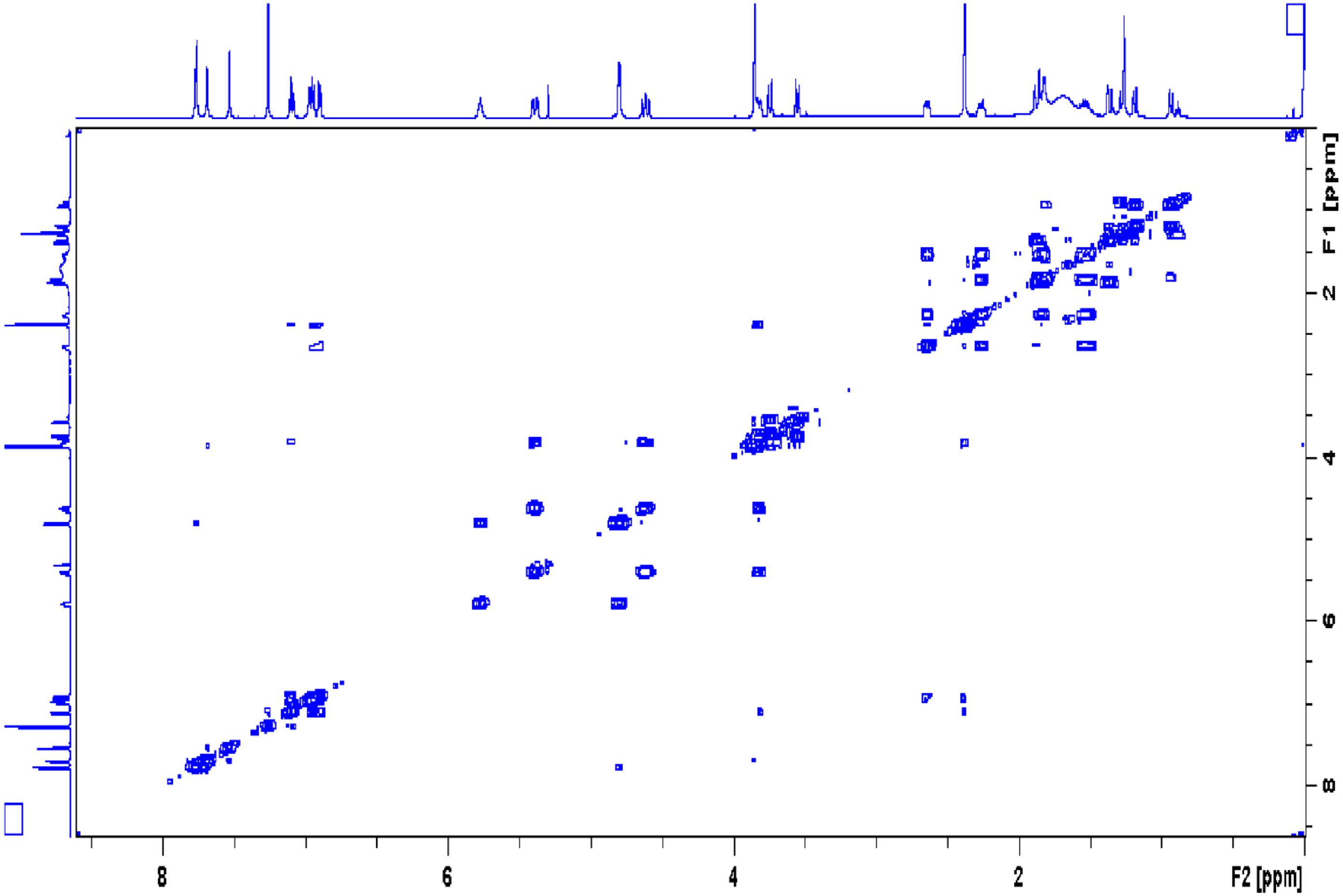

Figure S105. NOESY-NMR of compound **30**

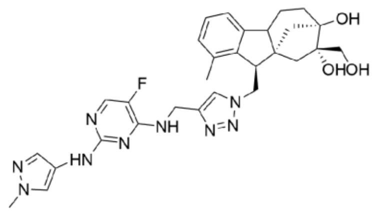

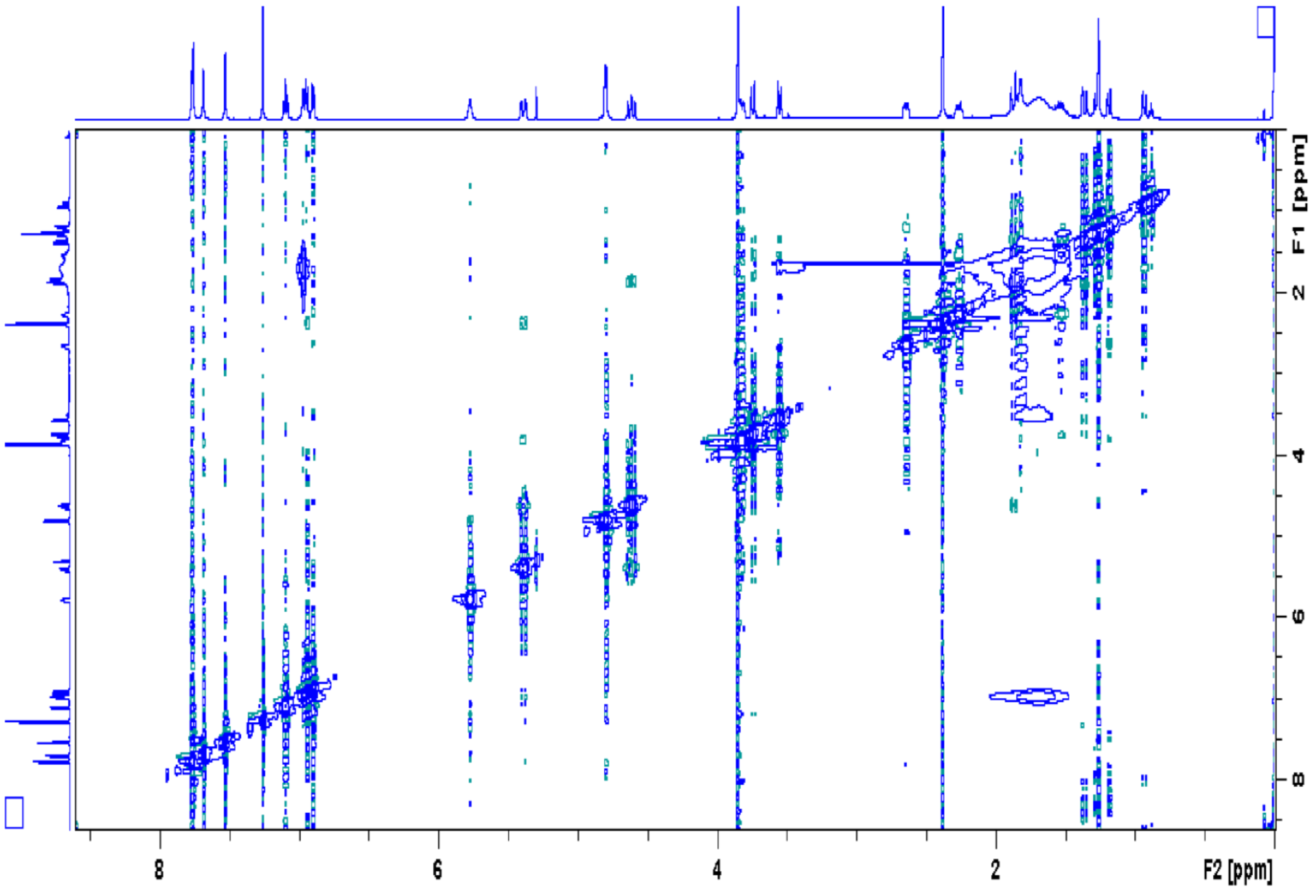

Figure S106. HSQC-NMR of compound **30**

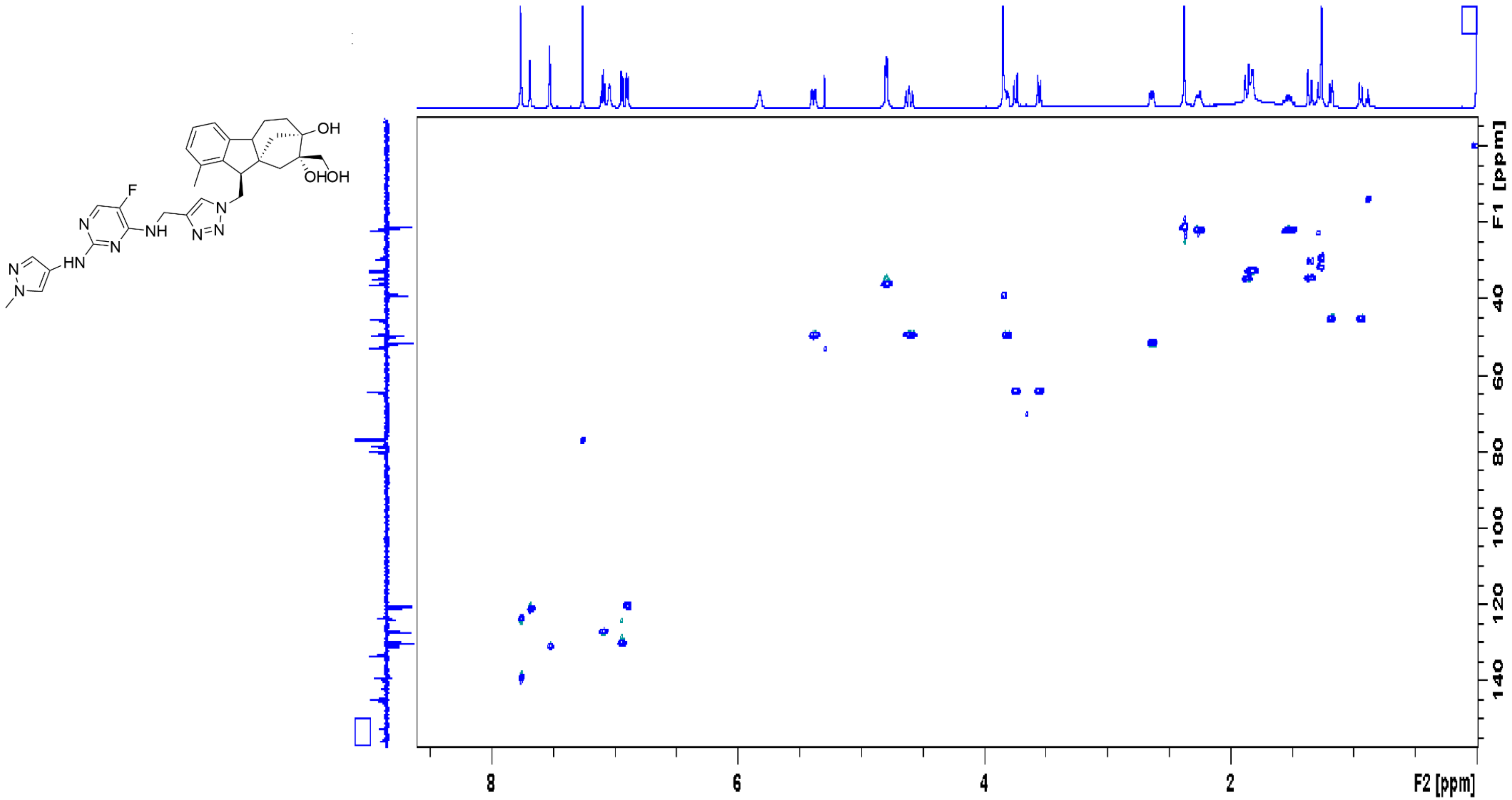

Figure S107. HMBC-NMR of compound **30**

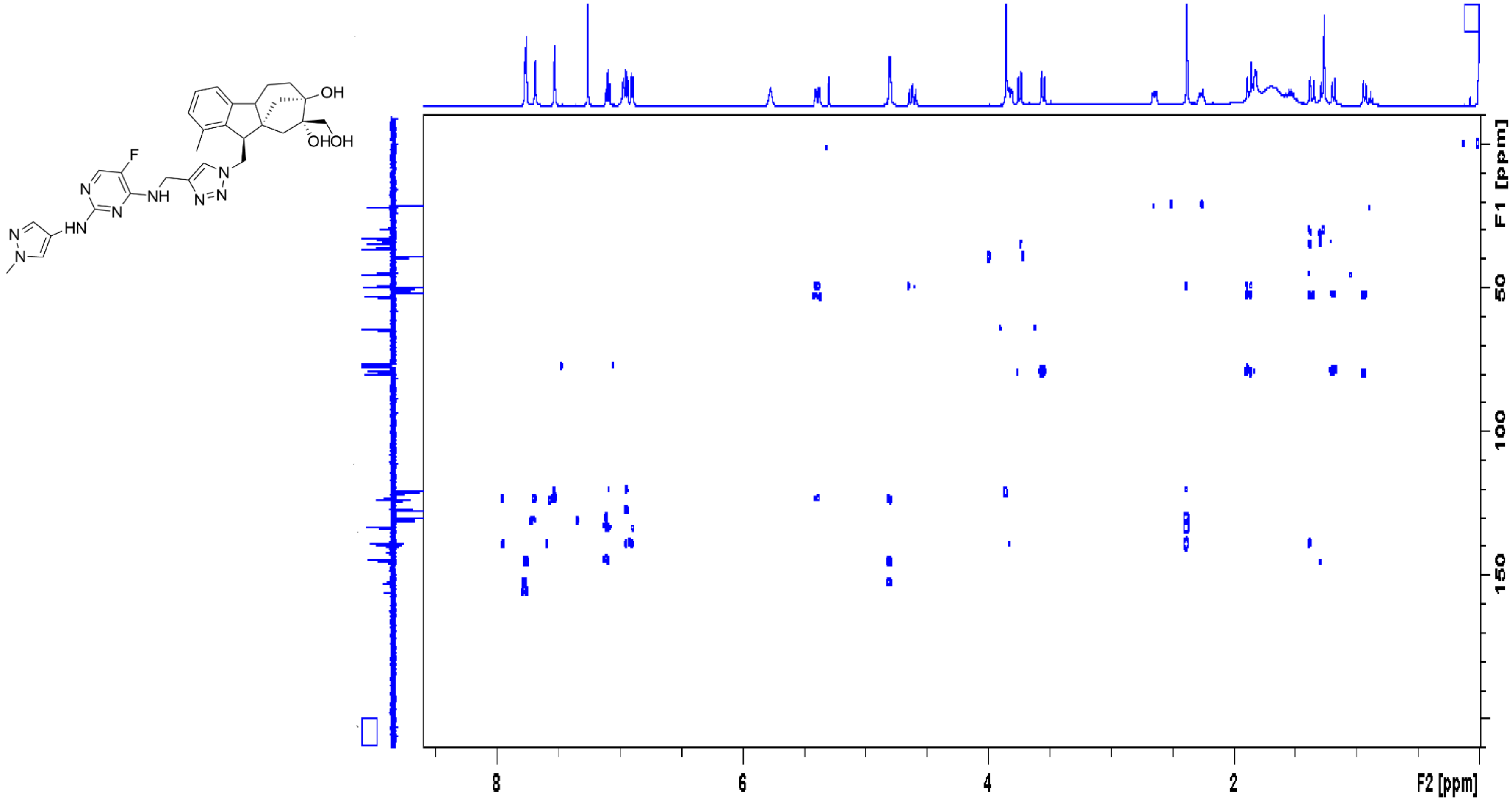

(7S,8S,9aR,10R)-10-((4-(((5-chloro-2-((1-methyl-1’H-pyrazol-4-yl)amino)pyrimidin-4-yl)amino)methyl)-1H-1,2,3-triazol-1-yl)methyl)-8-(hydroxymethyl)-1-methyl-4b,5,6,8,9,10-hexahydro-7H-7,9a-methanobenzo[a]azulene-7,8-diol (**31**)

Figure S108. ^{1}H-NMR of compound **31**

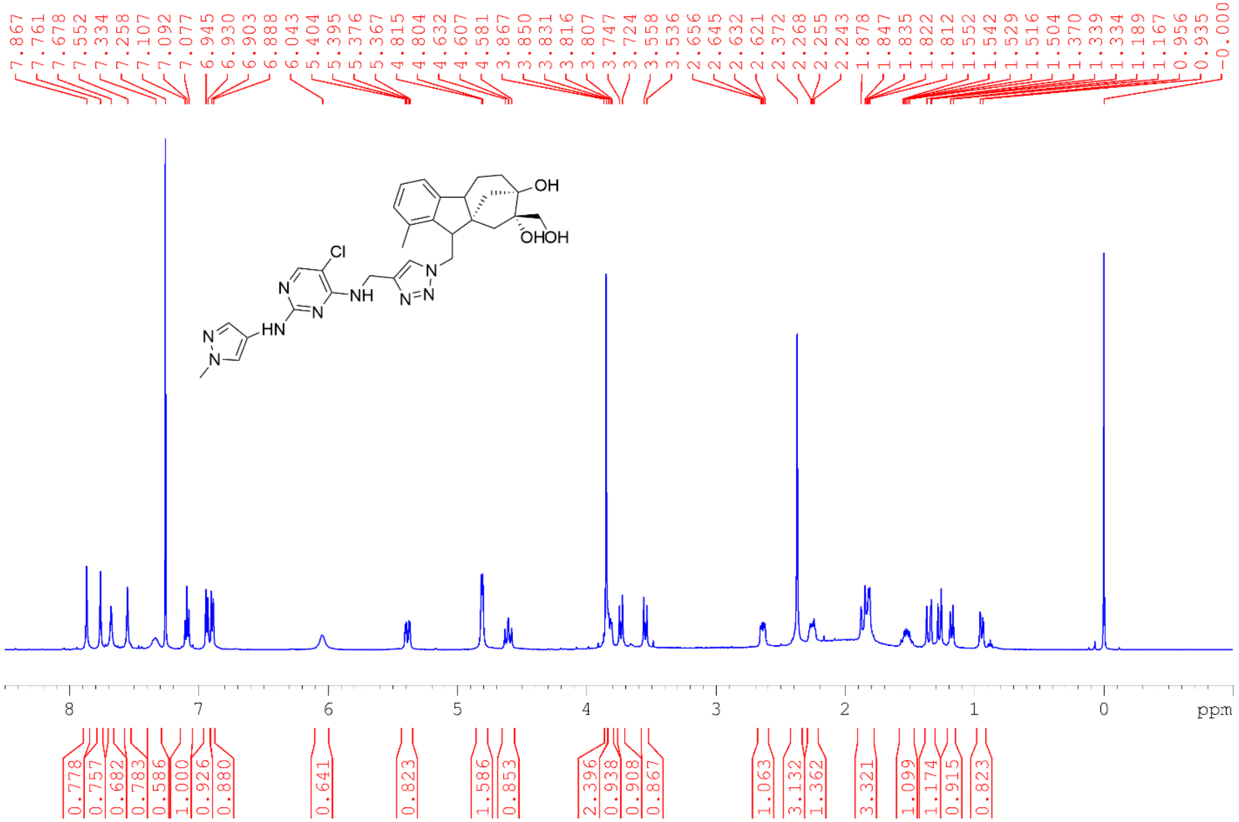

Figure S109. ^{13}C-NMR of compound **31**

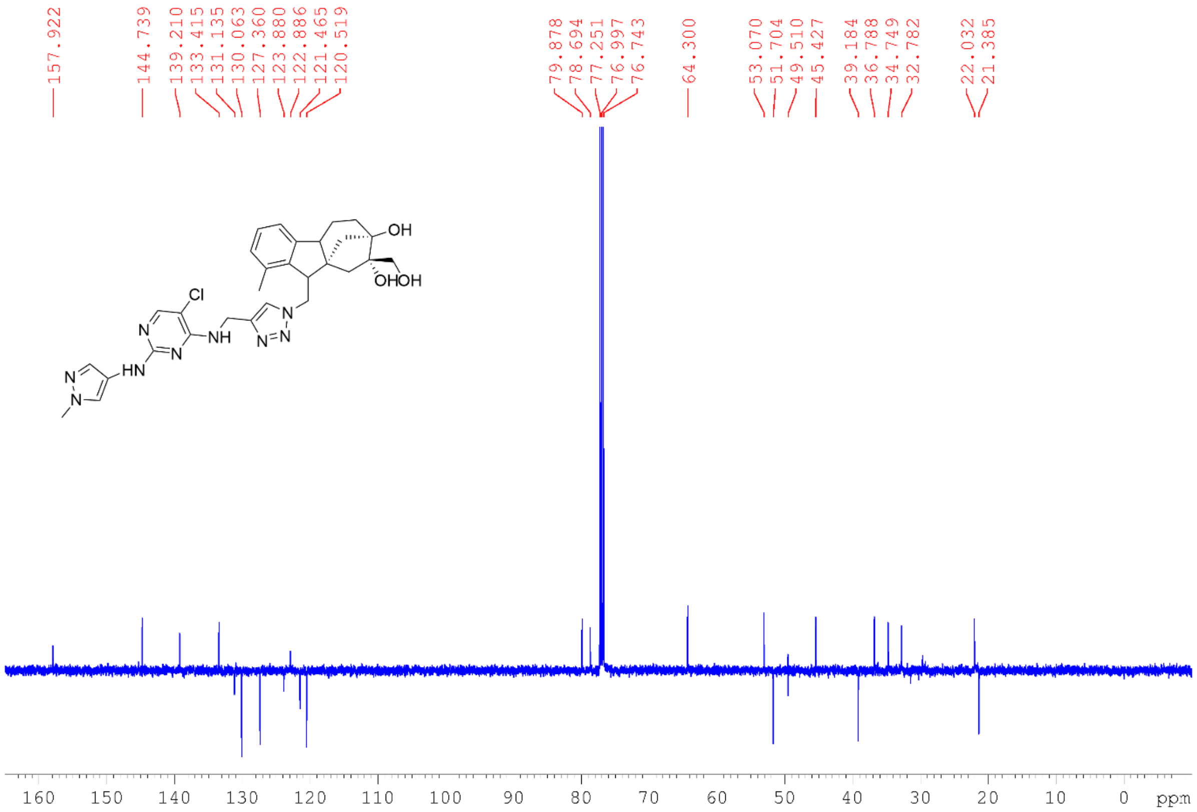

Figure S110. COSY-NMR of compound **31**

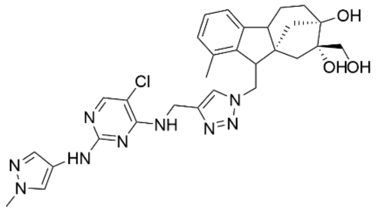

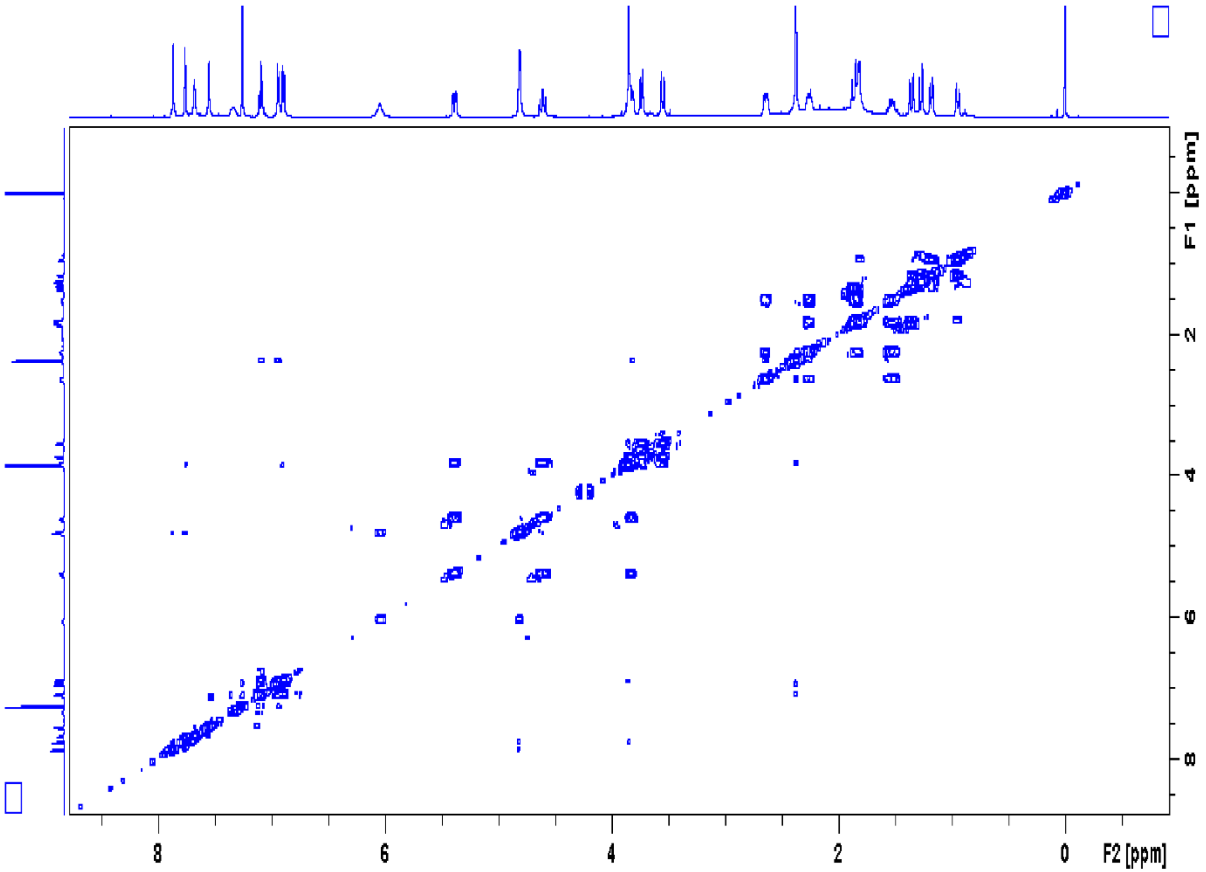

Figure S111. NOESY-NMR of compound **31**

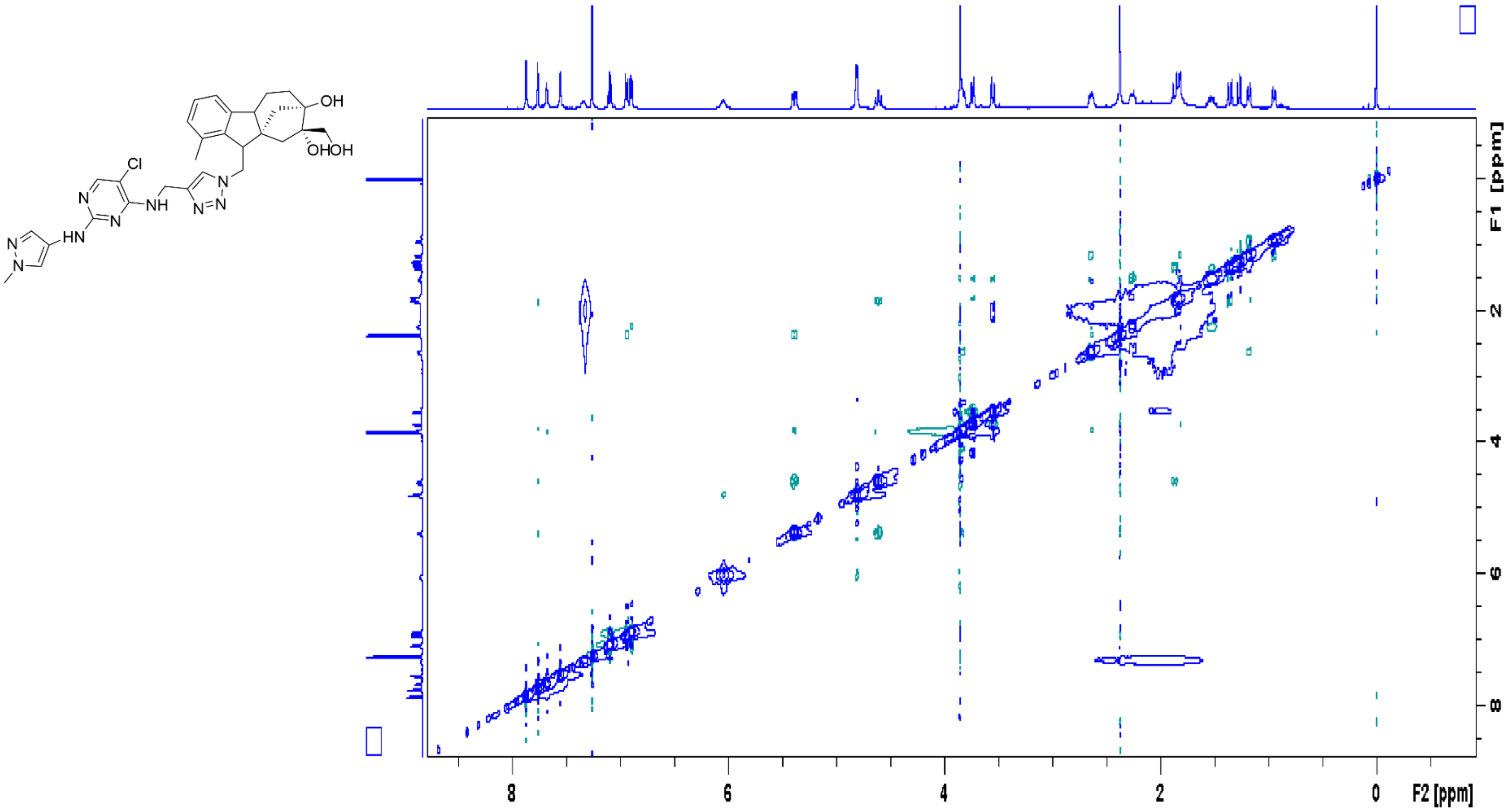

Figure S112. HSQC-NMR of compound **31**

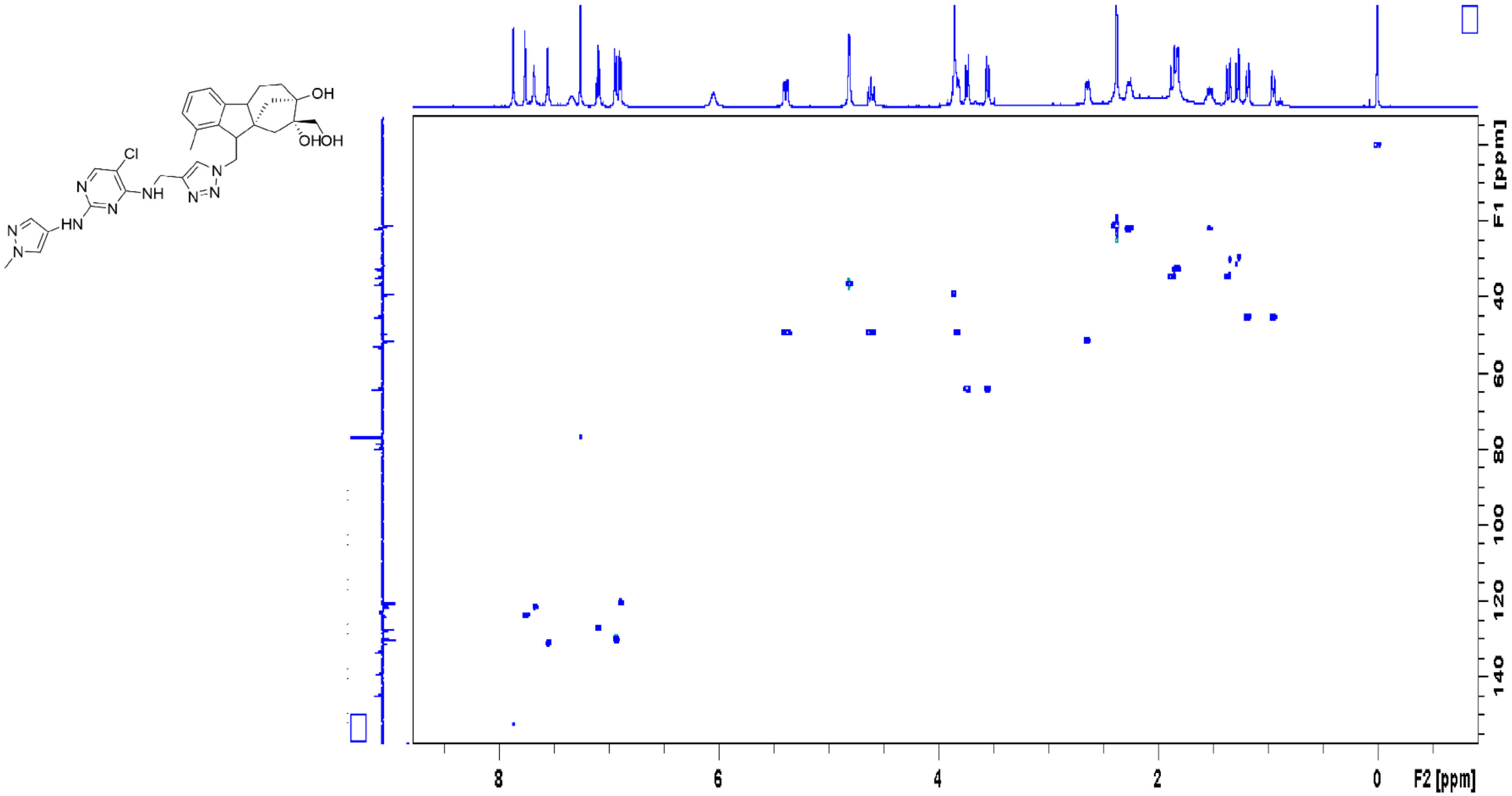

Figure S112. HMBC-NMR of compound **31**

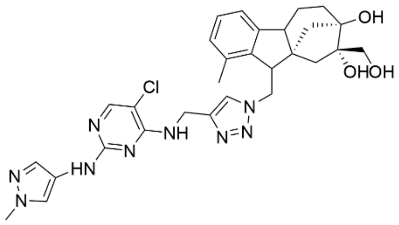

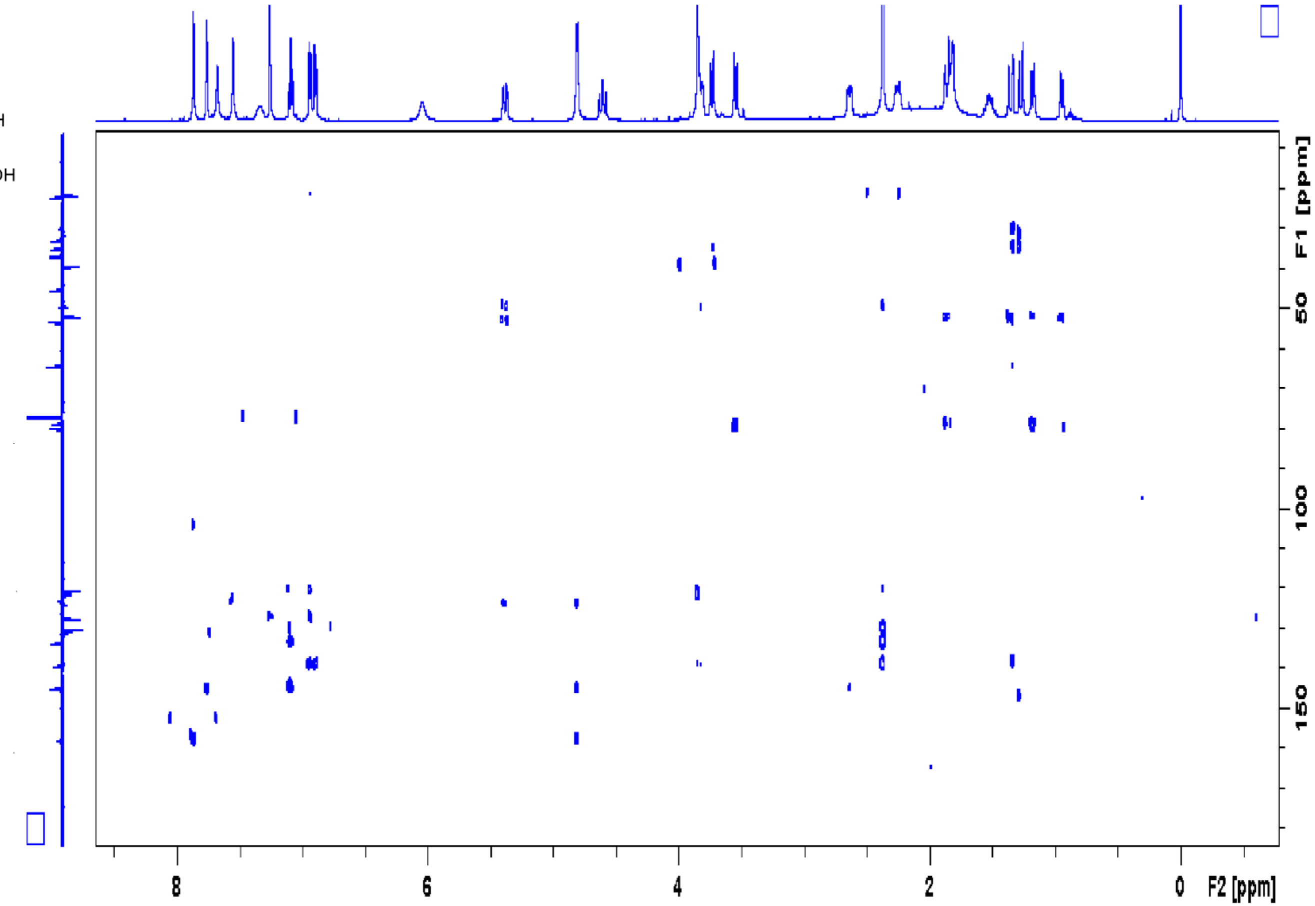

Methyl 4-((4-(((1-(((7S,8S,9aR,10R)-7,8-dihydroxy-8-(hydroxymethyl)-1-methyl-4b,6,7,8,9,10-hexahydro-5H-7,9a-methanobenzo[a]azulen-10-yl)methyl)-1H-1,2,3-triazol-4-yl)methyl)amino)-5-fluoropyrimidin-2-yl)amino)benzoate (**32**)

Figure S113. ^{1}H-NMR of compound **32**

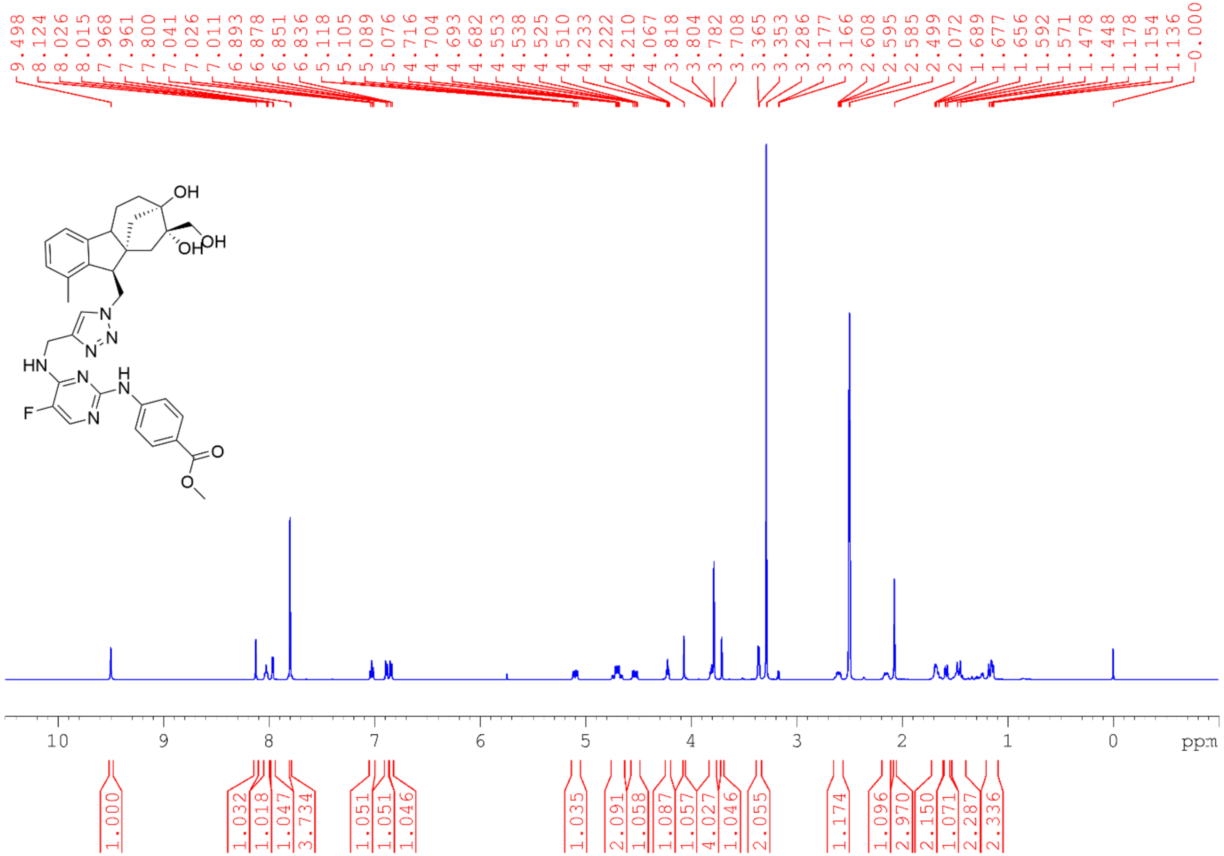

Figure S114. ^{13}C-NMR of compound **32**

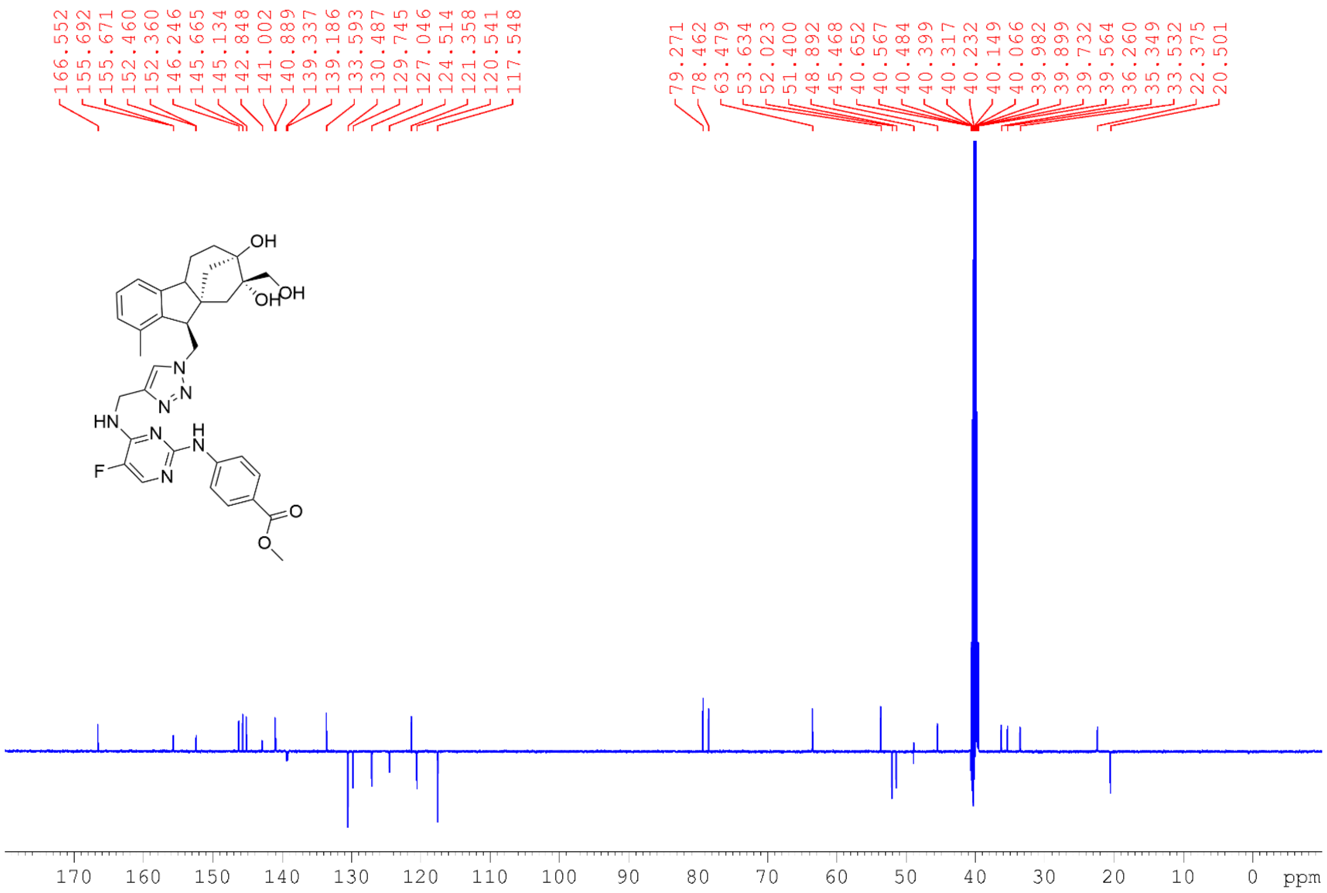

Figure S115. ^{19}F-NMR of compound **32**

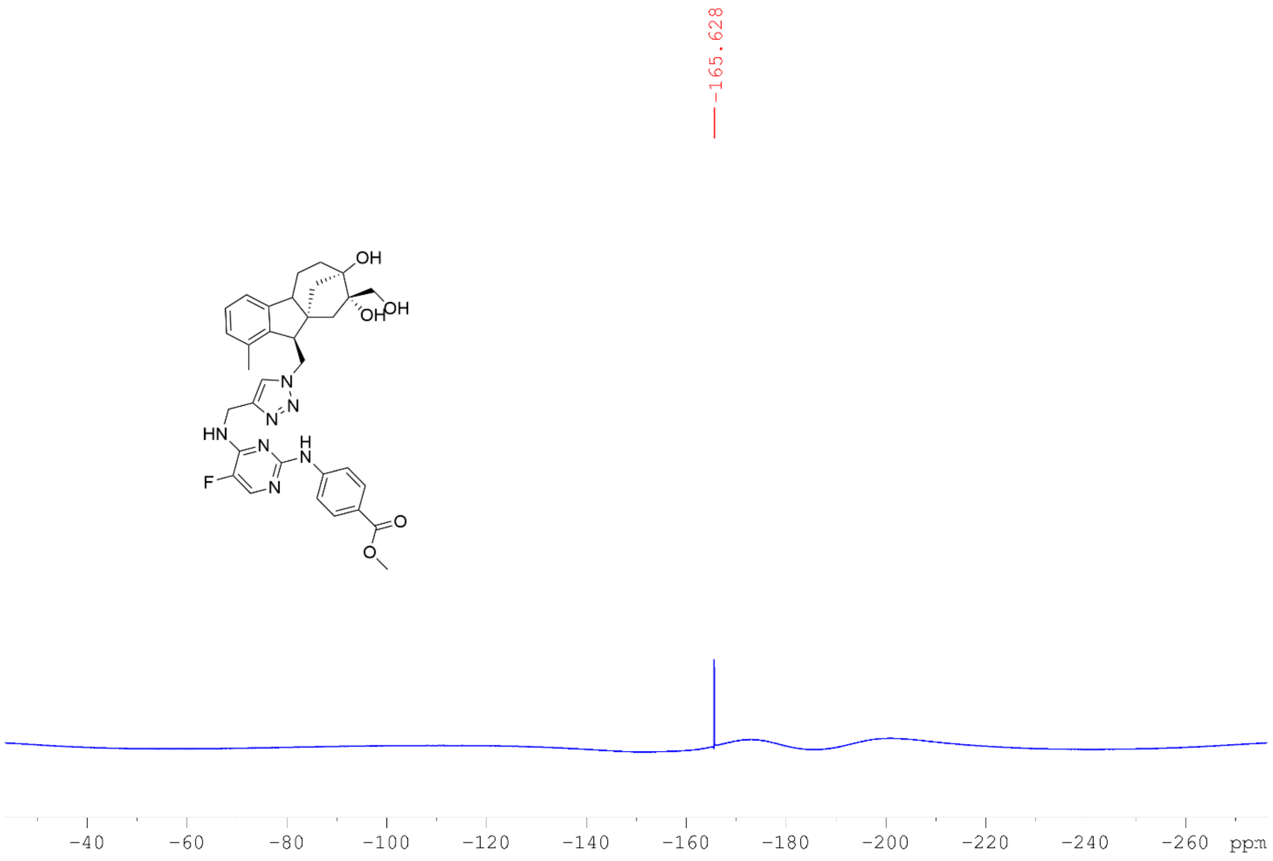

Figure S116. COSY-NMR of compound **32**

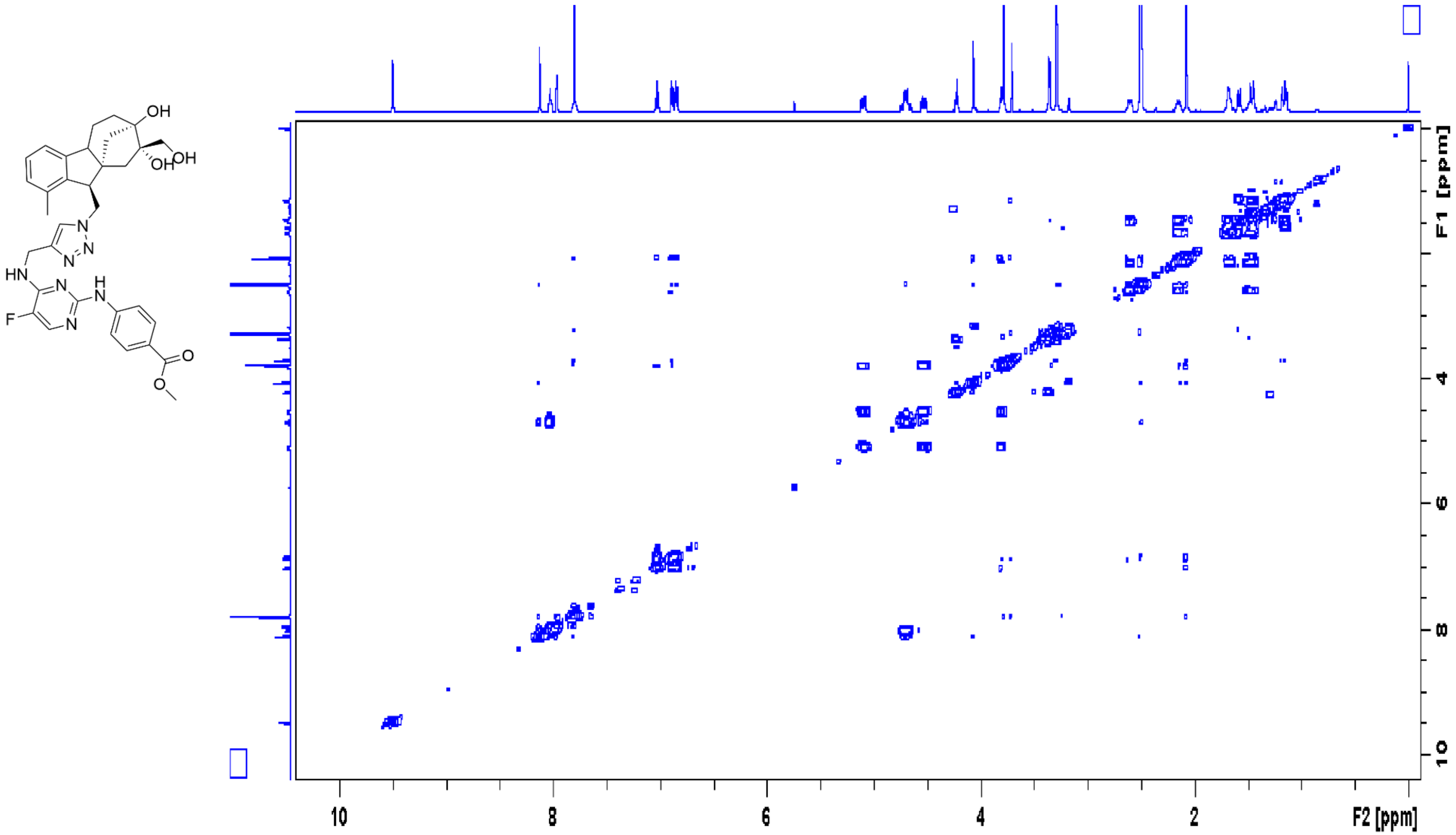

Figure S117. NOESY-NMR of compound **32**

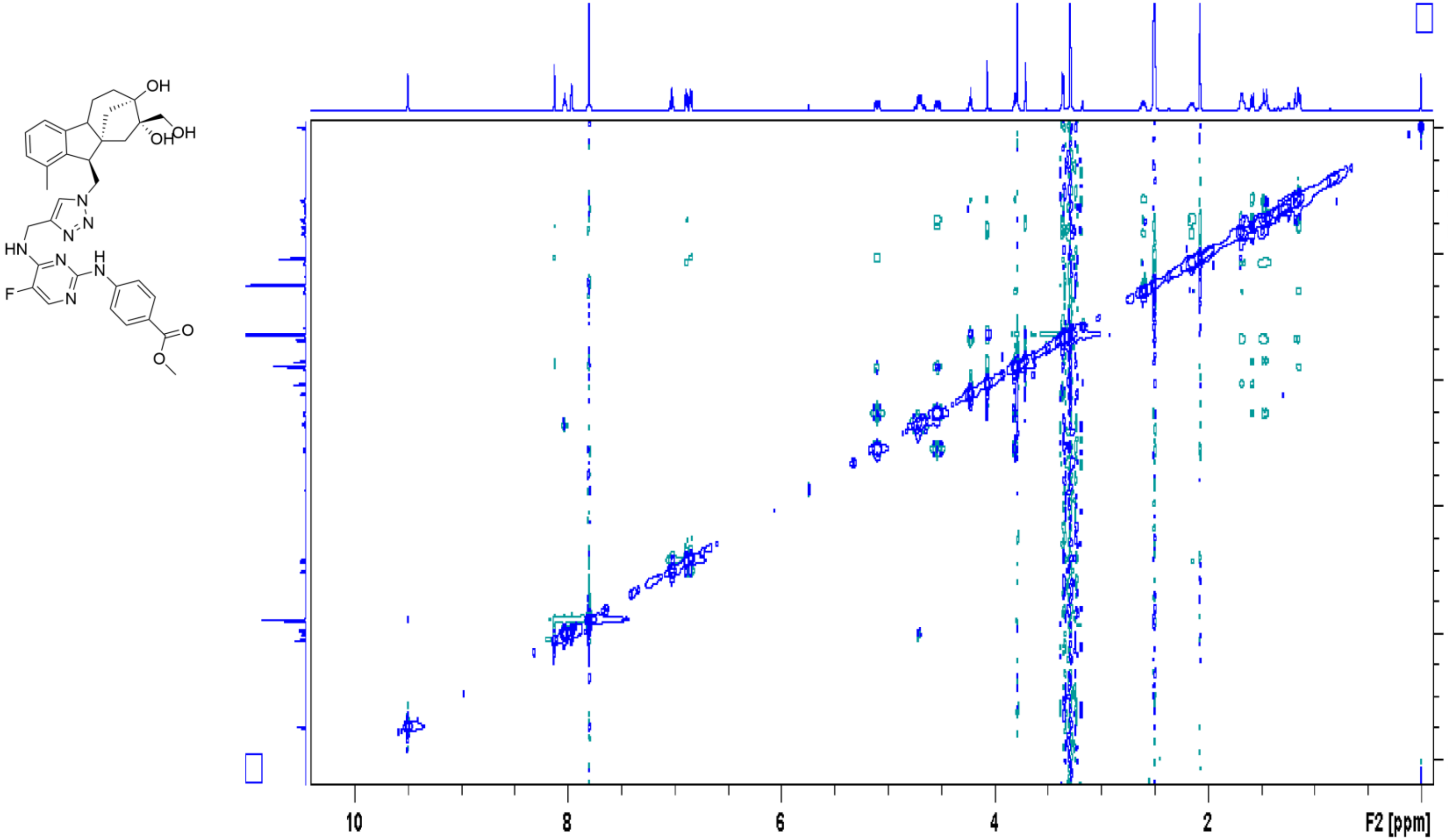

Figure S118. HSQC-NMR of compound **32**

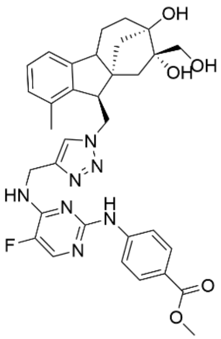

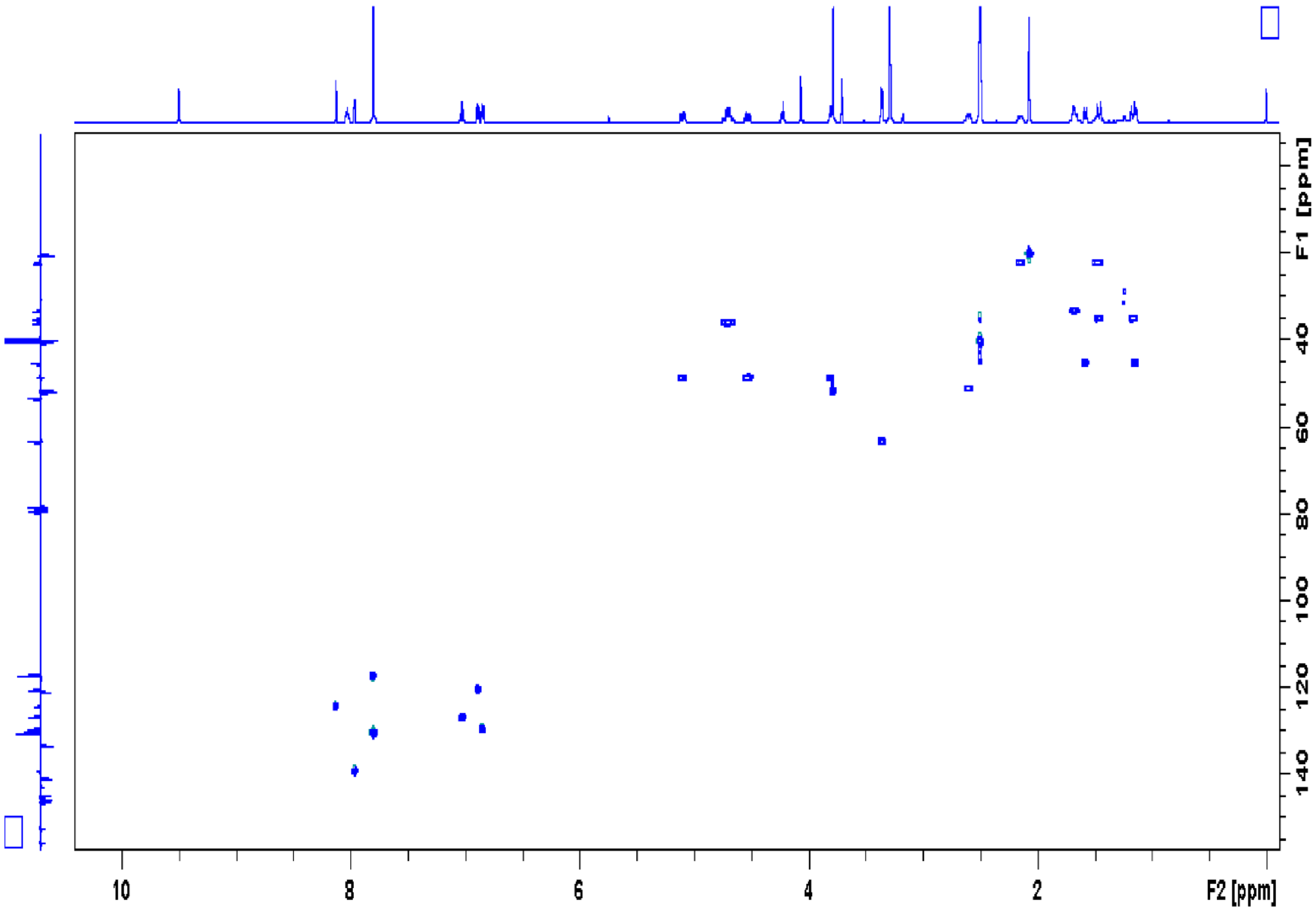

Figure S118. HMBC-NMR of compound **32**

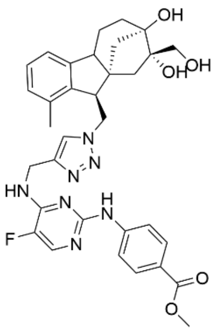

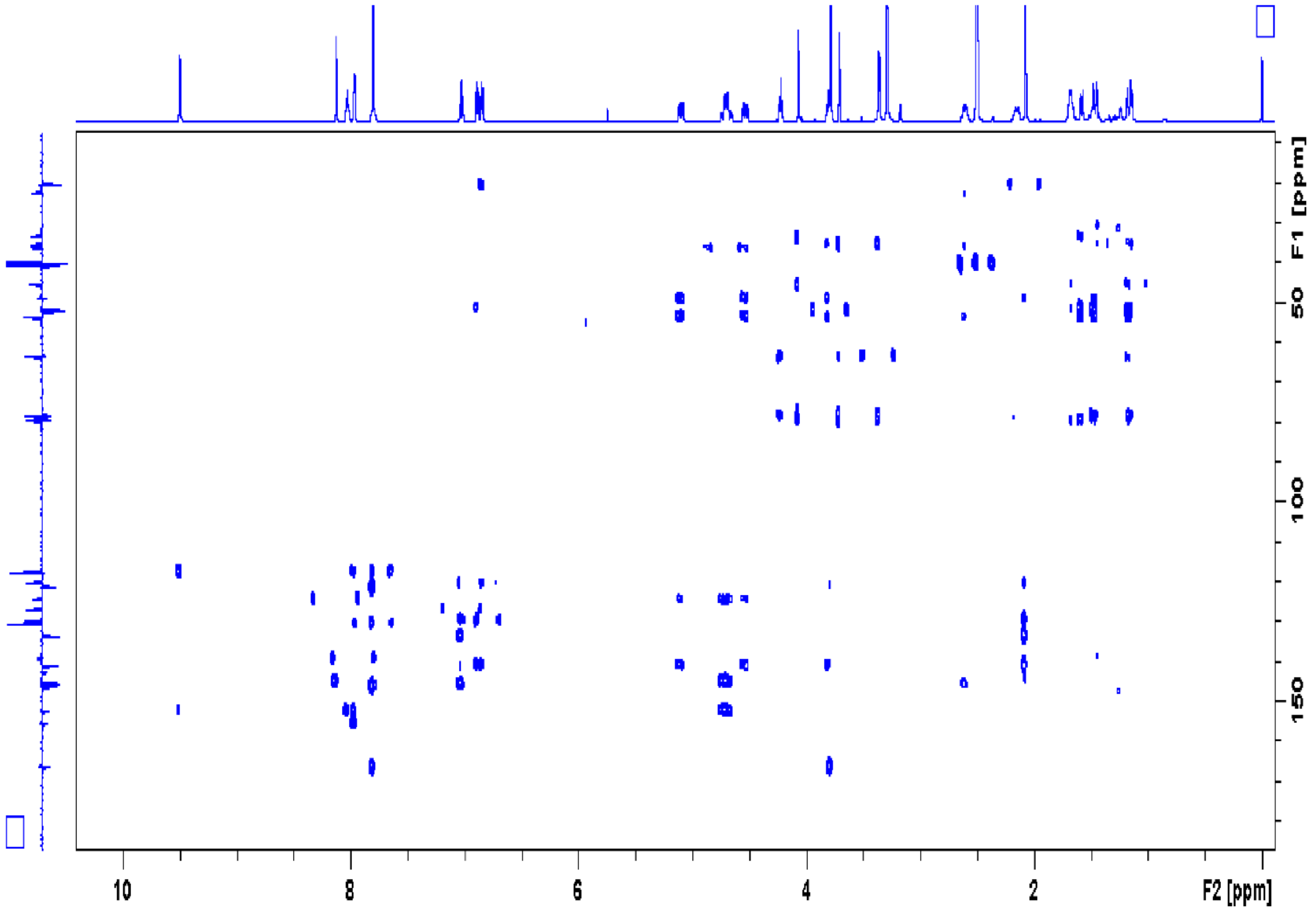

Methyl 4-((5-chloro-4-(((1-(((7S,8S,9aR,10R)-7,8-dihydroxy-8-(hydroxymethyl)-1-methyl-4b,6,7,8,9,10-hexahydro-5H-7,9a-methanobenzo[a]azulen-10-yl)methyl)-1H-1,2,3-triazol-4-yl)methyl)amino)pyrimidin-2-yl)amino)benzoate (**33**)

Figure S119. ^{1}H-NMR of compound **33**

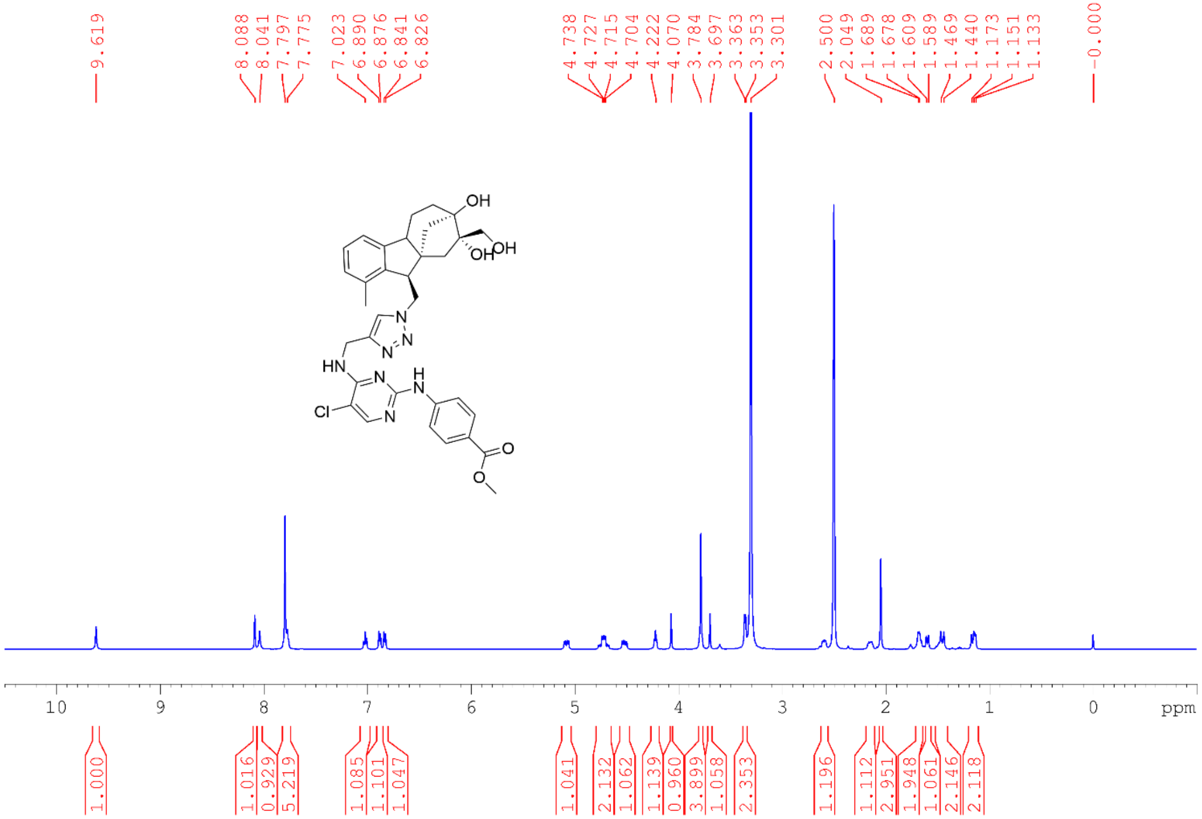

Figure S120. ^{13}C-NMR of compound **33**

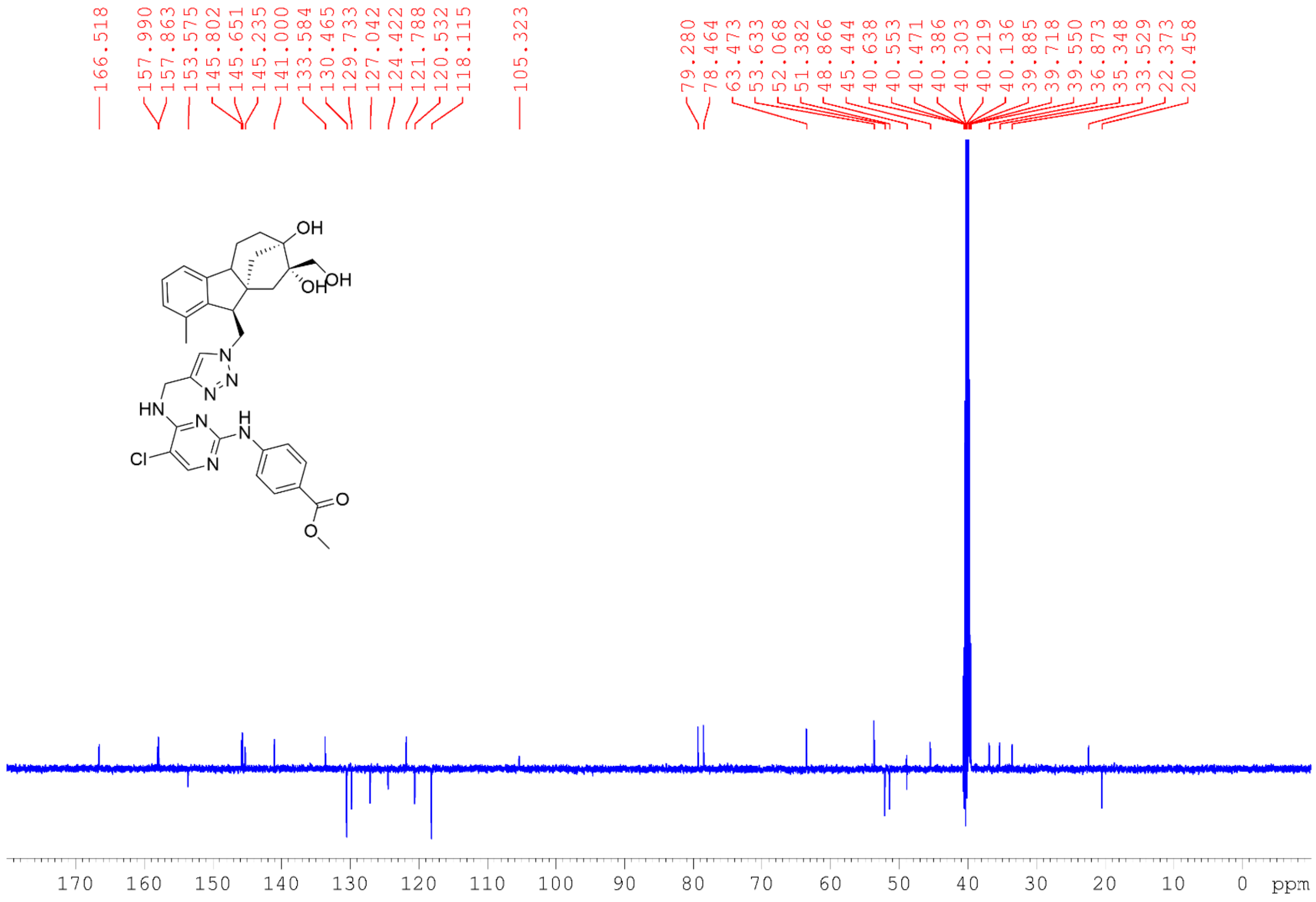

Figure S121. COSY-NMR of compound **33**

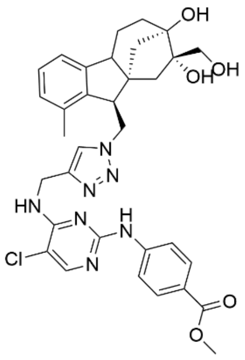

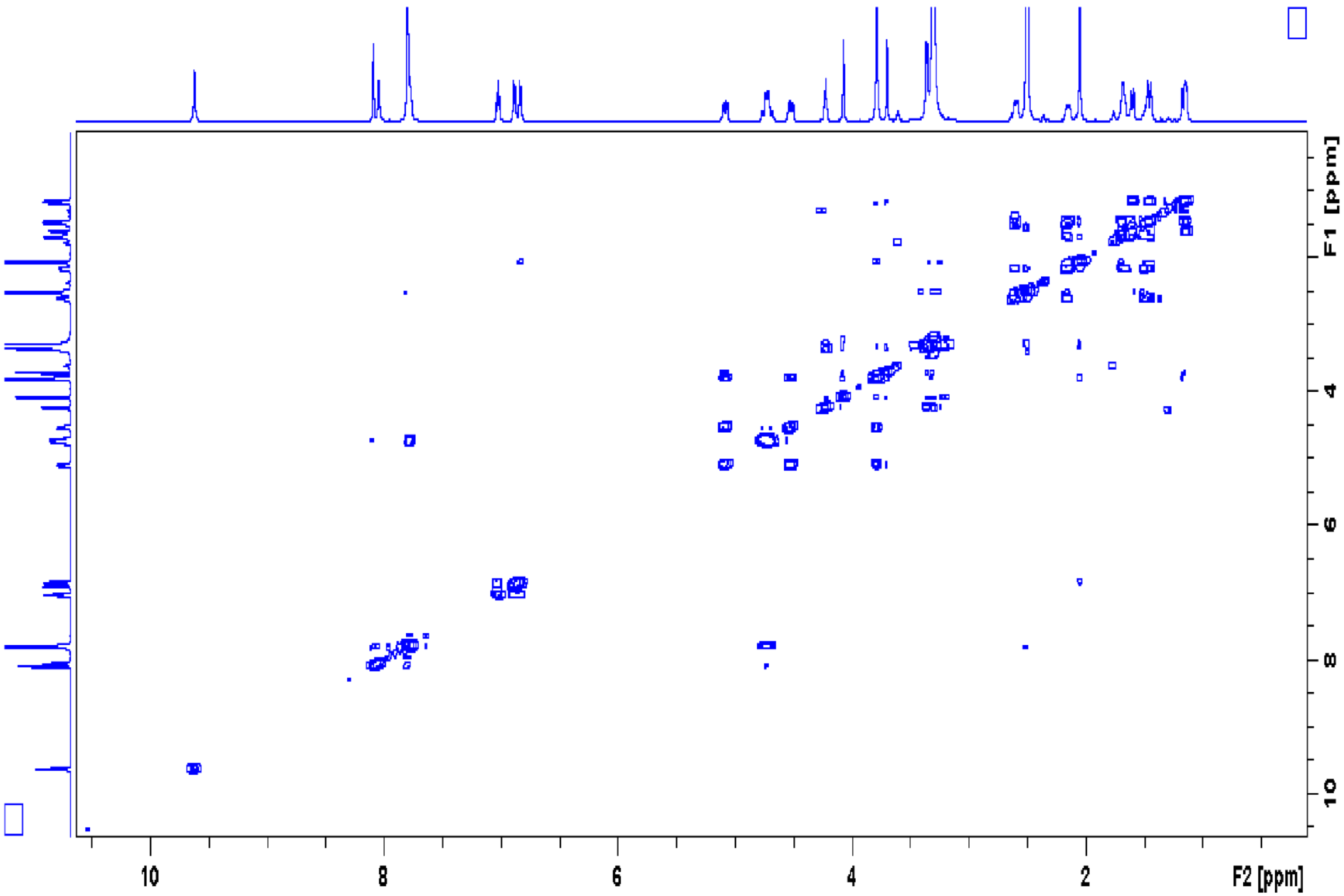

Figure S122. NOESY-NMR of compound **33**

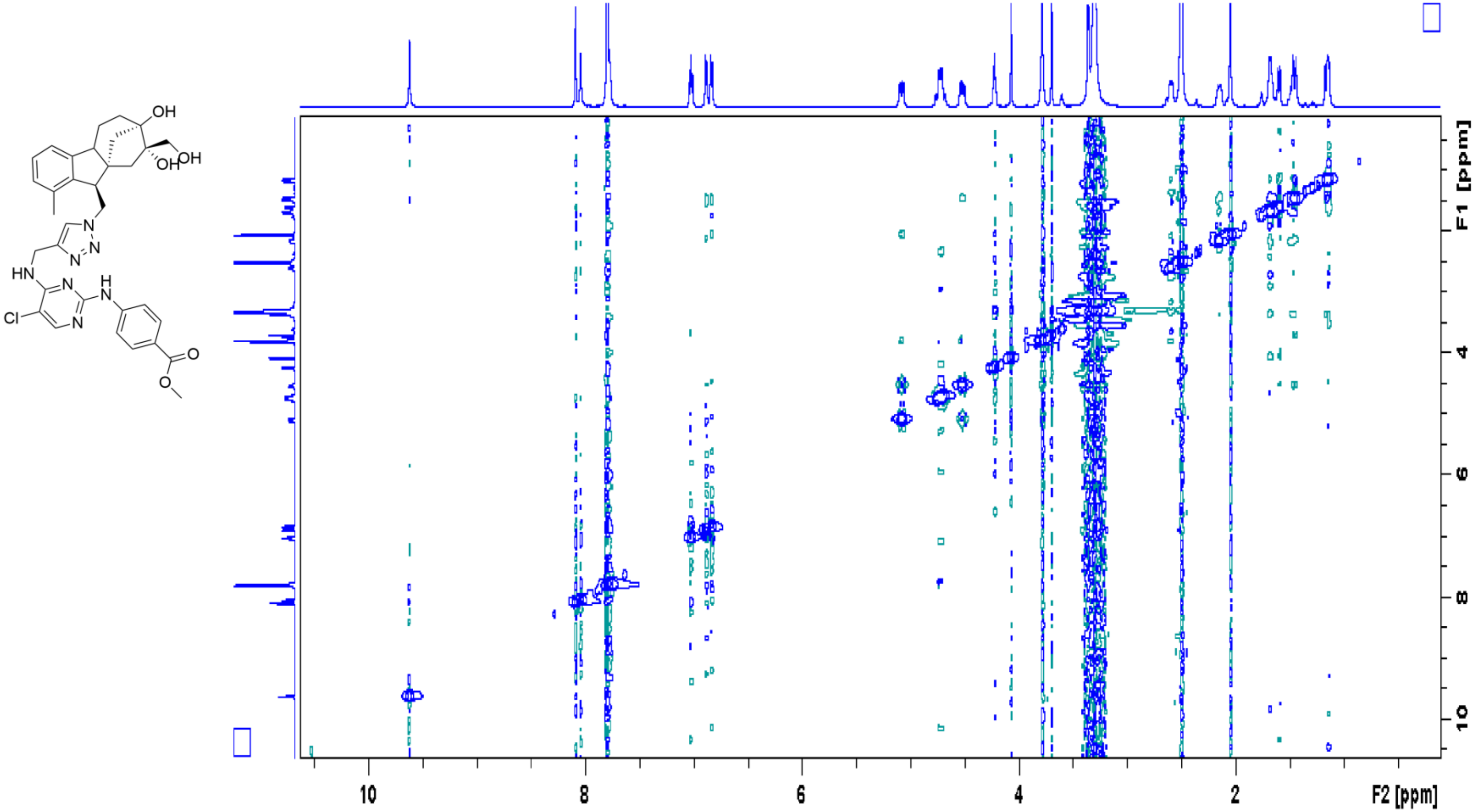

Figure S123. HSQC-NMR of compound **33**

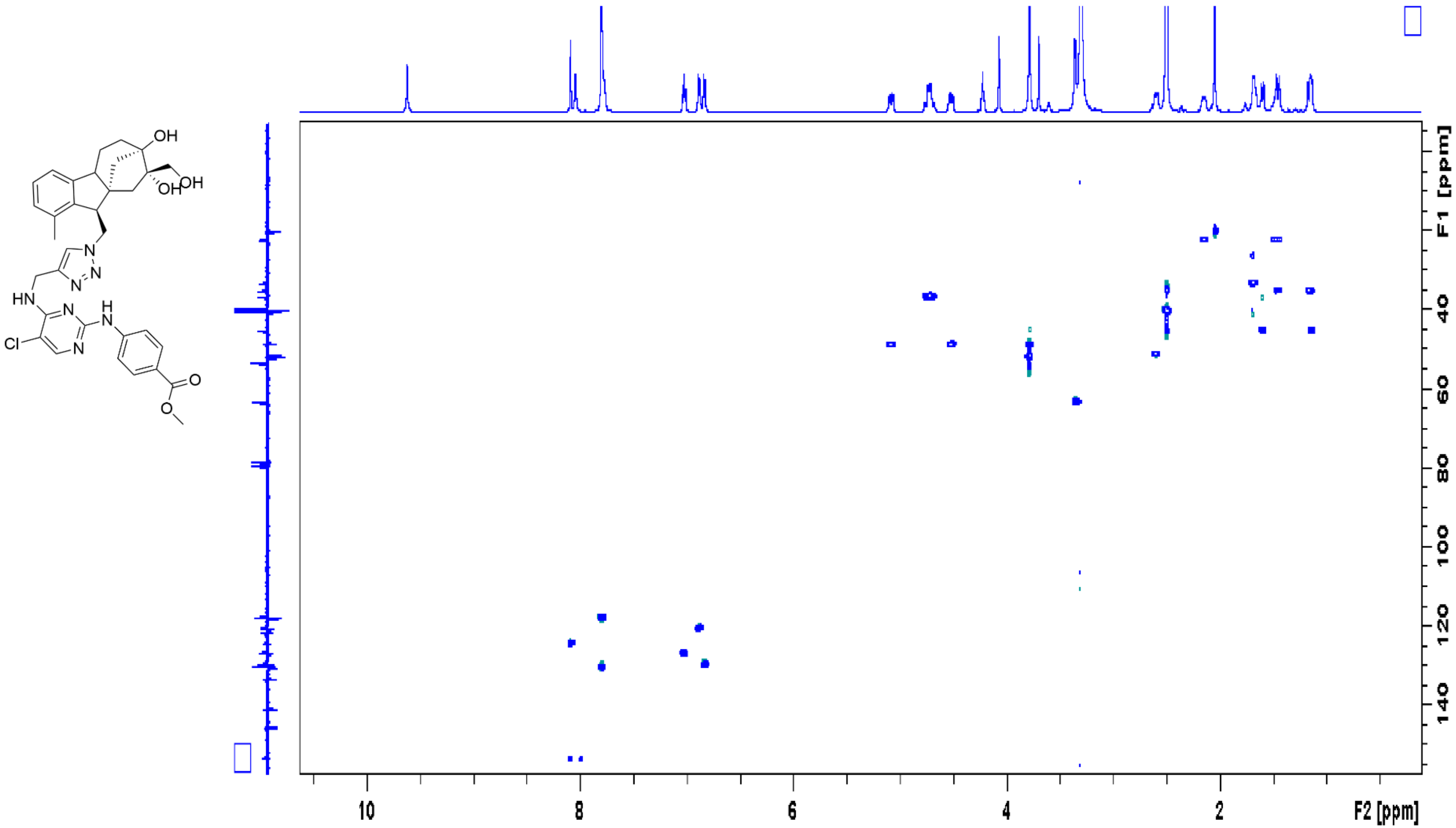

Figure S124. HMBC-NMR of compound **33**

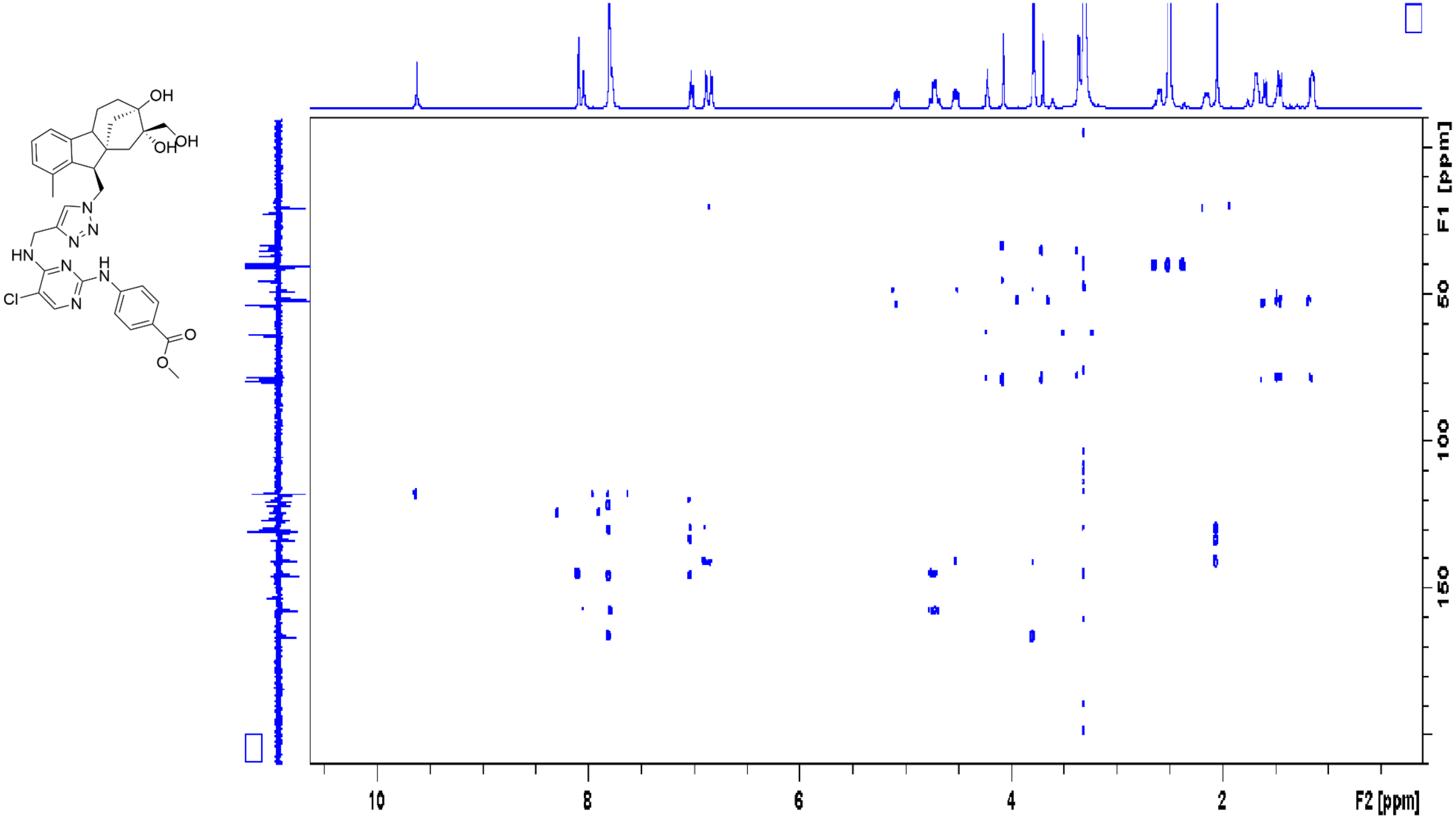

(7S,8S,9aR,10R)-10-((4-(((5-Fluoro-2-((4-morpholinophenyl)amino)pyrimidin-4-yl)amino)me-thyl)-1H-1,2,3-triazol-1-yl)methyl)-8-(hydroxymethyl)-1-methyl-4b,5,6,8,9,10-hexahydro-7H-7,9a-methanobenzo[a]azulene-7,8-diol (**34**)

Figure S125. ^{1}H-NMR of compound **34**

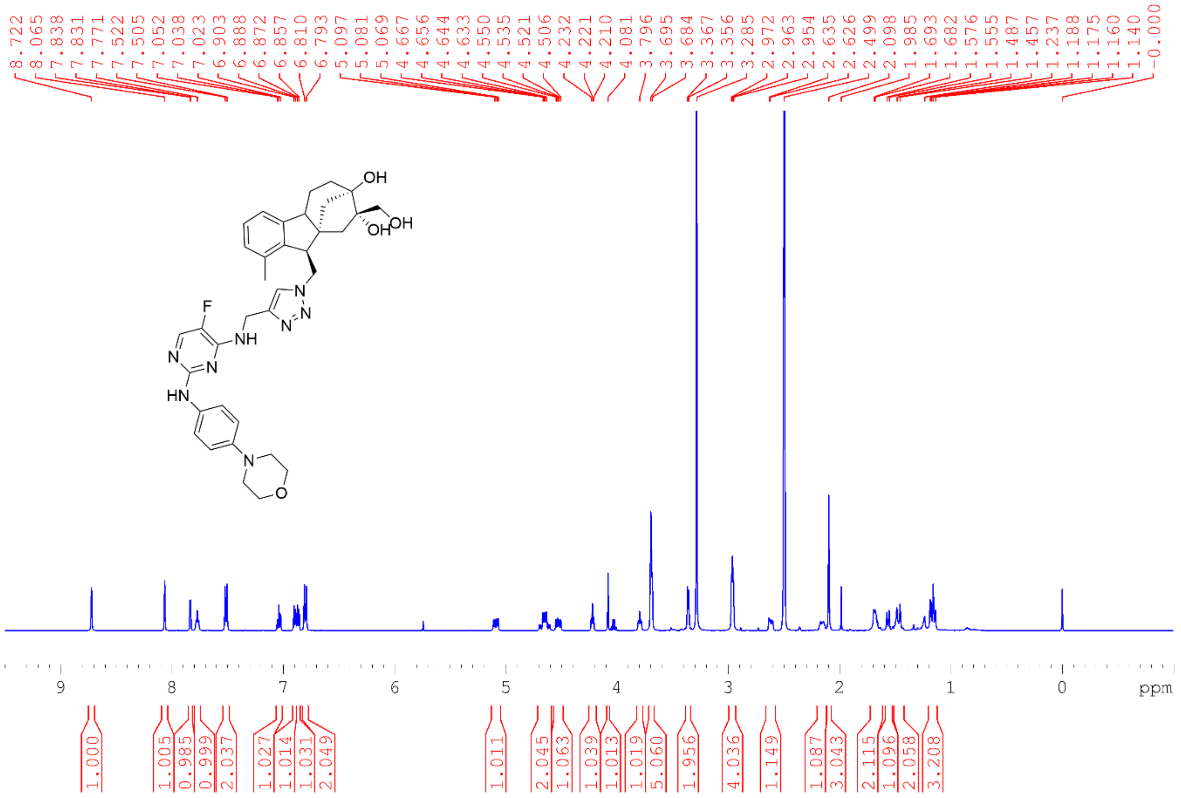

Figure S126. ^{13}C-NMR of compound **34**

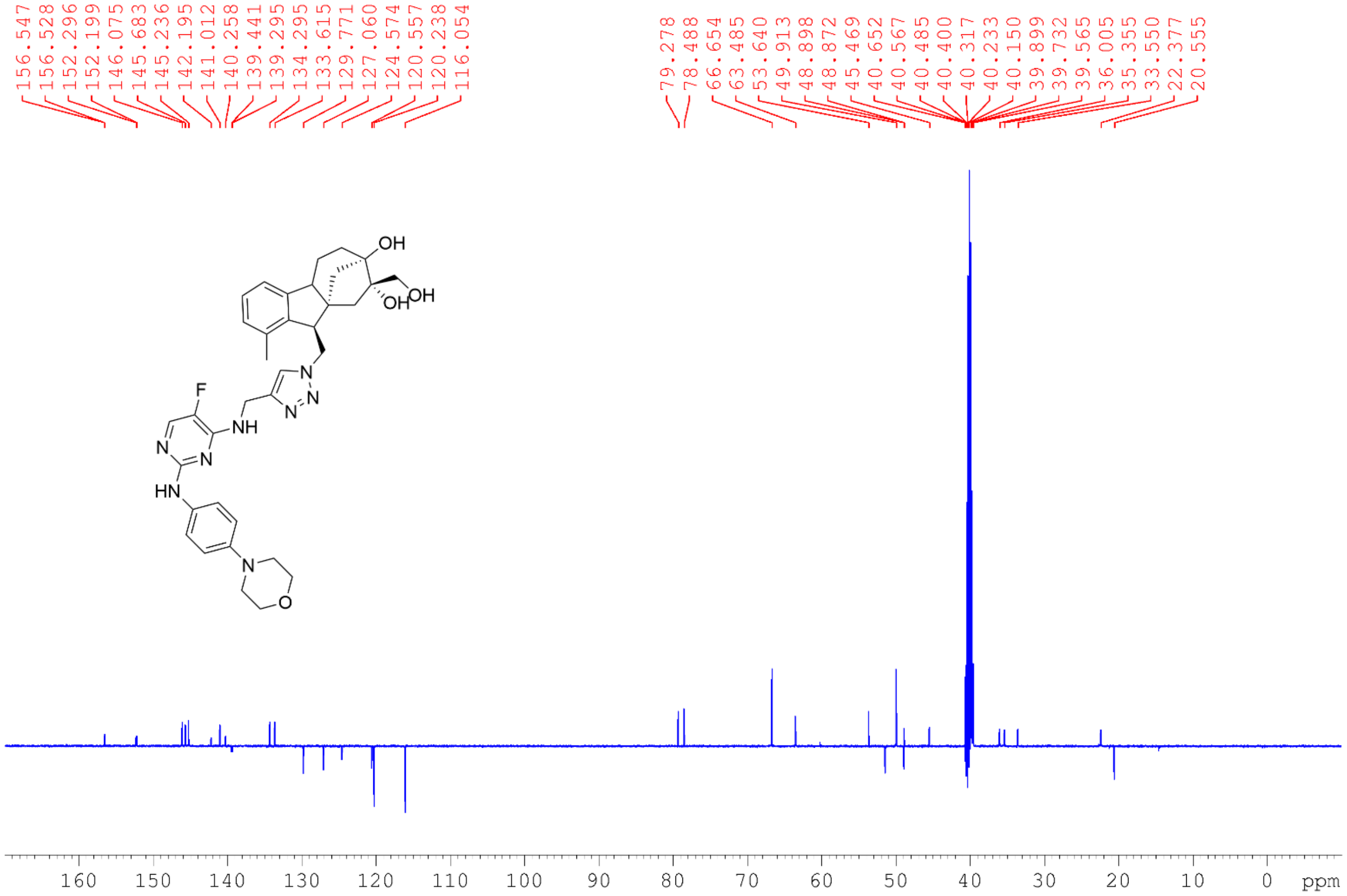

Figure S127. ^{19}F-NMR of compound **34**

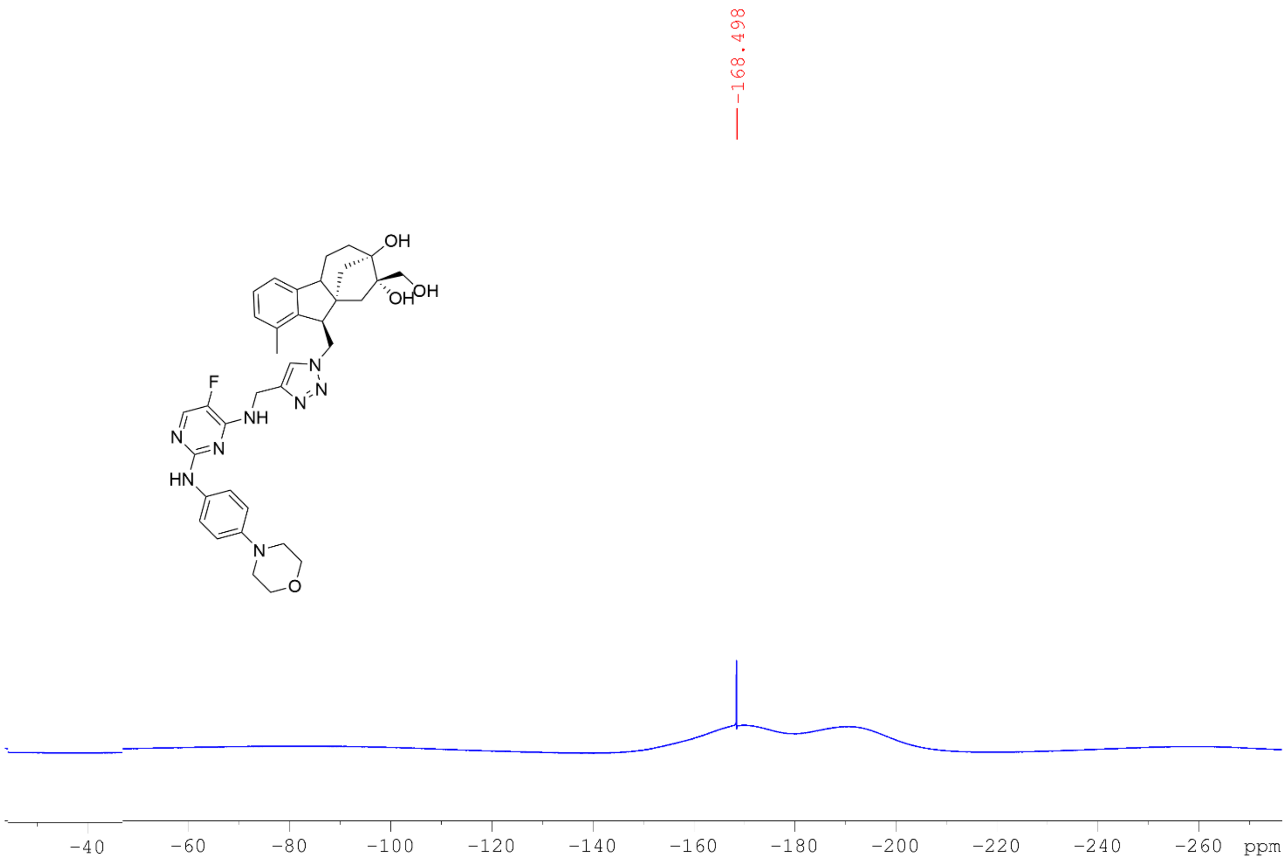

Figure S128. COSY-NMR of compound **34**

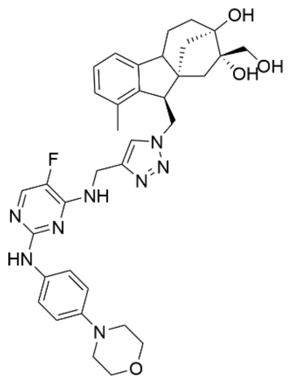

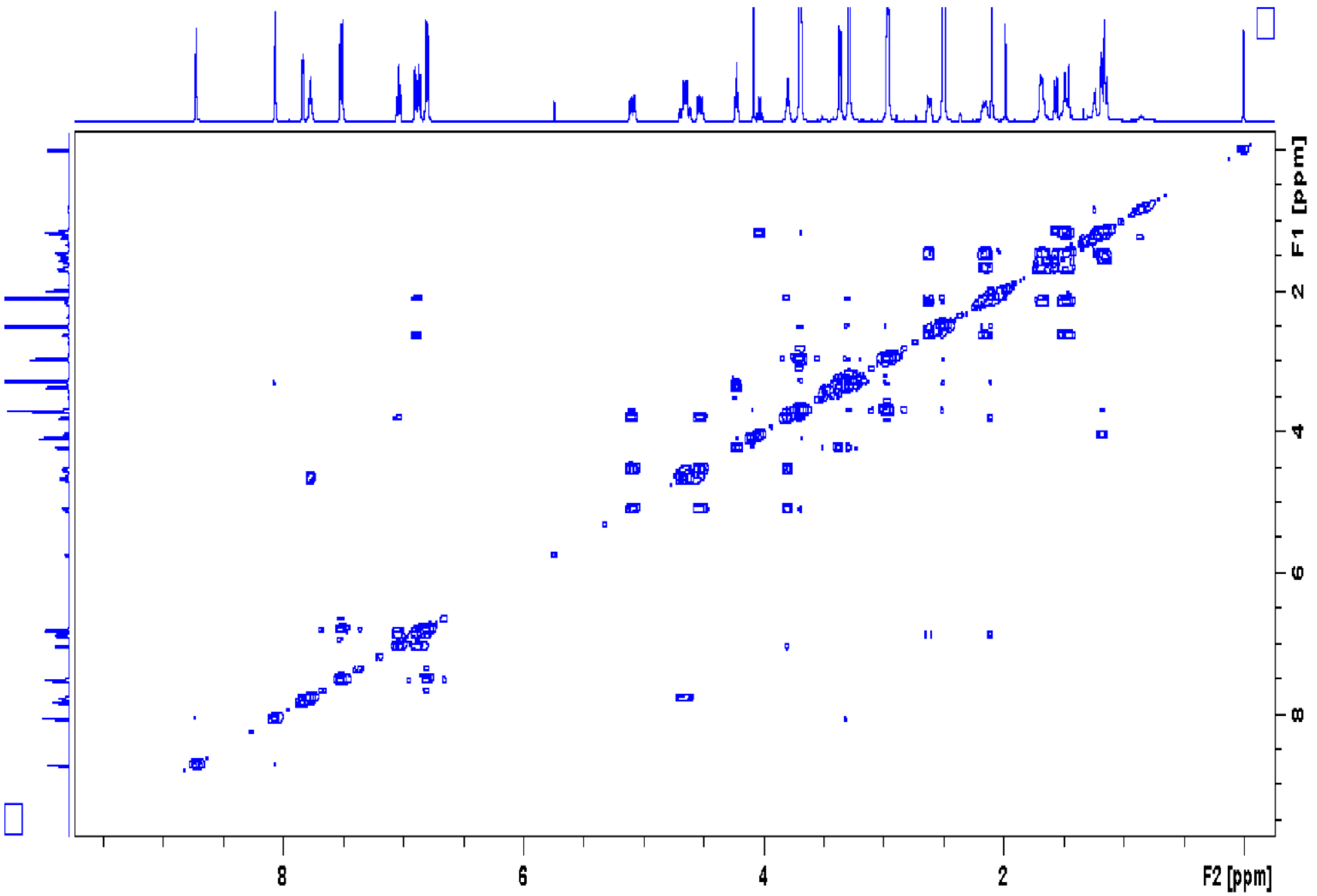

Figure S129. NOESY-NMR of compound **34**

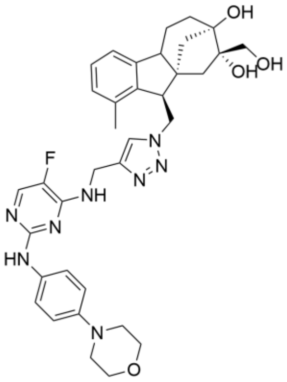

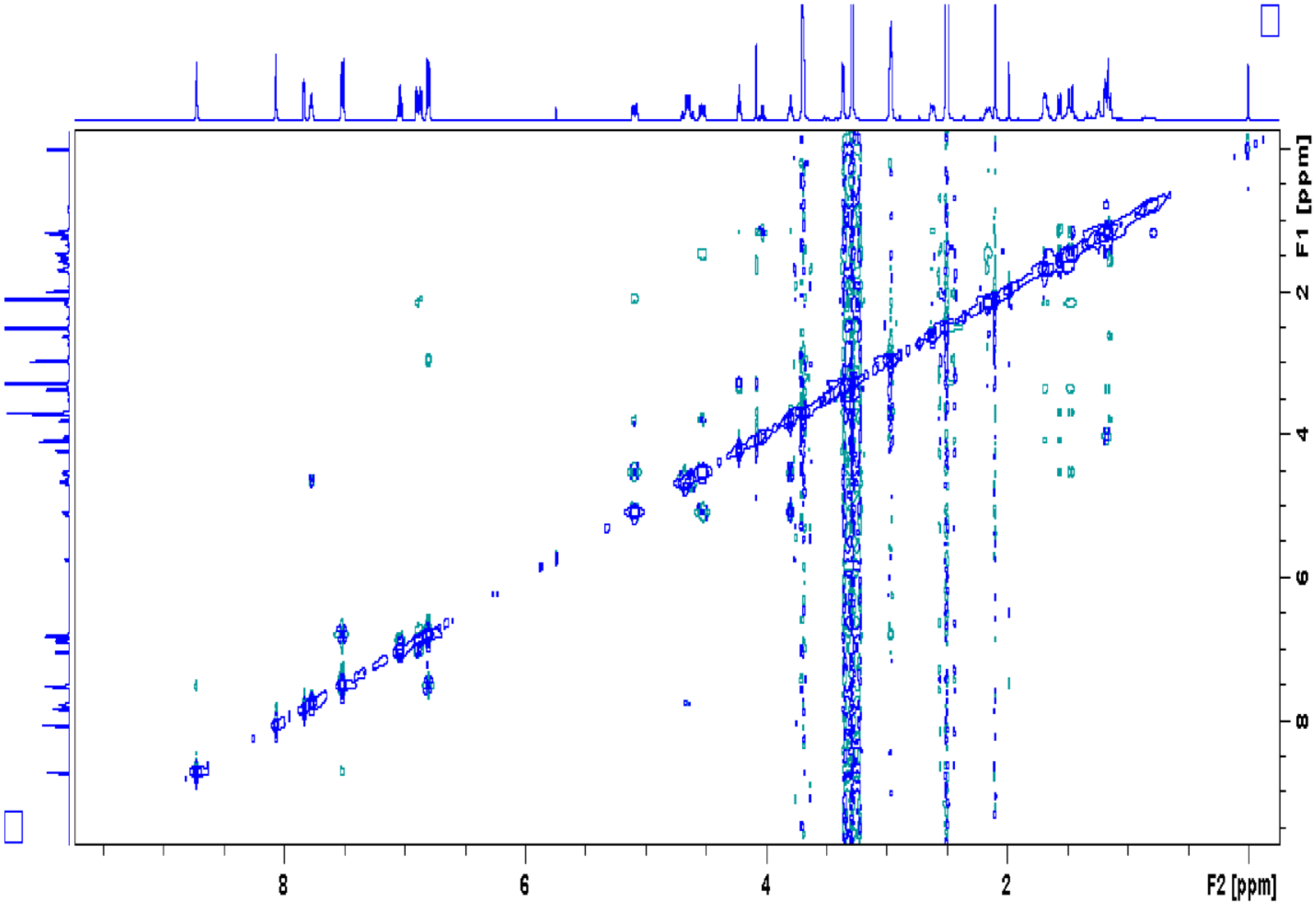

Figure S130. HSQC-NMR of compound **34**

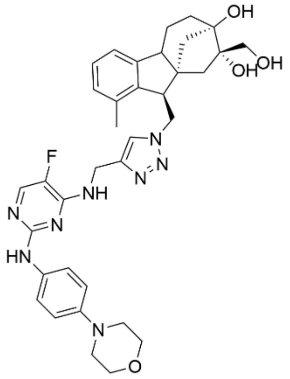

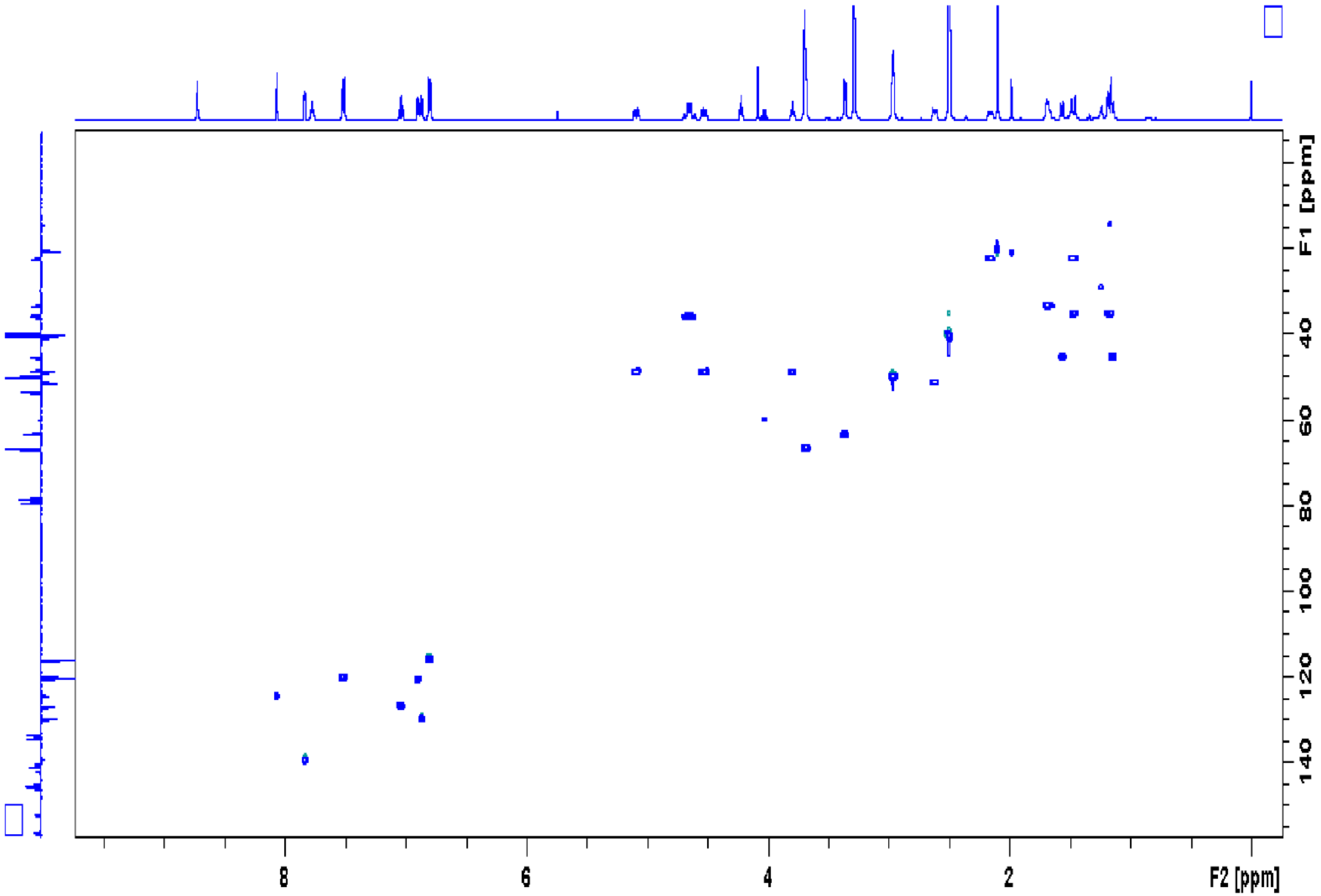

Figure S131. HMBC-NMR of compound **34**

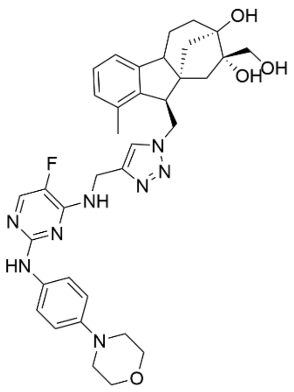

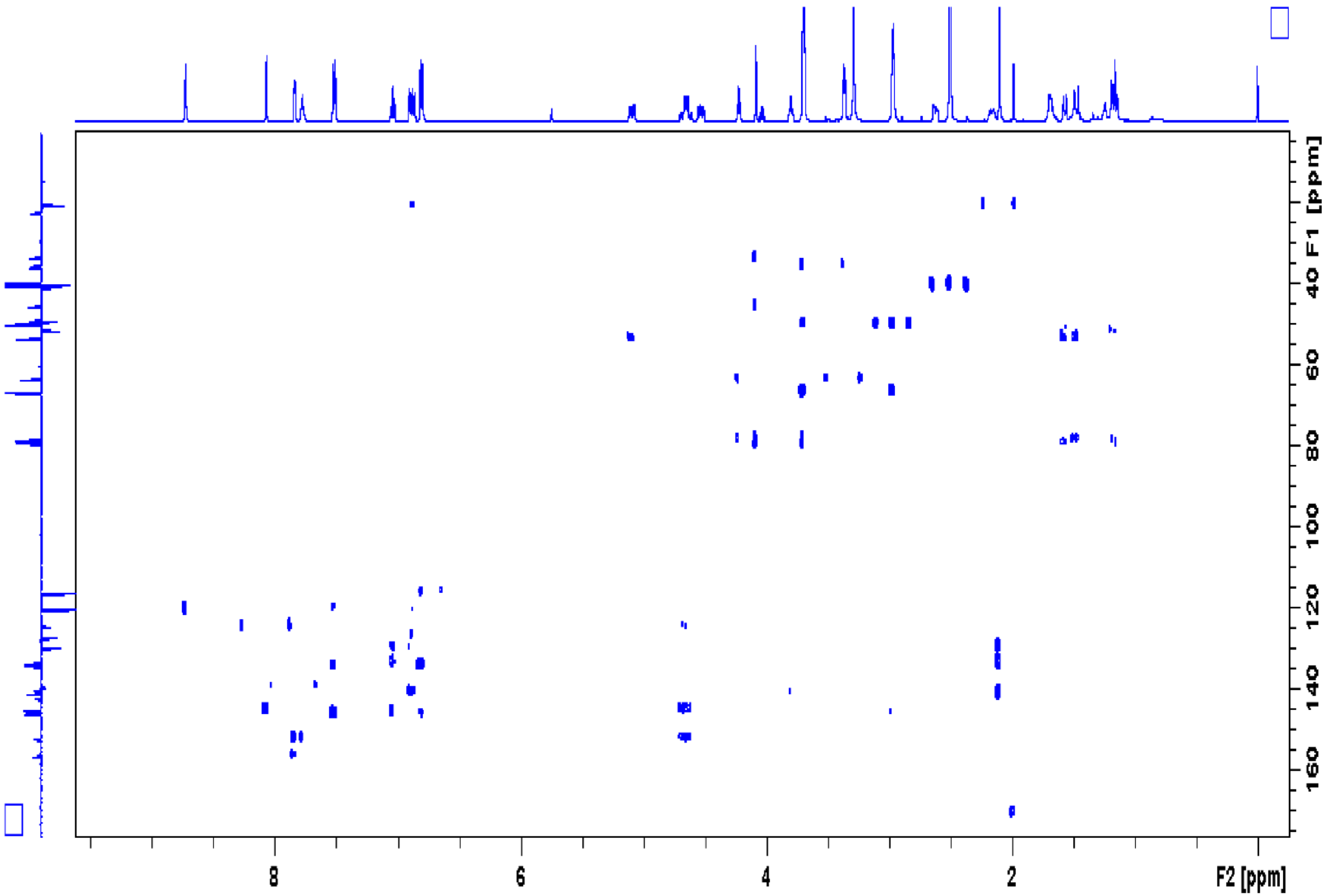

Docking study

Table S1. Binding affinities of the re-docked co-crystallized ligand and the RMSD of the top 5 generated poses relative to the original pose of the co-crystallized ligand

Pose	Affinity kcal/mol	RMSD (A°)	Reference
output-ligand_model_0 1	**-8.2**	**2.5339**	co-crystallized Ligand
output-ligand_model_0 2	**-8.2**	**2.6378**	co-crystallized Ligand
output-ligand_model_0 3	**-7.9**	**2.1259**	co-crystallized Ligand
output-ligand_model_0 4	**-7.5**	**1.0276**	co-crystallized Ligand
output-ligand_model_0 5	**-7.5**	**2.0849**	co-crystallized Ligand

Figure S132. 2D-diagram of the interactions between the re-docked co-crystallized ligand and *P. aeruginosa* DNA gyrase (PDB code: 6M1S)

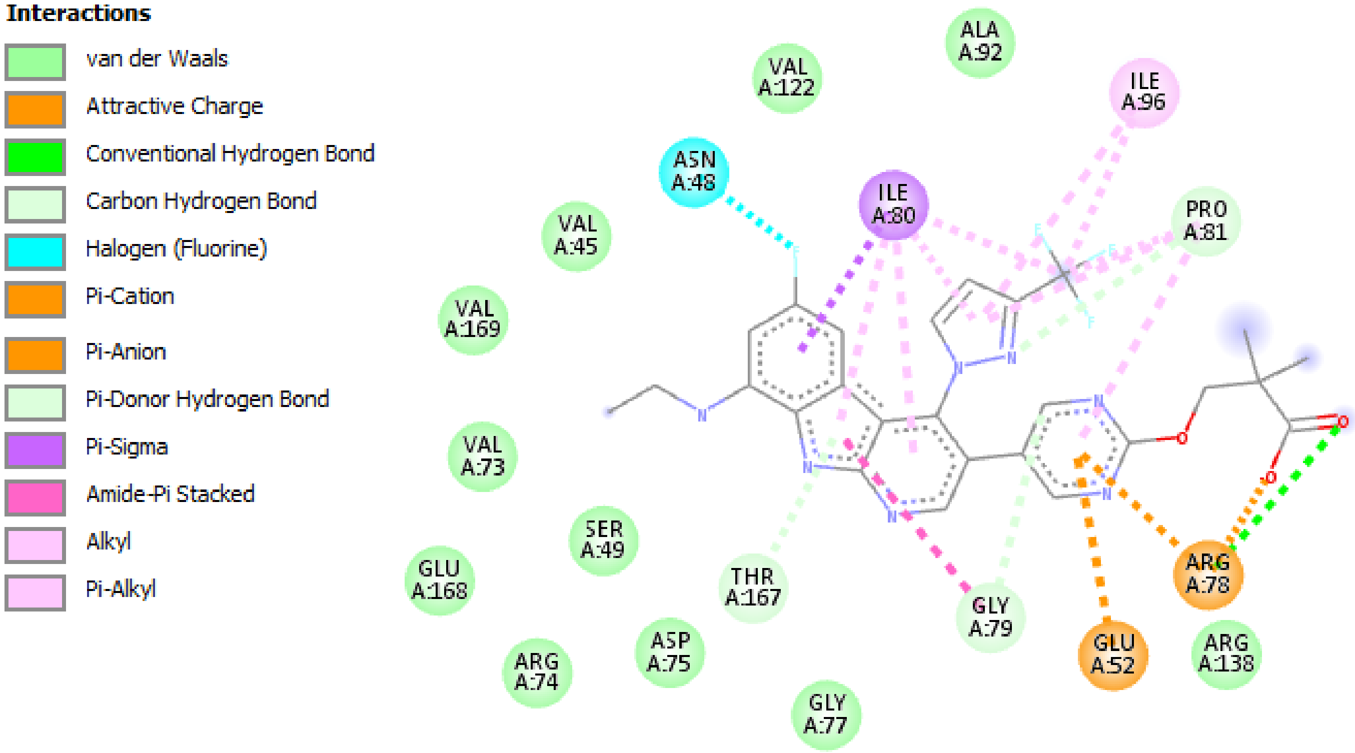

Table S2. Binding affinities of docking compound **20** with *P. aeruginosa* DNA gyrase (PDB code: 6M1S)

**Pose**	**Affinity kcal/mol**
1	-8.9
2	-8.8
3	-8.6
4	-8.6
5	-8.6
6	-8.6
7	-8.6
8	-8.2
9	-8.2

Figure S133. 2D-diagram showing interactions between the best pose of compound **20** and *P. aeruginosa* DNA gyrase (PDB code: 6M1S)

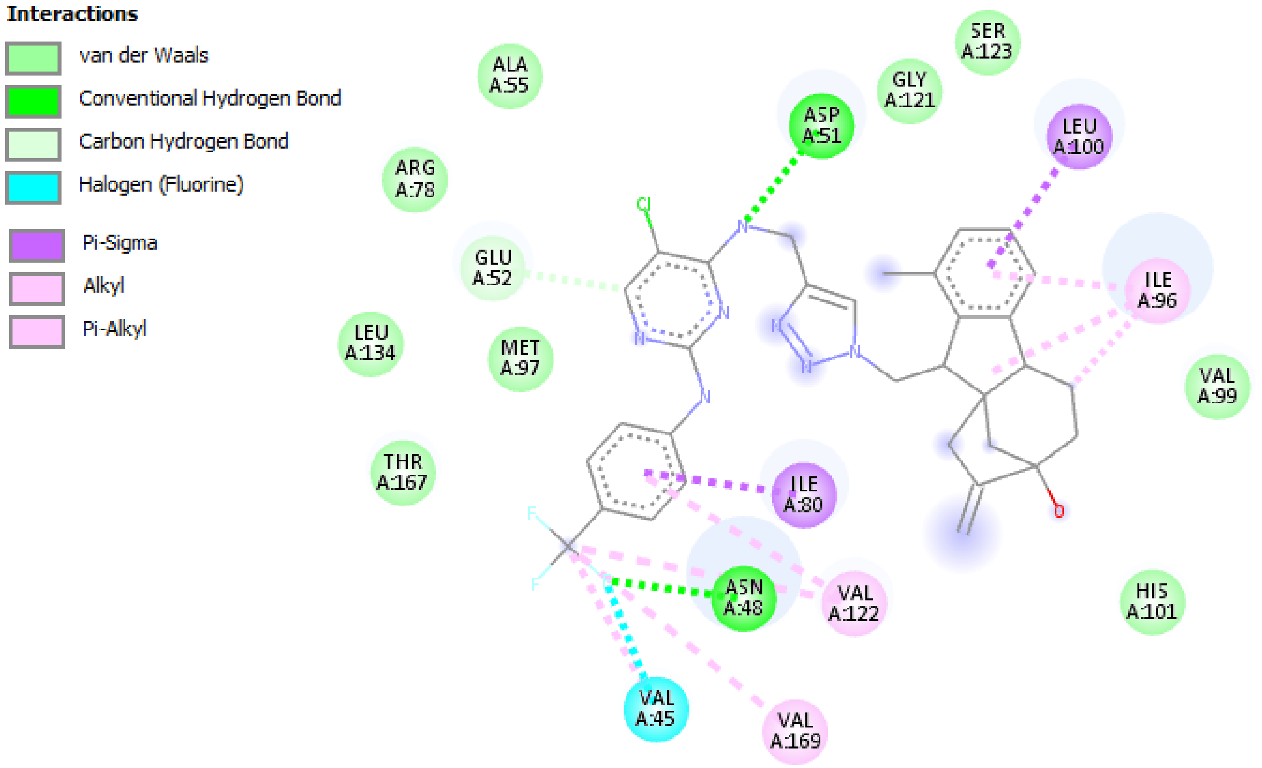

Table S3. Binding affinities of docking compound **29** with *P. aeruginosa* DNA gyrase (PDB code: 6M1S)

**Pose**	**Affinity kcal/mol**
1	-9.0
2	-8.8
3	-8.6
4	-8.5
5	-8.4
6	-8.4
7	-8.2
8	-8.2
9	-8.1

Figure S134. 2D-diagram showing interactions between the best pose of compound **29** and *P. aeruginosa* DNA gyrase (PDB code: 6M1S)

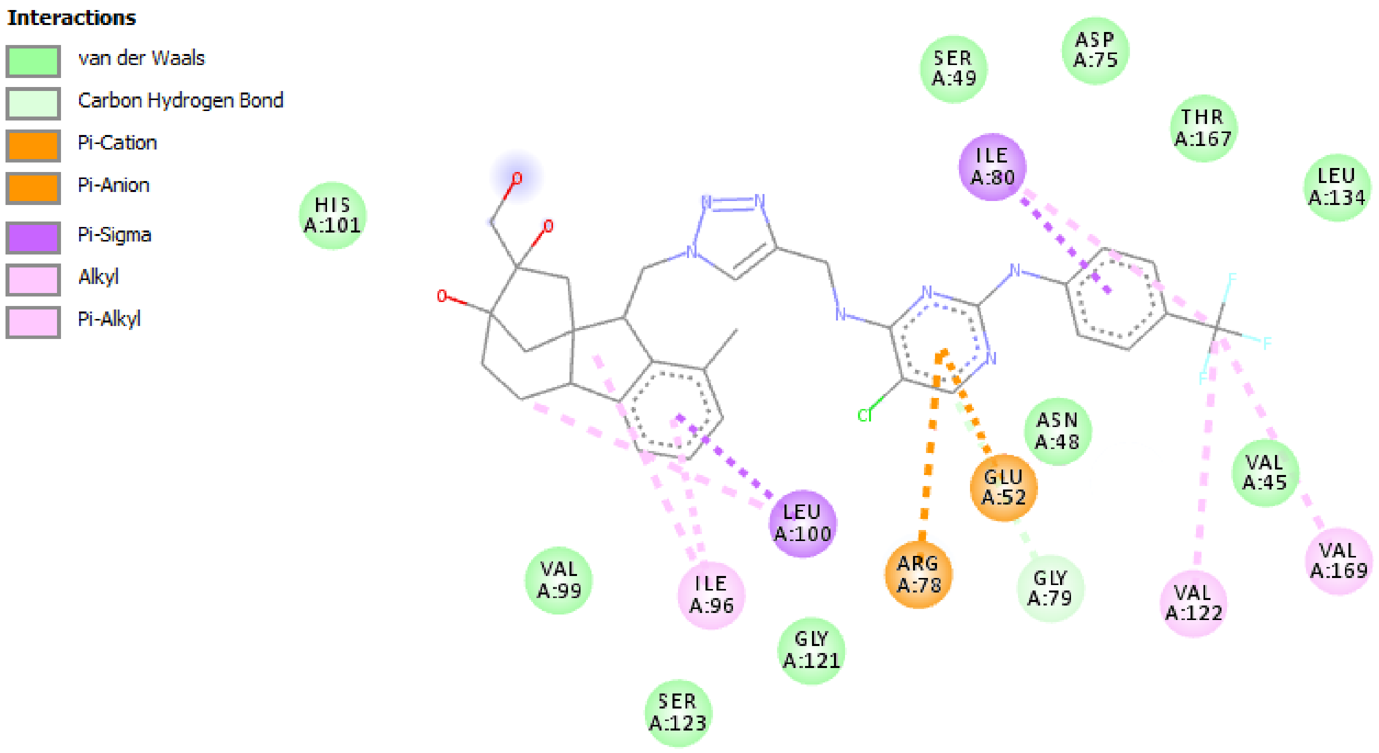

Table S4. Binding affinities of docking compound **30** with *P. aeruginosa* DNA gyrase (PDB code: 6M1S)

**Pose**	**Affinity kcal/mol**
1	-7.4
2	-7.3
3	-7.2
4	-7.2
5	-6.9
6	-6.8
7	-6.8
8	-6.8
9	-6.8

Figure S135. 2D-diagram showing interactions between the best pose of compound **30** and *P. aeruginosa* DNA gyrase (PDB code: 6M1S)

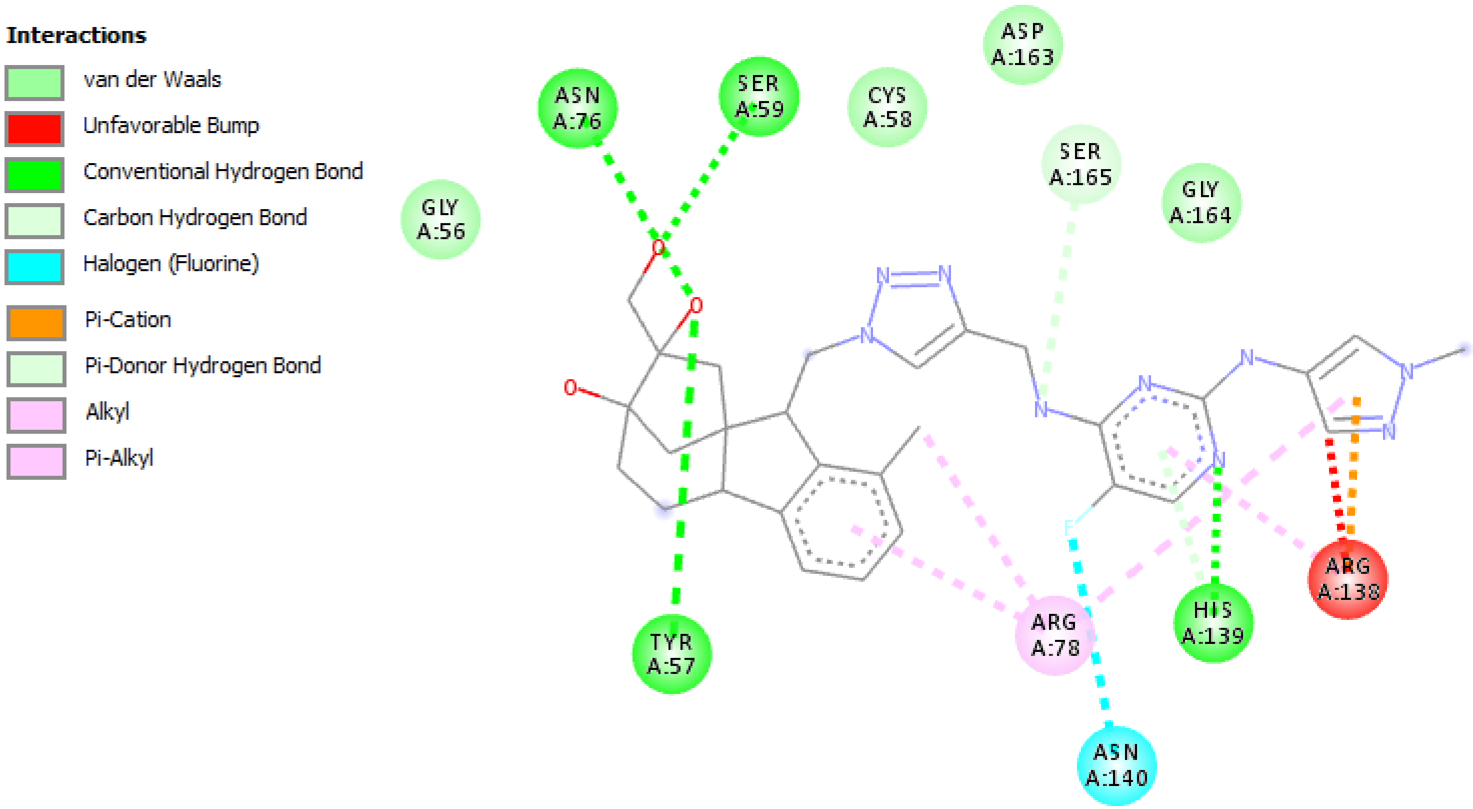

Figure S136. 3D presentation showing the binding location and accumulated poses predicted by the software of the original ligand and *P. aeruginosa* DNA gyrase (PDB code: 6M1S).

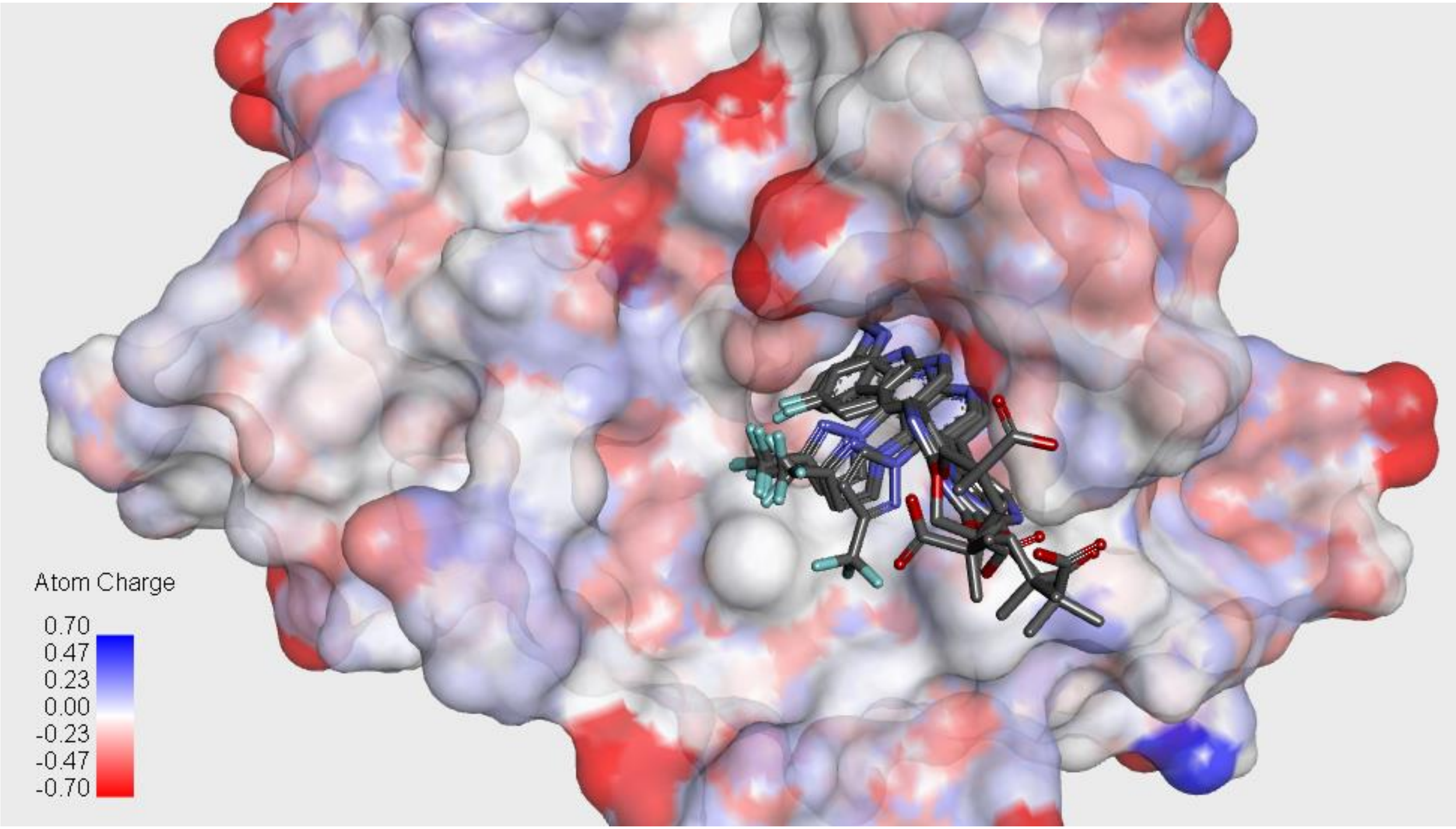

Figure S137. 3D presentation showing the binding location and accumulated poses predicted by the software of compounds **20** and *P. aeruginosa* DNA gyrase (PDB code: 6M1S).

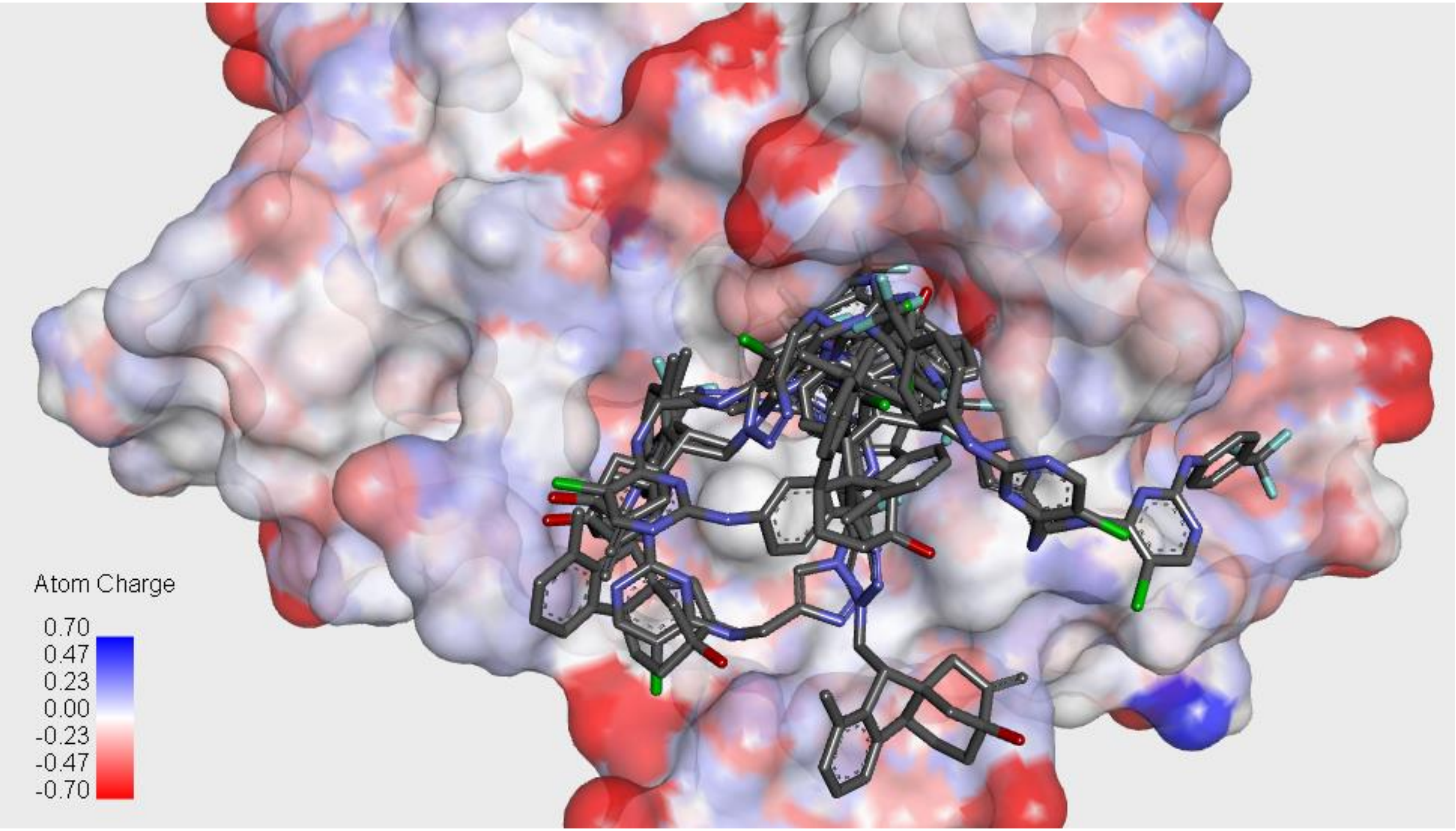

Figure S138. 3D presentation showing the binding location and accumulated poses predicted by the software of compounds **29** and *P. aeruginosa* DNA gyrase (PDB code: 6M1S).

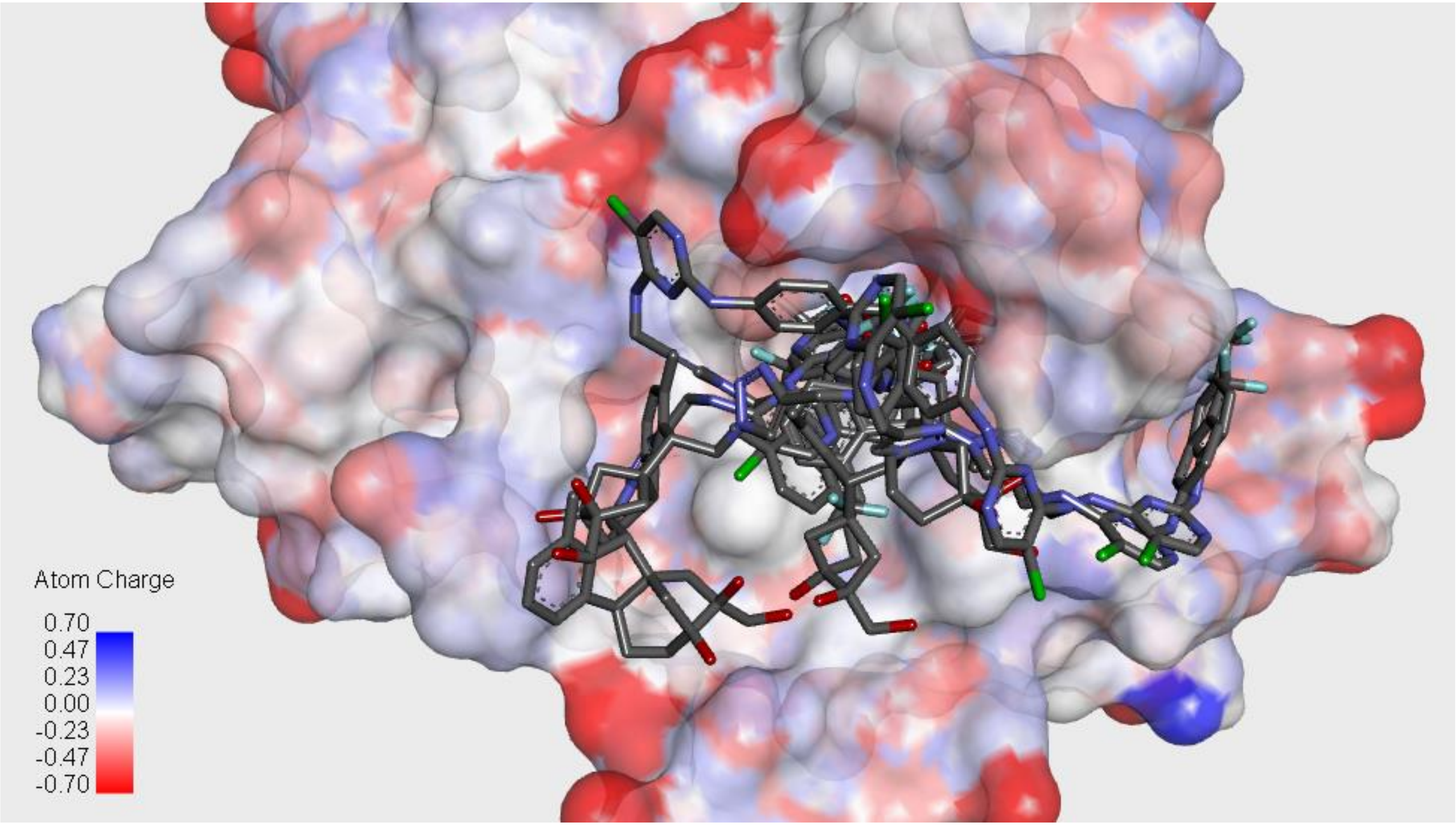

Figure S139. 3D presentation showing the binding location and accumulated poses predicted by the software of compounds **30** and *P. aeruginosa* DNA gyrase (PDB code: 6M1S).

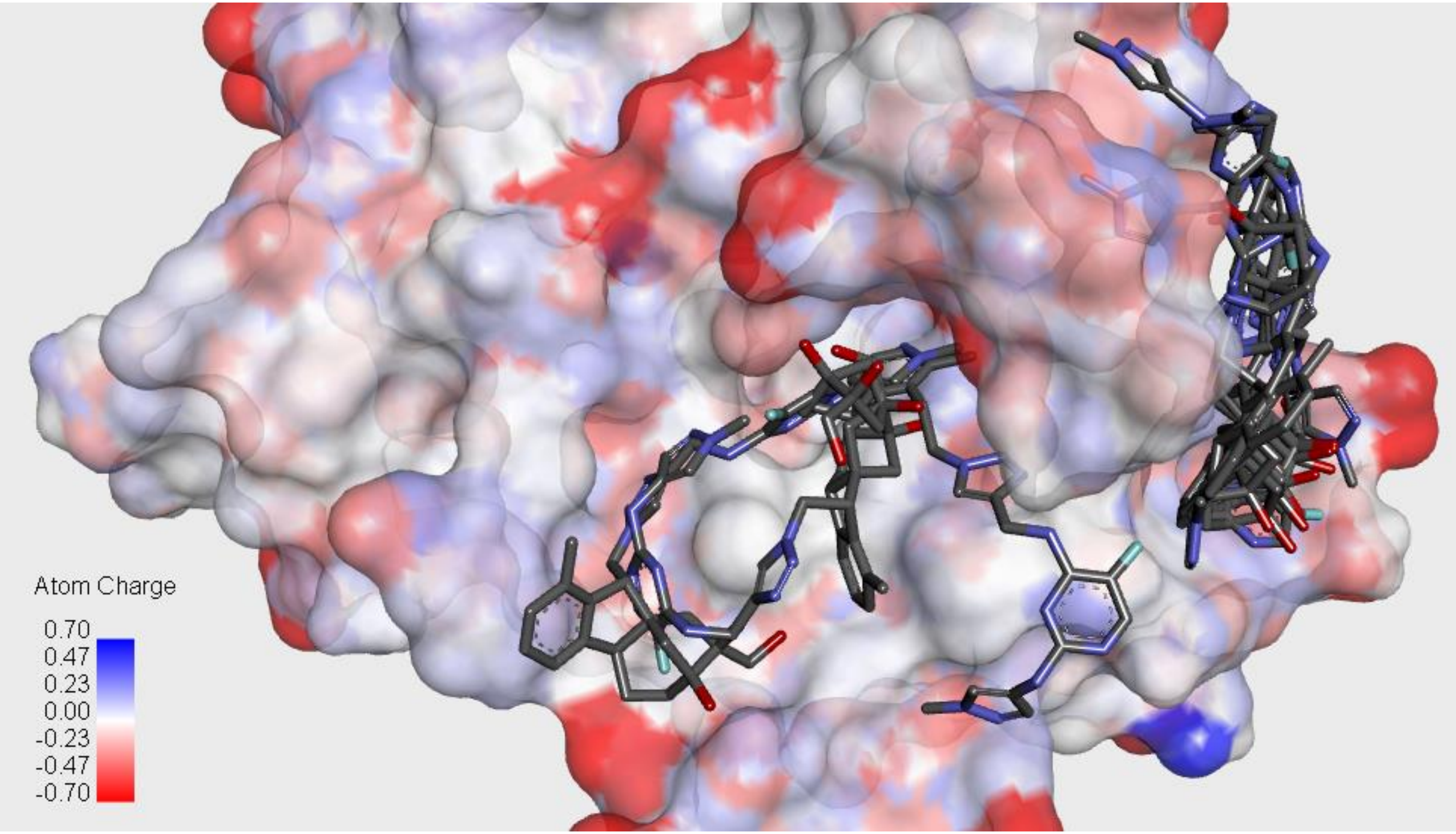

Supplement: Supplementary file 1 [file pharmaceuticals-18-00168-s001.zip › pharmaceuticals-3442446-supplementary.pdf]
